# Supplementary material for: The AhR‐SRC axis as a therapeutic vulnerability in BRAFi‐resistant melanoma
Source: EMBO Mol Med. 2022 Oct 28;14(12):e15677. doi: 10.15252/emmm.202215677 (PMC9728058; doi:10.15252/emmm.202215677)
Supplement: Supplementary file 1 — Appendix [file EMMM-14-e15677-s007.pdf]

Table of content

Appendix Figure S1. The AhR signature correlates with the invasive and dedifferentiated phenotype of melanoma cell lines

Appendix Figure S2. Identification of genes involved in "phenotype switching" that are regulated by the AhR transcription factor

Appendix Figure S3. AhR target gene expression correlates with cell-dedifferentiation states and resistance to BRAFi in patient tumors

Appendix Figure S4. Cross-regulation of genes after induction by ITE or specific inhibition of SRC by dasatinib

Appendix Figure S5. AhR participates in the activation of oncogenic signatures associated with BRAFi resistance

Appendix Figure S6. AhR regulates the expression of specific genes involved in SRC/FAK activation, promoting the focal adhesion pathway during the acquisition of BRAFi resistance

Appendix Figure S7. Correlation of AhR expression with drug efficiency in tumors

Appendix Figure S8. Comparison of the correlation of gene expression signatures with drug efficiency and AhR expression in tumors

Appendix Figure S9. Inhibition of SRC sensitizes melanoma to BRAFi treatment in a PDX model (in complement to Figure 6)

Appendix Table S1. List of genes for different signatures

Appendix Table S2. Description of target genes

Appendix Table S3. Correlation of dasatinib efficiency with gene expression level (Z-score)

Appendix Table S4. List of primers

Appendix Table S5. List of antibodies

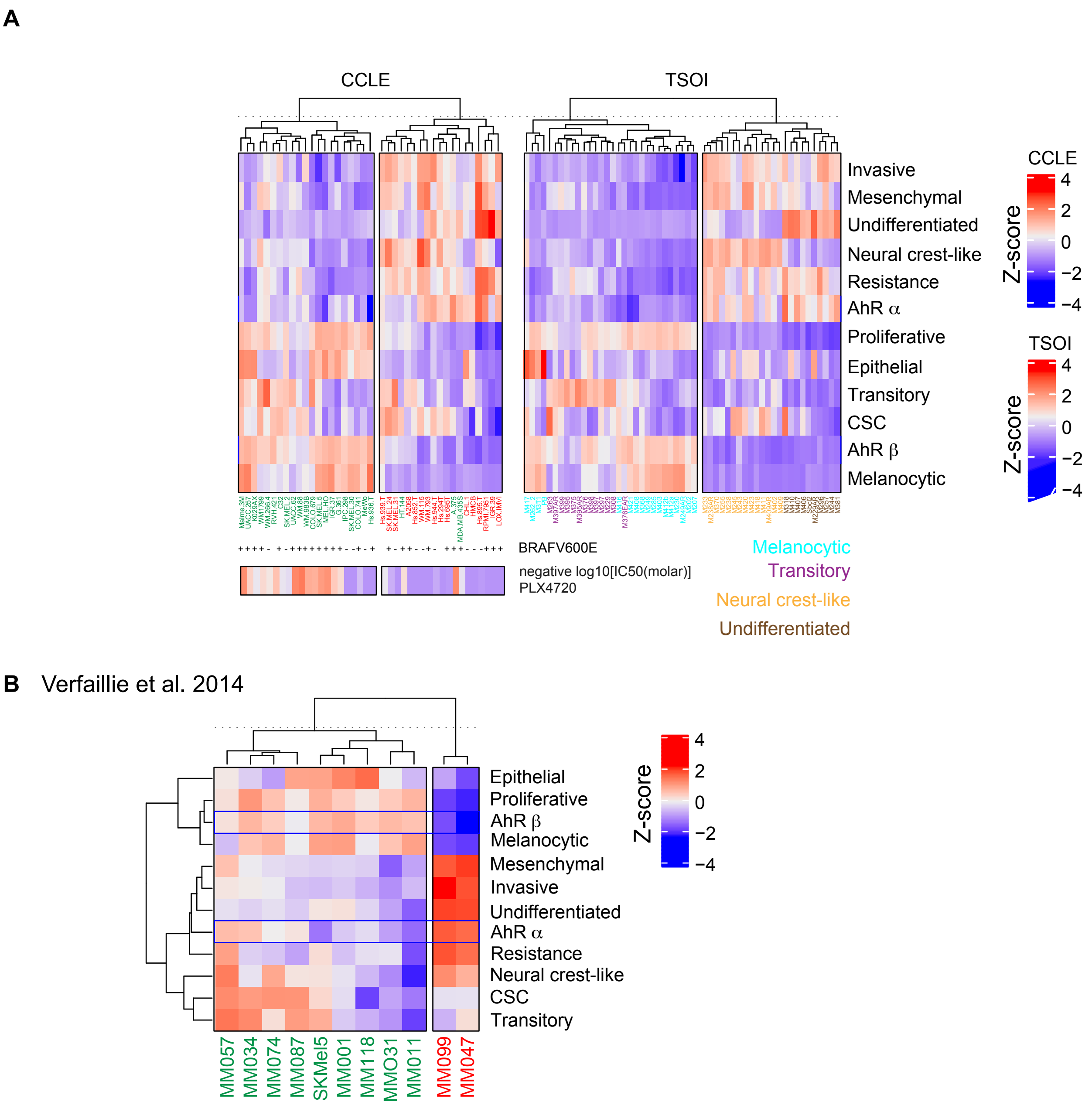

Appendix Figure S1. The AhR signature correlates with the invasive and dedifferentiated phenotype of melanoma cell lines.

A. Expression heatmap for various gene signatures (Table EV1) in different Vem-sensitive (green) or resistant melanoma cell lines (red) from the Cancer Cell Line Encyclopedia RNAseq (CCLE) dataset (Barretina et al. 2012) and the melanoma cell line dataset from the GEO dataset (GSE80824 (Tsoi et al. 2018)), depending on their differentiation status, consistent with the four-stage differentiation model (melanocytic: M, transitory: T, neural-crest like: and N: undifferentiated) established by Tsoi et al. (Tsoi et al. 2018). Genes and clusters with similar expression profiles across the cohort are placed close to each other in the grid. The scale corresponds to the Z scores.

B. Expression heatmap for various gene signatures in the melanoma cell line dataset from the GEO dataset (GSE60664 (Verfaillie et al. 2015)) depending on their proliferative (green) or invasive states (red). The scale corresponds to the Z scores.

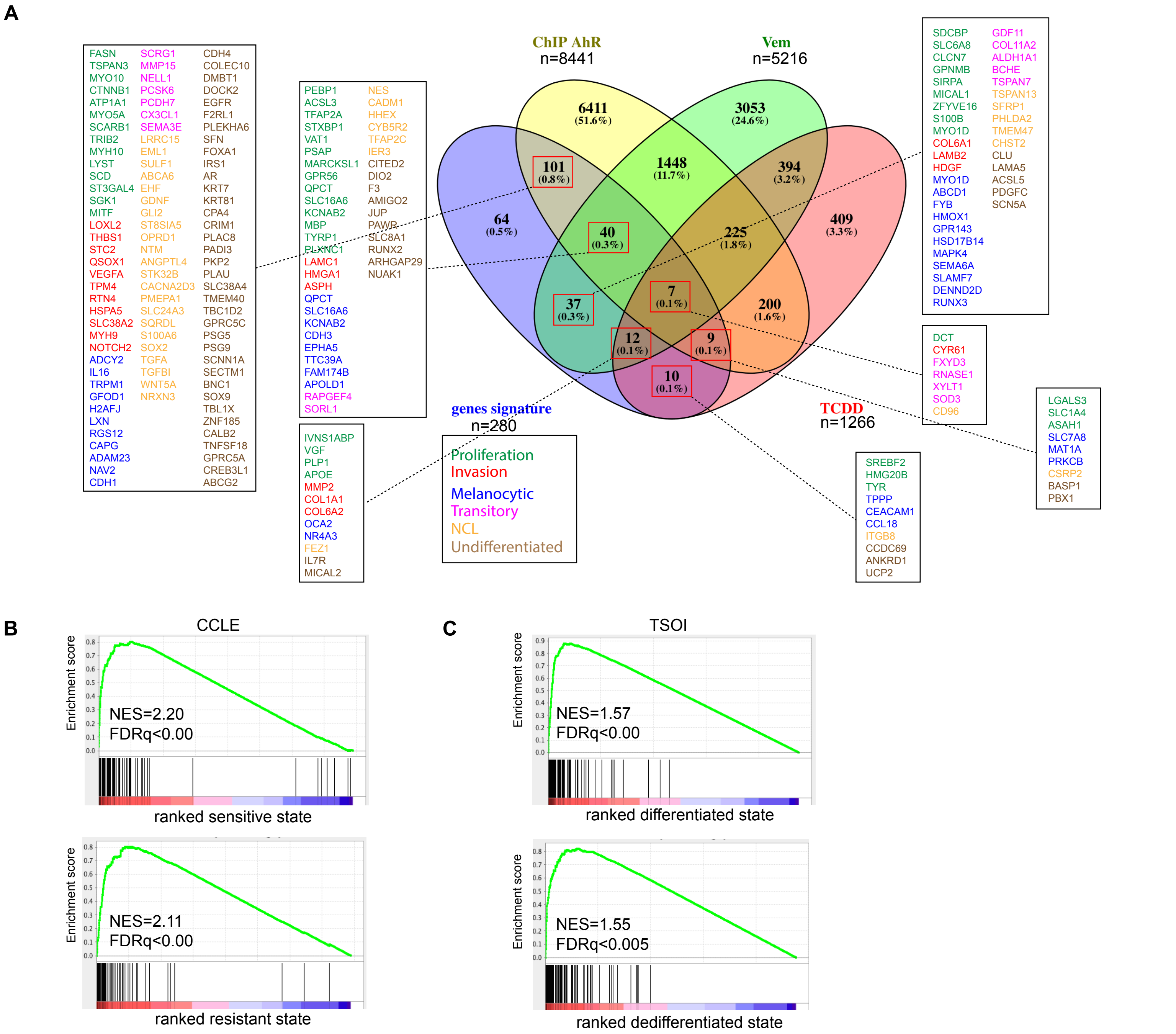

Appendix Figure S2. Identification of genes involved in “phenotype switching” that are regulated by the AhR transcription factor.

A. Venn diagram representing the overlap between the genes corresponding to thevarious signatures between genes (n = 280) that are differentially expressed in 501Mel cells exposed to vehicle, Vem (1 µM) (n = 5,216) or TCDD (10 nM) (n = 1,266) for 48h (GSE104869 (Corre et al. 2018)) and AhR target genes identified by ChIPseq experiments performed by the laboratory of Mathews for AhR transcription factor 45 min to 24 h after induction by TCDD (10 nM) of MCF7 cells (n = 8,441) (GSE90550 (S. Y. Yang et al. 2018)).

B-C. GSEA showing that selected gene sets are enriched (B) in both BRAFi-sensitive (top) and resistant melanoma (bottom) cell lines from the Cancer Cell Line Encyclopedia RNAseq dataset (GEO, GSE36134 (Barretina et al. 2012)) and in (C) the differentiation (melanocytic or transitory) (top) or dedifferentiation (neural crest-like or undifferentiated) (bottom) states of melanoma cell lines from the melanoma cell lines dataset of the GEO dataset (GSE80824 (Tsoi et al. 2018)) established by Tsoi et al.

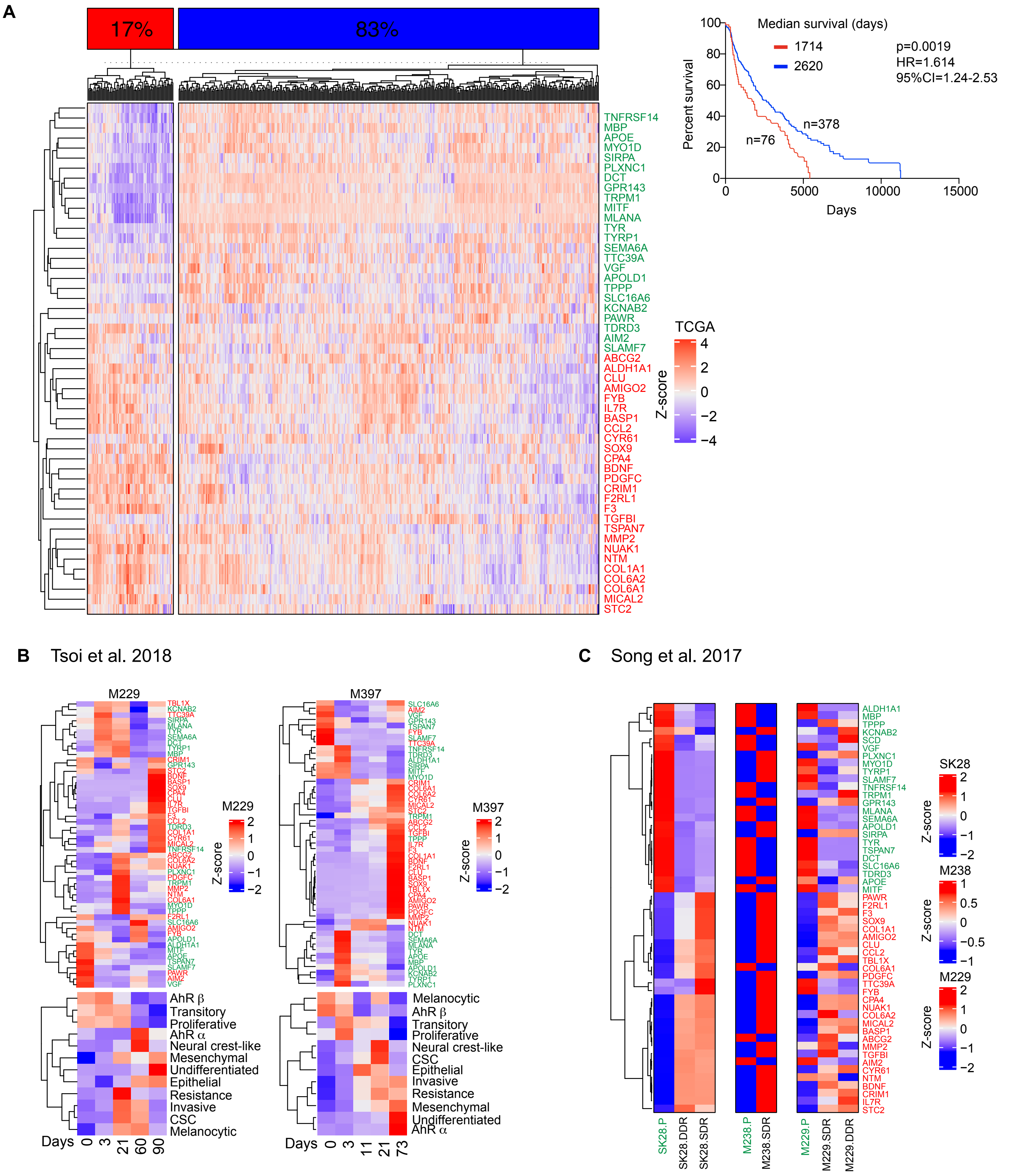

Appendix Figure S3. AhR target gene expression correlates with cell-dedifferentiation states and resistance to BRAFi in patient tumors

A. Expression heatmap depicting mRNA expression for the most highly enriched genes in the sensitive/differentiation (n = 25) or resistant/dedifferentiation (n = 25) states of melanoma cell lines from the non-treated melanoma patient dataset of the TCGA (SKCM, n = 459) (Barretina et al. 2012). Determination of the overall survival curves by Kaplan-Meier analysis of the two subpopulations (red or blue) (from the TCGA SKCM dataset). Cox regression was used to calculate p values, hazard ratios (HR), and 95% confidence intervals (CI).

B. Expression heatmap for various genes in cell lines (M229, M397) recovered at different times after BRAFi treatment (0 to 90 days) during the dedifferentiation process during the acquisition of resistance (GEO, GSE110054 (Tsoi et al. 2018)).

C. Expression heatmap for various genes in parental (P) cell lines (SK28, M238 and M229) and long-term sub-lines (months to years on BRAFi or BRAFi+MEKi: PLX4032+selumitinib) resulting in single-drug resistant (SDR) or double-drug resistant (DDR) lines. (GEO, GSE75299 (Song et al. 2017)). The SDR and DDR sub-lines were derived as described (Nazarian et al. 2010; Moriceau et al. 2015). The scale corresponds to the Z scores.

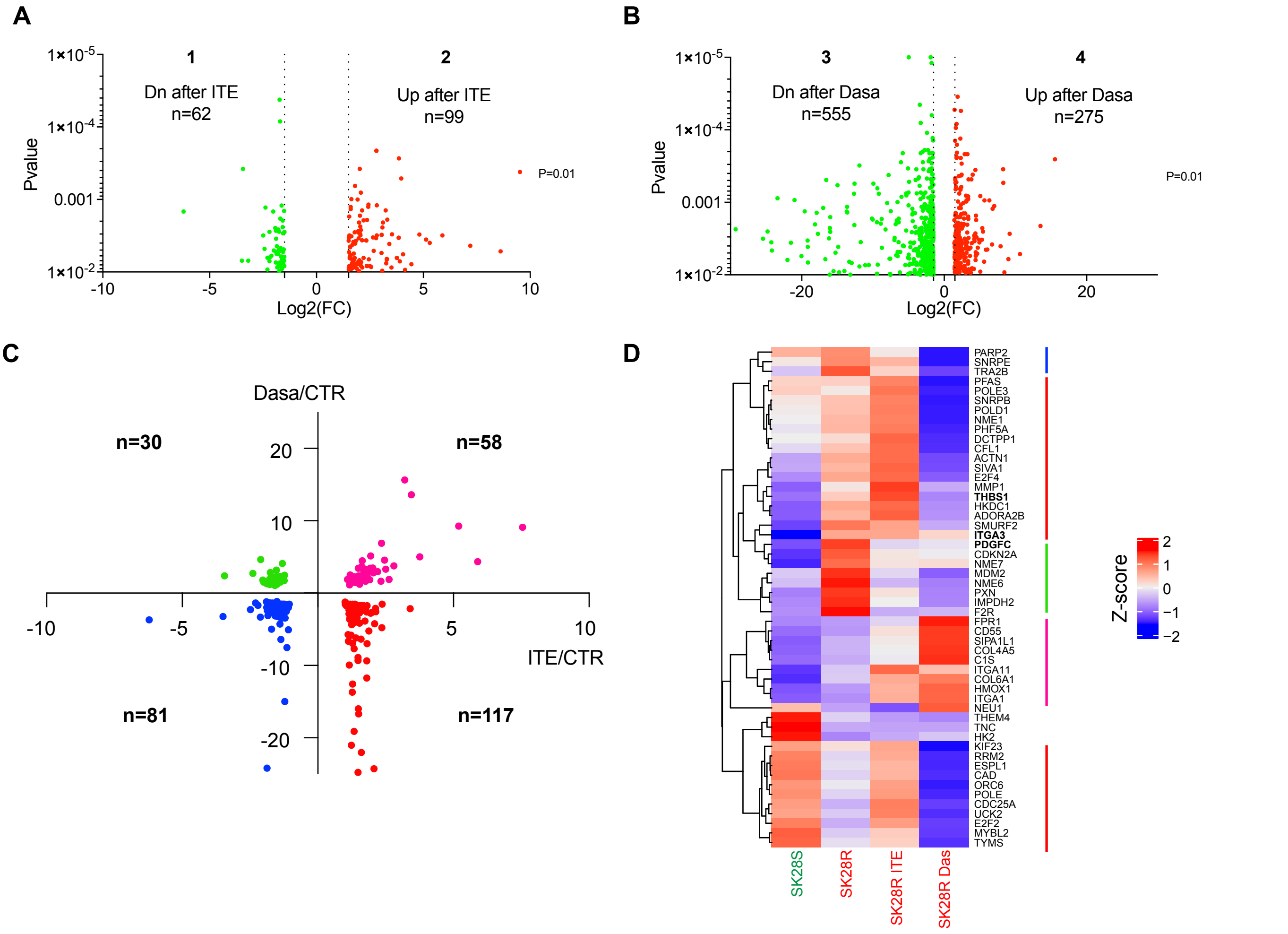

Appendix Figure S4. Cross-regulation of genes after induction by ITE or specific inhibition of SRC by dasatinib

A. Volcano plot representing the magnitude of the fold change of significantly differentially expressed genes (Up in red = 99, Dn in green = 62, $p < 0.01$) in SK28R cells after 24 h of treatment or not with ITE (5µM).

B. Volcano plot representing the magnitude of the fold change of significantly differentially expressed genes (Up in red = 275, Dn in green = 555, $p < 0.01$) in SK28R cells after 24 h of treatment or not with dasatinib (1 µM).

C. Dot blot showing common DEG in SK28R cells after ITE or dasatinib treatment.

D. Expression heatmap for the previously identified DEG. The scale corresponds to the Z scores.

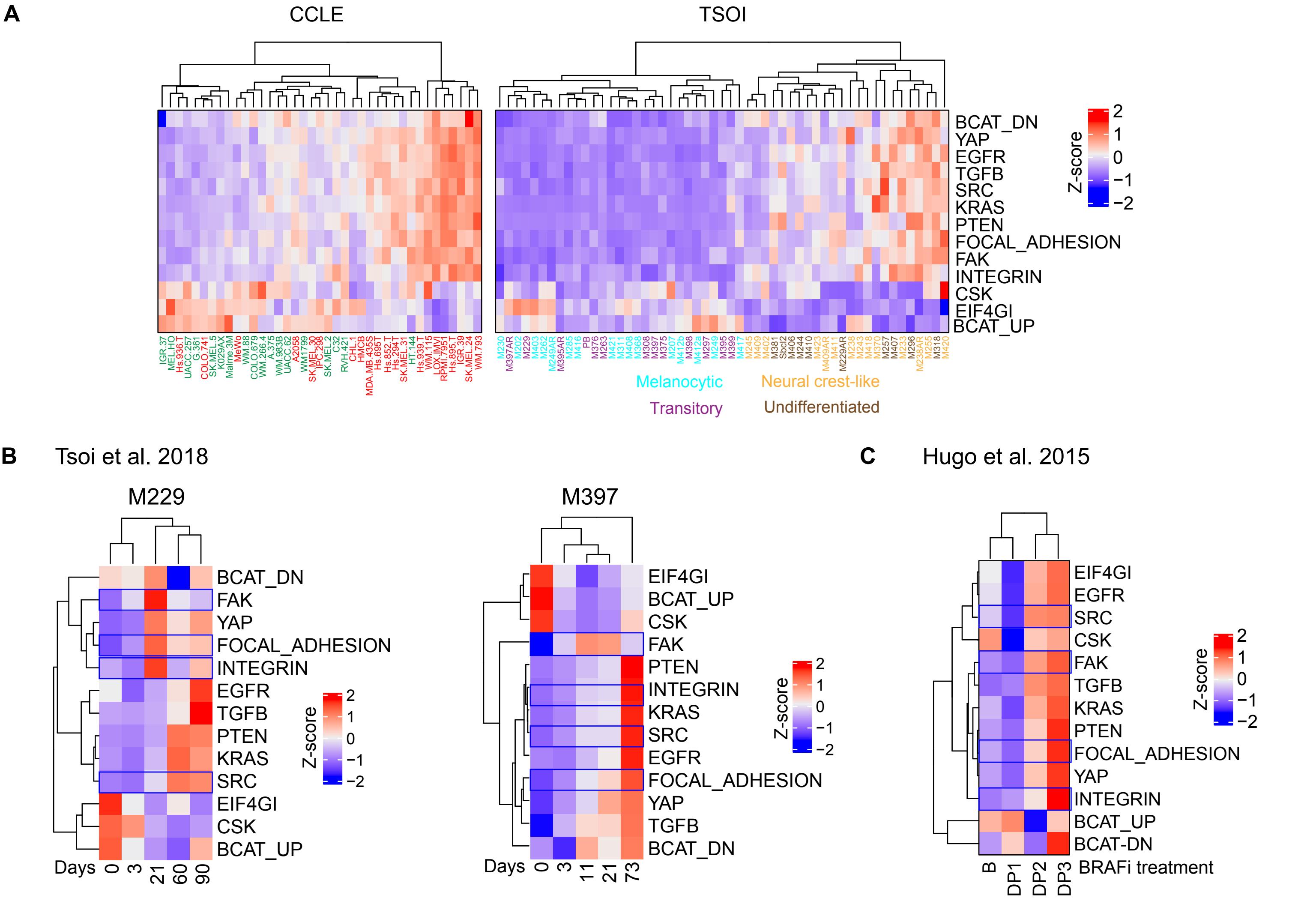

Appendix Figure S5. AhR participates in the activation of oncogenic signatures associated with BRAFi resistance

A. Expression heatmap for oncogenic gene signatures (SRC, FAK, EGFR, YAP, KRAS, TGFβ, Integrin, etc.) see Table EV1) in the BRAFi-sensitive or resistant cell lines dataset (CCLE) or the differentiated or dedifferentiated melanoma cell lines dataset (TSOI). Genes and clusters with similar expression profiles across the cohort are placed close to each other in the grid.

B. Expression heatmap for oncogenic gene signatures in cell lines (M229, M397) recovered at different times after BRAFi treatment (0 to 90 days) and during the dedifferentiation process during the acquisition of resistance (GSE110054) (Tsoi et al. 2018).

C. Expression heatmap for oncogenic gene signatures in BRAFi-treated melanoma patients during the acquisition of resistance (GSE65185) (Hugo et al. 2015). The scale corresponds to the Z scores.

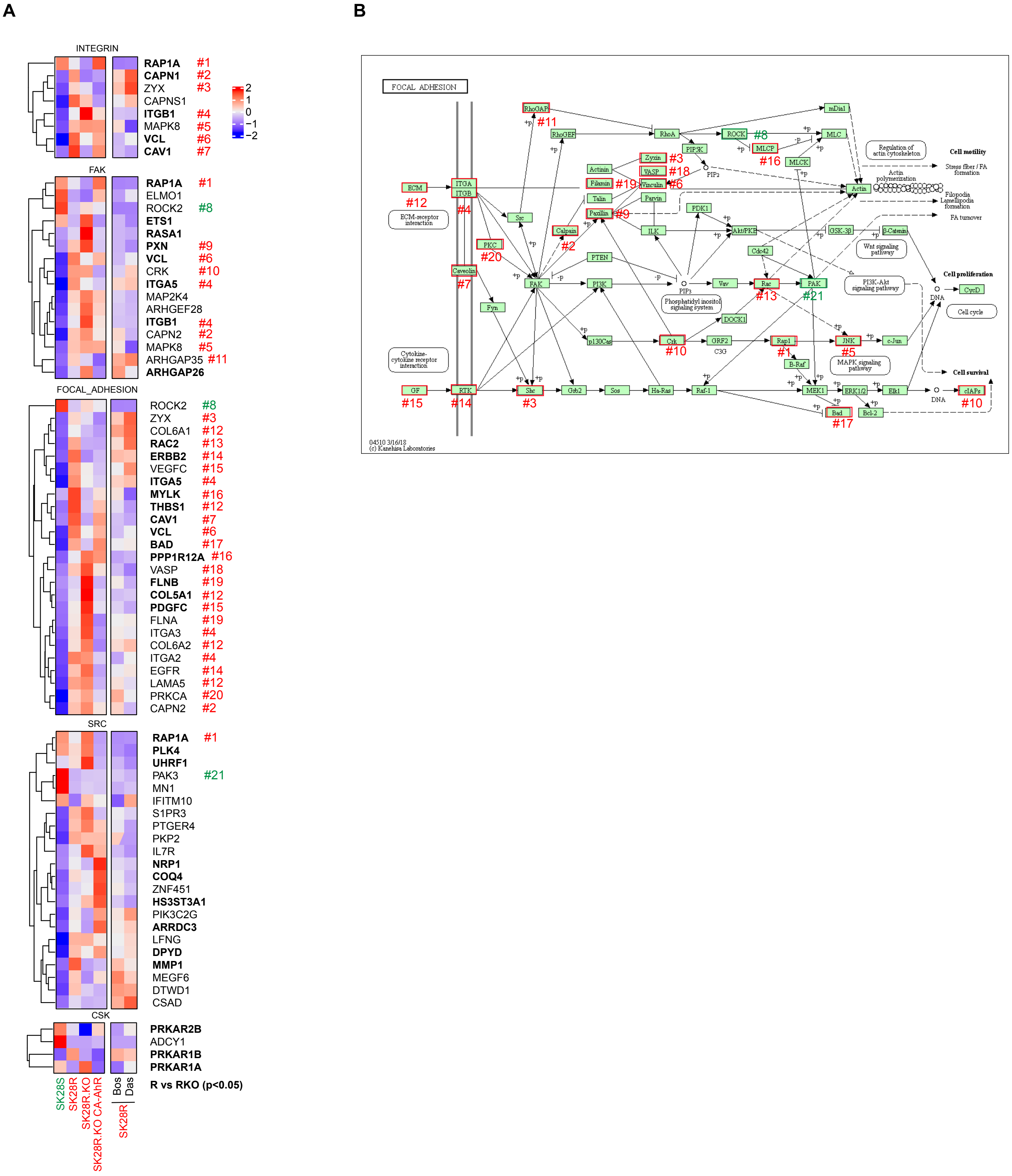

Appendix Figure S6 AhR regulates the expression of specific genes involved in SRC/FAK activation, promoting the focal adhesion pathway during the acquisition of BRAFi resistance

A. Expression heatmap for genes from various signatures (KEGG_FOCAL_ADHESION, BIOCARTA_INTEGRIN_PATHWAY, BIOCARTA_CSK_PATHWAY, SRC_UP.V1_DN, SRC_UP.V1_UP, and PID_FAK_PATHWAY see Table EV1) that were significantly differentially regulated between SK28S and R ($p < 0.01$) and between SK28 wild type and AhR KO ($p < 0.01$) (in bold).

B. Positioning of differentially expressed genes in the focal adhesion pathway https://www.genome.jp/kegg/pathway/hsa/hsa04510.html).

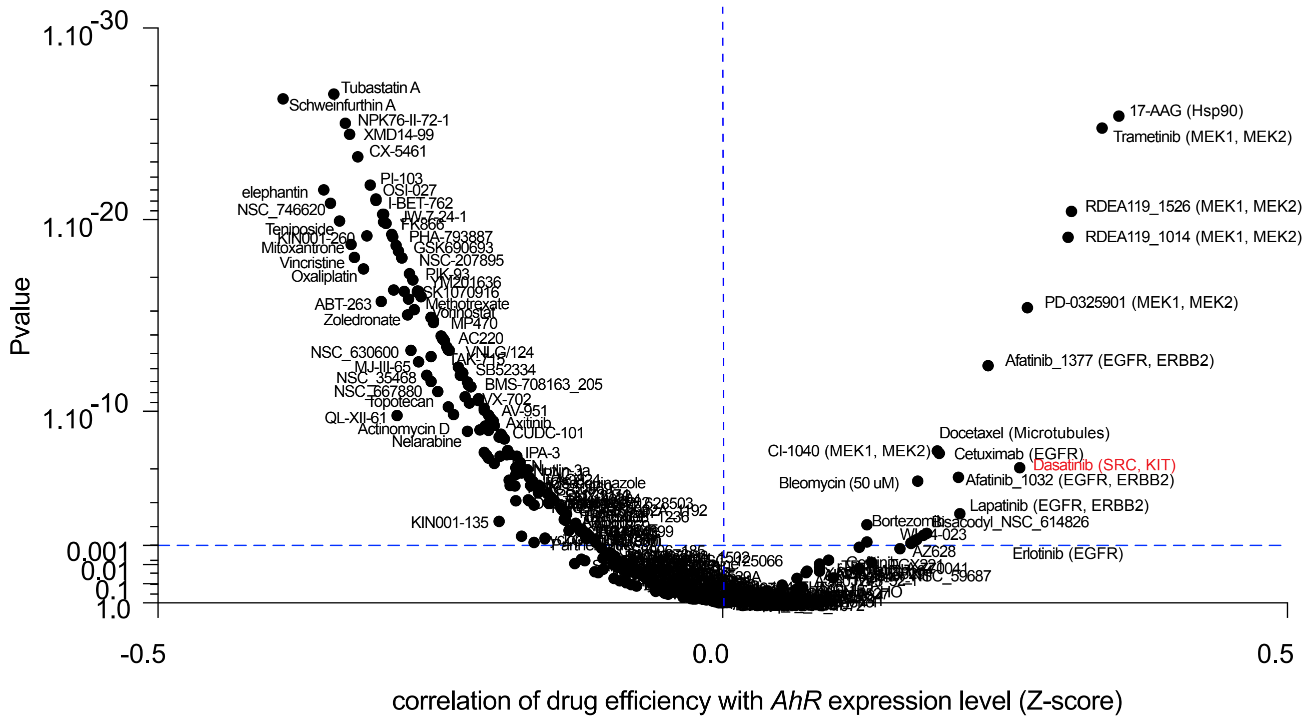

Appendix Figure S7. Correlation of AhR expression with drug efficiency in tumors.
Volcano plot representing the correlation of drug efficiency (y = p-value) (IC50 for up to 300 drugs) in all cancer cell lines (~1000) from the GDSC database (Genomics of Drug Sensitivity in Cancer) (W. Yang et al. 2013), https://discover.nci.nih.gov/cellminercdb (Reinhold et al. 2012)) with the level of AhR mRNA. The drugs that are most efficient when AhR levels are low are shown on the left of the graph and those that are most efficient when AhR levels are high are shown on the right.

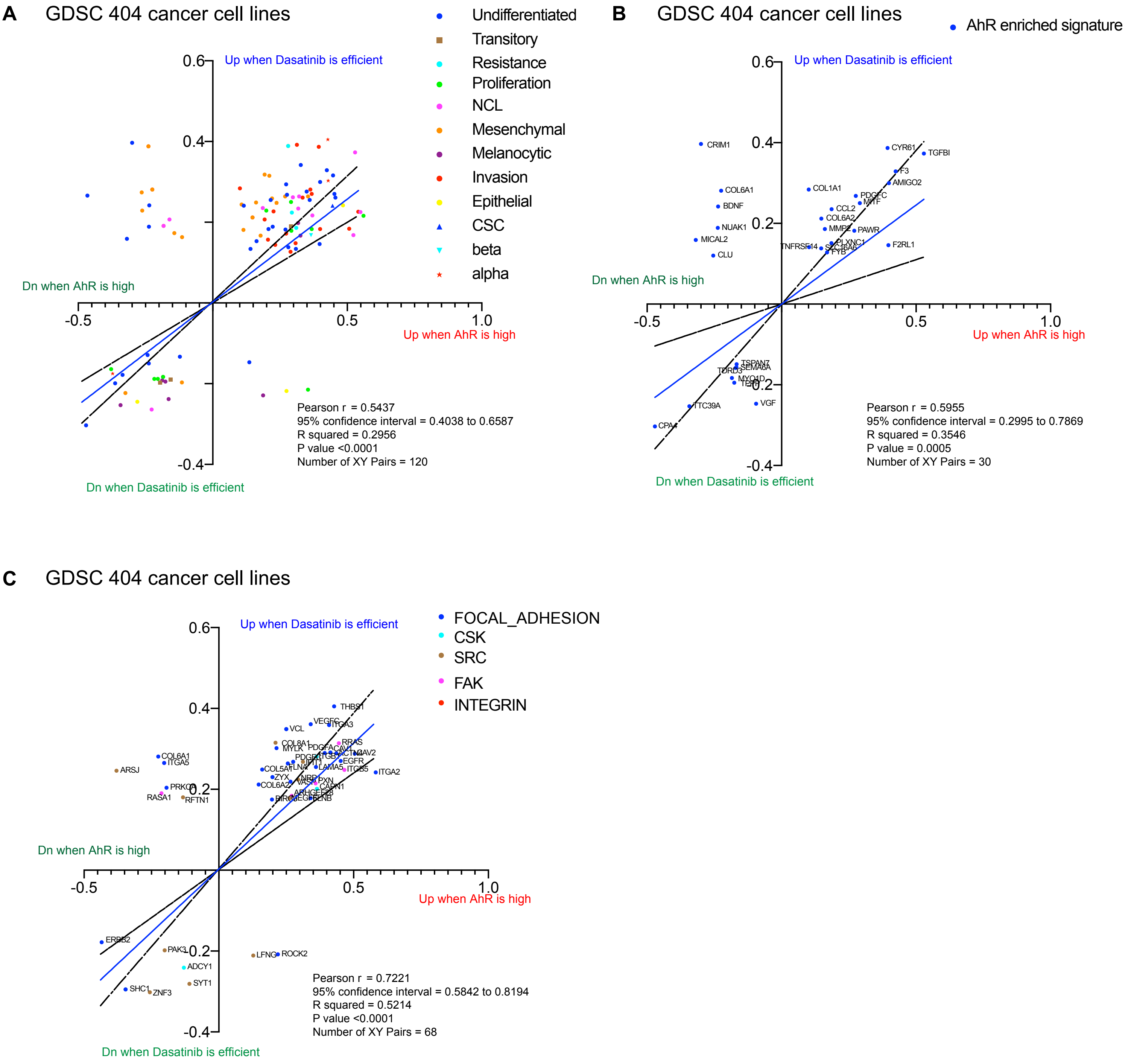

Appendix Figure S8. Comparison of the correlation of gene expression signatures with drug efficiency and AhR expression in tumors.

A. Dot blot representing the comparison of the correlation rate for dasatinib drug efficiency (IC50) in various cancer cell lines (n = 404) from the GDSC database (Genomics of Drug Sensitivity in Cancer) (W. Yang et al. 2013), https://discover.nci.nih.gov/cellminercdb (Reinhold et al. 2012)) with the normalized level of the genes for the various signatures (Fig. 2G) (y) with the correlation rate between AhR mRNA expression and gene expression (x).

B. Dot blot representing the comparison of the correlation rate for dasatinib drug efficiency with putative AhR targets involved in BRAFi resistance (Fig. 3).

C. Dot blot representing the comparison of the correlation rate for dasatinib drug efficiency with genes for the SRC, integrin, FAK, and focal-adhesion pathways. Pearson correlation analysis was performed using GraphPad PRISM 9.0®.

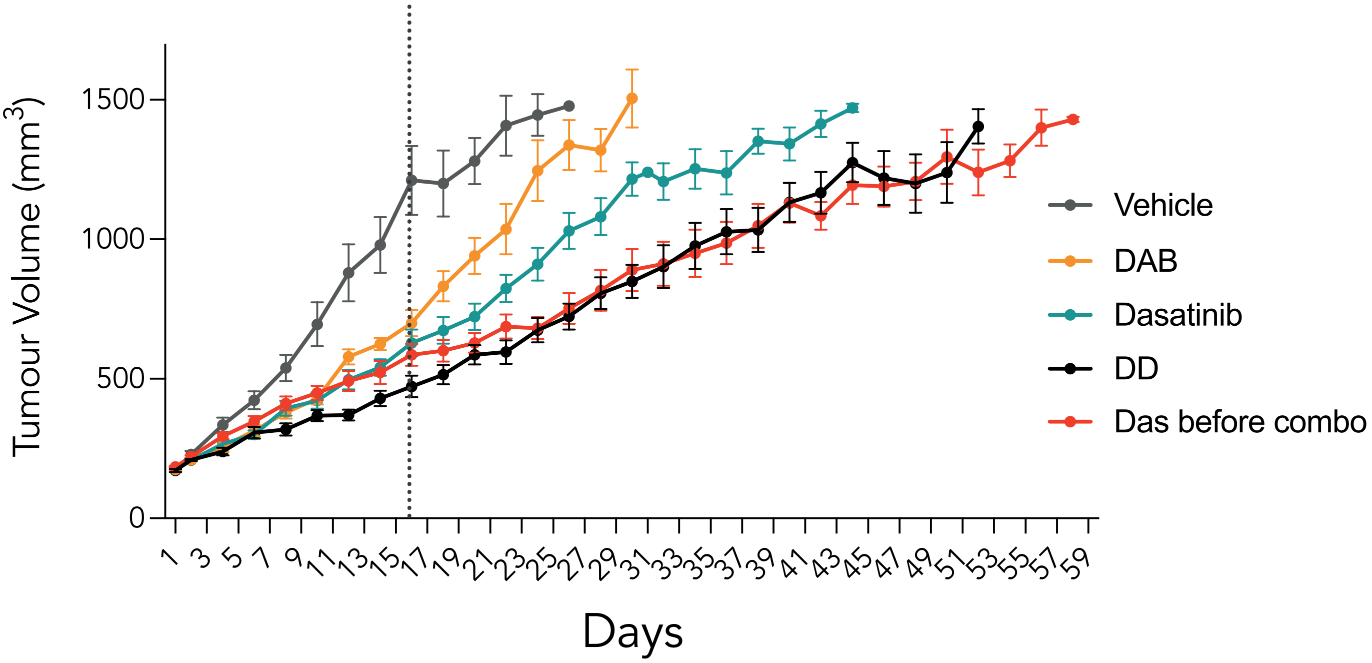

Appendix Figure S9. Inhibition of SRC sensitizes melanoma to BRAFi treatment in a PDX model (in complement to Figure 6)

PDX model MEL006R (BRAFi resistant) was implanted in NMRI nude mice. Mice with tumors reaching 200mm³ were treated daily with vehicle (n=6, gray), dabrafenib alone (Dab, Biorbyt, 30 mg/kg, n=5, Orange) dasatinib alone (Das, Selleckchem, 30 mg/kg, n=9, green) or in combination dabrafenib + dasatinib (Dab + Das, combo, 30 mg/kg, n=12, black) or dasatinib 16 days before combo (Dab + Das, combo, 30 mg/kg, n=12, red) .

Appendix Table S1 **List of genes for different signatures**

	Description	From									
**alpha**	AhR target genes	Corre et al, 2018	CYP1B1	INHBA	LRRC49	OSMR	PMAIP1	REEP2	RUNX2	THBS1	
**beta**	Pigmentation genes	Corre et al, 2018	GPR143	Mitf	MLANA	MLPH	Rab38	SLC45A2	SNCA	TYR	
**res**	BRAFi resistance	Corre et al, 2018	AXL	GCNT1	ITGA1	LPAR1	NRP1	ZEB1			
**Invasive**	invasive phenotype	Supplemented Table 2 Verfaillie et al., 2015	ASPH	CD151	COL1A1	COL1A2	COL6A1	COL6A2	CYR61	FAM129B	GNAI2
			HDGF	HMGA1	HSPA5	ITGB1	LAMB2	LAMC1	LOXL2	MAP4K4	MYH9
			NOTCH2	QSOX1	RTN4	SLC38A2	STC2	THBS1	TMSB10	TPM4	VEGFA
**Proliferative**	proliferative phenotype	Supplemented Table 2 Verfaillie et al., 2015	ACSL3	AP1S2	APOE	ASAH1	ATP1A1	BACE2	CDK2	CLCN7	CTNNB1
			DCT	FASN	FMN1	GPNMB	GPR56	GSTO1	HMCN1	HMG20B	HPS4
			ITPKB	IVNS1ABP	KCNAB2	LGALS3	LYST	MARCKSL1	MBP	MED15	MFI2
			MFSD12	MICAL1	MITF	MLANA	MT-CO1	MYH10	MYO10	MYO1D	MYO5A
			PACSIN2	PEBP1	PLP1	PLXNC1	PMEL	PRKD3	PSAP	QPCT	RPLP1
			RPS12	S100B	SCARB1	SCD	SDC3	SDCBP	SGK1	SIRPA	SLC16A6
			SLC1A4	SLC6A8	SORT1	SOX10	SREBF2	ST3GAL4	STXBP1	TBC1D16	TFAP2A
			TNFRSF14	TRIB2	TSPAN3	UBL3	TYRP1	VAT1	VGF	ZFYVE16	
**Melanocytic**	differenciation phenotype	Supplemented information Tsoi et al., 2018	ABCD1	ADAM23	ADCY2	APOLD1	CAPG	CCL18	CDH1	CDH3	CEACAM1
			DENND2D	EPHA5	FAM174B	FYB	GFOD1	GPR143	H2AFJ	HMOX1	HSD17B14
			IL12RB2	IL16	KCNAB2	LXN	MAPK4	MAT1A	MLANA	MYO1D	NAV2
			NR4A3	OCA2	PRKCB	PYCARD	QPCT	RGS12	RUNX3	SEMA6A	SLAMF7
			SLC16A6	SLC7A8	TDRD3	TNFRSF14	TPPP	TRPM1	TTC39A		
**Transitory**	differenciation phenotype	Supplemented information Tsoi et al., 2018	ALDH1A1	BCHE	COL11A2	COL9A1	CX3CL1	FXYD3	GDF11	LRRTM4	LSAMP
			MCAM	MMP15	NELL1	NPR1	PCDH7	PCSK6	PLBD1	RAPGEF4	RNASE1
			SCRG1	SELENBP1	SEMA3E	SOD3	SORL1	TSPAN7	XYLT1		
**Neural crest-like**	dedifferenciation phenotype	Supplemented information Tsoi et al., 2018	ABCA6	AIM2	ANGPTL4	BST1	CACNA2D3	CADM1	CADM3	CCL2	CD96
			CHST1	CHST2	CSRP2	CTSS	CYB5R2	DRD2	EHF	EML1	FEZ1
			FOXC2	GDNF	GLI2	HHEX	IER3	ITGB8	LRRC15	MUC5B	NES
			NRXN3	NTM	OPRD1	PCSK5	PHLDA2	PLA2G7	PMEPA1	RAMP1	S100A6
			SEMA3B	SFRP1	SH3PXD2A	SLC24A3	SOX2	SQRDL	ST8SIA5	STK32B	SULF1
			TFAP2C	TGFA	TGFBI	TMEM47	TSPAN13	WNT5A			
**Undifferentiated**	dedifferenciation phenotype	Supplemented information Tsoi et al., 2018	ABCG2	ACSL5	AMIGO2	ANKRD1	AR	ARHGAP29	BASP1	BDNF	BNC1
			CALB2	CCDC69	CDH4	CITED2	CLU	CNN1	COLEC10	CPA4	CREB3L1
			CRIM1	DIO2	DMBT1	DOCK2	DSE	EGFR	F2RL1	F3	FERMT1
			FLNC	FMNL1	FOXA1	GALNT6	GATA2	GPRC5A	GPRC5C	IL4R	IL7R
			IRS1	JUP	KISS1	KRT7	KRT81	KRT86	LAMA5	MICAL2	NMT2
			NUAK1	PADI3	PAWR	PBX1	PDGFC	PITX1	PKP2	PLAC8	PLAGL1
			PLAU	PLEKHA6	PSG4	PSG5	PSG9	REN	RUNX2	SCN5A	SCNN1A
			SECTM1	SERPINB7	SFN	SLC38A4	SLC8A1	SLPI	SOX9	TBC1D2	TBL1X
			TMEM40	TNFAIP2	TNFSF18	TOX	UCP2	ZNF185	ZNF467		
**Epithelial**	epithelial phenotype	Supplemented Table 2 Mak et al. 2016	AP1G1	ATP8B1	CDH1	CDS1	CGN	CLDN4	CNOT1	CTNND1	ERBB3
				ESRP1	ERBB2	F11R	GALNT3	GPR56	GRHL2	HOOK1	IRF6
				MAP7	MARVELD3	OCLN	PRSS8	SPINT1			
**Mesenchymal**	mesenchymal phenotype	Supplemented Table 2 Mak et al. 2016	ADAM12	ADAMTS12	ADAMTS2	AEBP1	ANGPTL2	ANTXR1	AXL	BNC2	CALD1
			CDH2	CMTM3	CNRIP1	COL10A1	COL1A1	COL1A2	COL3A1	COL5A1	COL5A2
			COL6A1	COL6A2	COL6A3	COL8A1	DACT1	EMP3	FAP	FBN1	FN1
			FSTL1	GPC6	GYPC	HTRA1	INHBA	ITGA11	LOXL2	LRRC15	NAP1L3
					NID2	OLFML2B	PCOLCE	PDGFRB	PMP22	POSTN	SPARC
					SPOCK1	SULF1	SYT11	THBS2	VCAN	VIM	ZEB2
**CSC**	cancer stem cells		ABCG2	ALDH1A1	CD44	CXCR4	KLF4	NES	NGFR	PROM1	SOX2
**FOCAL_ADHESION**	https://www.gsea-msigdb.or	Supplemented Table 4	ACTN4	BAD	BIRC3	CAPN2	CAV1	CAV2	COL5A1	COL5A2	COL6A1
	after GSEA between BRAFi resistant and sensitive melanoma cell lines (CCL		COL6A2	EGFR	ERBB2	FLNA	FLNB	ITGA2	ITGA3	ITGA5	JUN
			LAMA5	LAMB3	MYLK	PDGFA	PDGFB	PDGFC	PDGFRB	PPP1R12A	PRKCA
			RAC2	ROCK2	SHC1	THBS1	TLN1	TLN2	VASP	VCL	VEGFA
			VEGFC	ZYX							
**INTEGRIN**	https://www.gsea-msigdb.or	Supplemented Table 4	JUN	CAPN1	CAPNS1	CAV1	HRAS	ITGB1	MAPK8	PPP1R12B	RAP1A
	after GSEA between BRAFi resistant and sensitive melanoma cell lines (CCL		ROCK1	SHC1	TLN1	VCL	ZYX				
**CSK**	https://www.gsea-msigdb.or	Supplemented Table 4	ADCY1	CSK	PRKAR1A	PRKAR1B	PRKAR2A	PRKAR2B			
	after GSEA between BRAFi resistant and sensitive melanoma cell lines (CCLE) and between dedifferentiated and differentiated melanoma cell lines (TSOI)										
**SRC**	https://www.gsea-msigdb.or	Supplemented Table 4	ARRDC3	ARSJ	CHAC2	COL8A1	COQ4	CSAD	DPYD	DTWD1	E2F8
	after GSEA between BRAFi resistant and sensitive melanoma cell lines (CCL		EREG	GFRA3	HS3ST3A1	IFIT1	IFITM10	IL7R	IRAK2	LFNG	MEGF6
			MMP1	MMP3	MN1	MYSM1	NRP1	PAK3	PHLDA2	PIK3C2G	PKP2
			PLK4	PTBP2	PTGER4	RACGAP1	RAP1A	RFTN1	S1PR3	SCRN2	SEMA6B
			SERPINB2	SYT1	UHRF1	ZNF3	ZNF436	ZNF451			
**FAK**	https://www.gsea-msigdb.or	Supplemented Table 4	ARHGAP26	ARHGAP35	ARHGEF28	CAPN2	CRK	ELMO1	ETS1	ITGA5	ITGB1
	after GSEA between BRAFi resistant and sensitive melanoma cell lines (CCL		ITGB5	JUN	MAP2K4	MAPK8	PAK1	PXN	RAP1A	RASA1	ROCK2
			RRAS	SH3GL1	TLN1	VCL					
**BCAT_DN**	https://www.gsea-msigdb.or	Supplemented Table 4	STARD13	IL1A	PLAGL1	IRAK2	SMURF2	ARHGAP29	SHC1	ZSWIM6	FRYL
	after GSEA between BRAFi resistant and sensitive melanoma cell lines (CCL		ATP10D	PRPF38B	RASAL2	TAF1D	OSBPL10	ASH1L	RIF1	RAP1A	PTHLH
**BCAT_UP**	https://www.gsea-msigdb.or	Supplemented Table 4	ITGA9	DYNC1I1	LEF1	BAMBI	ASRGL1	QPCT	SRPX	MYB	HHEX
	after GSEA between BRAFi resistant and sensitive melanoma cell lines (CCL		FAH	FZD7	NEDD9						
**TGFB**	https://www.gsea-msigdb.or	Supplemented Table 4	NDST1	LIF	CARF	ARHGAP12	SEC14L2	JUNB	TBX3	HTR1D	EPHA4
	after GSEA between BRAFi resistant and sensitive melanoma cell lines (CCL		PPP2R2B	ATF3	DPY19L1	HCAR3	LARP6	ETS2	SKIL	FERMT2	ADAM22
			CYP26B1	COL16A1	IL1RL1	NFATC4	RUSC2	WNT11	CYP24A1	PPP1R13L	PAIP2B
			SSBP3	DLC1	HS3ST2	SMTN	TNFAIP8	JMJD1C	CLCF1	ITGA5	KLF7
			PMEPA1	JUN	CHST3	PDGFA	NGF	KLF4	GADD45B	CDKN2B	HLX
			FOXF2	RNASE4	FOXD1	SMURF2	S100A3	IL11	RGCC	NTF3	FGF1
			IRS1	KCNN4	ELL2	TAGLN	LTBP2	JAM2	EDN1	KHDC1	STC1
			HRH1	FAT4	PKIA	F2RL1					
**KRAS**	https://www.gsea-msigdb.or	Supplemented Table 4	LOXL2	VCAN	NRP1	VEGFC	AKR1C3	SERPINE1	DPYD	SCG5	DKK1
	after GSEA between BRAFi resistant and sensitive melanoma cell lines (CCL		TMEM158	SLC22A4	BMP2	RAC2	TFPI2	PID1	TSPAN5	ANPEP	IFITM1
			RGS17	LTBP3	BCL6	CXCL3	INHBA	COL6A3	IFIT1	SEC24D	PXDN
			MMP1	KHDRBS3	SPAG4	SMTN	GREM1	BCL3	IFI44L	IFI44	RGS2
			ASPH	DPYSL2	CDA	IRF9	FADS2	CSF3	GATA6	PLAU	S100P
			FUT8	EHBP1L1	PLIN3						
**YAP**	https://www.gsea-msigdb.or	Supplemented Table 4	CRIM1	AXL	THBS1	FSCN1	SERPINE1	SLIT2	TGM2	FGF2	DLC1
	after GSEA between BRAFi resistant and sensitive melanoma cell lines (CCL		NDRG1	GADD45B	BICC1	AMOTL2	GAS6	FSTL1	DUSP1	DDAH1	FLNA
			EMP2								
**EGFR**	https://www.gsea-msigdb.or	Supplemented Table 4	LOXL2	ITGA2	CCL2	MAP3K5	GBP1	TFPI	MGLL	WNT5A	ELL2
	after GSEA between BRAFi resistant and sensitive melanoma cell lines (CCL		RGS16	IL1R1	LIF	IDO1	AOX1	TNFRSF11B	CYP1B1	PLAGL1	INHBA
			IRF1	EGFR	EDN1	TMEM45A	GBP2	KRT7	EXT1	CMAHP	ITGA3
			MMP1	IFIT3	SGMS1	CTNNAL1	FERMT2	BCL3	TNFAIP8	PSMB9	ASPH
			ABCC3	IL15RA	GCNT1	F2RL1	SNAPC1	PGM3	KLF4	TAP1	GJA1
			PMAIP1	S100P							
**PTEN**	https://www.gsea-msigdb.or	Supplemented Table 4	COL13A1	NAV3	LOXL2	AKR1C3	IL7R	IL1B	JUN	BIRC3	ARL4C
	after GSEA between BRAFi resistant and sensitive melanoma cell lines (CCL		PTGS2	KRT15	CPA4	DENND2A	RTN1	SECTM1	DNAJB5	APOL3	PCSK7
			G0S2	NID2	TNFRSF10D	AKR1B10	AGPAT4	CUEDC1	IL32	SPOCK1	PLAU
**EIF4GI**	https://www.gsea-msigdb.or	Supplemented Table 4	DDX54	UBE2E1	LRPAP1	PERP	UQCRC2	QARS	ADSL	ATP5A1	IGF2R
	after GSEA between BRAFi resistant and sensitive melanoma cell lines (CCL		NQO1	GSS	RMND1	ZBED1	GLUD1	GIN1	MRPS30	PSAP	ECI1
			SPG20	ATP1A1	PDHA1	TNNT1	TRAF3IP2	SETDB1	HADHB	FXYD3	CA2
			FRAT2	SAMM50	ASS1	AFG3L2	ASAH1	CTSH			

(Appendix Table S2) target genes						
**Gene ID**	**Name**	**#OMIM**	**Description**	**Function**	**Role in melanoma and in resistance, invasion, differentiation**	**Regulation by AhR**
ABCG2	ATP-BINDING CASSETTE, SUBFAMILY G, MEMBER 2	603756	membrane transporter belonging to the ATP-binding cassette (ABC) superfamily of membrane transporters	xenobiotic transporter, multidrug resistance transporter **(Hasanabady et al., 2016)**	highly expressed in Vem resistant cells **(Zubrilov et al., 2015; Michaelis et al., 2014)** and associated with bad prognosis **(Speigl et al., 2017)**	induction of expression **(Yan et al., 2018; Wu et al., 2018)**
ALDH1A1	ALDEHYDE DEHYDROGENASE 1 FAMILY, MEMBER A1	100640	liver cytosolic isoform of acetaldehyde dehydrogenase	detoxification of endogenous and exogenous aldheyde substrates, stem cells and cancer stem cells marker **(Tomita et al., 2016)**	Inhibition significantly reduces melanoma tumor growth and invasion **(Contador-Troca et al., 2015)**	induction of expression **(Contador-Troca et al., 2015)**
BASP1	BRAIN-ABUNDANT SIGNAL PROTEIN, MEMBRANE-ATTACHED, 1	605940		Intercellular signaling and nerve outgrowth	susceptibility locus associated with melanoma **(Chahal et al., 2016; Ransohoff et al., 2017)**	
BDNF	BRAIN-DERIVED NEUROTROPHIC FACTOR	113505	prosurvival factor	survival of striatal neurons in the brain	involved in melanoma dedifferentiation reprogramming **(Zhang et al., 2016)**	potential regulation **(Lin et al., 2009)**
CCL2	CHEMOKINE, CC MOTIF, LIGAND 2	158105	member of the small inducible gene (SIG) family	recruitment of monocytes to sites of injury and infection, tumor progression, angiogenesis **(Yoshimura et al., 2018)**	induced specific mIRNA expression in resistant melanoma **(Vergani et al., 2015)**, BRAFi decreased CCL2 expression **(Knight et al., 2013)**, CCLE favorize melanoma invasion **(Li et al., 2013)**, therapeutic target in melanoma **(Gazzaniga et al., 2007)**	induction of expression **(Goode et al., 2014; Watanabe et al, 2013)**
CLU	CLUSTERIN	185430	member of the human complement system	control mechanism of the complement cascade; role in cancer chemoresistance **(Koltai et al., 2014)**	regulates drug resistance in melanoma **(Hoeller et al., 2005)** Loss of expression induces apoptosis in melanoma **(Mustafiet al., 2017)**	
COL1A1	COLLAGEN, TYPE I, ALPHA-1	120150	protein containing 2 alpha-1 polypeptide chains and 1 alpha-2 chain	Skin Collagen component	expressed in melanoma metastasis **(Ilhan-Mutlu et al., 2016)**	FICZ inhibits induction of expression by TGFB **Murai et al., 2018; Monteleone et al., 2016)**
COL6A1	COLLAGEN, TYPE VI, ALPHA-1	120220	Members of the collagen VI family	form distinct networks of microfibrils in connective tissue and interact with other extracellular matrix components	high expression is a poor prognosis in cancer **(Turtoi et al., 2014; Wan et al., 2014; Hou et al., 2016)**	
COL6A2	COLLAGEN, TYPE VI, ALPHA-2	120240	alpha-2 subunit of type VI collagen	extracellular matrix protein	high expression is a poor prognosis in cancer **(Cheon et al., 2015)**	
CPA4	CARBOXYPEPTIDASE A4	607635	metallocarboxypeptidase	cell proliferation and differentiation	prognostic markers in several cancers **(Sun et al., 2016; Sun et al., 2017; Tanco et al., 2010)**	
CRIM1	CYSTEINE-RICH MOTOR NEURON PROTEIN 1	606189	Membrane protein	interaction with growth factors implicated in motor neuron differentiation and survival, antagonist of BMP	invasion and migration **(Ogasawara et al., 2018)**, high expression in cancer **(Tang et al., 2014)**	
CYR61	CYSTEINE-RICH, ANGIOGENIC INDUCER, 61	602369	secreted, cysteine-rich, heparin-binding protein acting as an extracellular matrix-associated signaling protein	promotes the adhesion of endothelial cells through interaction with integrin and augments growth factor-induced DNA synthesis in the same cell type **(Lau et al., 2011)**	tumor suppressor in melanoma **(Dobroff et al., 2009)**, therapeutic target **(Chen et al., 2016)**	potential regulation **(Collins et al., 2009)**
F2RL1	COAGULATION FACTOR II RECEPTOR-LIKE 1	600933	7-transmembrane-region receptor	Inflammation	contributes to tumor cell motility and metastasis **(Shi et al., 2004)**, loss of PAR2 contributes to the limitation of local metastatic spread of melanoma **(Olejar et al., 2014)**	
IL7R	INTERLEUKIN 7 RECEPTOR	146661	receptor for interleukin-7	regulation of lymphopoiesis		
MICAL2	MICROTUBULE-ASSOCIATED MONOOXYGENASE, CALPONIN AND LIM DOMAINS-CONTAINING, 2	608881	cytoskeleton dynamics regulator	cell growth, axon guidance, vesicle trafficking, apoptosis	involved in cell migration, invasion and EMT **(Mariotti et al.; Wang et al., 2018; Cai et al., 2018)**	
NTM	NEUROTRIMIN	607938	IgLON family of GPI-anchored cell adhesion molecules			
NUAK1	NUAK FAMILY, SNF1-LIKE KINASE, 1	608130		tumor cell survival factor	high expression is a poor prognosis in cancer **(Port et al., 2018; Phippen et al., 2016; Chen et al., 2017)**, involved in chemoresistance by inducing EMT **(Xu et al., 2016)**, potential target in melanoma **(Bell *et al.* , 2014)**	
PAWR	PRKC, APOPTOSIS, WT1, REGULATOR	601936		inhibition of transcription induced by WT1		
PDGFC	PLATELET-DERIVED GROWTH FACTOR C	608452	monomeric protein secreted as a latent dimeric factor, PDGFCC,	major mitogens and stimulants of motility in mesenchymal cells	paracrine stimulation of NRP1 by PDGFC contributes to the acquisition of a metastatic phenotype by melanoma cell **(Ruffini et al., 2017)**	
SOX9	SRY-BOX 9	608160	transcription factor essential for both sex and skeletal development	formation of testes from bipotential gonads	high expression is a poor prognosis in melanoma and is associated with invasion phenotype **(Cheng et al., 2015)**	
STC2	STANNIOCALCIN 2	603665		regulation of Ca2+, highly expressed in cancers **(Yeung et al., 2012)**	induces cell proliferation in cisplatin resistant cervical cancer **(Wang et al., 2015)**	induction of expression **(Joshi et al., 2015; Harper et al., 2013)**
TBL1X	TRANSDUCIN-BETA-LIKE 1, X-LINKED	300196	component of the nuclear corepressor (NCOR) complex	specific adaptor that recruits the ubiquitin-conjugating/19S proteasome complex	important for wnt signalling activation **(Li et al., 2008)**	
TGFBI	TRANSFORMING GROWTH FACTOR, BETA-INDUCED	601692	extracellular matrix protein	binding to fibronectin, collagen, and integrins	high expression is a poor prognosis in melanoma and is associated with invasion phenotype **(Lauden et al., 2014; Nummela et al., 2012)**	

Appendix Table S3 (related to Appendix Figure S8A, correlation of dasatinib efficiency with gene expression level (Z-score))													
	AhR	Undifferentiated	Transitory	Resistance	Proliferation	NCL	Mesenchymal	Melanocytic	Invasion	Epithelial	CSC	beta	alpha
CRIM1	-0,3	0,397											
PLAU	0,327	0,342											
F3	0,424	0,329											
TBC1D2	0,447	0,316											
CPA4	-0,471	-0,303											
AMIGO2	0,399	0,3											
ARHGAP29	0,273	0,294											
TNFAIP2	0,349	0,276											
EGFR	0,452	0,27											
PDGFC	0,276	0,268											
ANKRD1	-0,466	0,266											
GPRC5A	0,456	0,261											
LAMA5	0,36	0,255											
DSE	0,215	0,255											
BDNF	-0,236	0,242											
NMT2	0,115	0,241											
TMEM40	-0,363	-0,199											
ZNF185	0,223	0,189											
NUAK1	-0,237	0,189											
IL4R	0,207	0,183											
PAWR	0,27	0,182											
FOXA1	-0,335	-0,178											
MICAL2	-0,319	0,159											
PADI3	0,163	0,153											
SCN5A	-0,238	-0,15											
PLEKHA6	0,136	-0,147											
F2RL1	0,397	0,146											
IRS1	0,281	0,138											
SERPINB7	0,309	0,134											
CREB3L1	0,14	0,134											

**ZNF467**	-0,122	-0,133											
**TOX**	-0,242	-0,129											
**LSAMP**	-0,196		-0,197										
**PCDH7**	0,291		0,19										
**RAPGEF4**	-0,157		-0,19										
**AXL**	0,28			0,389									
**NRP1**	0,294			0,224									
**LPAR1**	0,31			0,186									
**MITF**	0,291				0,25								
**MED15**	0,199				0,229								
**GPR56**	0,274				-0,218								
**SGK1**	0,354				-0,215								
**LGALS3**	0,56				0,216								
**GSTO1**	-0,217				-0,188								
**STXBP1**	-0,204				-0,188								
**MYO1D**	-0,185				-0,183								
**BACE2**	0,368				0,183								
**SDCBP**	0,292				0,179								
**GPNMB**	-0,378				-0,164								
**TGFBI**	0,529					0,373							
**DRD2**	-0,227					-0,264							
**SEMA3B**	0,321					0,264							
**ITGB8**	0,298					0,262							
**CCL2**	0,186					0,235							
**TGFA**	0,318					0,234							
**IER3**	0,549					0,225							
**LRRC15**	-0,325					-0,223							
**PMEPA1**	0,37					0,217							
**OPRD1**	-0,161					0,207							
**SULF1**	-0,183					0,191							
**S100A6**	0,523					0,168							
**AXL**	0,28						0,389						

**LOXL2**	-0,239						0,388						
**FSTL1**	0,193						0,318						
**COL8A1**	0,21						0,315						
**COL1A1**	0,101						0,284						
**COL6A1**	-0,225						0,281						
**PMP22**	-0,264						0,274						
**FN1**	0,269						0,264						
**HTRA1**	0,354						0,264						
**CALD1**	0,22						0,259						
**COL5A1**	0,161						0,249						
**INHBA**	0,237						0,248						
**SPARC**	0,107						0,244						
**OLFML2B**	-0,262						0,229						
**LRRC15**	-0,325						-0,223						
**COL6A2**	0,148						0,212						
**VIM**	-0,115						-0,197						
**SULF1**	-0,183						0,191						
**FBN1**	0,114						0,181						
**ADAM12**	-0,143						0,173						
**CMTM3**	0,178						0,167						
**ITGA11**	-0,114						0,163						
**TTC39A**	-0,343							-0,253					
**MAPK4**	-0,164							-0,238					
**LXN**	0,187							-0,229					
**CAPG**	0,317							0,198					
**TPPP**	-0,176							-0,195					
**ADCY2**	-0,188							-0,192					
**MYO1D**	-0,185							-0,183					
**HSD17B14**	0,326							0,167					
**THBS1**	0,428								0,405				
**MYH9**	0,313								0,392				
**LOXL2**	-0,239								0,388				

**CYR61**	0,394								0,387				
**COL1A1**	0,101								0,284				
**ITGB1**	0,363								0,281				
**COL6A1**	-0,225								0,281				
**LAMB2**	0,368								0,27				
**LAMC1**	0,334								0,261				
**NOTCH2**	0,226								0,227				
**ASPH**	0,541								0,226				
**COL6A2**	0,148								0,212				
**TPM4**	0,193								0,207				
**FAM129B**	0,507								0,184				
**CD151**	0,399								0,183				
**QSOX1**	0,273								0,172				
**SLC38A2**	0,205								0,157				
**HSPA5**	0,308								0,148				
**TMSB10**	0,232								0,144				
**VEGFA**	0,289								0,127				
**HOOK1**	-0,28									-0,245			
**CTNND1**	0,484									0,242			
**GPR56**	0,274									-0,218			
**CD44**	0,445										0,241		
**MLPH**	0,365											0,168	
**THBS1**	0,428												0,405
**OSMR**	0,429												0,303
**INHBA**	0,237												0,248
**REEP2**	-0,372												-0,175

Appendix Table S4 List of RT-qPCR primers				
**Genes**				
**AhR**	Forward	accagcctcaggatgtgaac	Reverse	tcattatgctgtacaagtcactgttt
**CCL2**	Forward	tgagtttttcatcactccattca	Reverse	gatatgaatcagcatcgaggagt
**CLU**	Forward	ctacttctggatgaatggtggtgacc	Reverse	cgggtgaagaacctgtcct
**COL6A1**	Forward	gaagagaaggccccgttg	Reverse	cggtagcctttaggtccgata
**CYR61**	Forward	aaacccggatttgtgaggt	Reverse	gctgcatttcttgccctttt
**IL7R**	Forward	aaagttttaatgcacgatgtagctt	Reverse	tgtgctggataaattcacatgc
**MICAL2**	Forward	cttcttcttcaaccagtgtttctg	Reverse	ggggctcagaagctgtca
**MMP1**	Forward	gctaacctttgatgctataactacga	Reverse	tttgtgcgcatgtagaatctg
**MMP2**	Forward	tacaggatcattggctacacacc	Reverse	ggtcacatcgctccagact
**PAWR**	Forward	gcagatcgagaagaggaagc	Reverse	tcatcttcgtactcatctaagcactc
**PDGFC**	Forward	cagcaacaaggaacagaacg	Reverse	tgggctgtgaatacttccatt
**TBL1X**	Forward	gtttcccaccaaaatccatc	Reverse	ggcttcgcgtgattattga
**TGFBI**	Forward	cgagtgctgtcctggatatg	Reverse	cccagggtctcgtaaaggtt
**THBS1**	Forward	caatgccacagttcctgatg	Reverse	tggagaccagccatcgtc

Appendix Table S5 List of Antibodies			
**Protein**	Companies	Ref	dilution
**AhR (A-3)**	Santa Cruz Biotechnology	sc-133088	1/1000
**HSC70 (B-6)**	Santa Cruz Biotechnology	sc-7298	1/1000
**P-SRC (Y416)**	Cell Signaling Technology	2101S	1/1000
**SRC**	Cell Signaling Technology	2108S	1/1000
**P-FAK (Y576/577)**	Cell Signaling Technology	3281S	1/1000
**FAK**	Cell Signaling Technology	13009S	1/1000
**P-EGFR (Y1068)**	Cell Signaling Technology	3777S	1/1000
**P-EGFR (Y845)**	Cell Signaling Technology	2231S	1/1000
**EGFR**	Cell Signaling Technology	4267S	1/1000
