# Supplementary material for: The AhR‐SRC axis as a therapeutic vulnerability in BRAFi‐resistant melanoma
Source: EMBO Mol Med. 2022 Oct 28;14(12):e15677. doi: 10.15252/emmm.202215677 (PMC9728058; doi:10.15252/emmm.202215677)
Supplement: Supplementary file 2 — Expanded View Figures PDF [file EMMM-14-e15677-s012.pdf]

Expanded View Figures

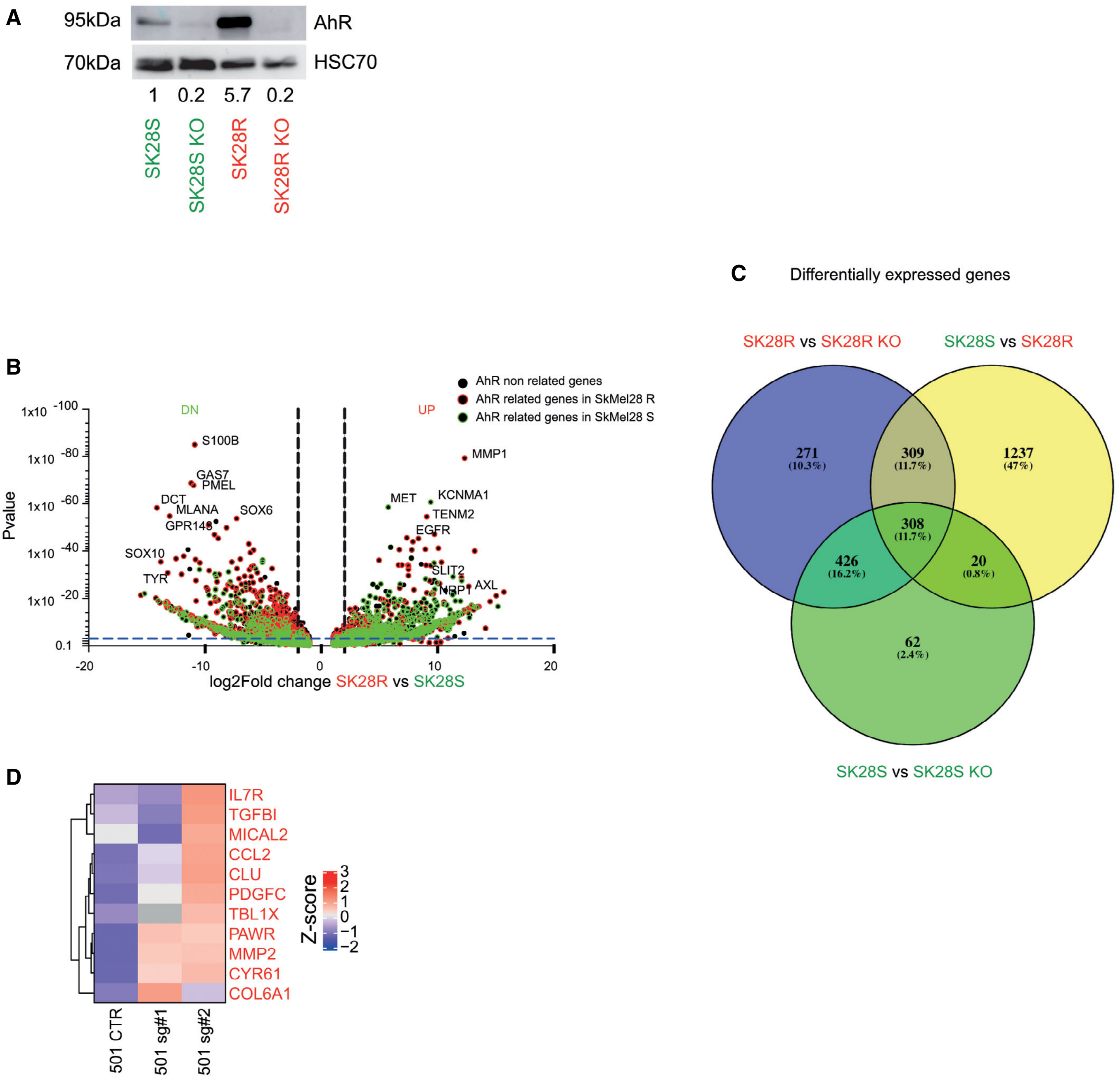

Figure EV1. Role of AhR in the regulation of gene expression in SK28 melanoma cell lines.

A Protein levels of AhR were analyzed by western blotting in BRAFi-sensitive or resistant SK28 cells invalidated or not for the AhR and quantified using Fiji® relative to the level of HSC70 protein.

B Volcano plot combining the magnitude of the fold change (ratio of expression) between SK28S (green), R (red) wild-type, or invalidated for AhR (KO) and the *P*-values.

C Venn diagram representing the overlap between the differentially expressed genes between SK28 R vs. R (Fig 2D), SK28S vs. SKO, and SK28R vs. RKO.

D Expression heatmap of the median expression ($n = 2$; RT-qPCR) for AhR target genes (invasion) in 501Mel CTR cells and those transduced with sgRNA targeting *AhR* (#1, #2). The scale corresponds to the Z scores.

Source data are available online for this figure.

 © 2022 The Authors

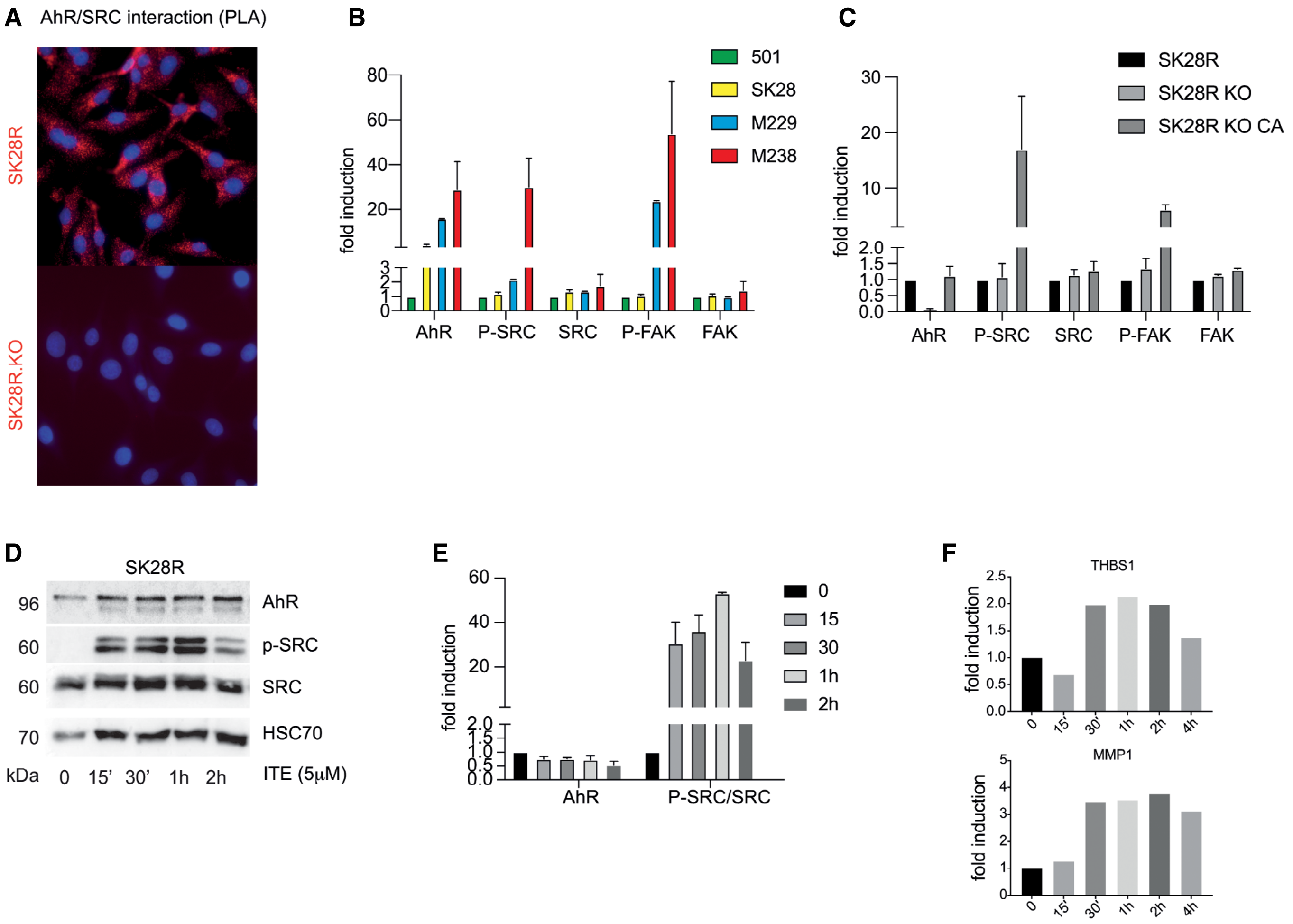

Figure EV2. Activation of SRC after canonical activation of AhR in melanoma cell lines.

A The AhR-SRC interaction was quantified by proximity ligation assay. Hoechst-stained nucleus in blue (20× magnification).

B Quantification of protein levels using ImageJ (Fiji) corresponding to Fig 4B ($n = 3$ biological experiments, mean ± s.d.).

C Quantification of protein levels corresponding to Fig 4C ($n = 3$ biological experiments, mean ± s.d.).

D, E Protein levels of the AhR, p-SRC (Y416), and SRC were analyzed by western blotting in SK28 after various times of treatment with ITE (5 μM) and quantified using ImageJ (Fiji) (E).

F Histogram showing the expression (RT-qPCR) of *THBS1* and *MMP1* in SK28 after treatment by ITE (5 μM) at different times ($n = 1$).

Source data are available online for this figure.

© 2022 The Authors

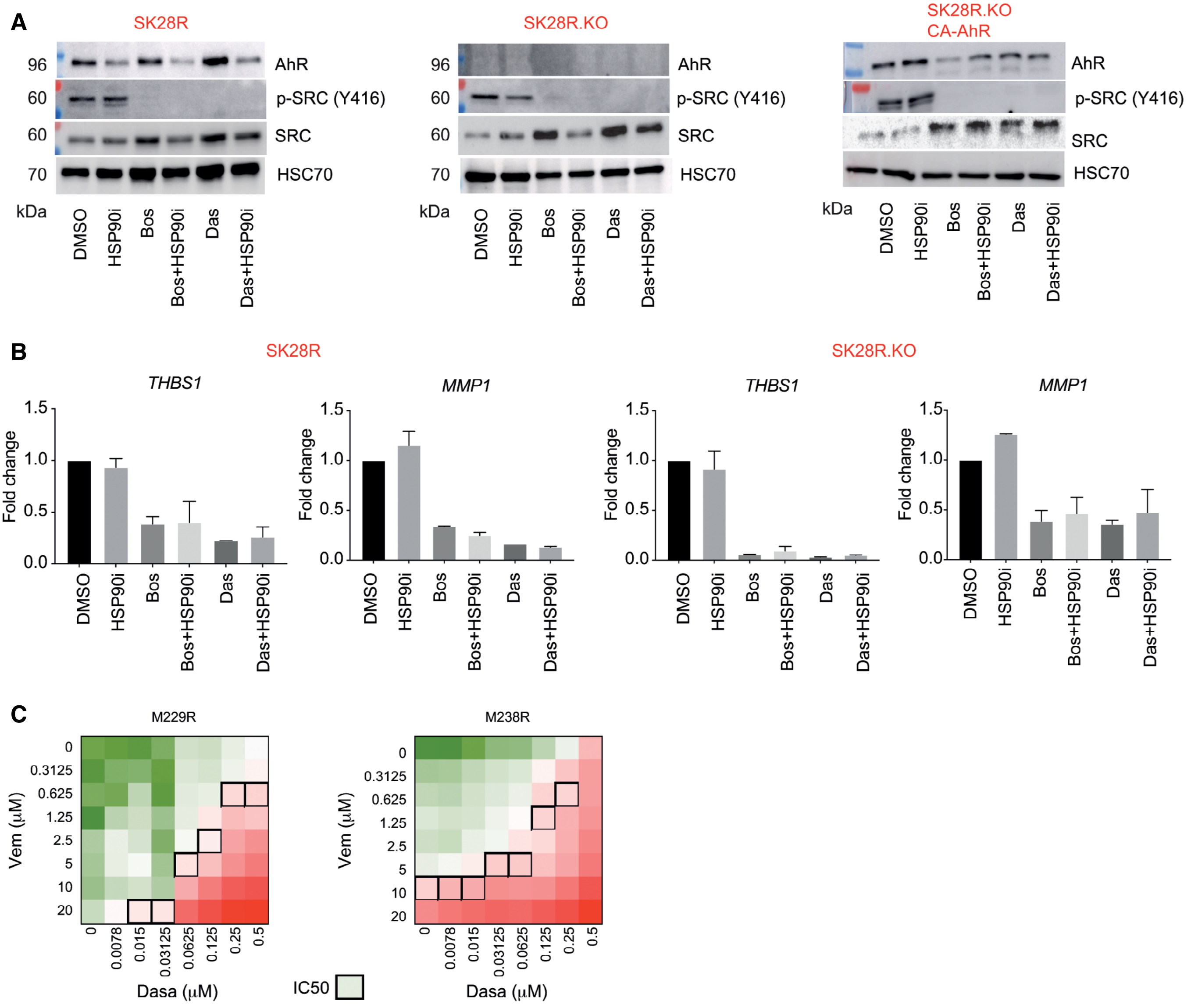

Figure EV3. SRC inhibitors sensitize melanoma cells to BRAFi treatment.

A Protein levels of AhR, p-SRC (Y416), and SRC in SK28R cells invalidated or not for AhR by CRISPR/Cas9 or after rescue with the activated-form of the AhR (CA-AhR) and after treatment for 24 h with an HSP90 inhibitor (HSP990, 10 nM) with or without two different SRC inhibitors: dasatinib (1 µM) or bosutinib (1 µM).

B Histogram representing the expression (RT-qPCR) of *THBS1* and *MMP1* in SK28R and SR28R KO treated for 24 h with an HSP90 inhibitor (HSP990, 10 nM) with or without two different SRC inhibitors: dasatinib (1 µM) or bosutinib (1 µM; $n = 3$ biological experiments, mean ± s.d.).

C Heatmap of the percentage of cell viability of M229R (left) and M238R cells treated with a combination of increasing doses of vemurafenib (y) and bosutinib or dasatinib (1 µM). IC50 values are represented by black squares.

Source data are available online for this figure.

Figure EV4. Impact of inhibition of SRC to sensitize PDX melanoma to BRAFi treatment.

A PDX model MEL006R (BRAFi resistant) was implanted in NMRI nude mice. Mice with tumors reaching 200 mm^3 were treated daily with vehicle ($n = 6$), dabrafenib alone (Dab, Biorbyt, 30 mg/kg, $n = 5$) dasatinib alone (Das, Selleckchem, 30 mg/kg, $n = 9$) or in combination dabrafenib + dasatinib (Das before combo, 30 mg/kg, $n = 12$). PDX tumor volumes were measured every 2 days until reaching 1,500 mm^3.

B IHC for AHR and P-SRC PDX model MEL006R representative for each group of treatment in order to confirm the action of SRCi to specifically prevent phosphorylation of SRC. Quantification of % of positive immunostaining area has been performed using ImageJ (Fiji) after integration of specific signal. Semi-quantitative analysis of optical density for several tumors for each group ($n = 4$) has been performed after calibration of the image using step tablet (Fiji).

 © 2022 The Authors

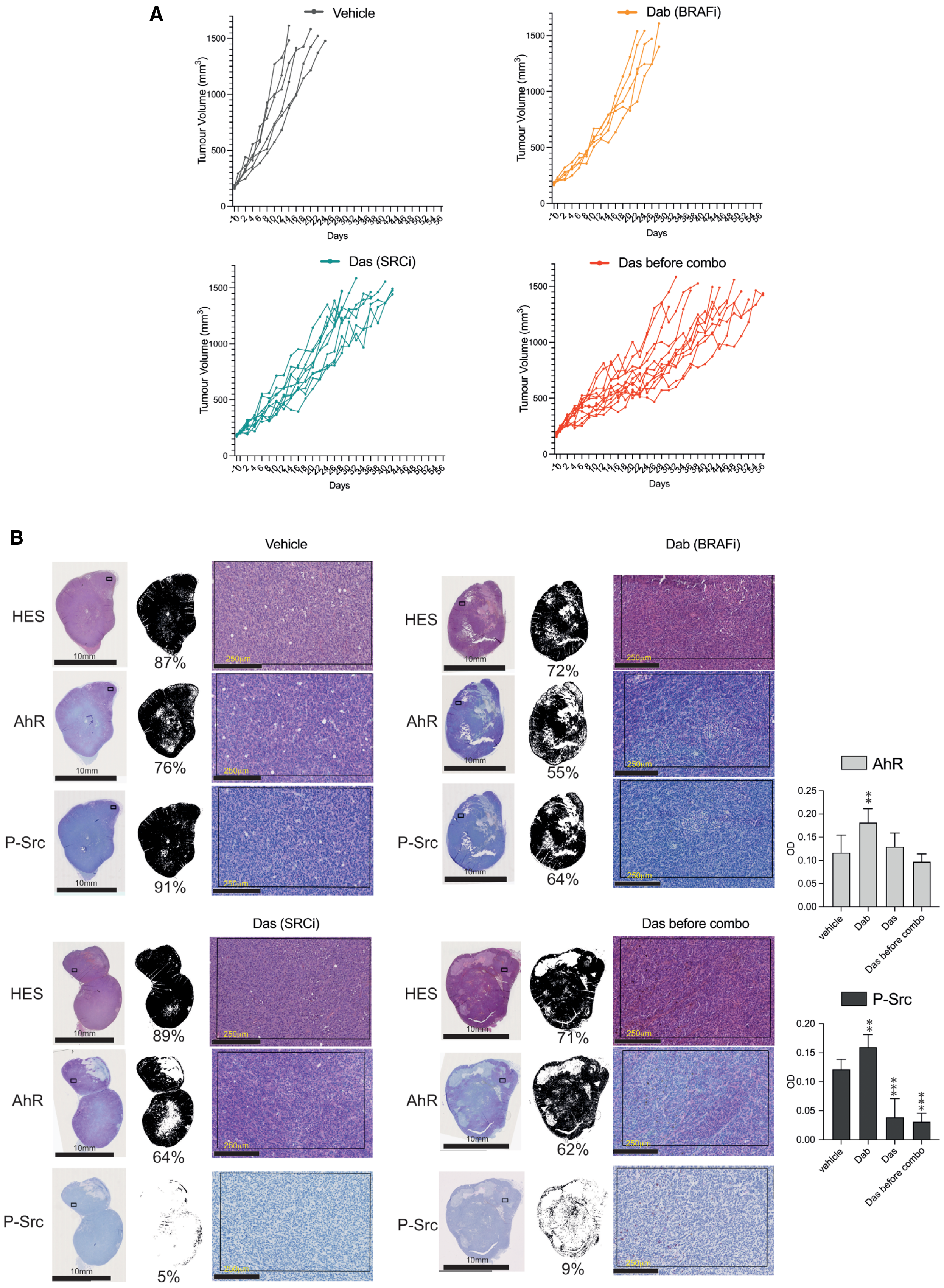
A
Vehicle
Dab (BRAFi)
Das (SRCi)
Das before combo
Tumour Volume (mm³)
Days
B
Vehicle
Dab (BRAFi)
Das (SRCi)
Das before combo
HES
AhR
P-Src
87%
76%
91%
72%
55%
64%
89%
64%
5%
71%
62%
9%
10mm
250µm
AhR
P-Src
OD
vehicle
Dab
Das
Das before combo

Figure EV4.

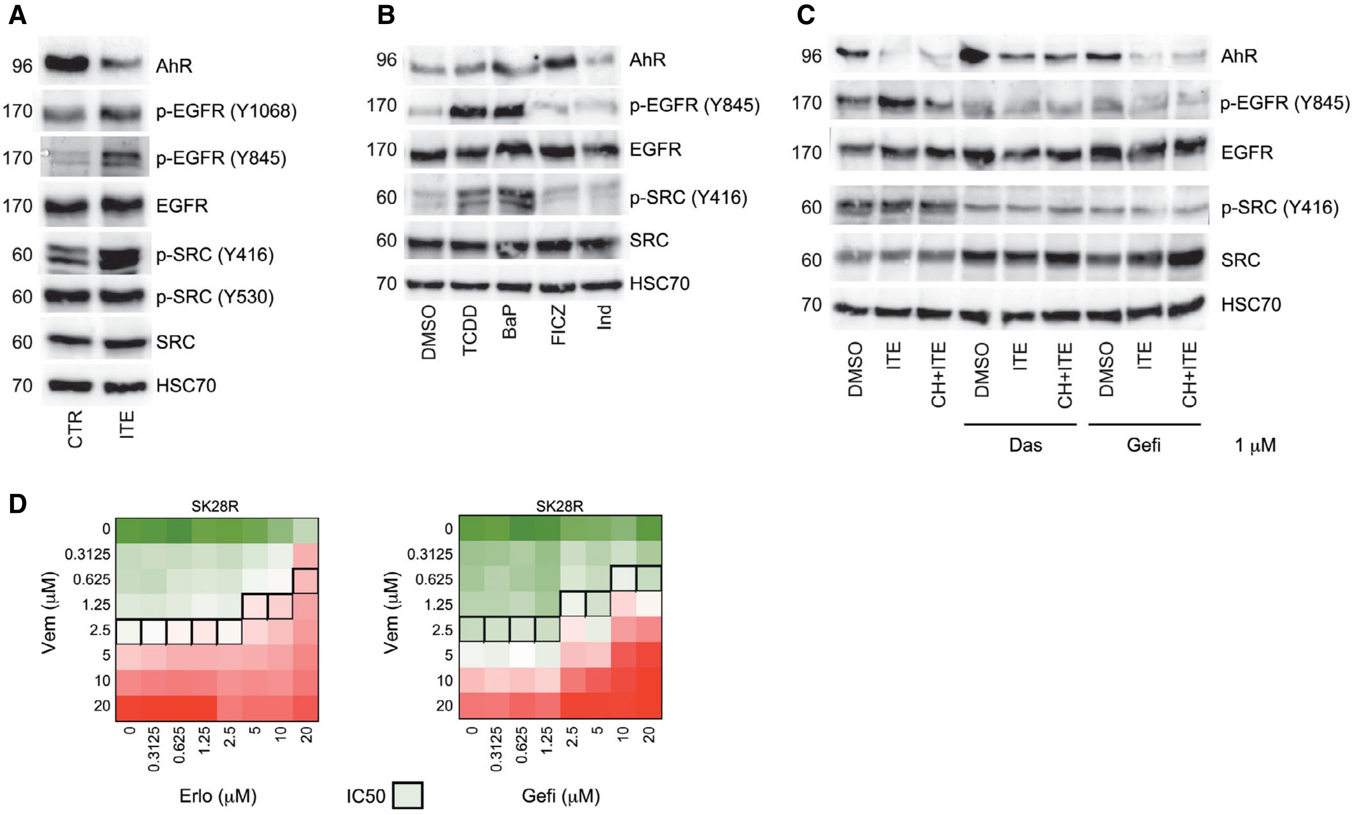

Figure EV5. Canonical activation of AhR leads to activation of the SRC/EGFR axis in melanoma cells.

A Protein levels of AhR, p-SRC (Y416, Y530), SRC, p-EGFR (Y1068, Y845) and EGFR were analyzed by western blotting in SK28R cells treated or not with ITE (5 μM, 24 h).

B Protein levels of AhR, p-SRC (Y416), SRC, p-EGFR (Y845), and EGFR, in the SK28R cell line after treatment with different AhR ligands for 24 h (10 nM TCDD, 5 μM BaP, 5 μM FICZ and 5 μM indirubin).

C Protein levels of AhR, p-SRC (Y416), SRC, p-EGFR (Y845) and EGFR were analyzed by western blotting in SK28R cells treated or not with ITE (5 μM, 24 h) with or not AhR inhibitor (CH-223191, 5 μM), Src inhibitor: dasatinib (0.5 μM) and EGFR inhibitor: gefitinib (20 μM).

D Heatmap of the percentage of cell viability in SKR cells treated with a combination of increasing doses of vemurafenib (y) and erlotinib (left) or gefitinib (right) (1 μM). IC50 values are represented by black squares.

Source data are available online for this figure.

 © 2022 The Authors
