# Supplementary figures and images for: The AhR‐SRC axis as a therapeutic vulnerability in BRAFi‐resistant melanoma
Source: EMBO Mol Med. 2022 Oct 28;14(12):e15677. doi: 10.15252/emmm.202215677 (PMC9728058; doi:10.15252/emmm.202215677)

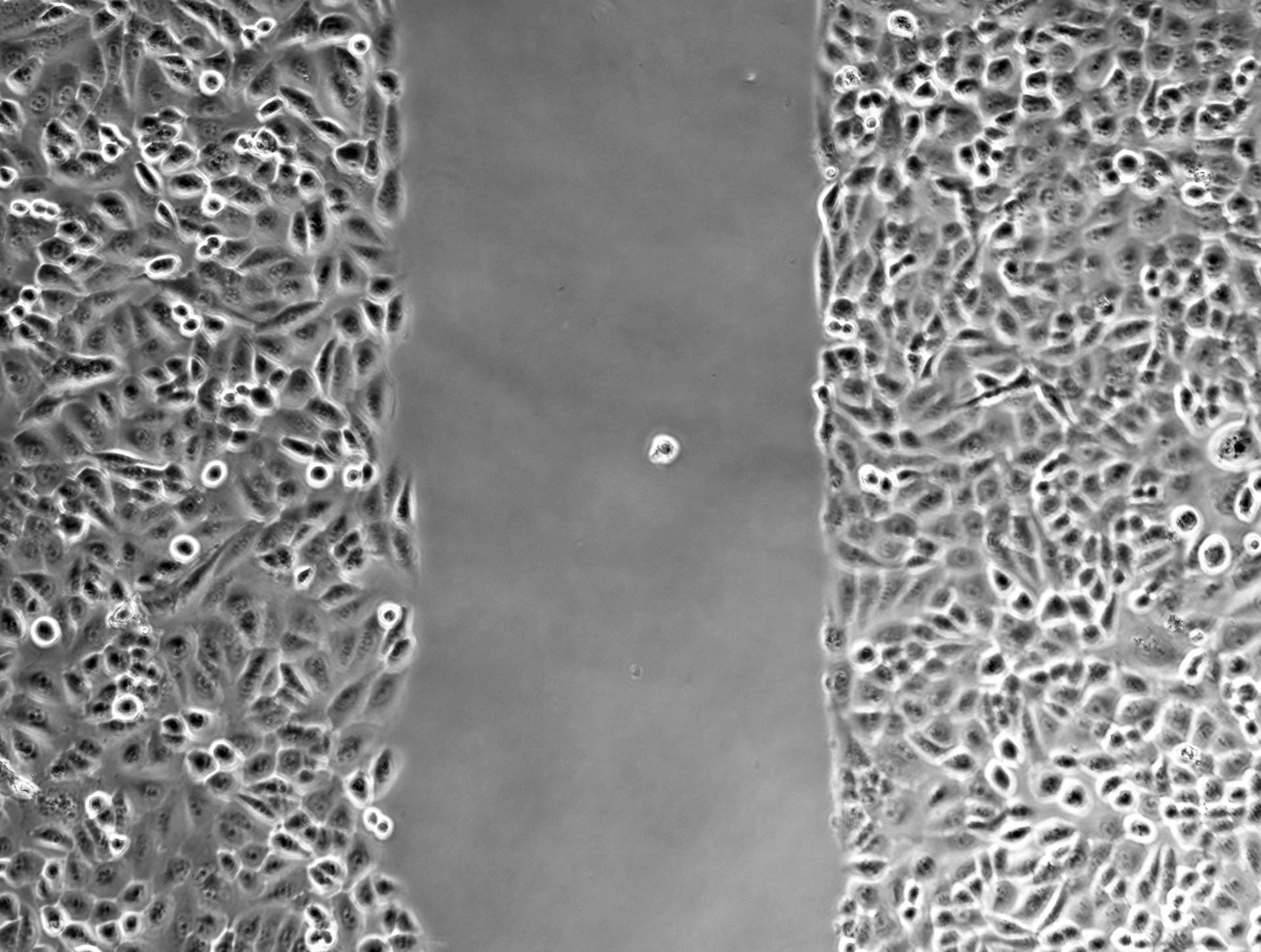

Supplement: Supplementary file 7 — Source Data for Figure 1 [file EMMM-14-e15677-s006.zip › Figure 1/Fig 1B-SKRKO (0).jpg]

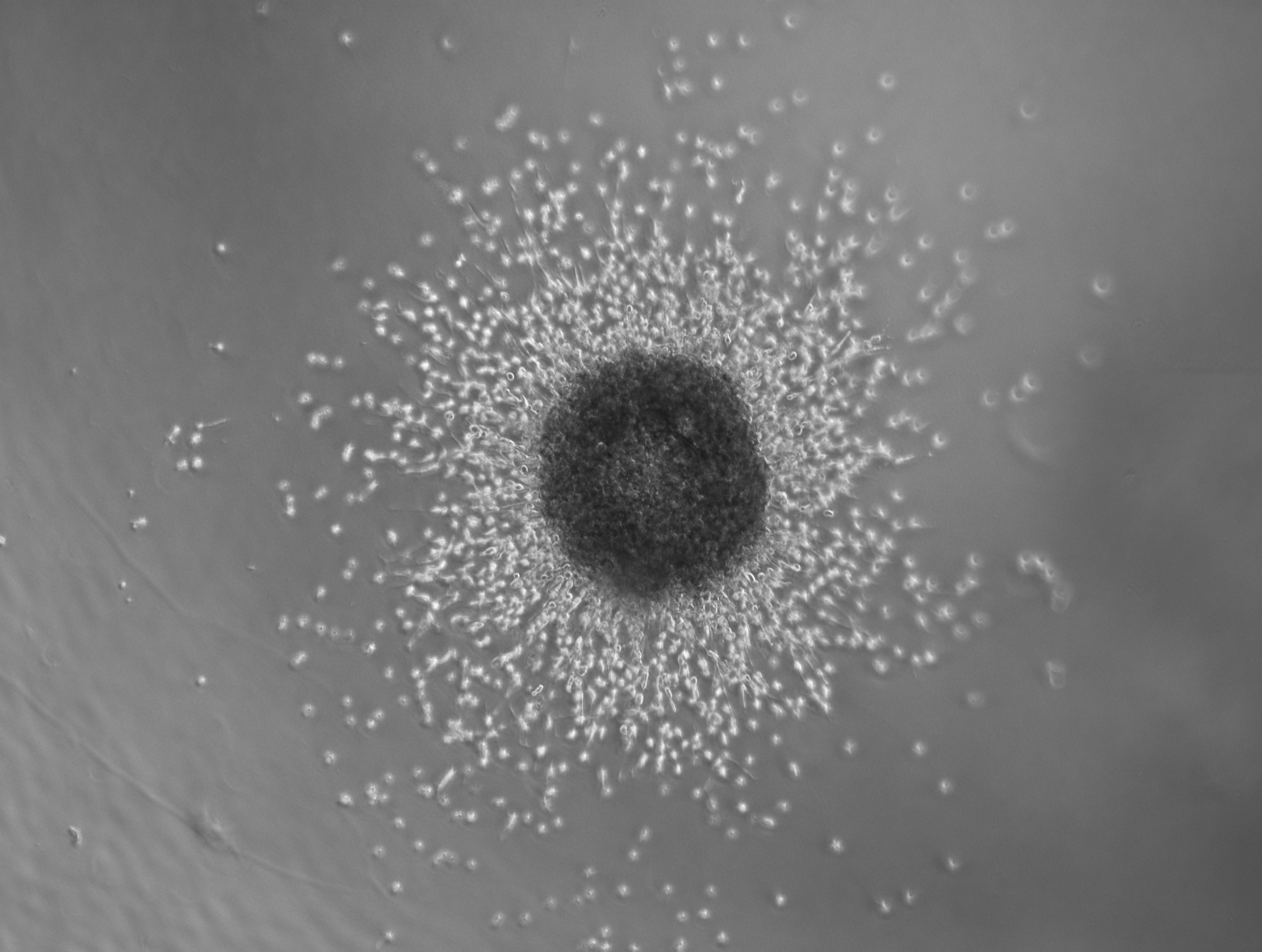

Supplement: Supplementary file 7 — Source Data for Figure 1 [file EMMM-14-e15677-s006.zip › Figure 1/Fig 1E-SKRKO (CH, D4).jpg]

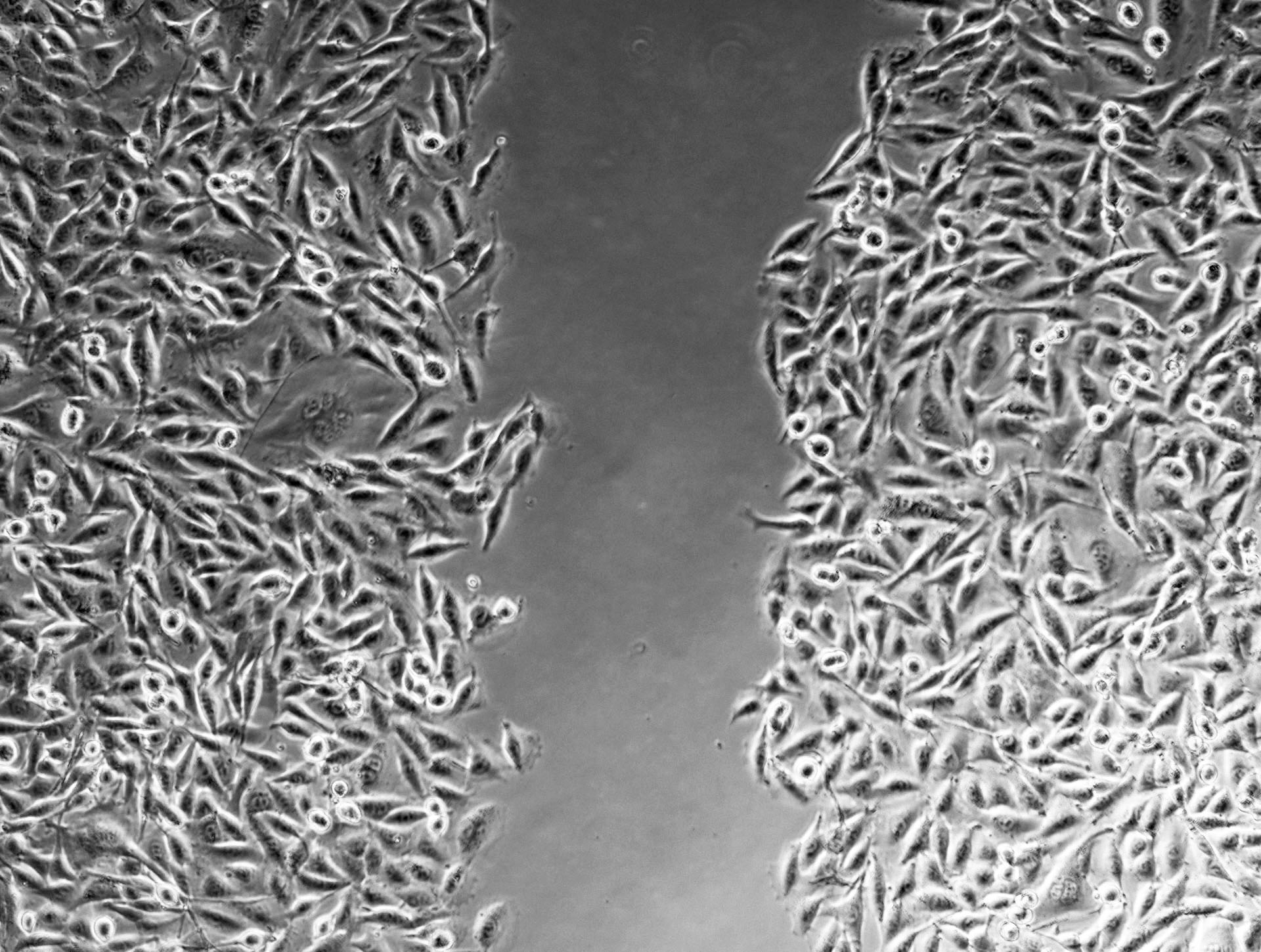

Supplement: Supplementary file 7 — Source Data for Figure 1 [file EMMM-14-e15677-s006.zip › Figure 1/Fig 1B-SKSKO (15h).jpg]

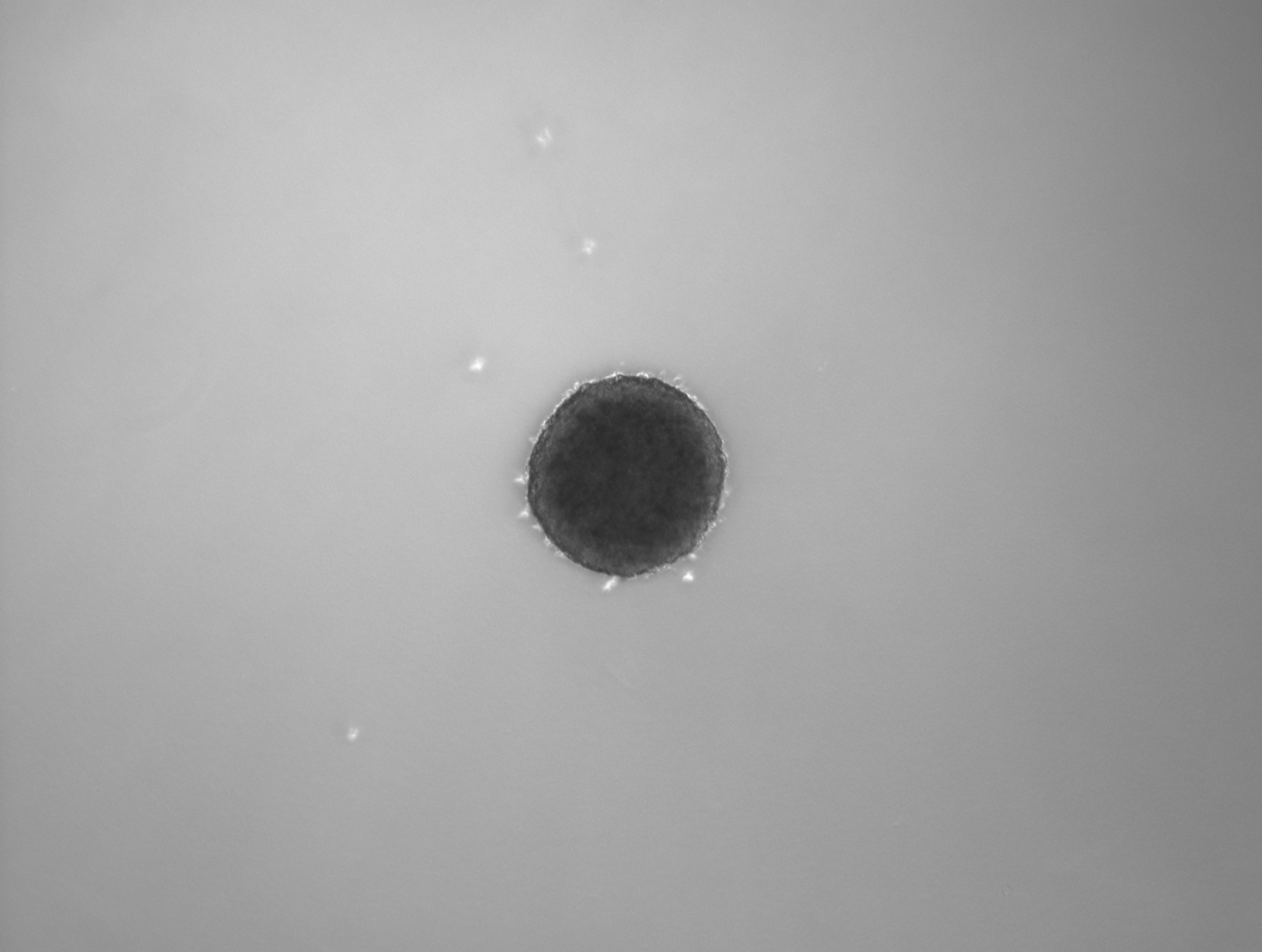

Supplement: Supplementary file 7 — Source Data for Figure 1 [file EMMM-14-e15677-s006.zip › Figure 1/Fig 1D-SKR (D0).jpg]

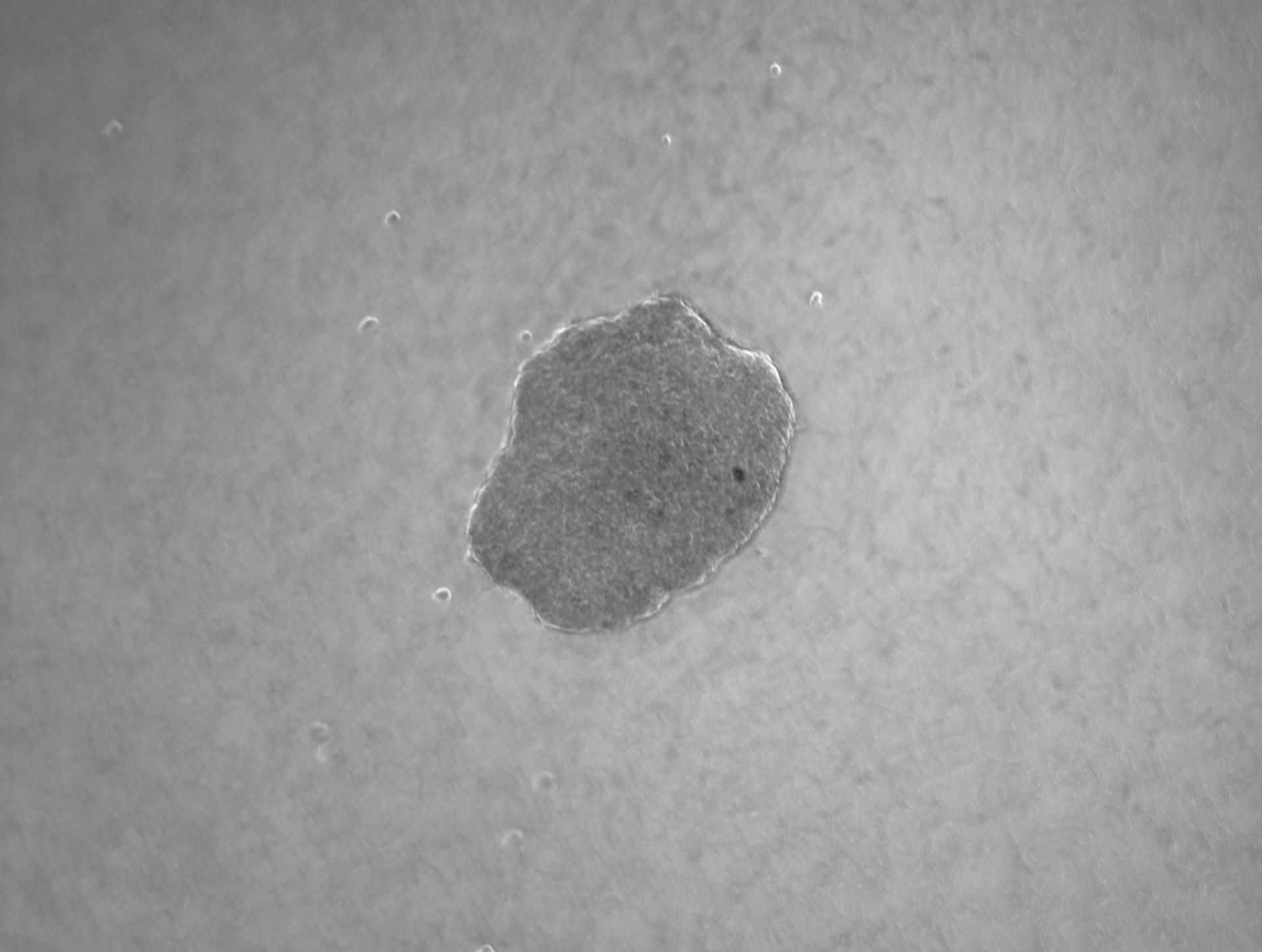

Supplement: Supplementary file 7 — Source Data for Figure 1 [file EMMM-14-e15677-s006.zip › Figure 1/Fig 1D-SKS (D0).jpg]

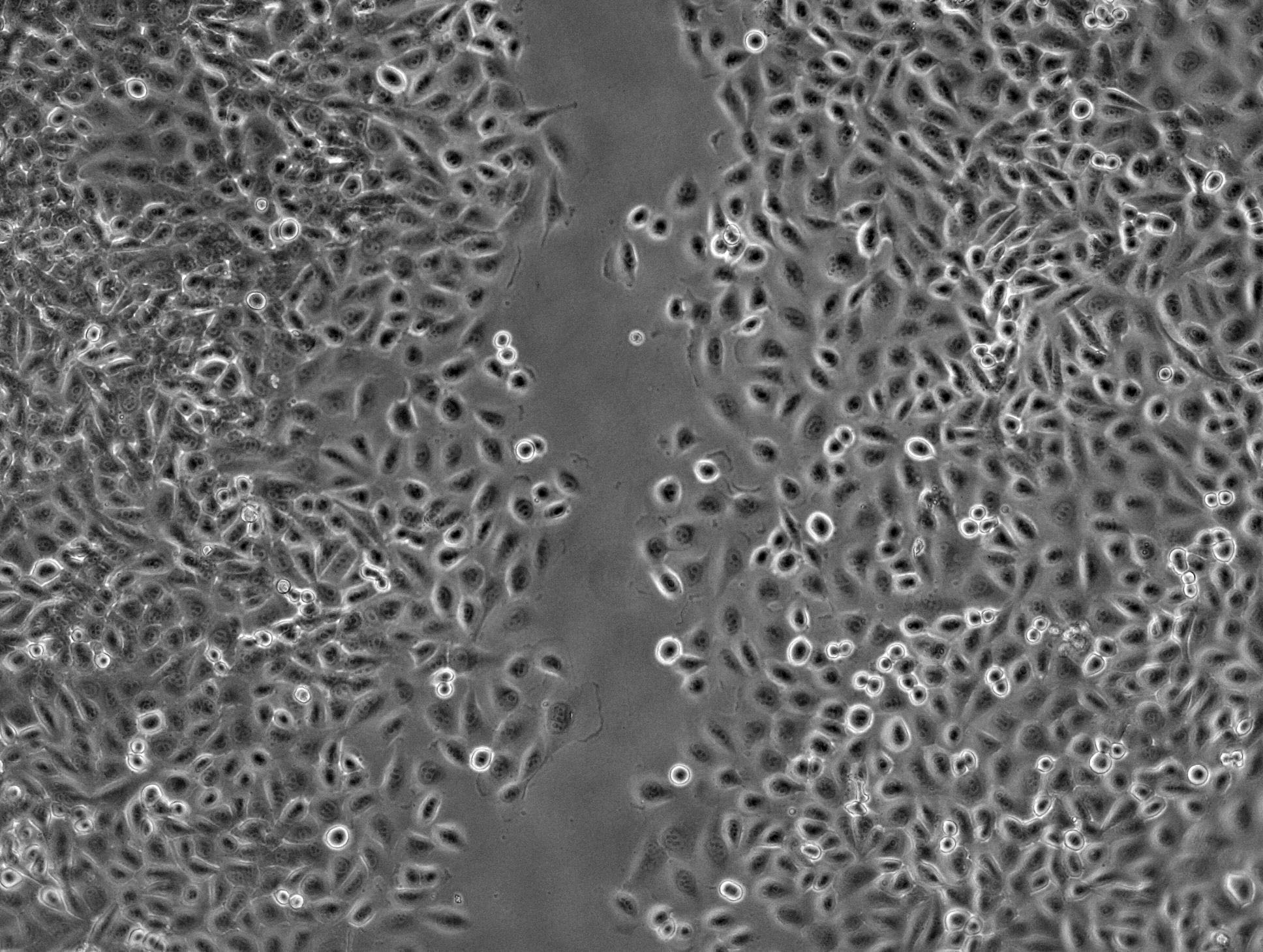

Supplement: Supplementary file 7 — Source Data for Figure 1 [file EMMM-14-e15677-s006.zip › Figure 1/Fig 1C-SKR(DMSO, 15h).jpg]

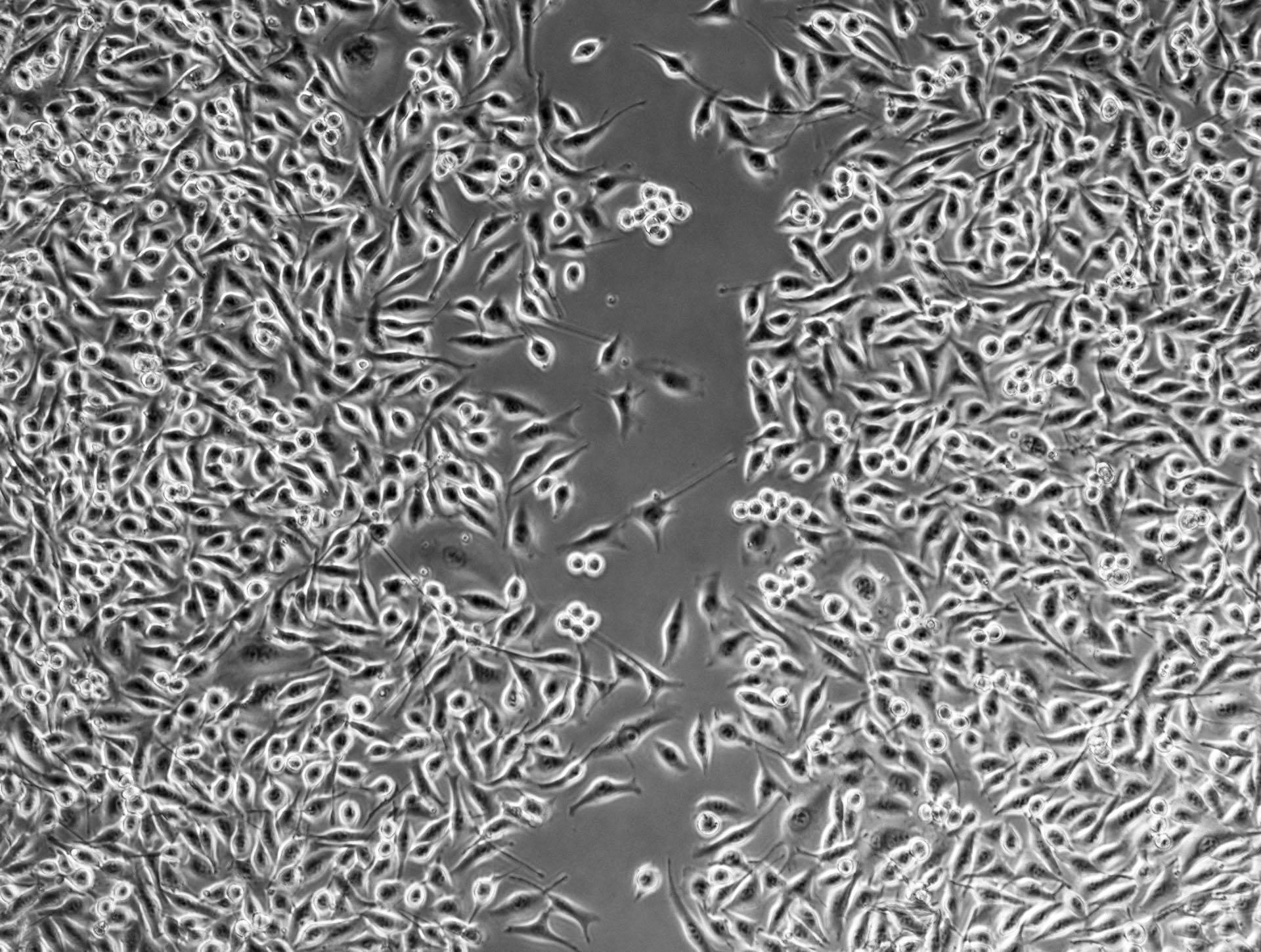

Supplement: Supplementary file 7 — Source Data for Figure 1 [file EMMM-14-e15677-s006.zip › Figure 1/Fig 1B-SKS (15h).jpg]

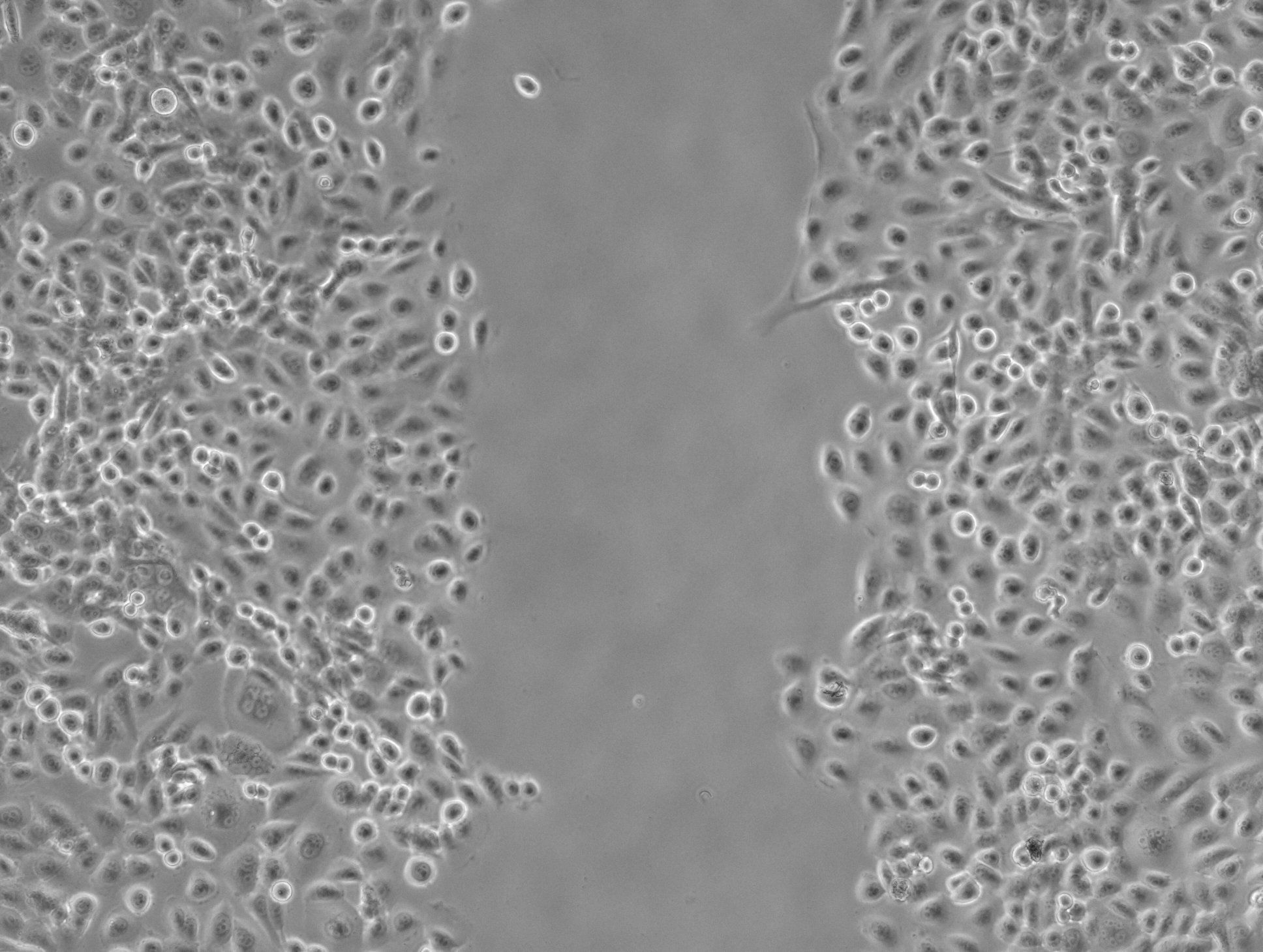

Supplement: Supplementary file 7 — Source Data for Figure 1 [file EMMM-14-e15677-s006.zip › Figure 1/Fig 1C-SKRKO (DMSO, 15h).jpg]

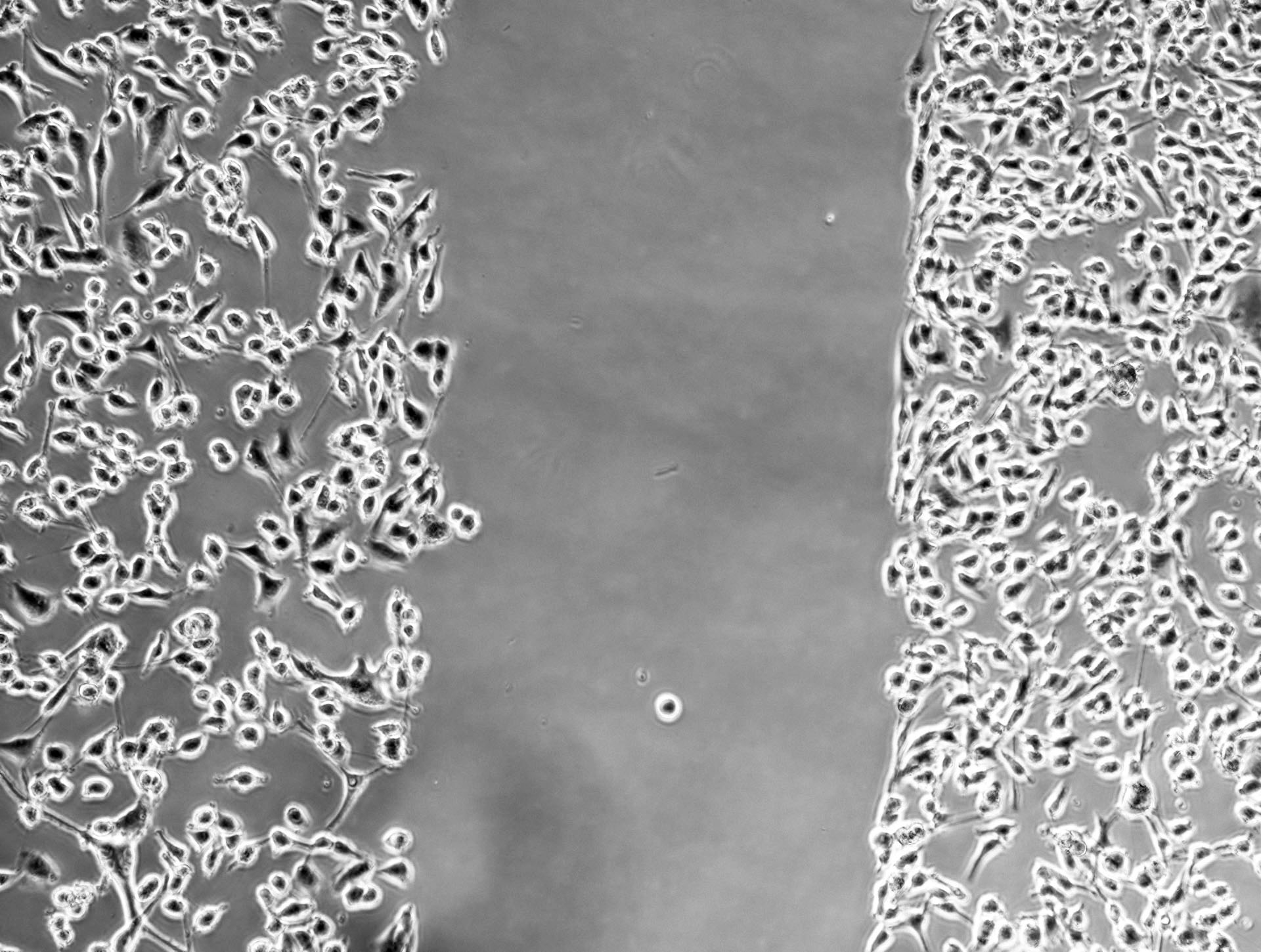

Supplement: Supplementary file 7 — Source Data for Figure 1 [file EMMM-14-e15677-s006.zip › Figure 1/Fig 1B-SKSKO (0).jpg]

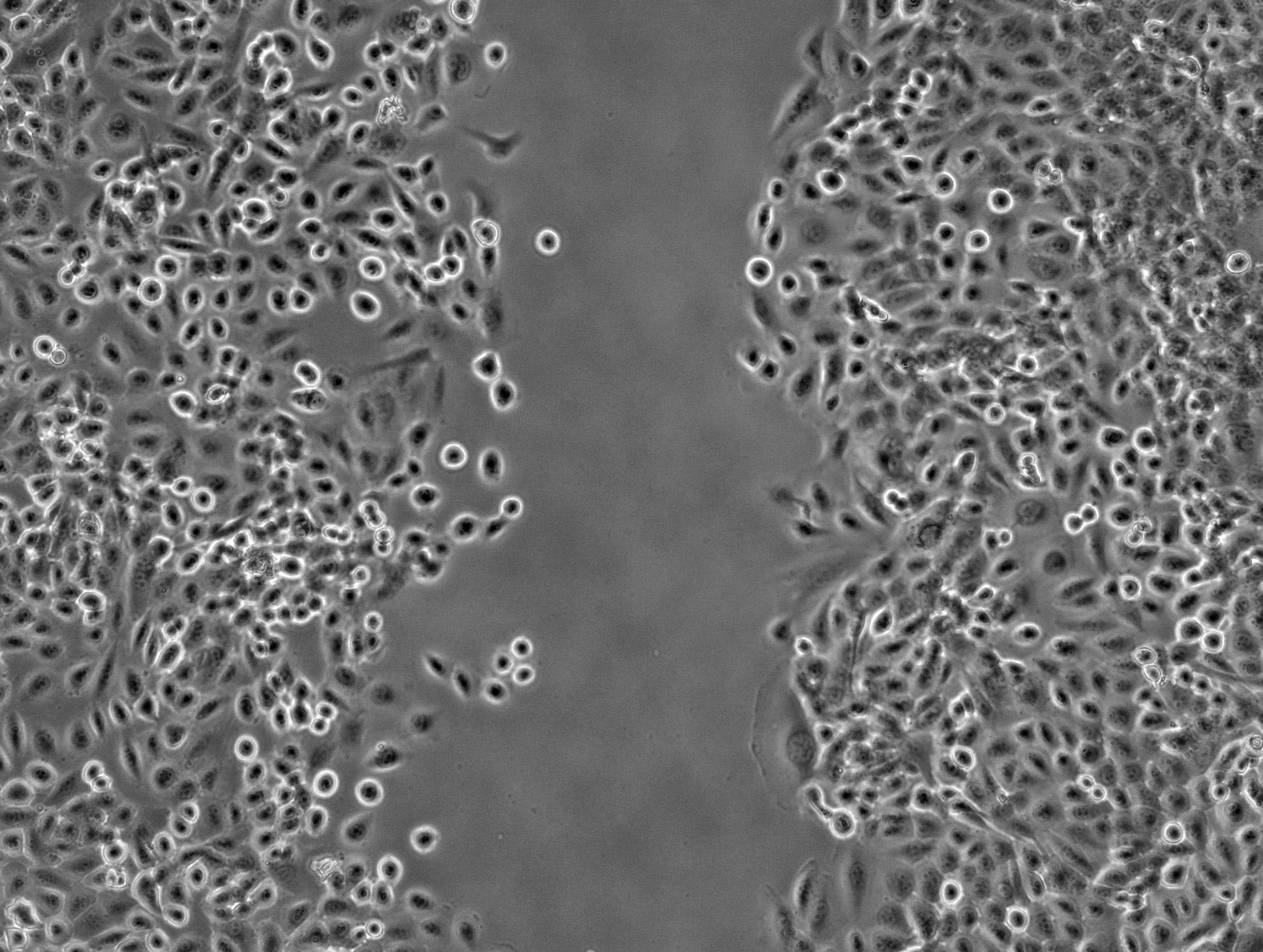

Supplement: Supplementary file 7 — Source Data for Figure 1 [file EMMM-14-e15677-s006.zip › Figure 1/Fig 1C-SKR (CH, 15h).jpg]

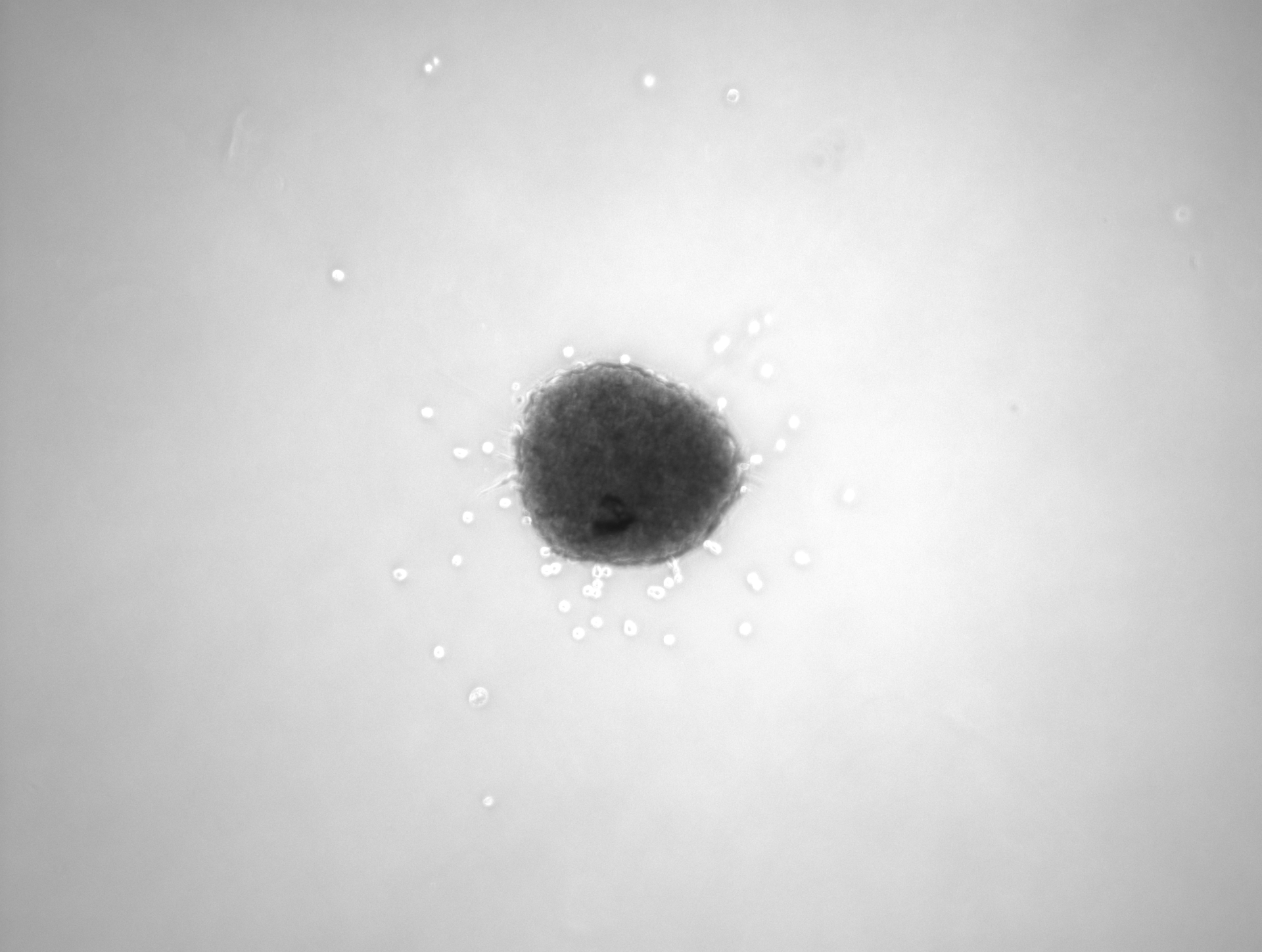

Supplement: Supplementary file 7 — Source Data for Figure 1 [file EMMM-14-e15677-s006.zip › Figure 1/Fig 1E-SKRKO (DMSO, D0).jpg]

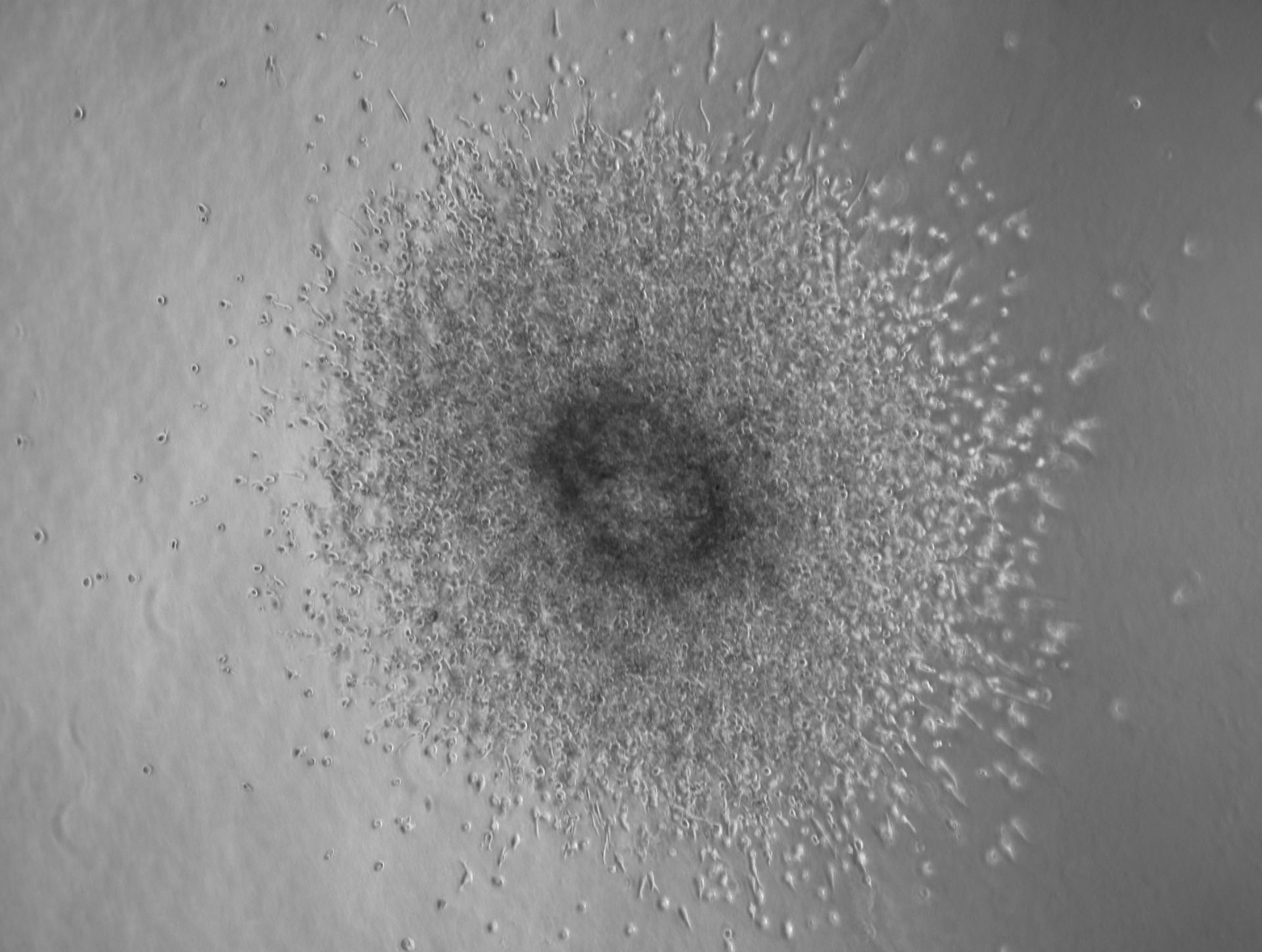

Supplement: Supplementary file 7 — Source Data for Figure 1 [file EMMM-14-e15677-s006.zip › Figure 1/Fig 1E-SKR (CH, D4).jpg]

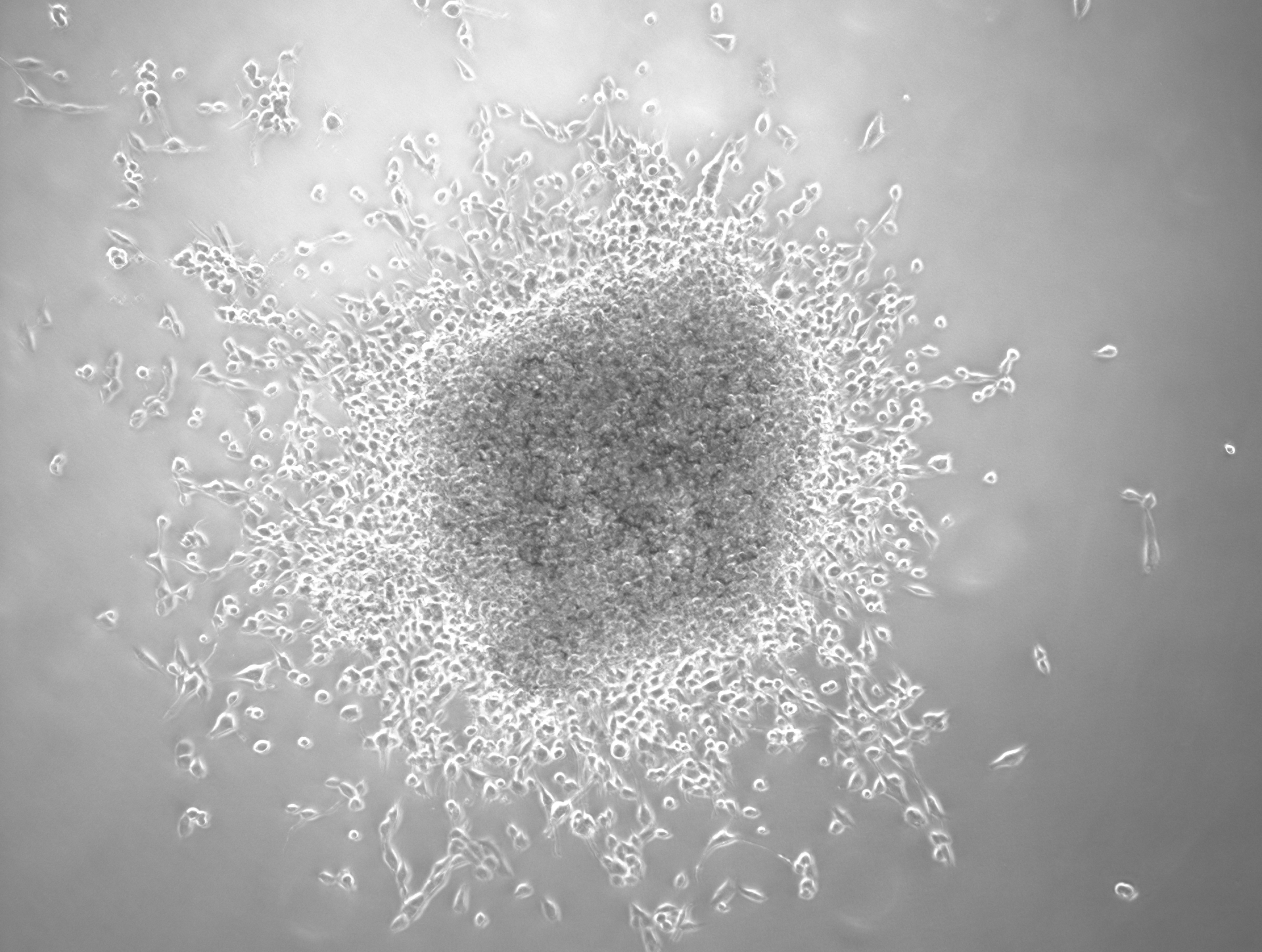

Supplement: Supplementary file 7 — Source Data for Figure 1 [file EMMM-14-e15677-s006.zip › Figure 1/Fig 1D-SKSKO (D4).jpg]

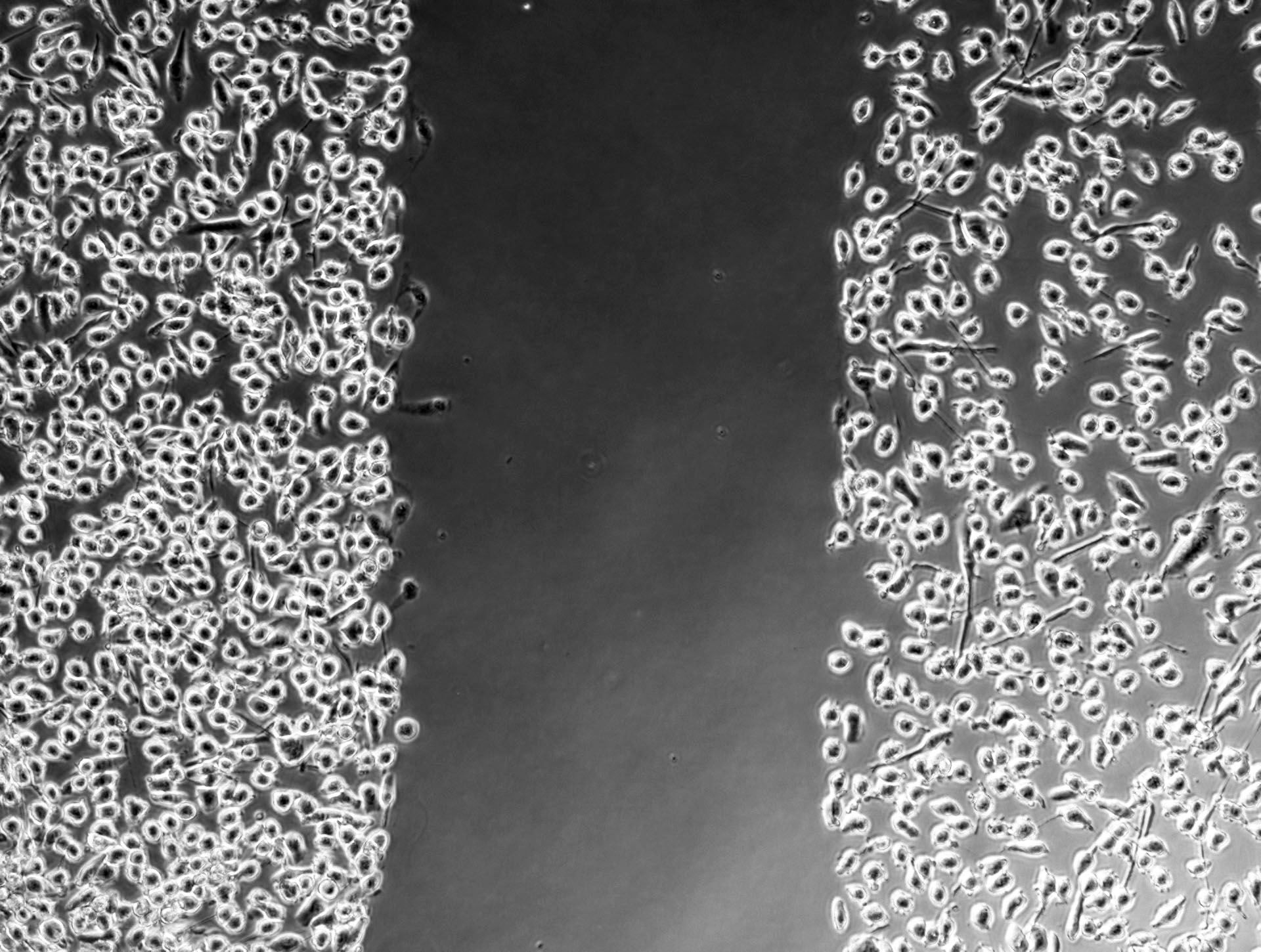

Supplement: Supplementary file 7 — Source Data for Figure 1 [file EMMM-14-e15677-s006.zip › Figure 1/Fig 1B-SKS (0).jpg]

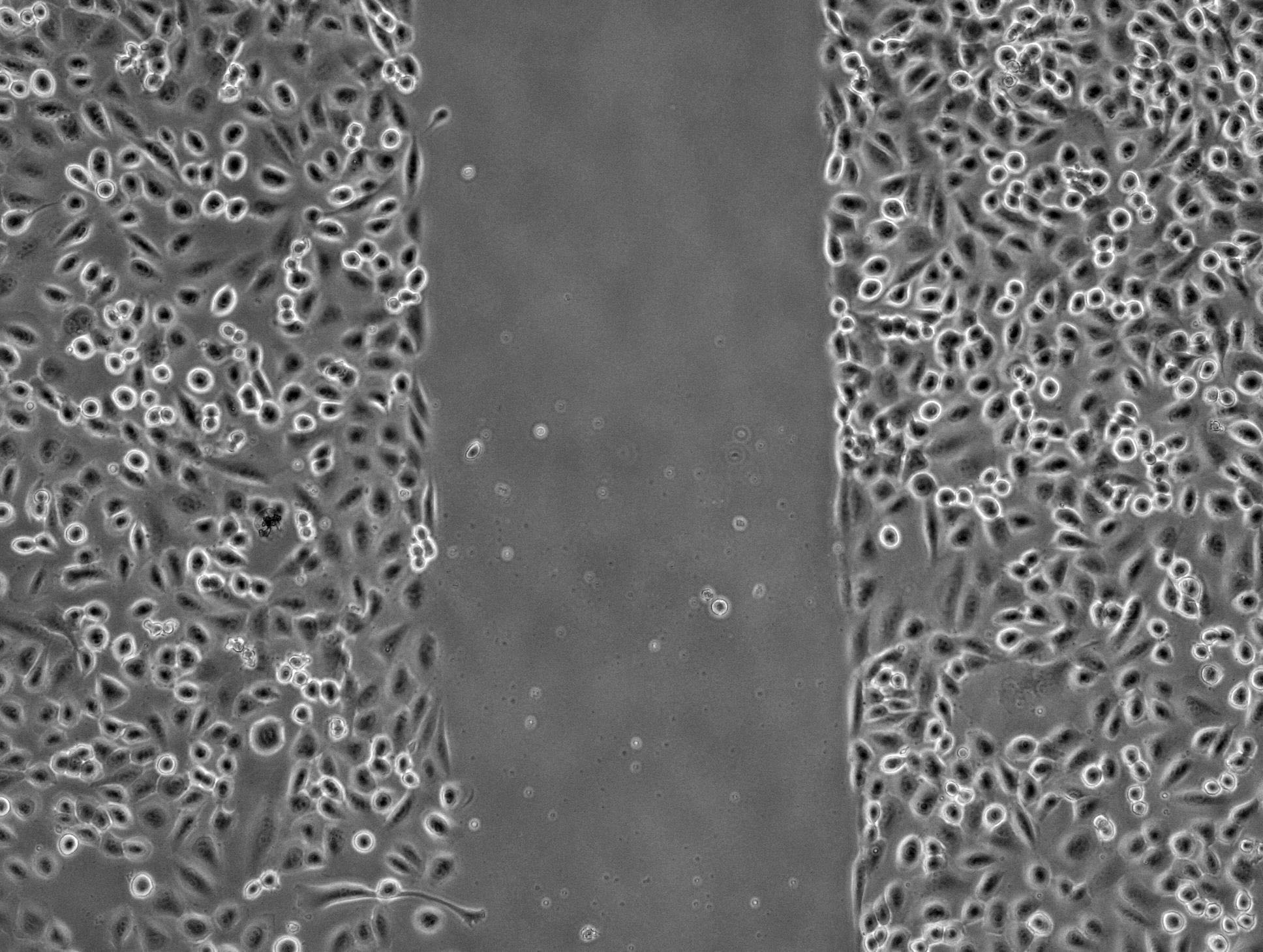

Supplement: Supplementary file 7 — Source Data for Figure 1 [file EMMM-14-e15677-s006.zip › Figure 1/Fig 1C-SKR (CH, 0).jpg]

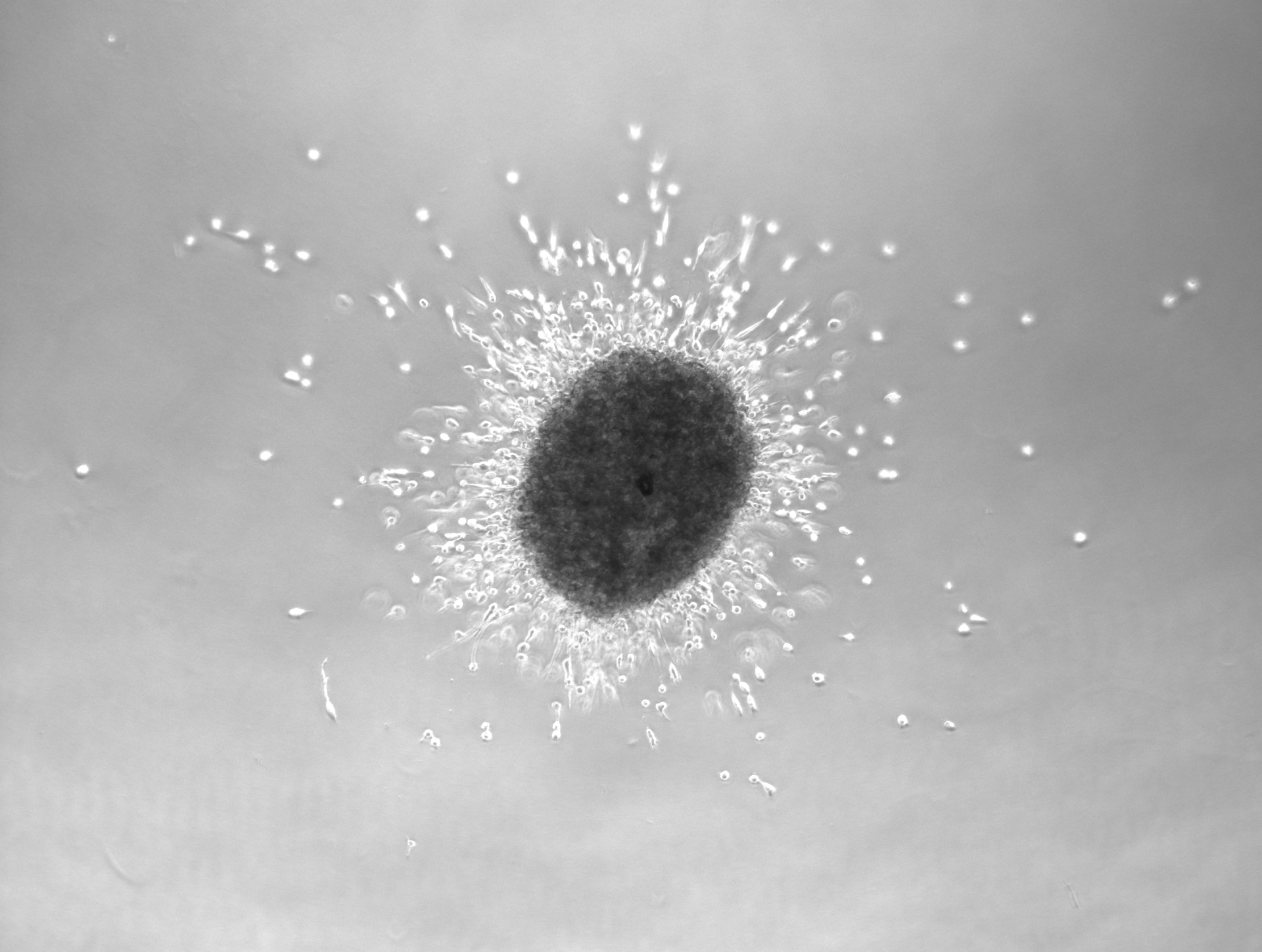

Supplement: Supplementary file 7 — Source Data for Figure 1 [file EMMM-14-e15677-s006.zip › Figure 1/Fig 1D-SKRKO (D0).jpg]

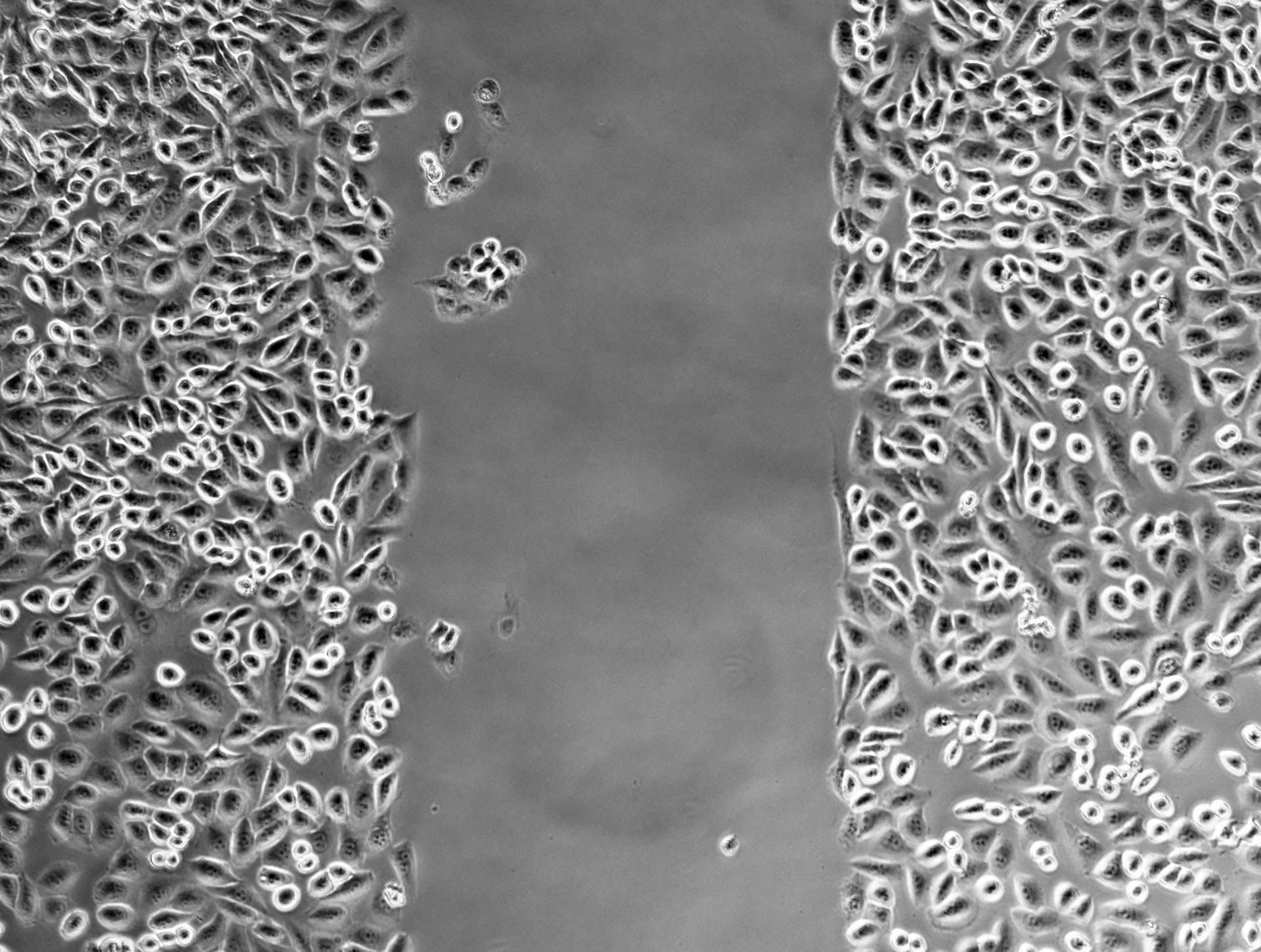

Supplement: Supplementary file 7 — Source Data for Figure 1 [file EMMM-14-e15677-s006.zip › Figure 1/Fig 1B-SKR (0).jpg]

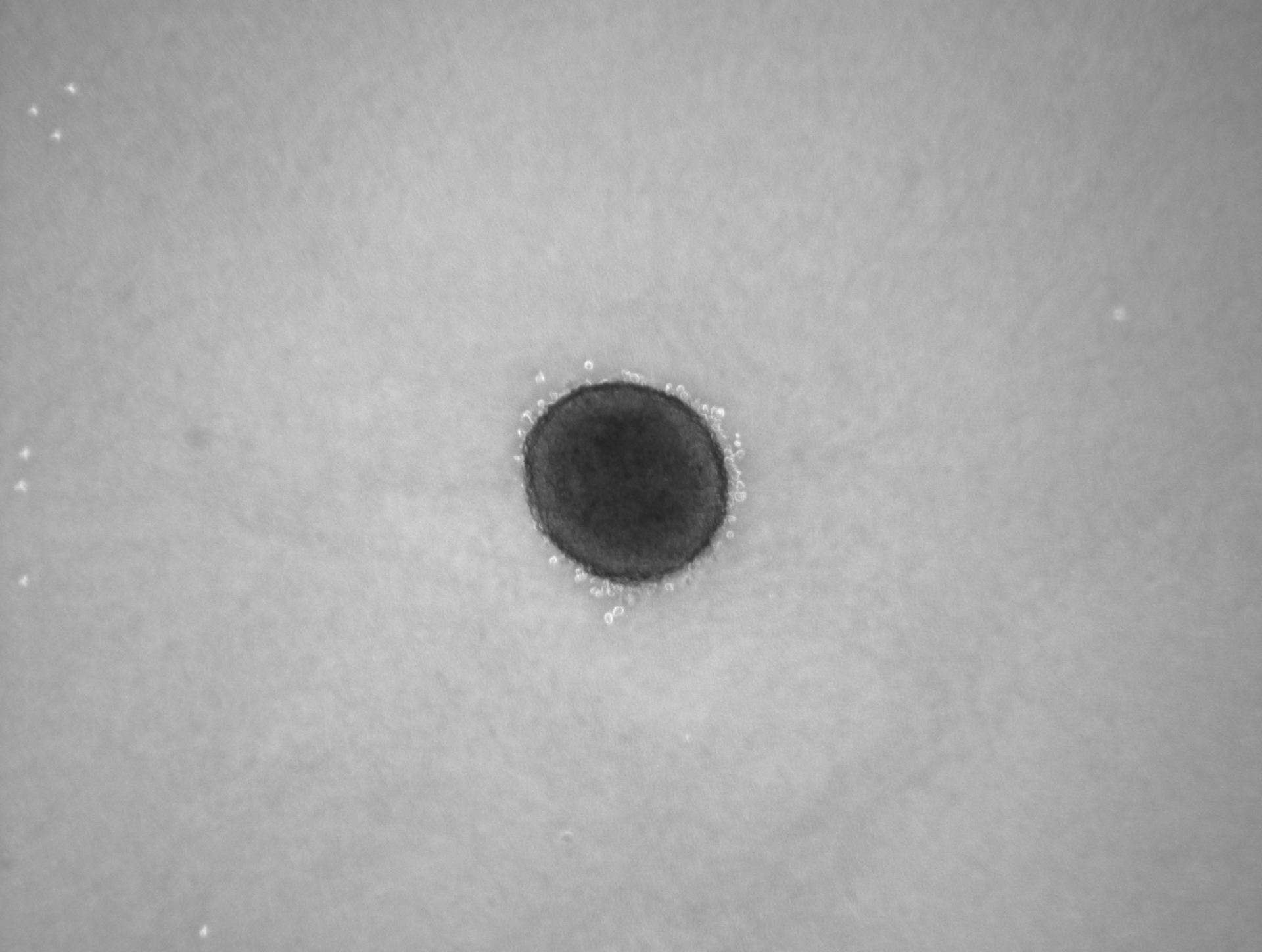

Supplement: Supplementary file 7 — Source Data for Figure 1 [file EMMM-14-e15677-s006.zip › Figure 1/Fig 1E-SKR (DMSO, D0).jpg]

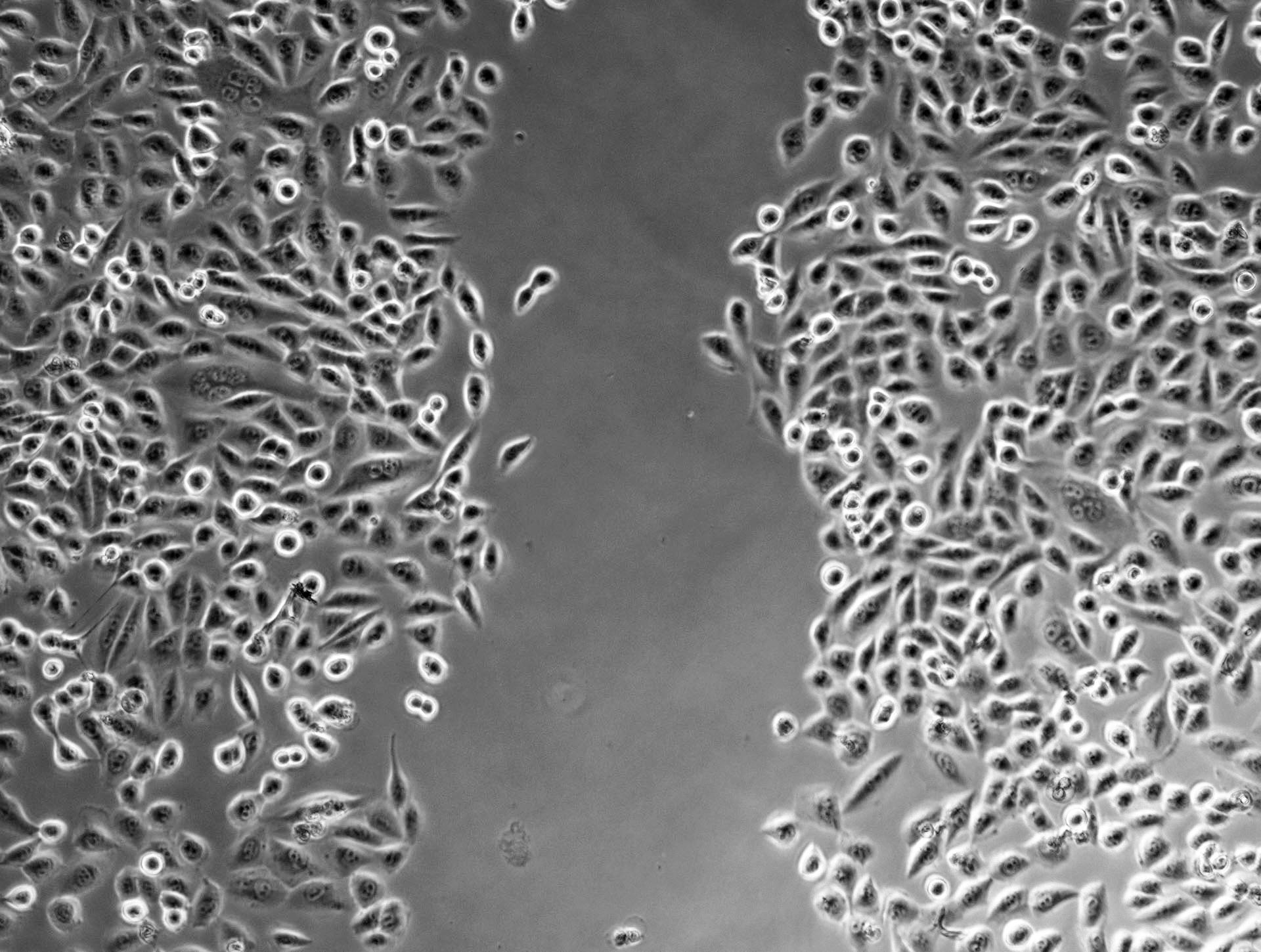

Supplement: Supplementary file 7 — Source Data for Figure 1 [file EMMM-14-e15677-s006.zip › Figure 1/Fig 1B-SKR (15h).jpg]

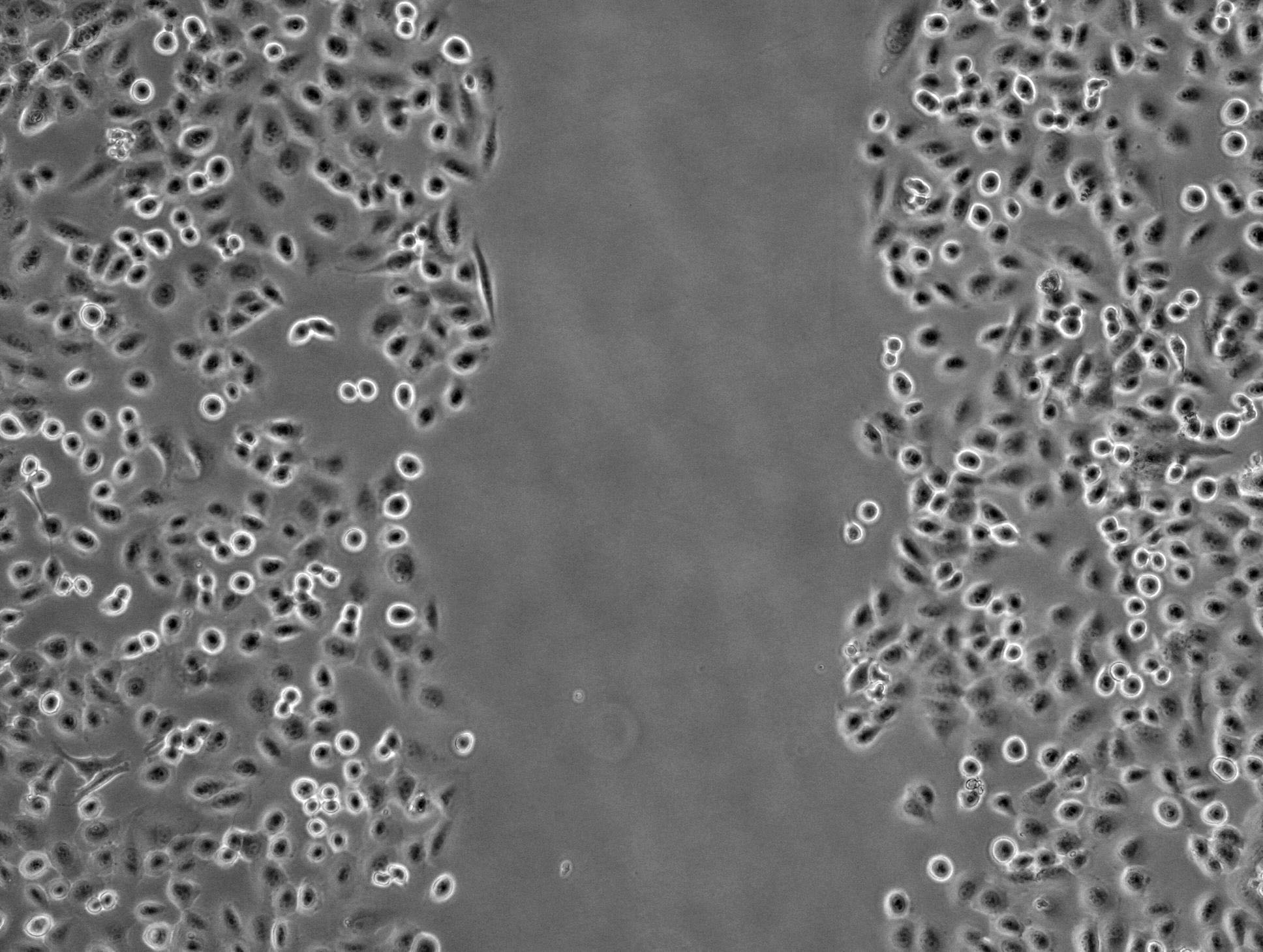

Supplement: Supplementary file 7 — Source Data for Figure 1 [file EMMM-14-e15677-s006.zip › Figure 1/Fig 1C-SKRKO (CH, 0).jpg]

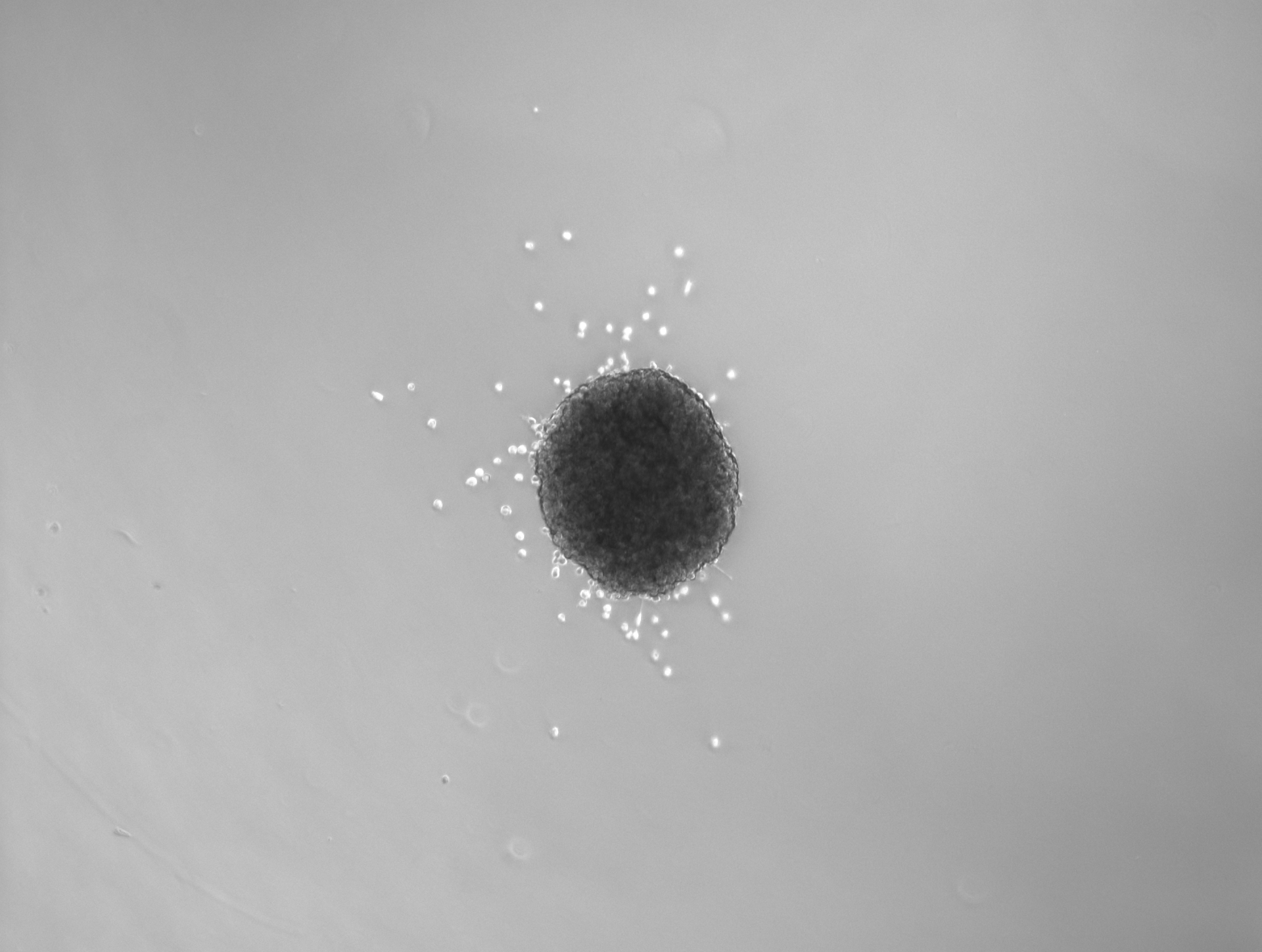

Supplement: Supplementary file 7 — Source Data for Figure 1 [file EMMM-14-e15677-s006.zip › Figure 1/Fig 1E-SKRKO (CH, D0).jpg]

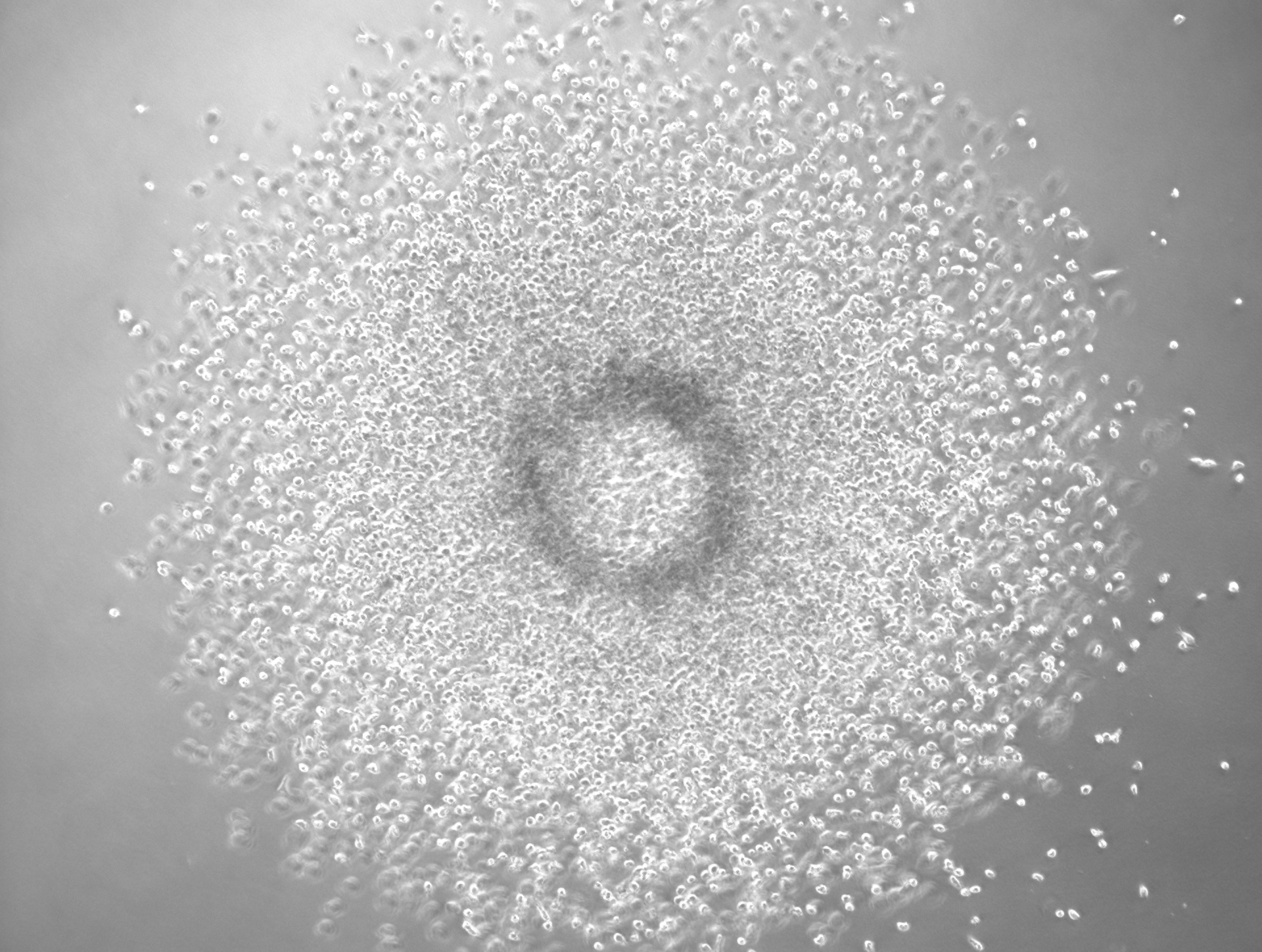

Supplement: Supplementary file 7 — Source Data for Figure 1 [file EMMM-14-e15677-s006.zip › Figure 1/Fig 1D-SKR (D4).jpg]

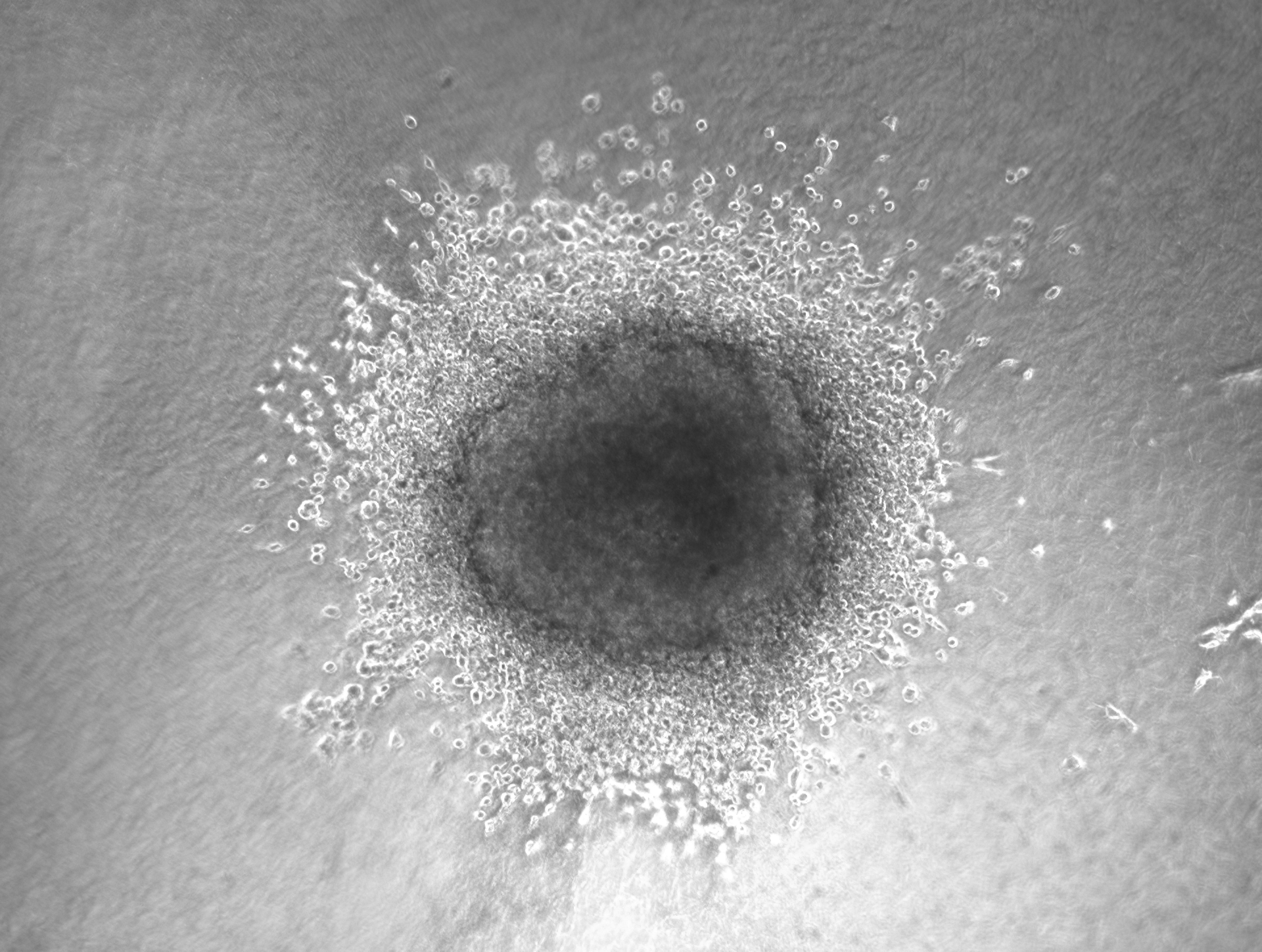

Supplement: Supplementary file 7 — Source Data for Figure 1 [file EMMM-14-e15677-s006.zip › Figure 1/Fig 1D-SKS (D4).jpg]

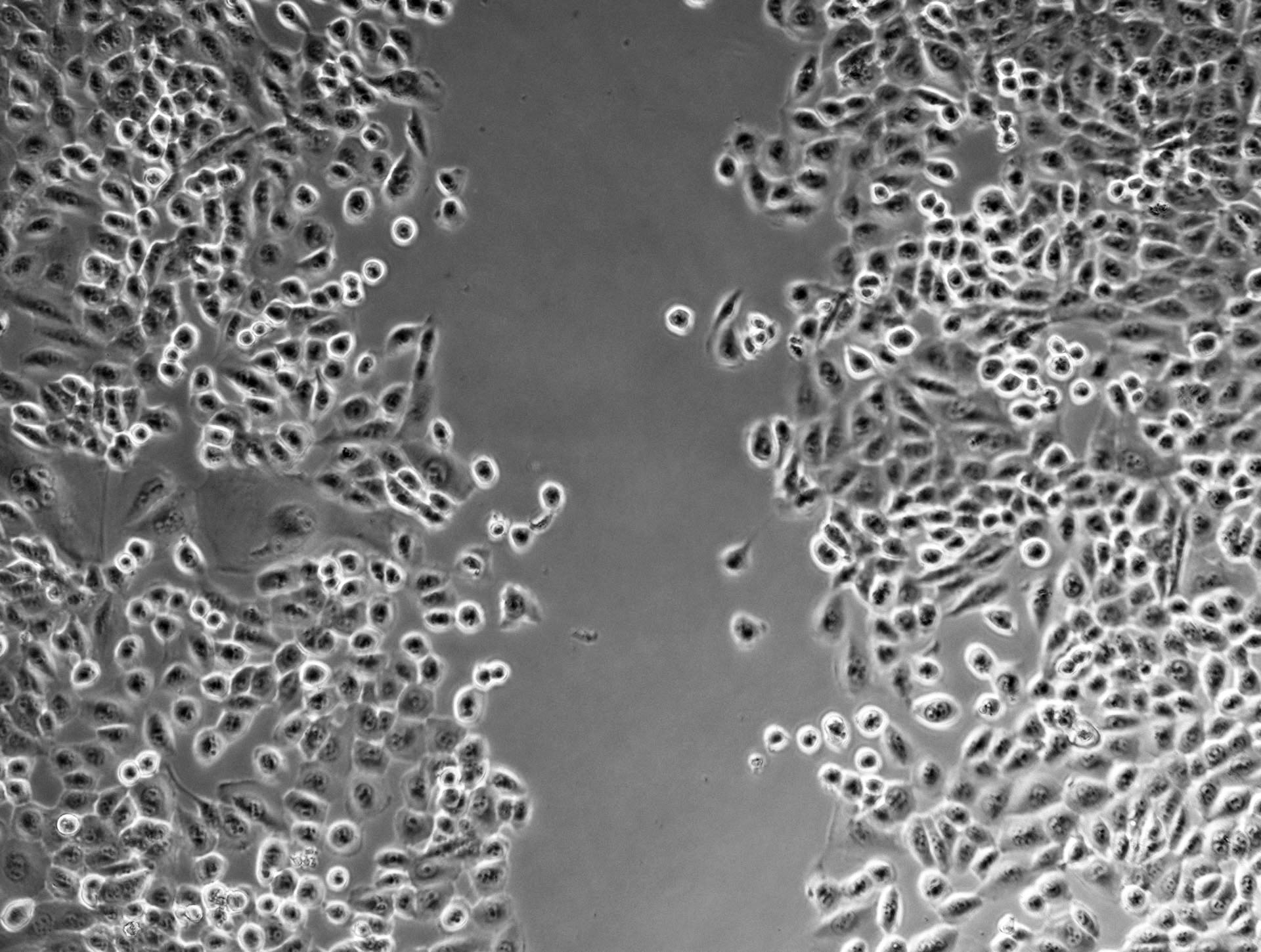

Supplement: Supplementary file 7 — Source Data for Figure 1 [file EMMM-14-e15677-s006.zip › Figure 1/Fig 1B-SKRKO (15h).jpg]

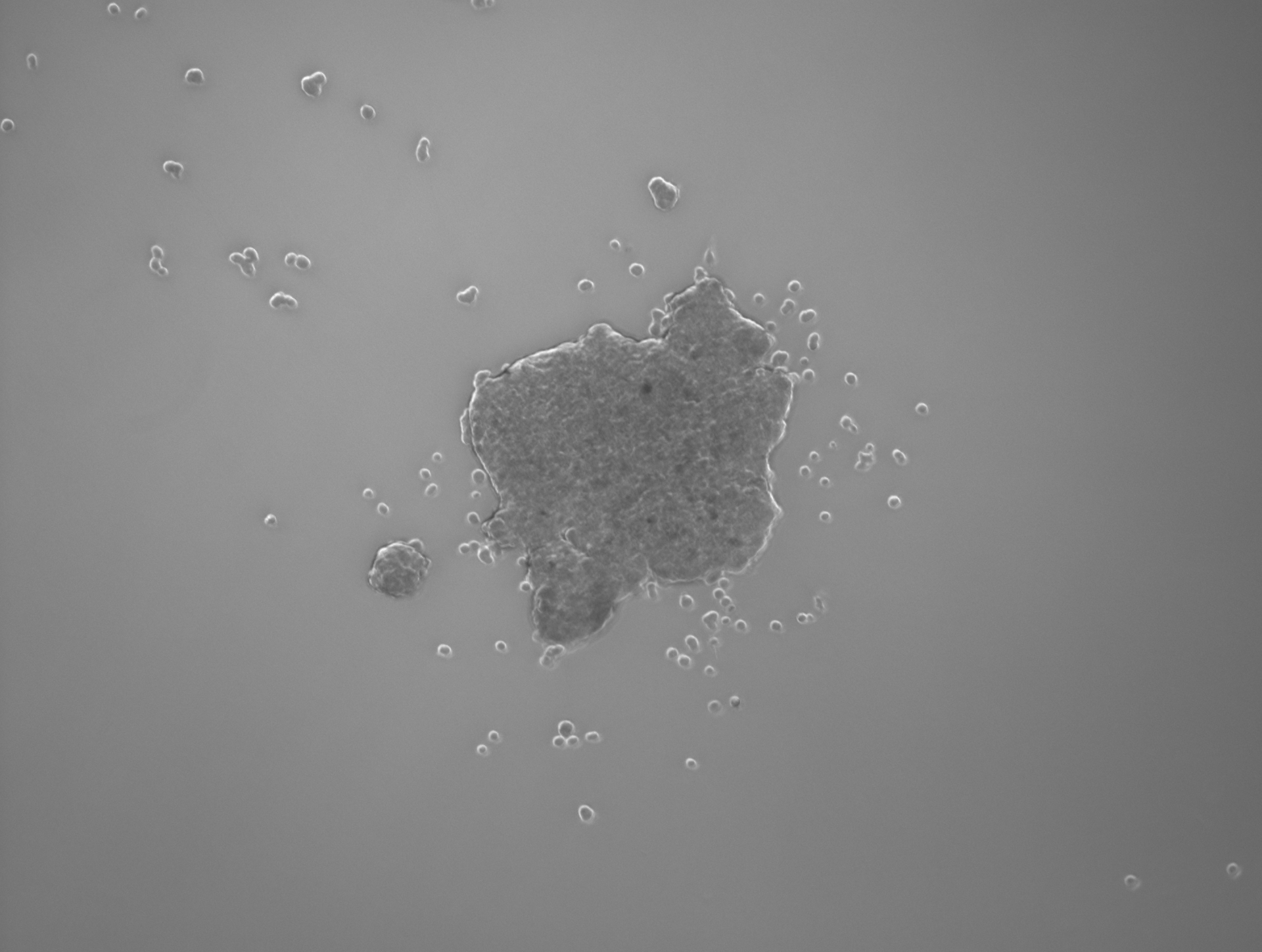

Supplement: Supplementary file 7 — Source Data for Figure 1 [file EMMM-14-e15677-s006.zip › Figure 1/Fig 1D-SKSKO (D0).jpg]

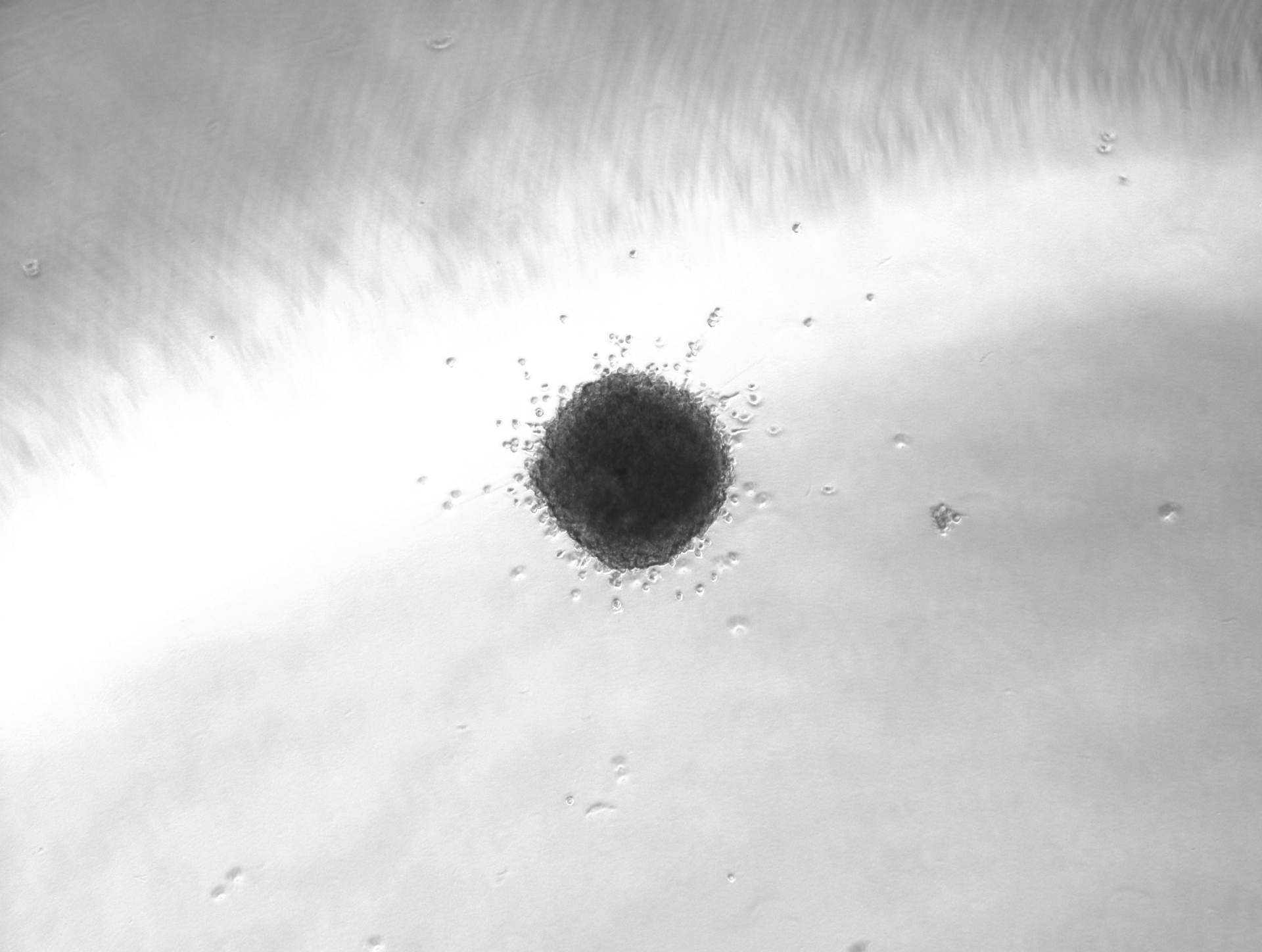

Supplement: Supplementary file 7 — Source Data for Figure 1 [file EMMM-14-e15677-s006.zip › Figure 1/Fig 1E-SKR (CH, D0).jpg]

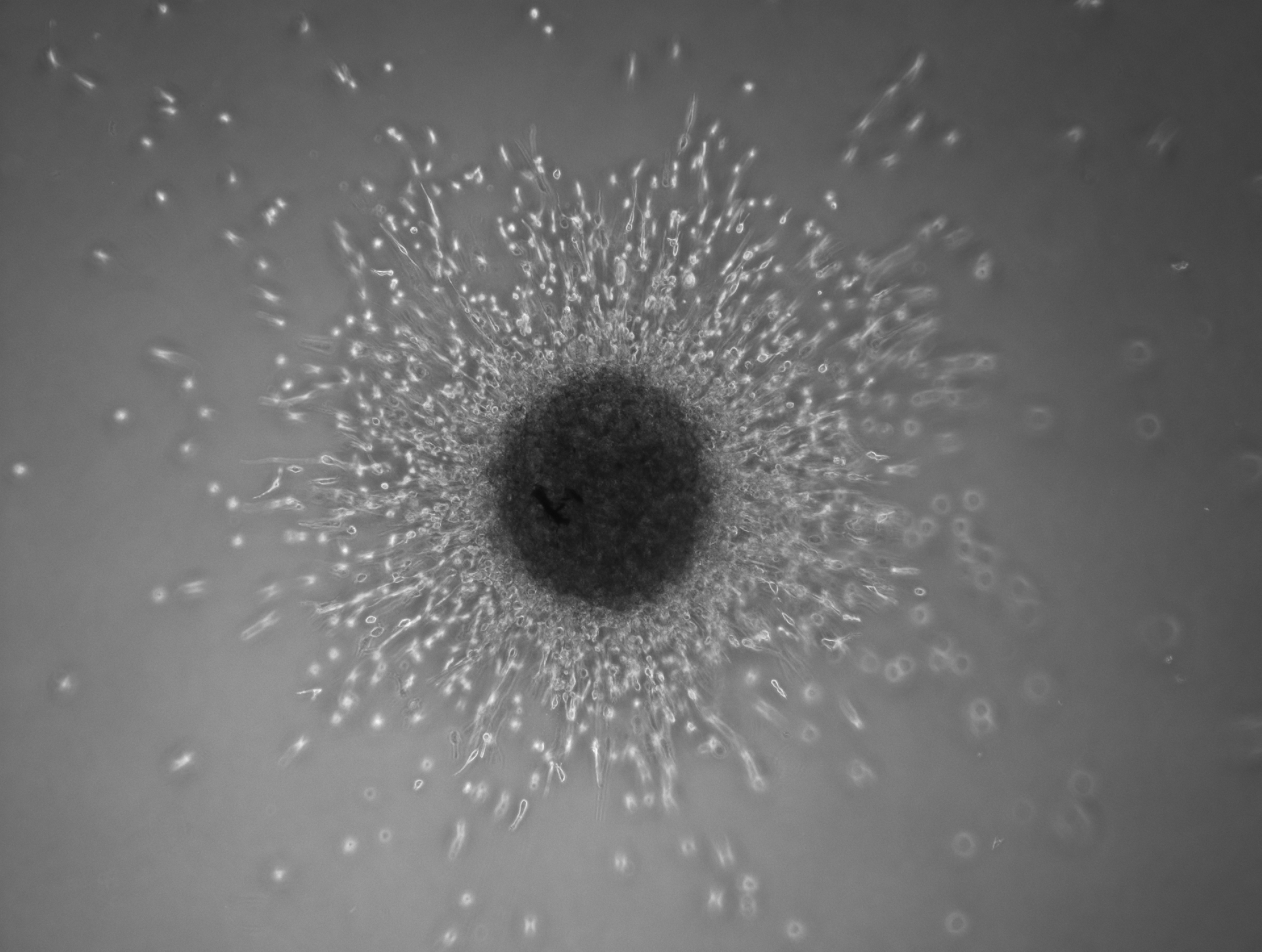

Supplement: Supplementary file 7 — Source Data for Figure 1 [file EMMM-14-e15677-s006.zip › Figure 1/Fig 1E-SKRKO (DMSO, D4).jpg]

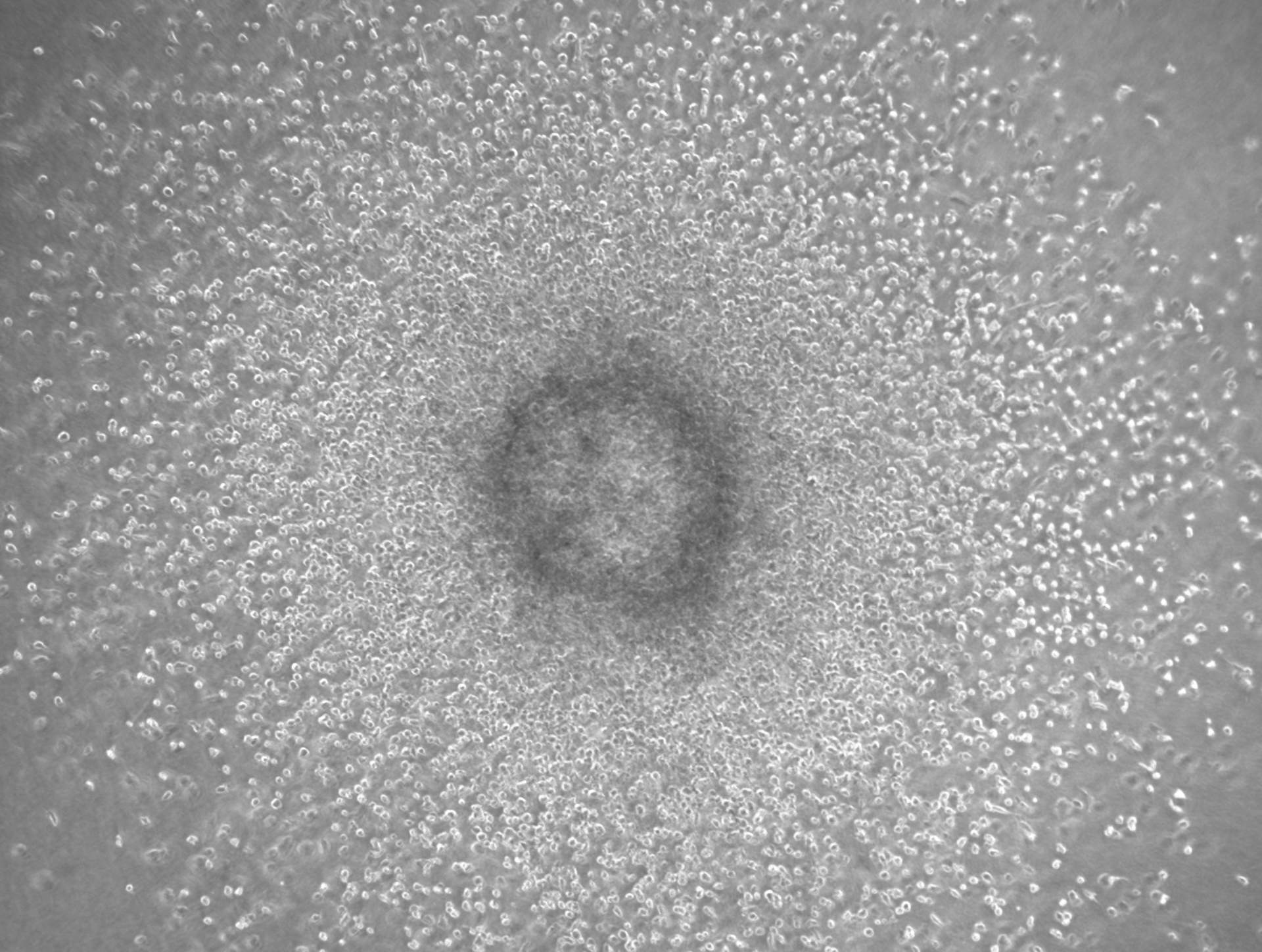

Supplement: Supplementary file 7 — Source Data for Figure 1 [file EMMM-14-e15677-s006.zip › Figure 1/Fig 1E-SKR (DMSO, D4).jpg]

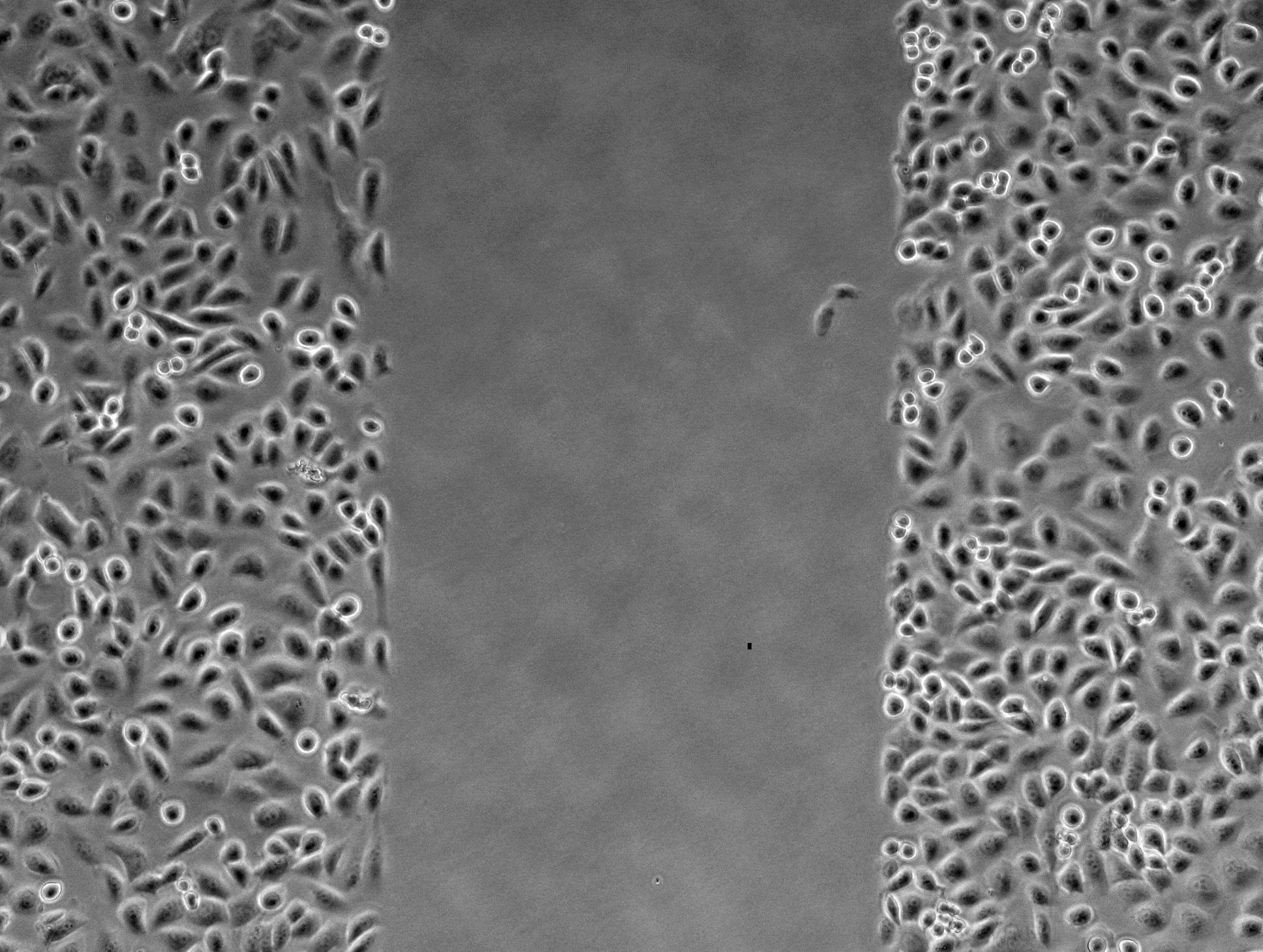

Supplement: Supplementary file 7 — Source Data for Figure 1 [file EMMM-14-e15677-s006.zip › Figure 1/Fig 1C-SKR(DMSO, 0).jpg]

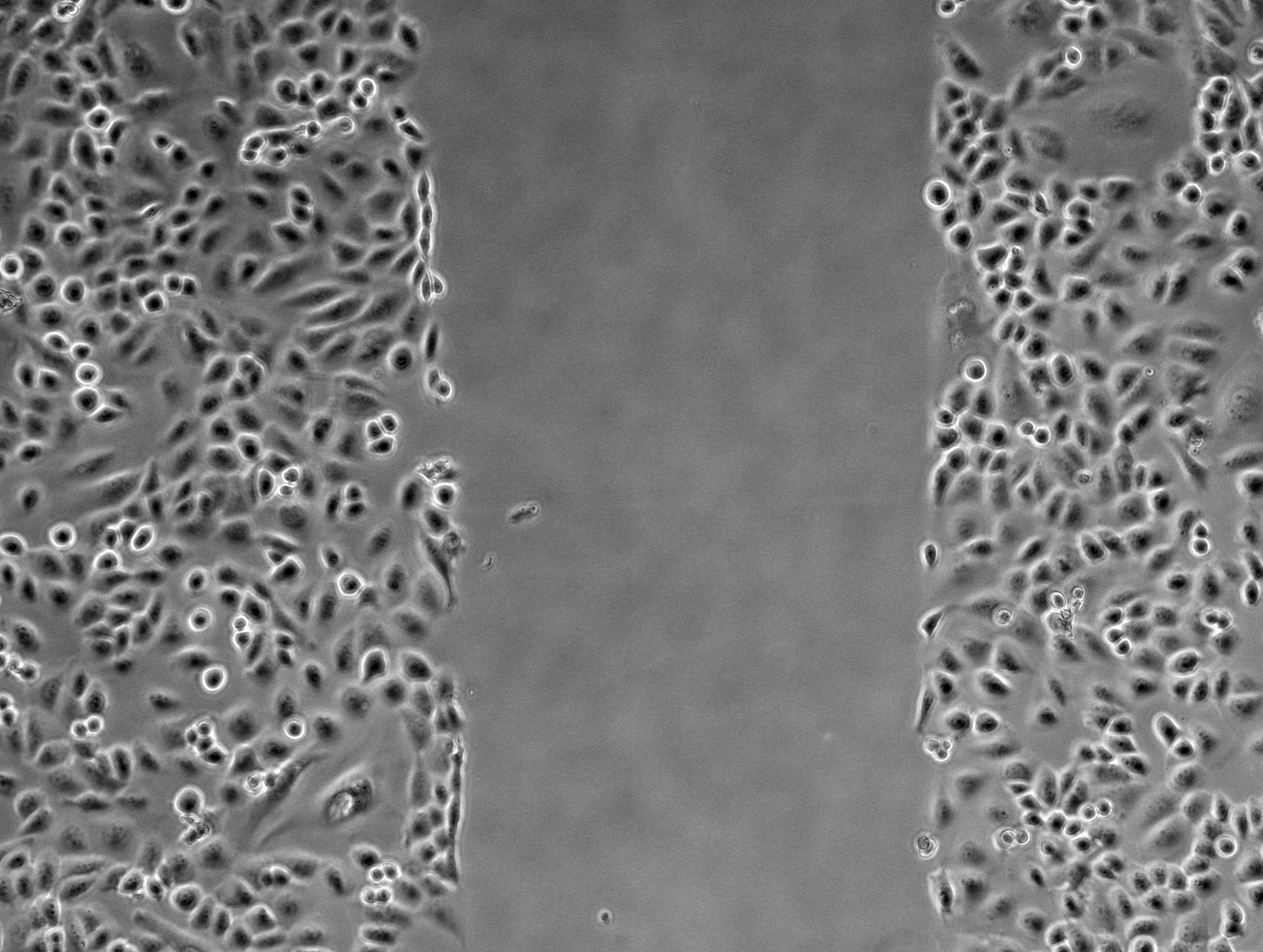

Supplement: Supplementary file 7 — Source Data for Figure 1 [file EMMM-14-e15677-s006.zip › Figure 1/Fig 1C-SKRKO (DMSO, 0).jpg]

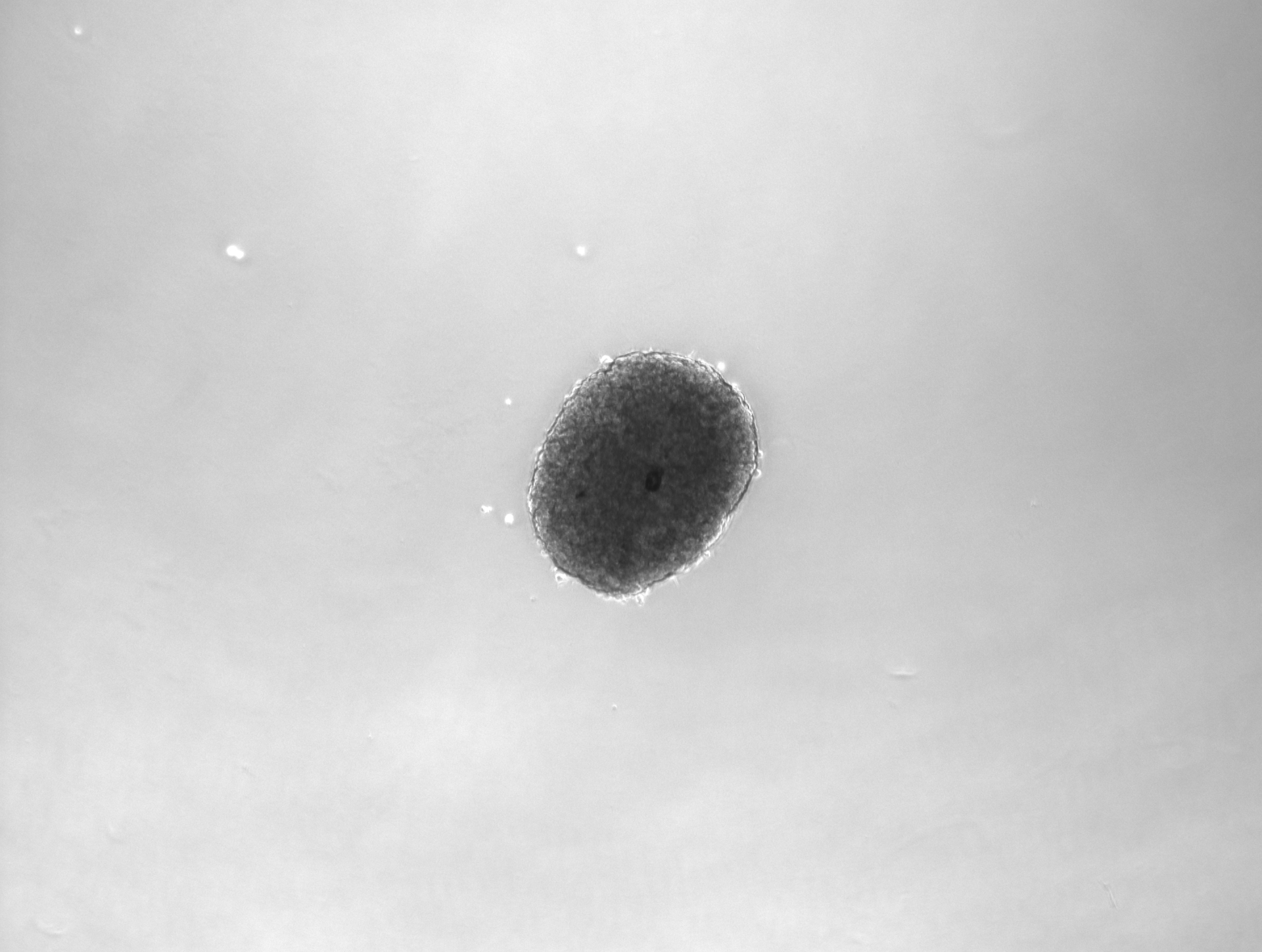

Supplement: Supplementary file 7 — Source Data for Figure 1 [file EMMM-14-e15677-s006.zip › Figure 1/Fig 1D-SKRKO (D4).jpg]

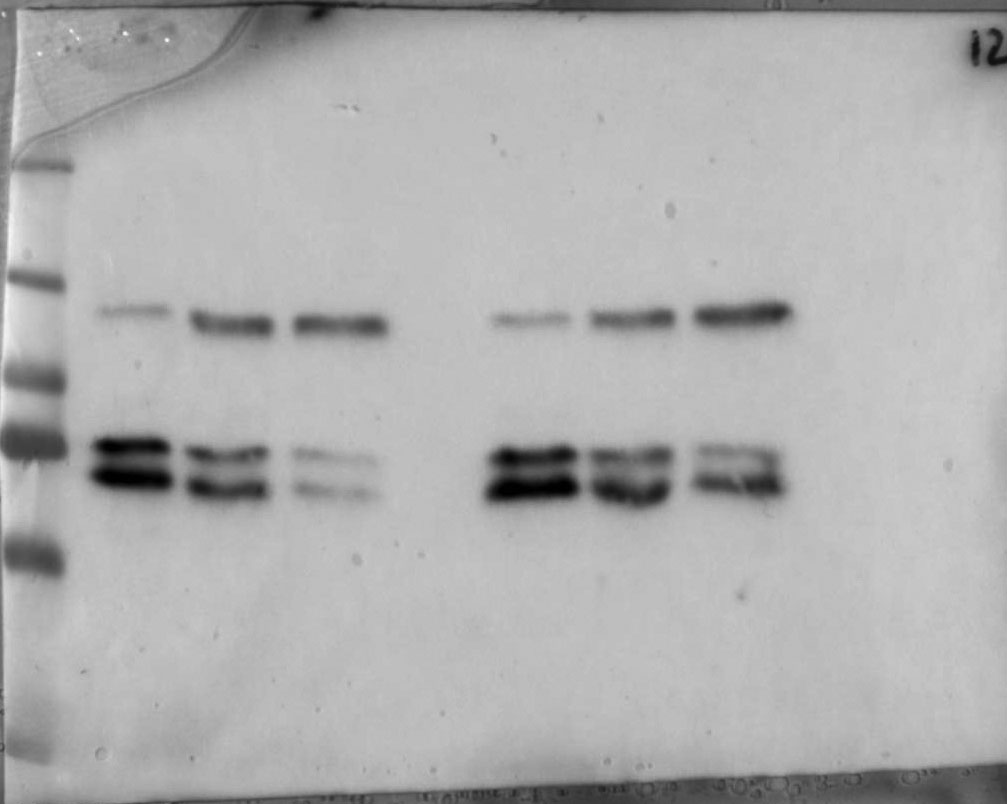

Supplement: Supplementary file 8 — Source Data for Figure 2 [file EMMM-14-e15677-s010.zip › Figure 2/Fig 2A (AhR).jpg]

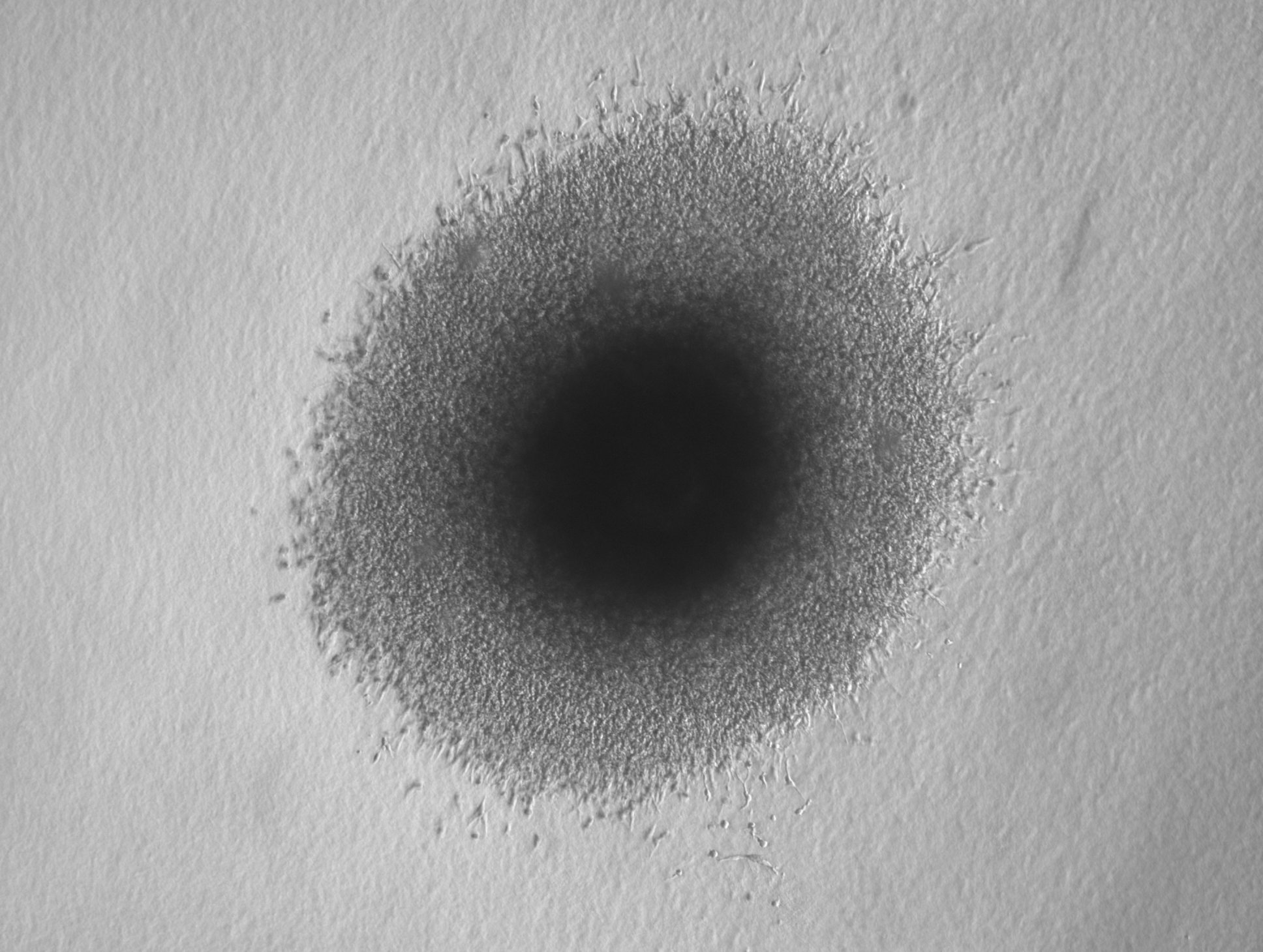

Supplement: Supplementary file 8 — Source Data for Figure 2 [file EMMM-14-e15677-s010.zip › Figure 2/Fig 2C-501sg#2 (D7).jpg]

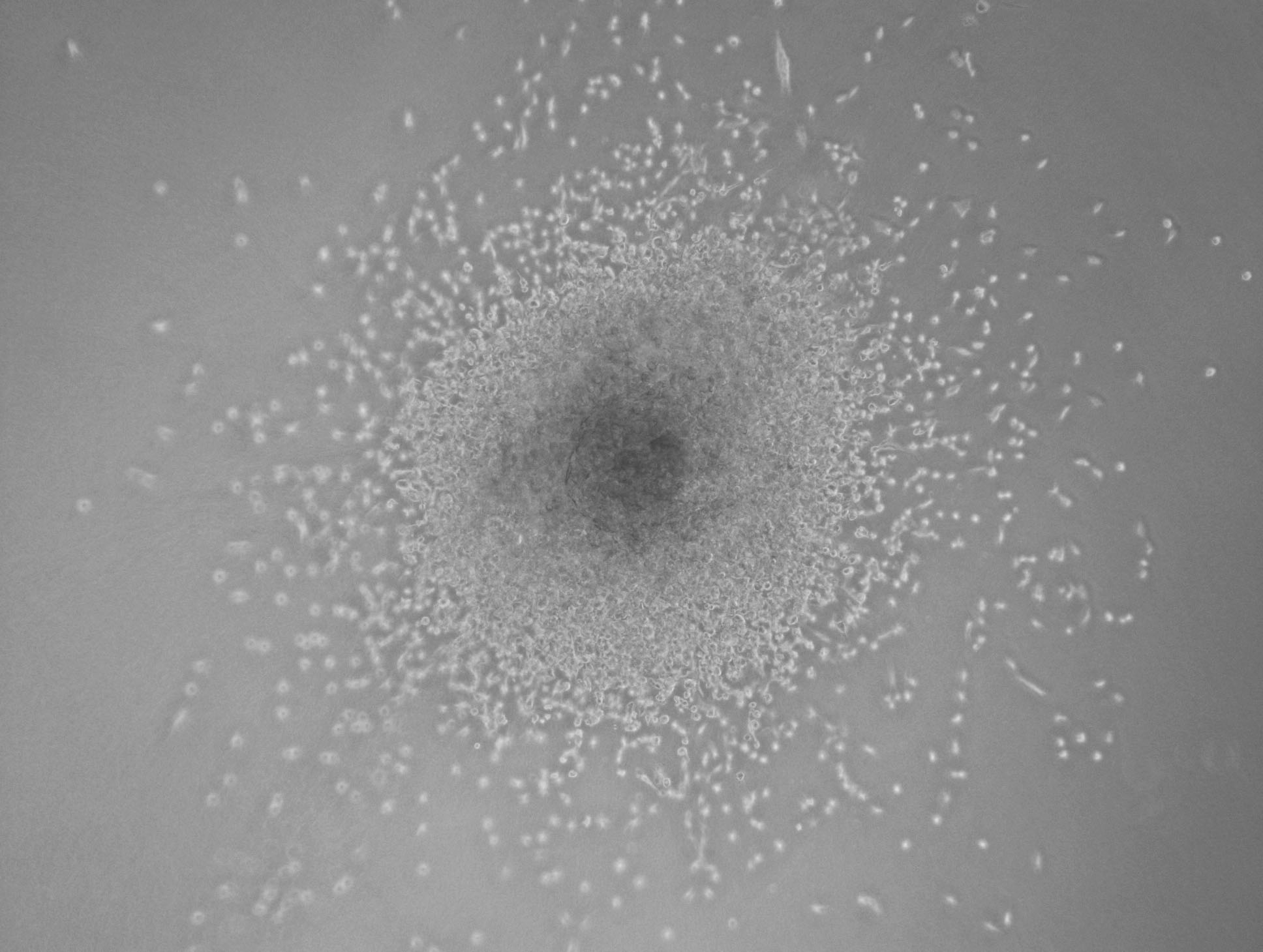

Supplement: Supplementary file 8 — Source Data for Figure 2 [file EMMM-14-e15677-s010.zip › Figure 2/Fig 2F-SK28R (D4).jpg]

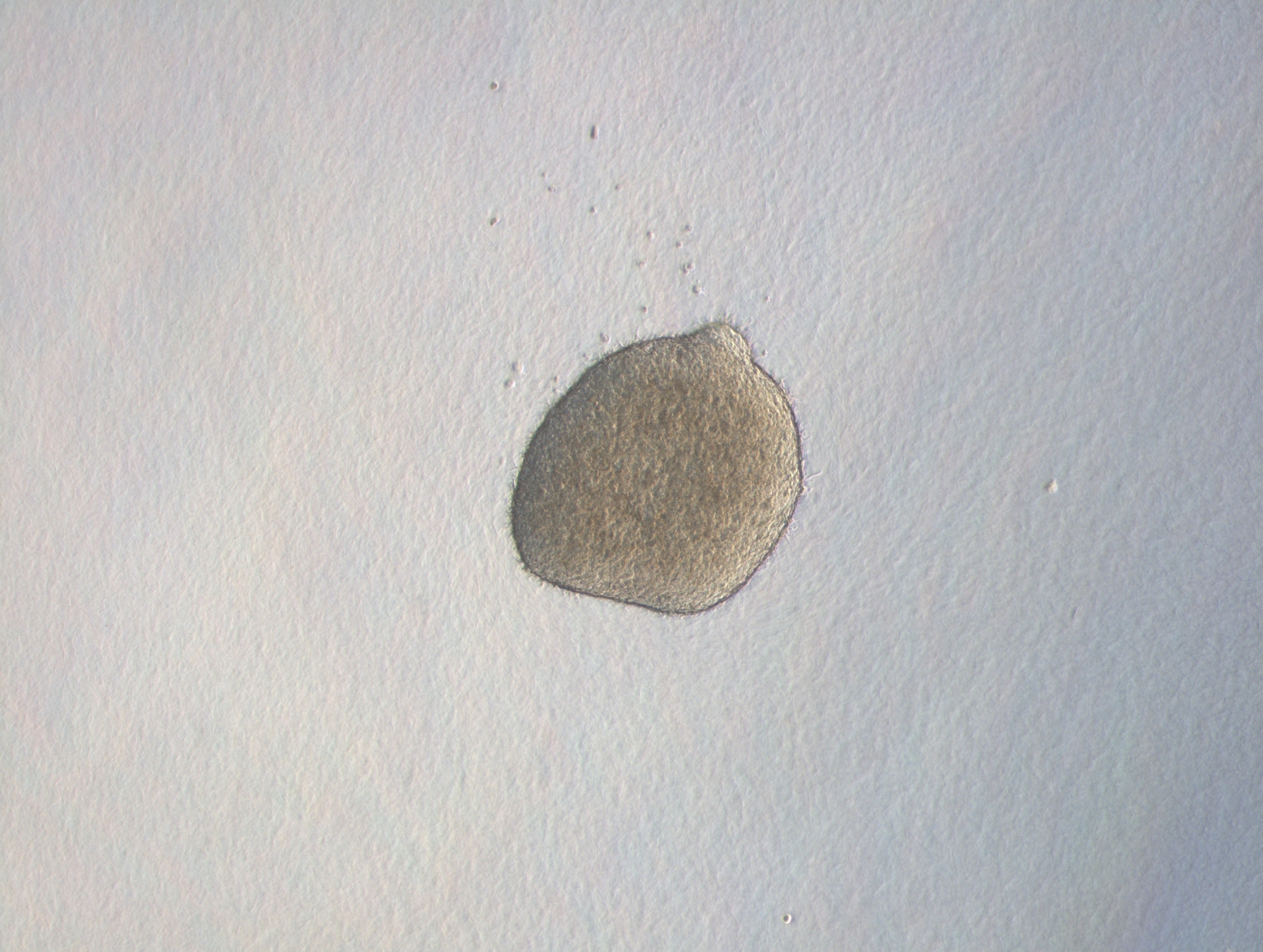

Supplement: Supplementary file 8 — Source Data for Figure 2 [file EMMM-14-e15677-s010.zip › Figure 2/Fig 2C-501sg#2 (D0).jpg]

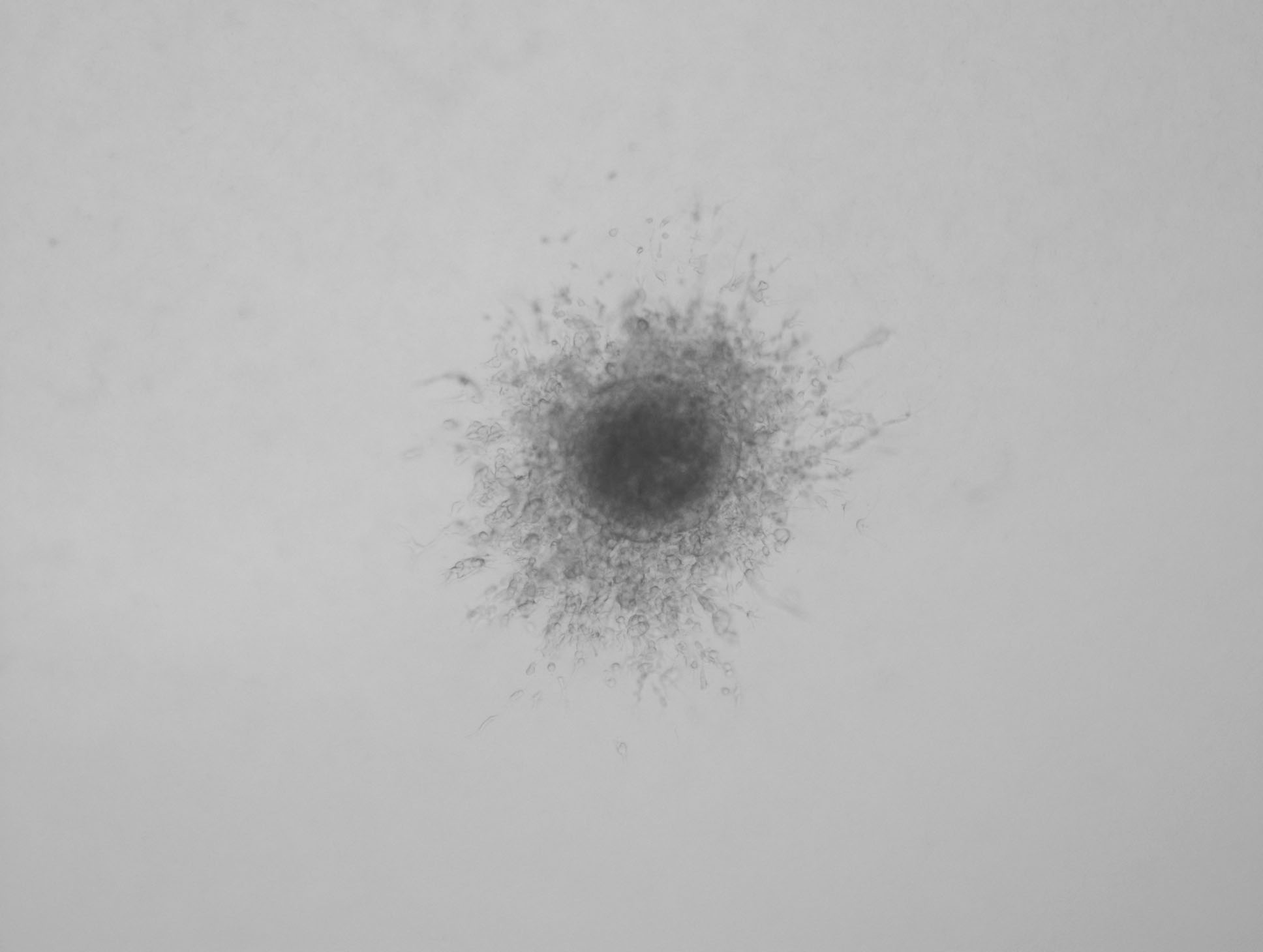

Supplement: Supplementary file 8 — Source Data for Figure 2 [file EMMM-14-e15677-s010.zip › Figure 2/Fig 2F-SK28RKO (D4).jpg]

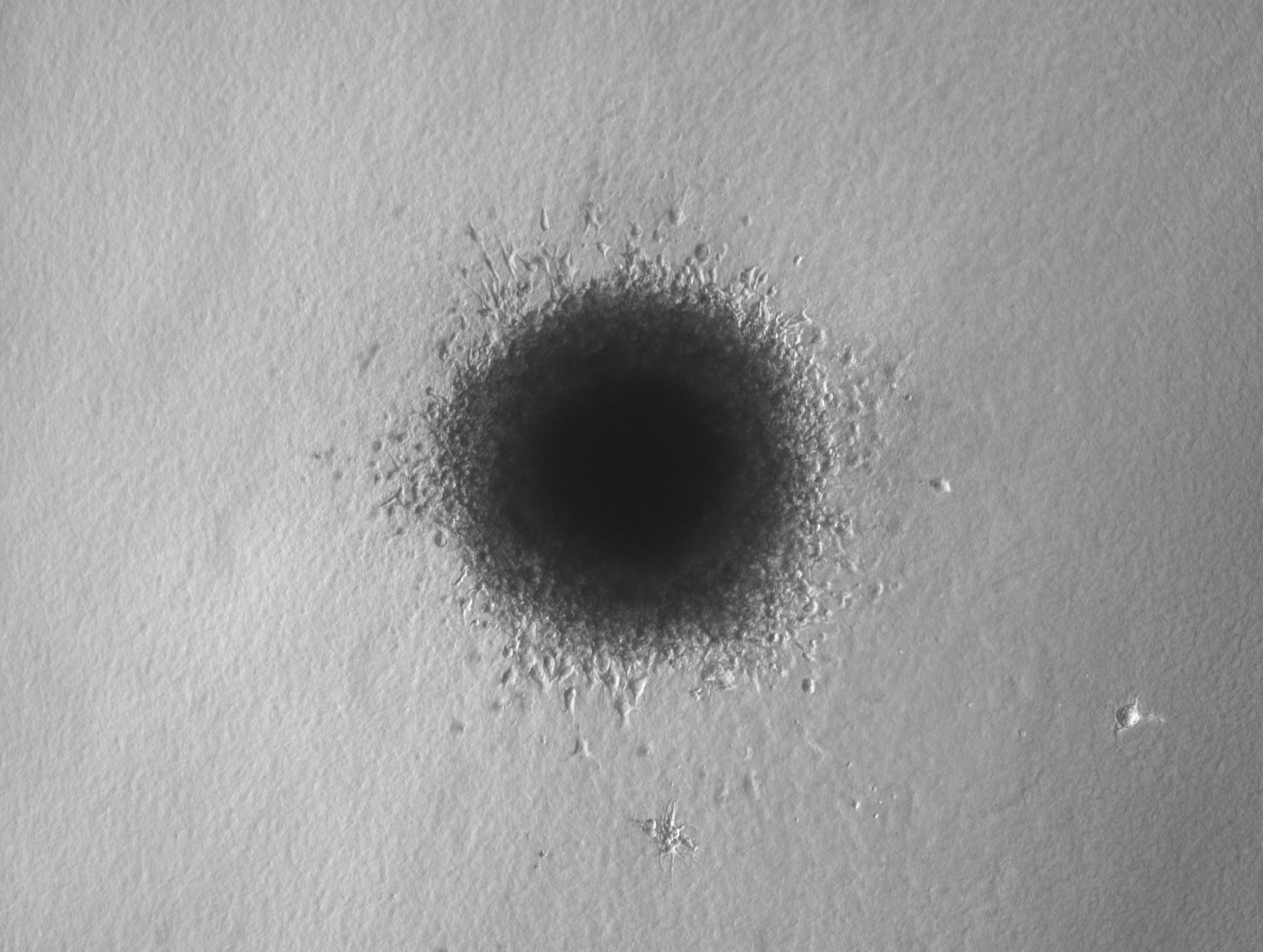

Supplement: Supplementary file 8 — Source Data for Figure 2 [file EMMM-14-e15677-s010.zip › Figure 2/Fig 2C-501CTR (D7).jpg]

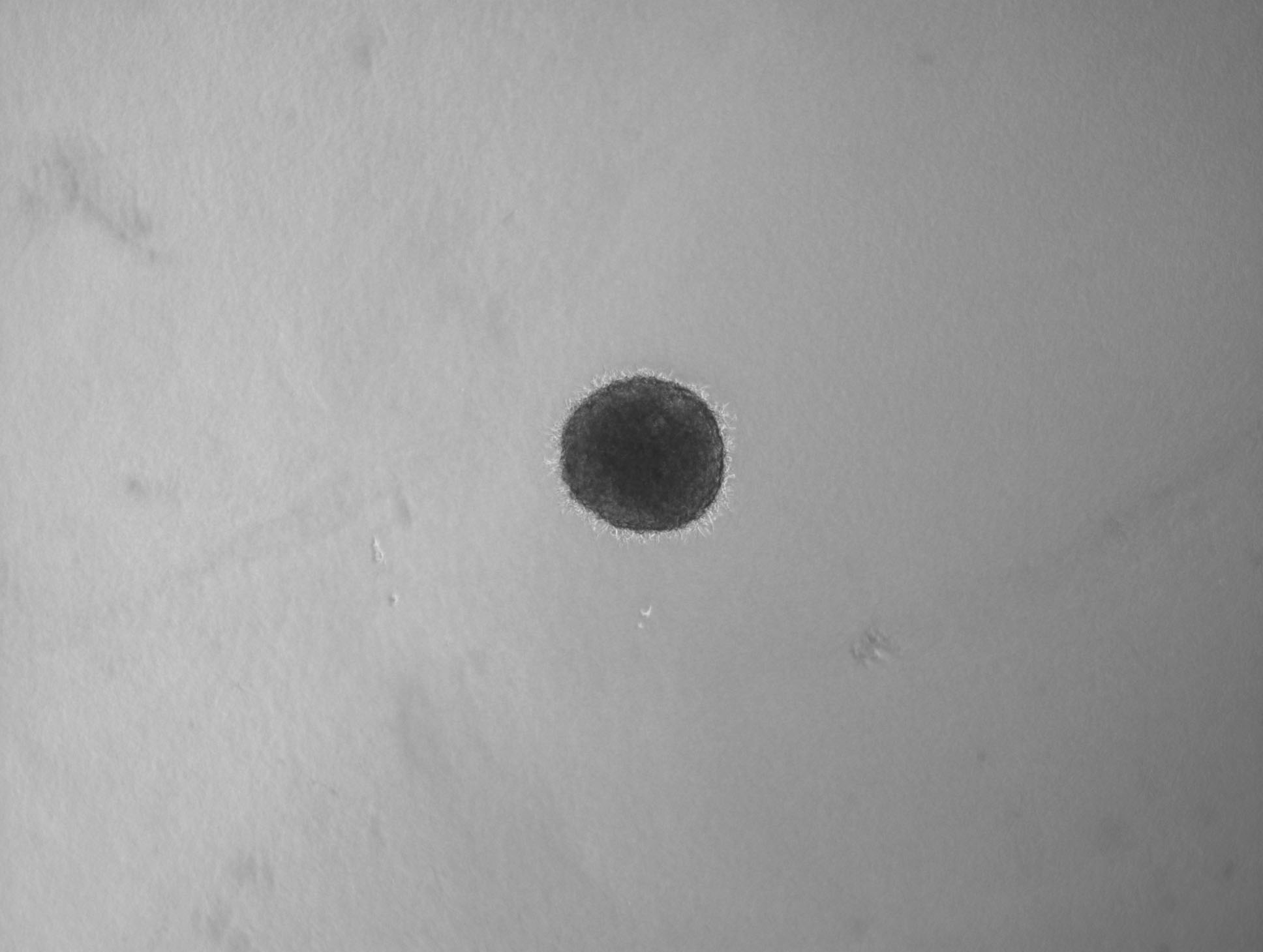

Supplement: Supplementary file 8 — Source Data for Figure 2 [file EMMM-14-e15677-s010.zip › Figure 2/Fig 2F-SK28RKO CA-AhR (D0).jpg]

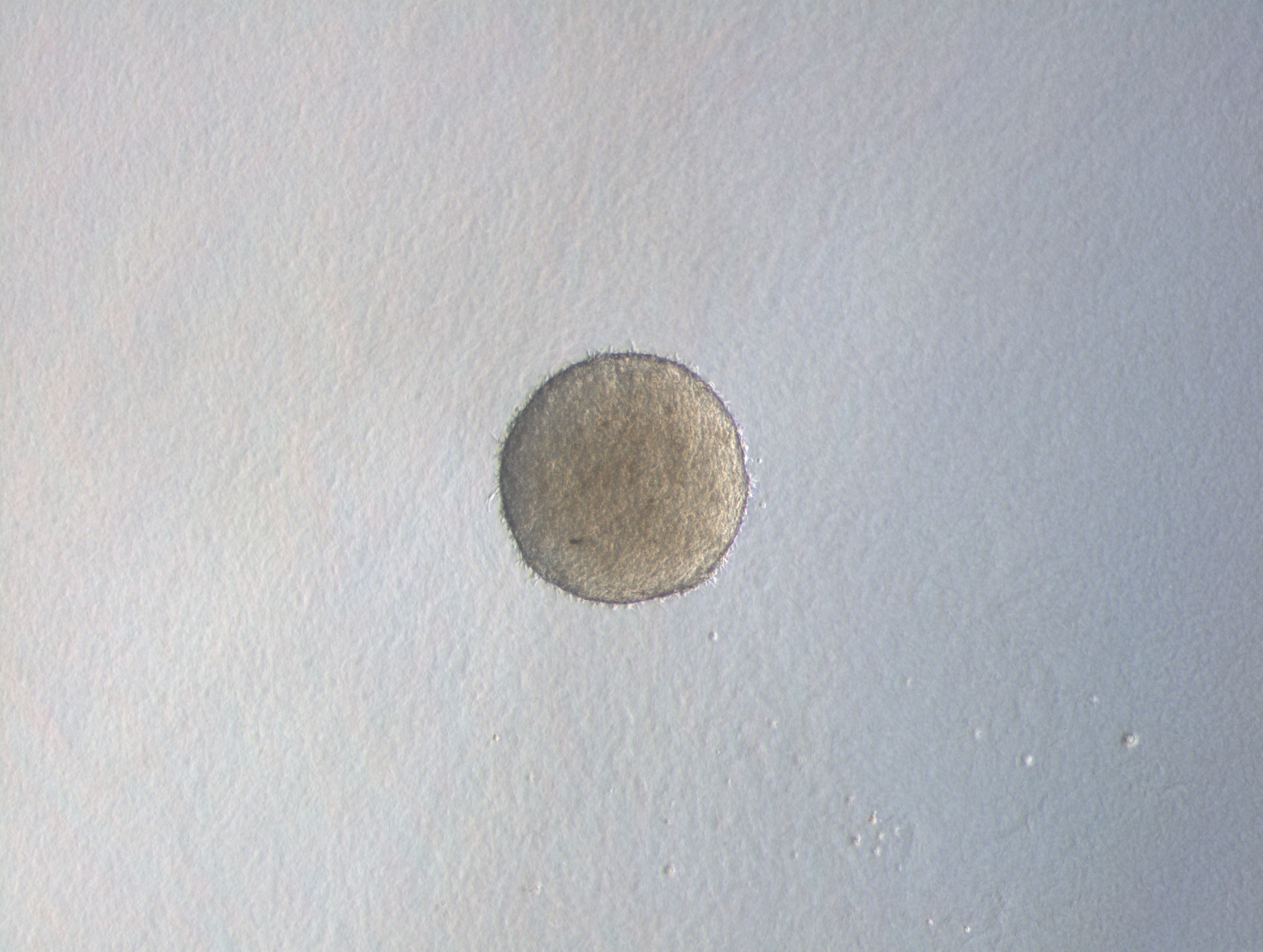

Supplement: Supplementary file 8 — Source Data for Figure 2 [file EMMM-14-e15677-s010.zip › Figure 2/Fig 2C-501CTR (DO).jpg]

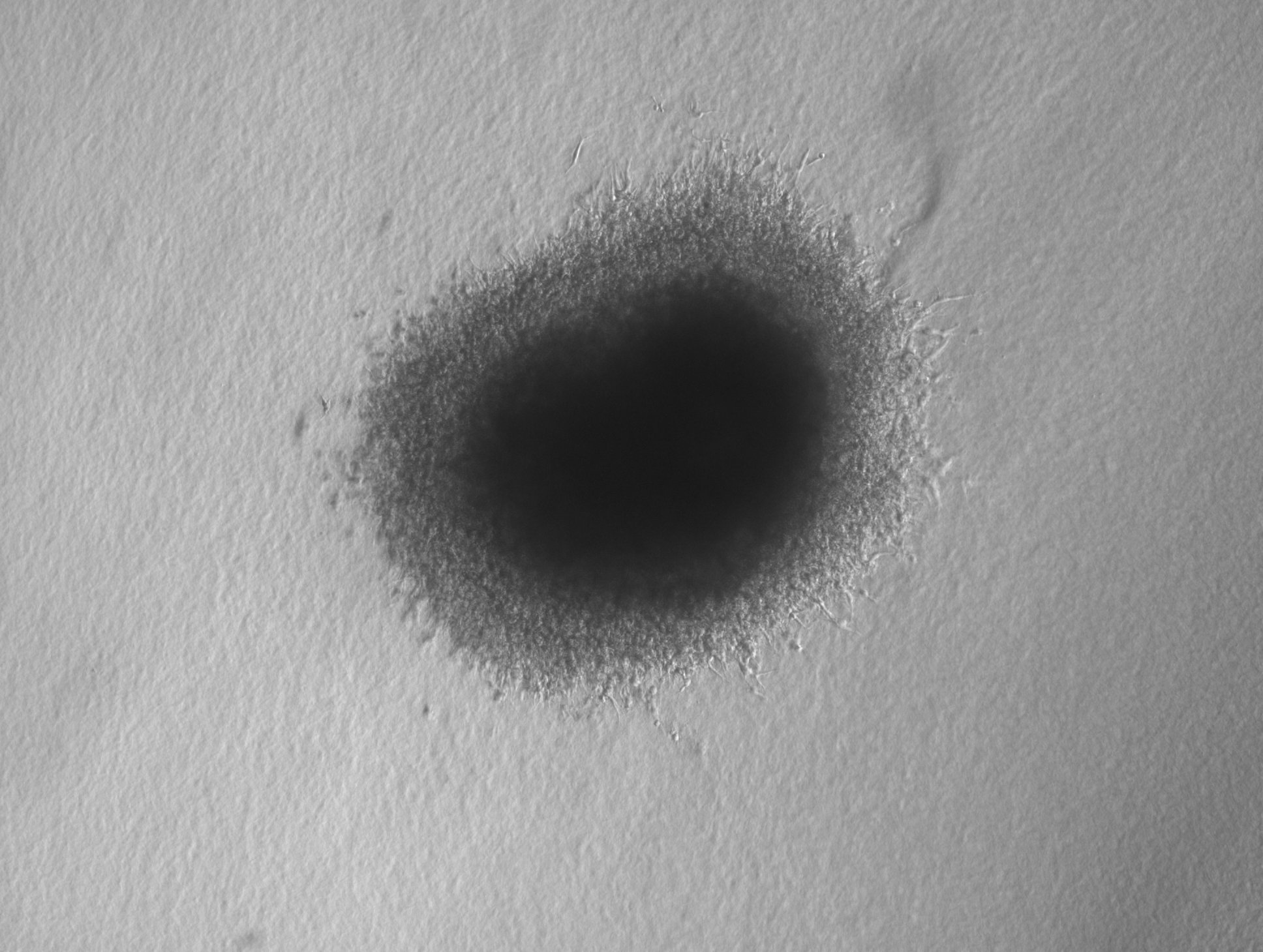

Supplement: Supplementary file 8 — Source Data for Figure 2 [file EMMM-14-e15677-s010.zip › Figure 2/Fig 2C-501sg#1 (D7).jpg]

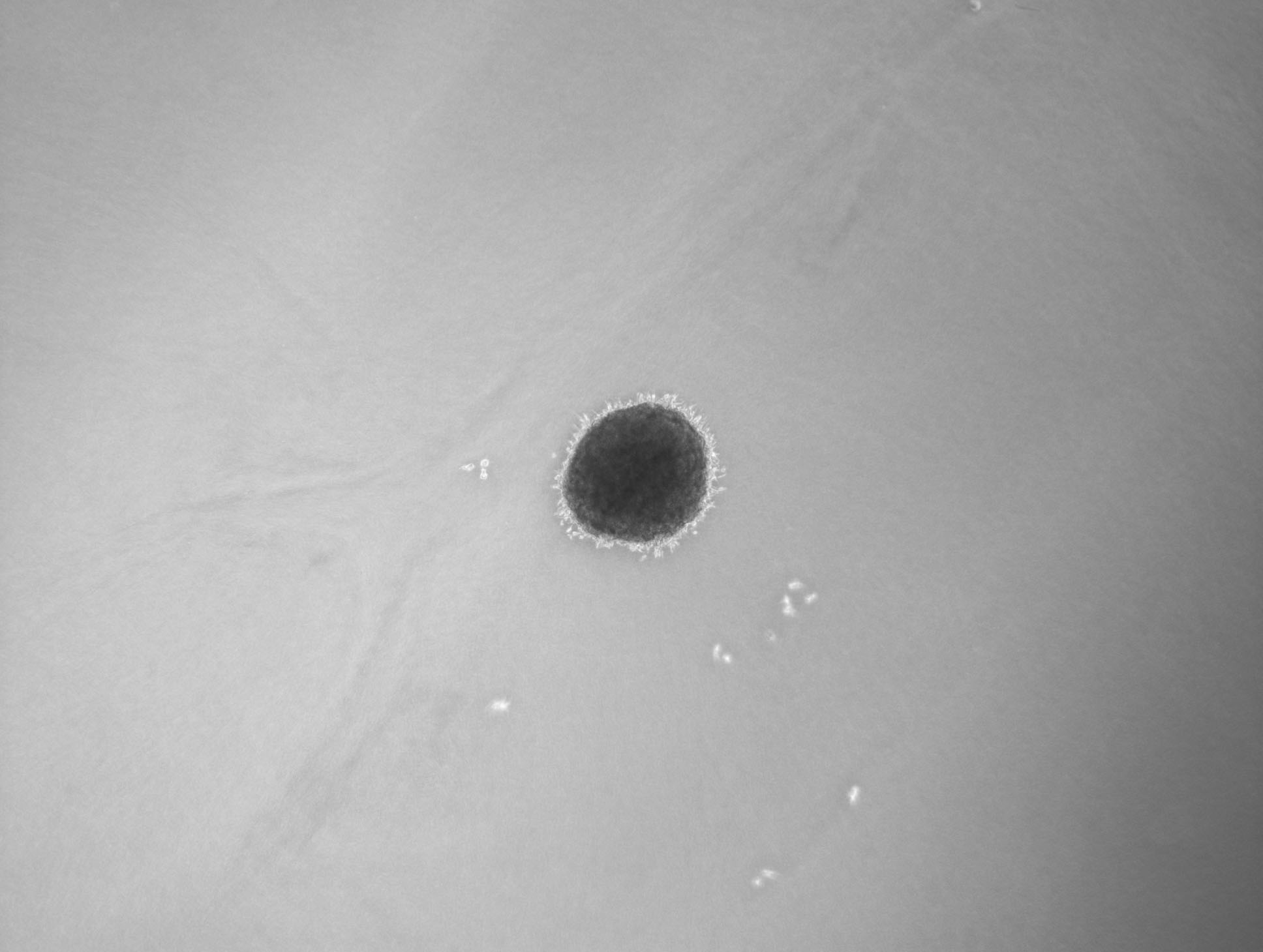

Supplement: Supplementary file 8 — Source Data for Figure 2 [file EMMM-14-e15677-s010.zip › Figure 2/Fig 2F-SK28R (D0).jpg]

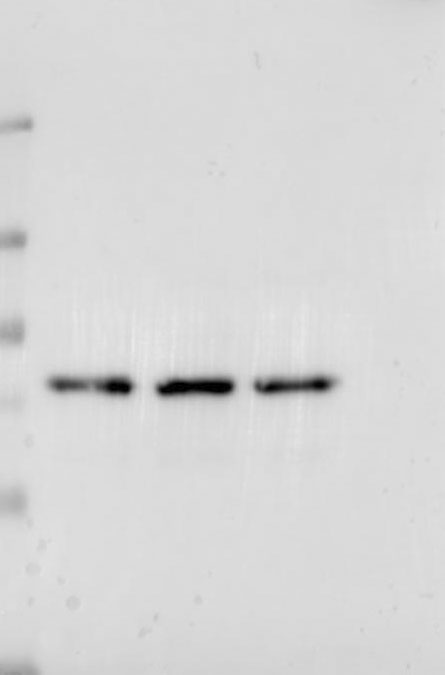

Supplement: Supplementary file 8 — Source Data for Figure 2 [file EMMM-14-e15677-s010.zip › Figure 2/Fig 2D (HSC70).jpg]

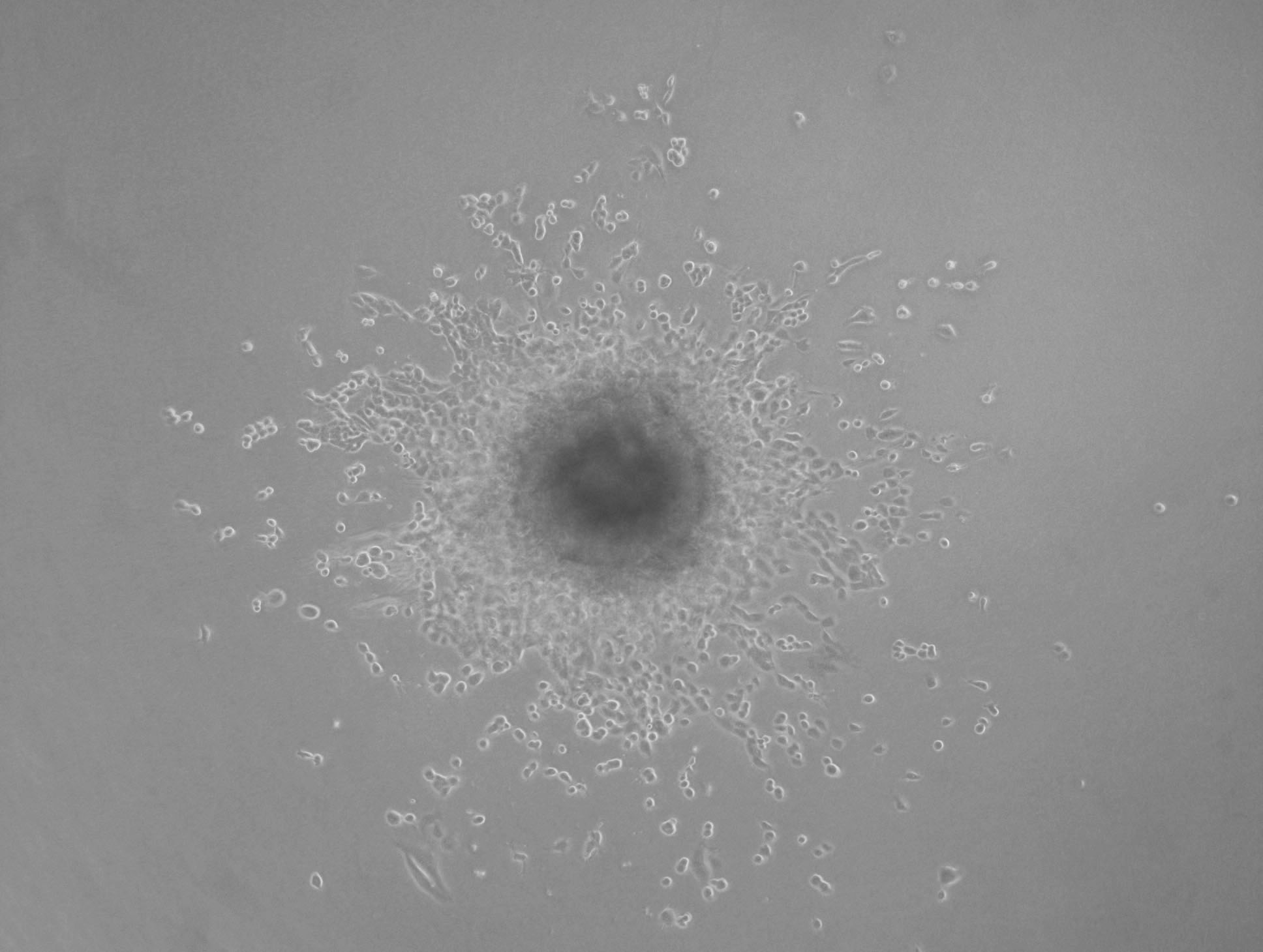

Supplement: Supplementary file 8 — Source Data for Figure 2 [file EMMM-14-e15677-s010.zip › Figure 2/Fig 2F-SK28RKO CA-AhR (D4).jpg]

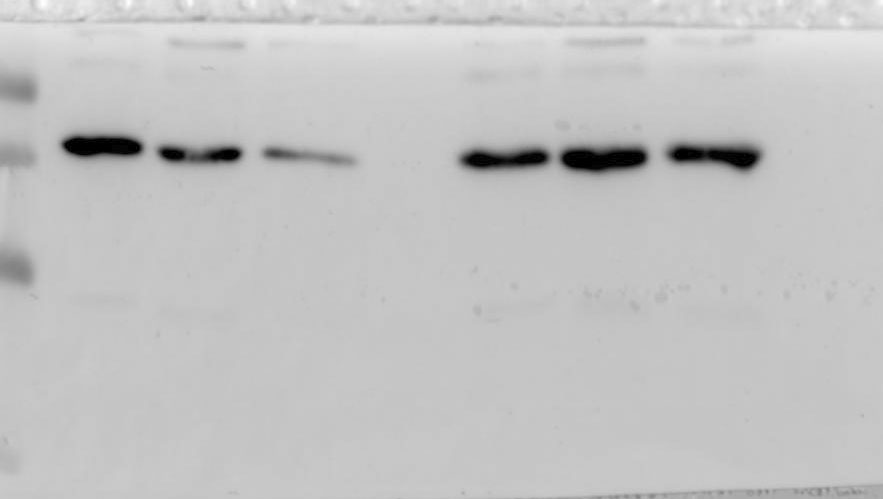

Supplement: Supplementary file 8 — Source Data for Figure 2 [file EMMM-14-e15677-s010.zip › Figure 2/Fig 2A (HSC70).jpg]

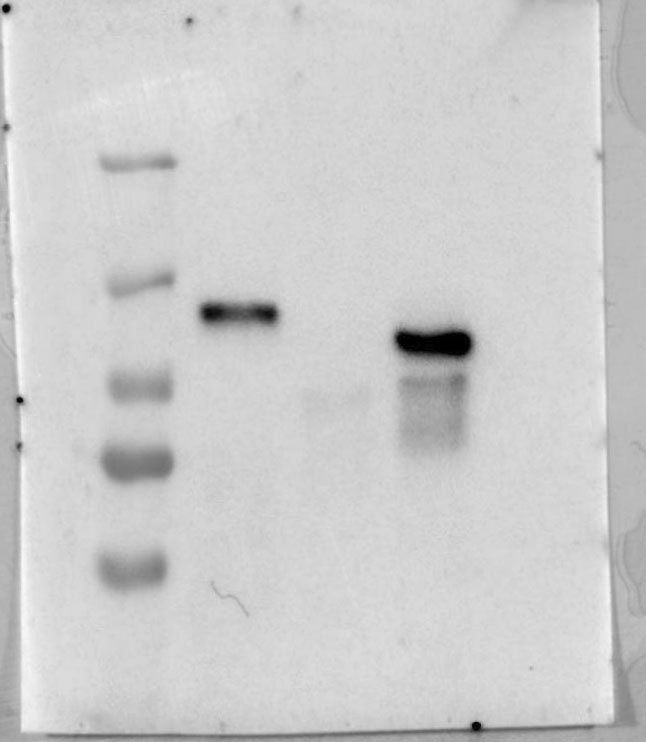

Supplement: Supplementary file 8 — Source Data for Figure 2 [file EMMM-14-e15677-s010.zip › Figure 2/Fig 2D (AhR).jpg]

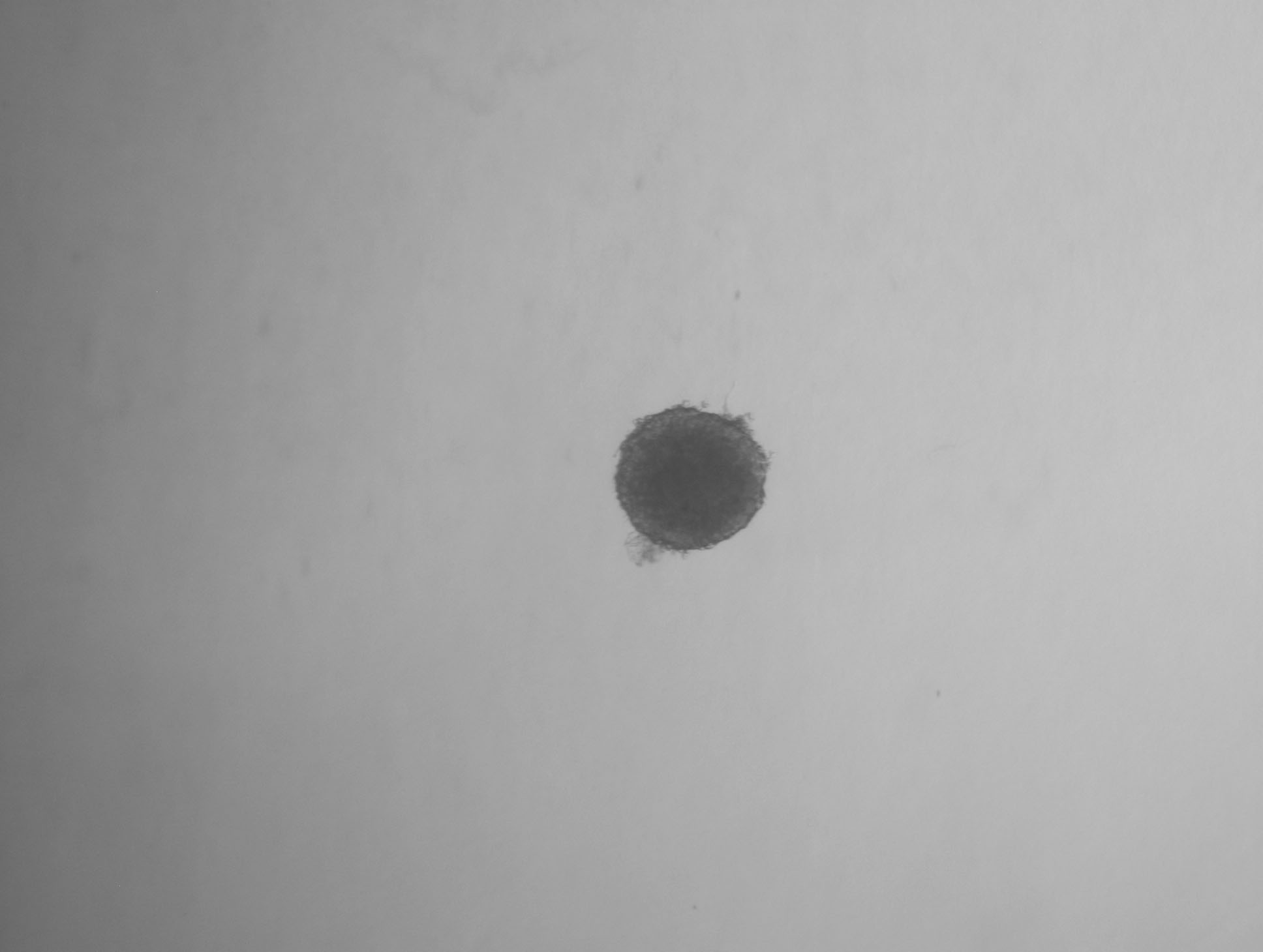

Supplement: Supplementary file 8 — Source Data for Figure 2 [file EMMM-14-e15677-s010.zip › Figure 2/Fig 2F-SK28RKO (D0).jpg]

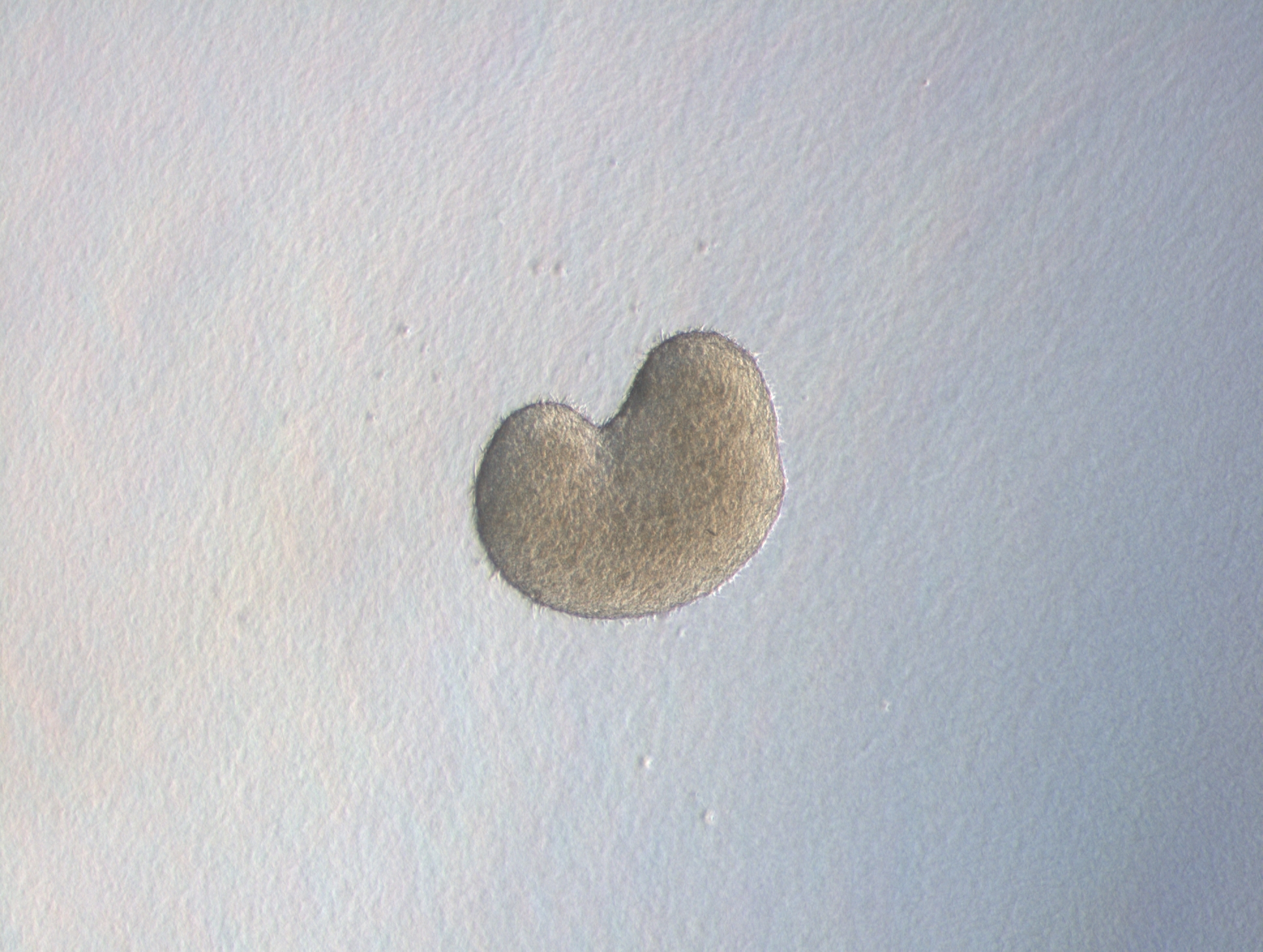

Supplement: Supplementary file 8 — Source Data for Figure 2 [file EMMM-14-e15677-s010.zip › Figure 2/Fig 2C-501sg#1 (D0).jpg]

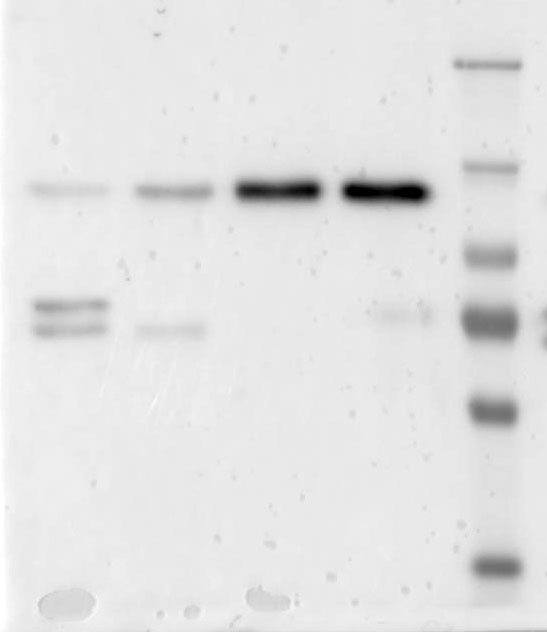

Supplement: Supplementary file 9 — Source Data for Figure 4 [file EMMM-14-e15677-s008.zip › Figure 4/Fig 4B (AhR).jpg]

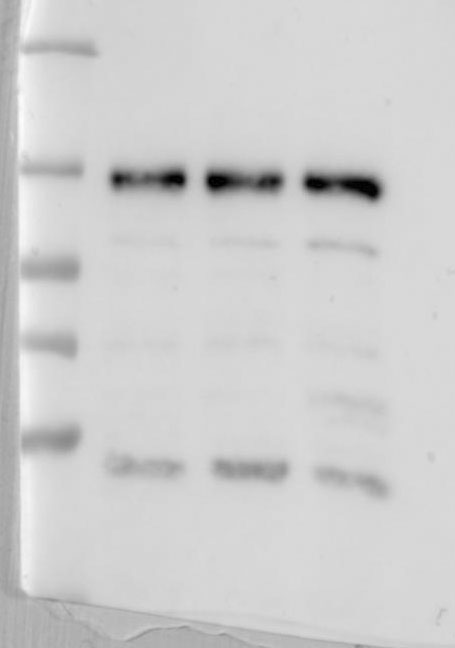

Supplement: Supplementary file 9 — Source Data for Figure 4 [file EMMM-14-e15677-s008.zip › Figure 4/Fig 4C (FAK).jpg]

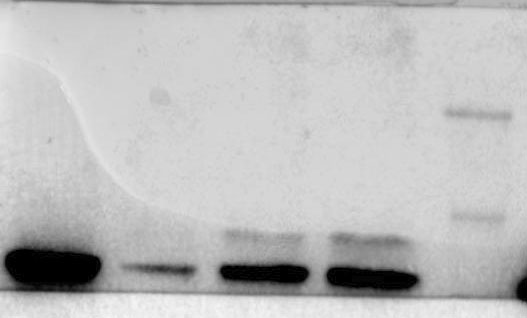

Supplement: Supplementary file 9 — Source Data for Figure 4 [file EMMM-14-e15677-s008.zip › Figure 4/Fig 4B (P-FAK).jpg]

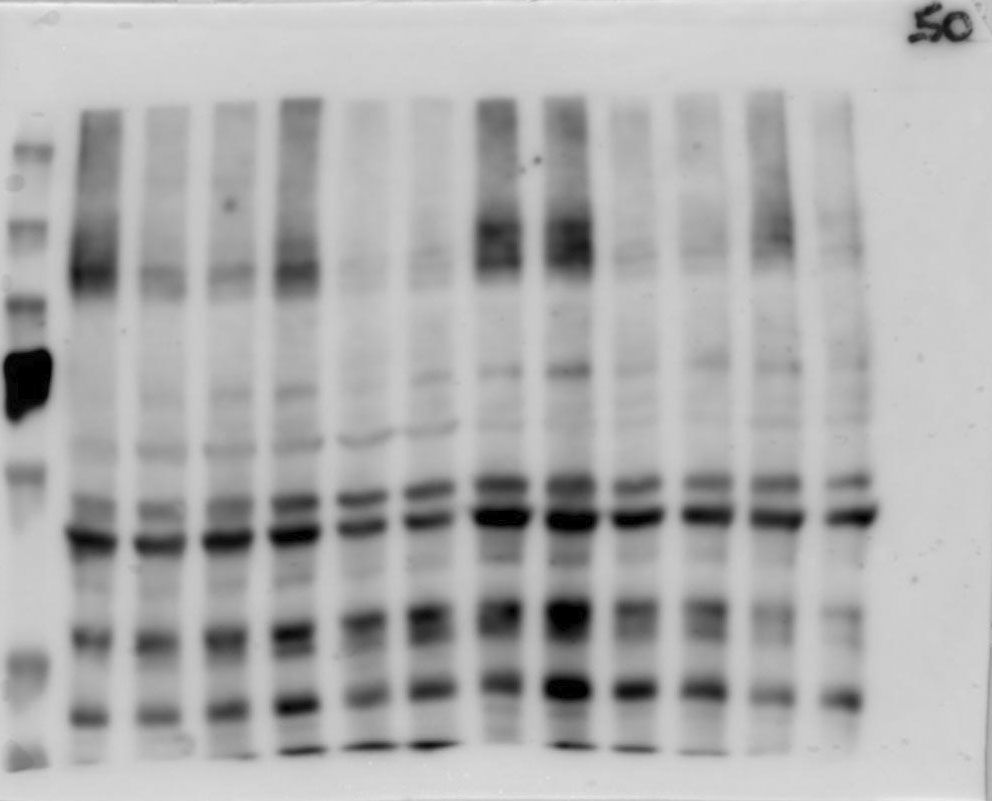

Supplement: Supplementary file 9 — Source Data for Figure 4 [file EMMM-14-e15677-s008.zip › Figure 4/Fig 4D (AhR).jpg]

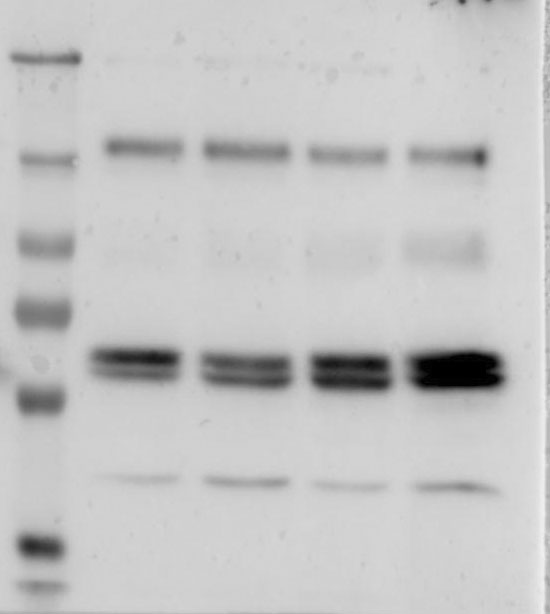

Supplement: Supplementary file 9 — Source Data for Figure 4 [file EMMM-14-e15677-s008.zip › Figure 4/Fig 4E (P-SRC).jpg]

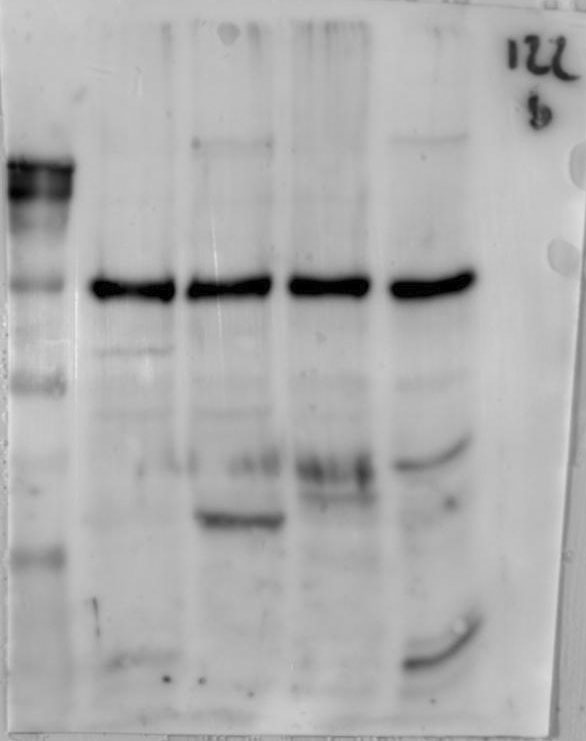

Supplement: Supplementary file 9 — Source Data for Figure 4 [file EMMM-14-e15677-s008.zip › Figure 4/Fig 4B (FAK).jpg]

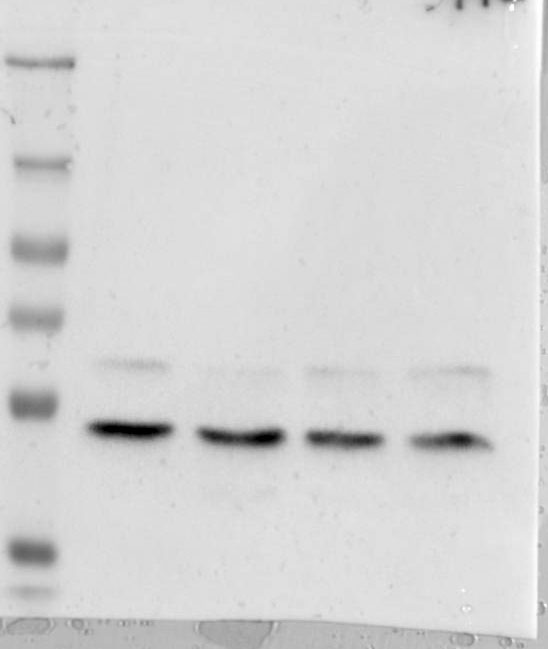

Supplement: Supplementary file 9 — Source Data for Figure 4 [file EMMM-14-e15677-s008.zip › Figure 4/Fig 4E (HSC70).jpg]

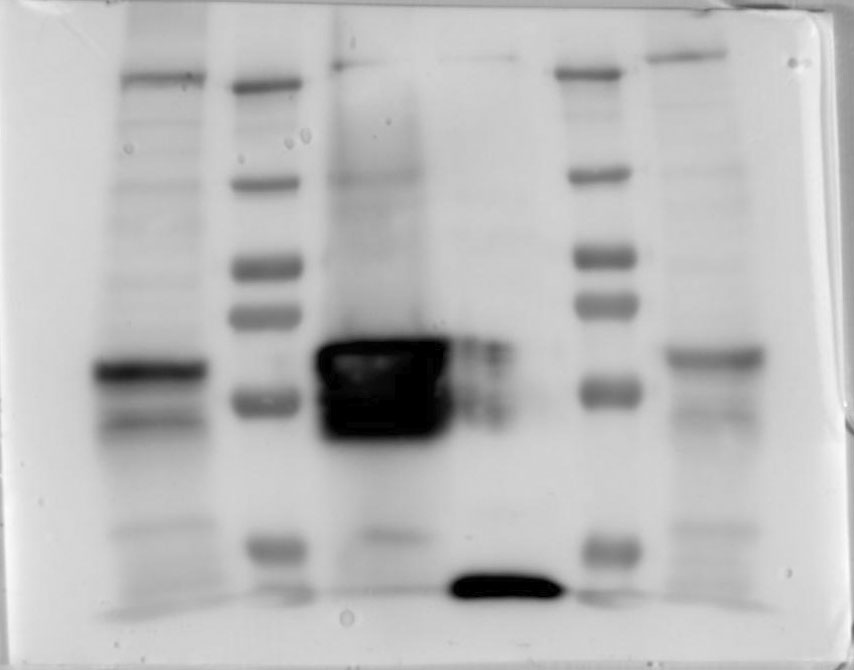

Supplement: Supplementary file 9 — Source Data for Figure 4 [file EMMM-14-e15677-s008.zip › Figure 4/Fig 4A (SRC).jpg]

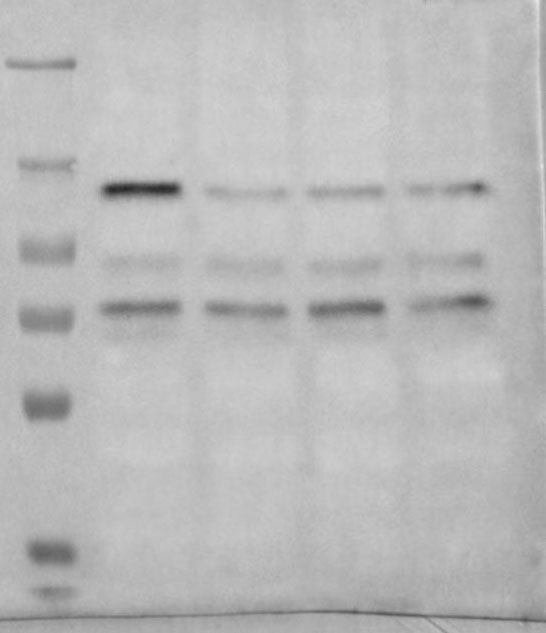

Supplement: Supplementary file 9 — Source Data for Figure 4 [file EMMM-14-e15677-s008.zip › Figure 4/Fig 4E (AhR).jpg]

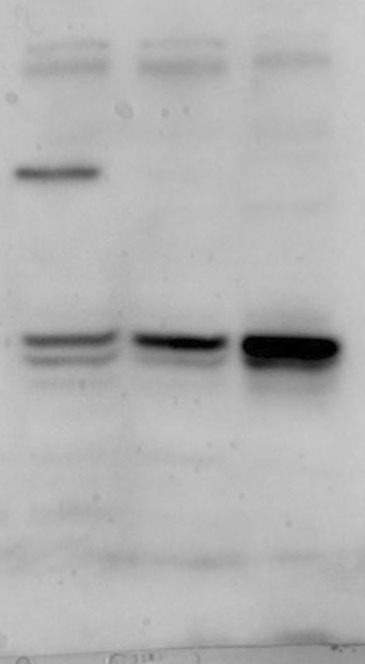

Supplement: Supplementary file 9 — Source Data for Figure 4 [file EMMM-14-e15677-s008.zip › Figure 4/Fig 4C (P-SRC).jpg]

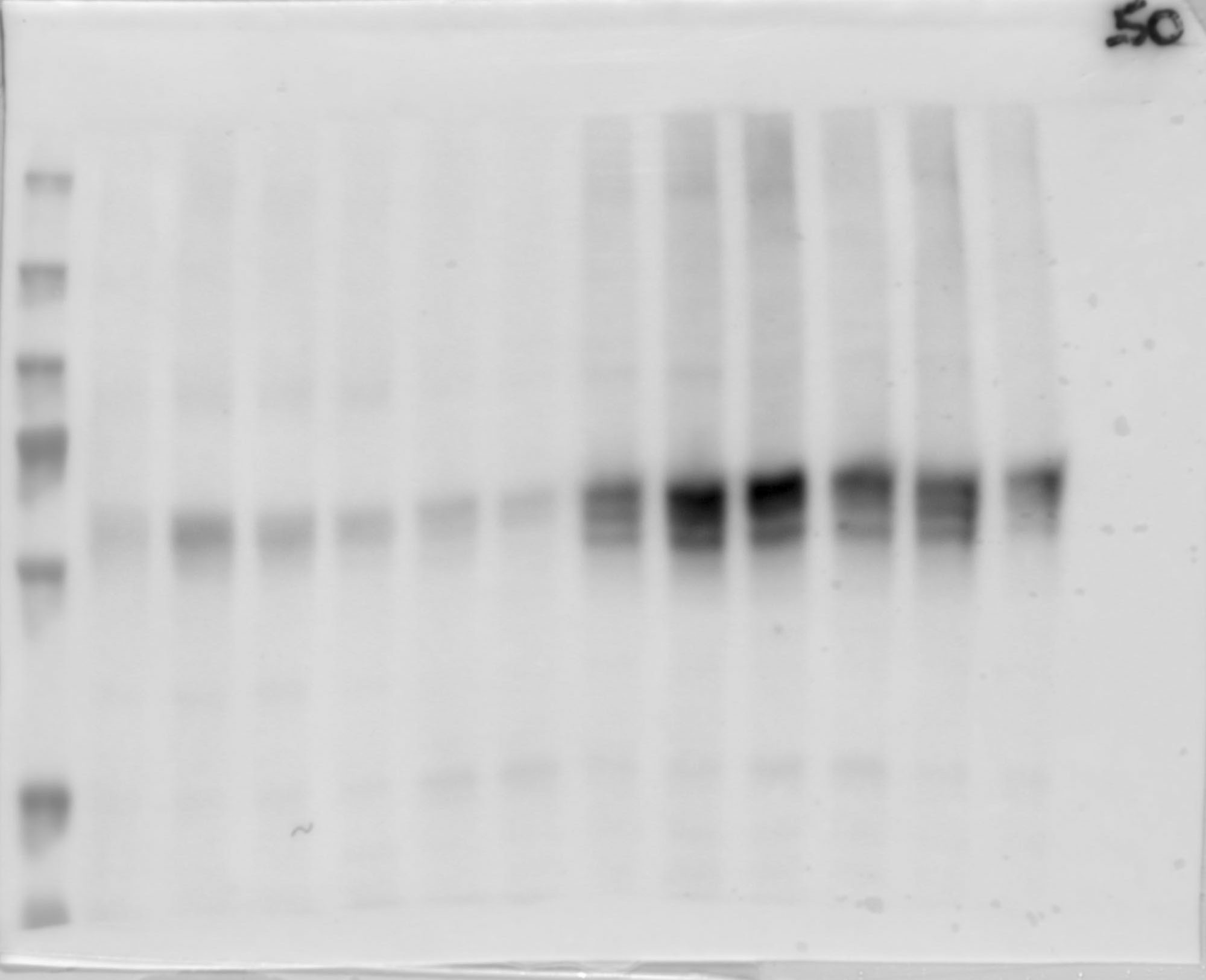

Supplement: Supplementary file 9 — Source Data for Figure 4 [file EMMM-14-e15677-s008.zip › Figure 4/Fig 4D (P-SRC).jpg]

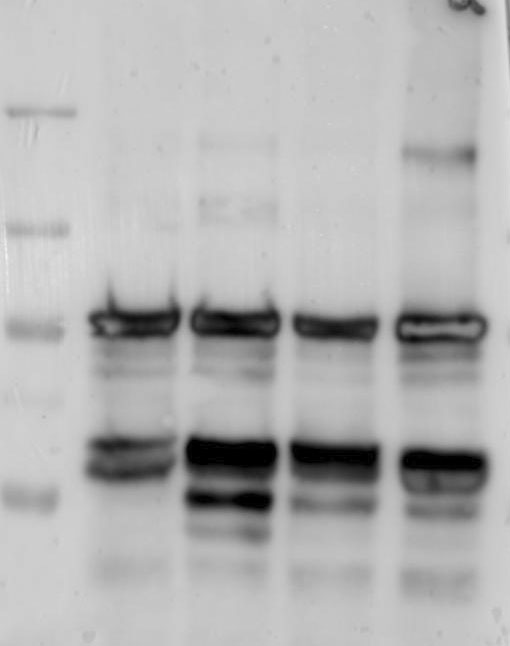

Supplement: Supplementary file 9 — Source Data for Figure 4 [file EMMM-14-e15677-s008.zip › Figure 4/Fig 4B (SRC).jpg]

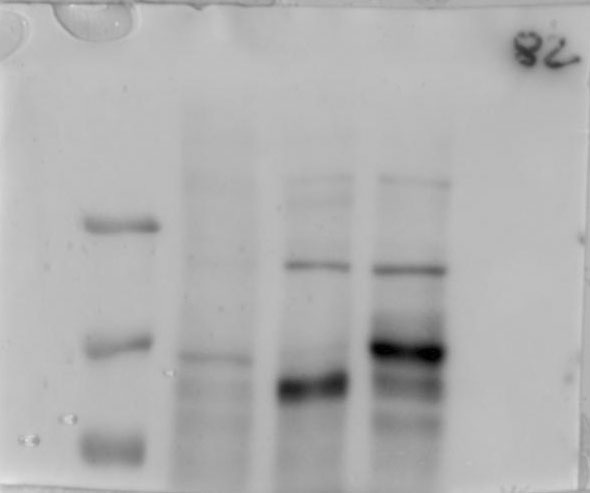

Supplement: Supplementary file 9 — Source Data for Figure 4 [file EMMM-14-e15677-s008.zip › Figure 4/Fig 4C (P-FAK).jpg]

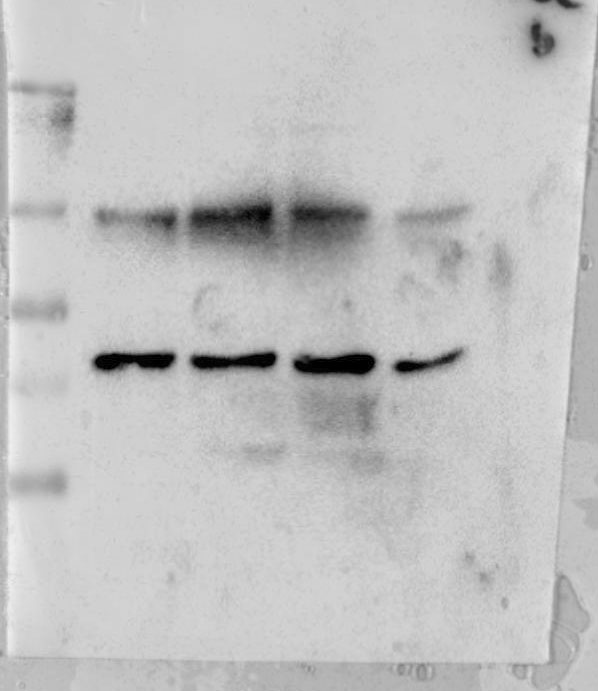

Supplement: Supplementary file 9 — Source Data for Figure 4 [file EMMM-14-e15677-s008.zip › Figure 4/Fig 4B (HSC70).jpg]

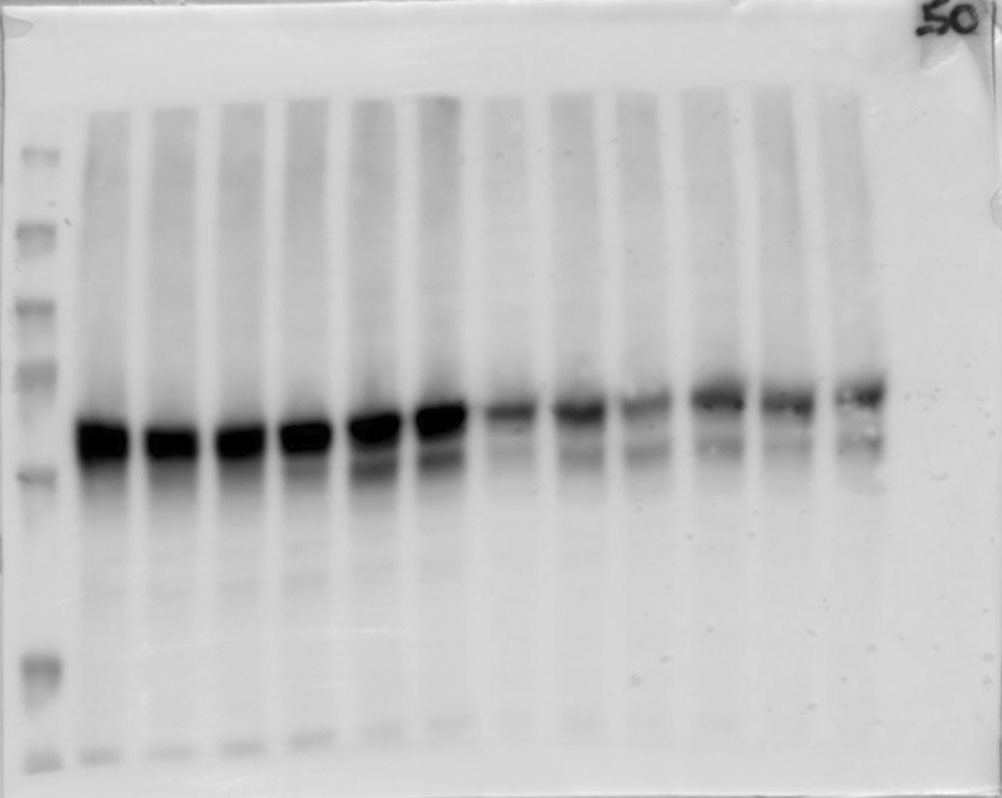

Supplement: Supplementary file 9 — Source Data for Figure 4 [file EMMM-14-e15677-s008.zip › Figure 4/Fig 4D (SRC).jpg]

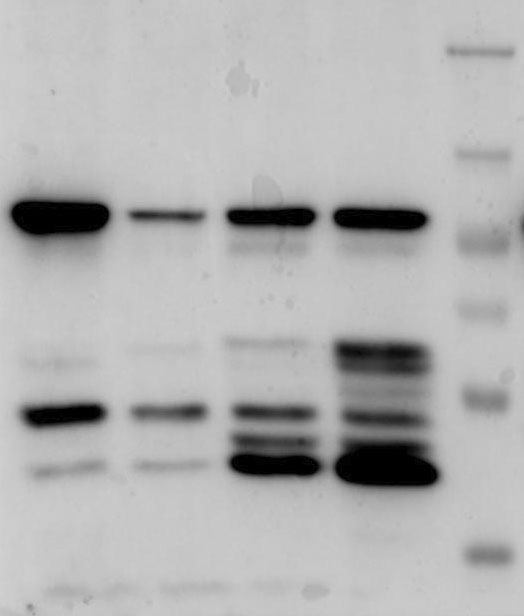

Supplement: Supplementary file 9 — Source Data for Figure 4 [file EMMM-14-e15677-s008.zip › Figure 4/Fig 4B (P-SRC).jpg]

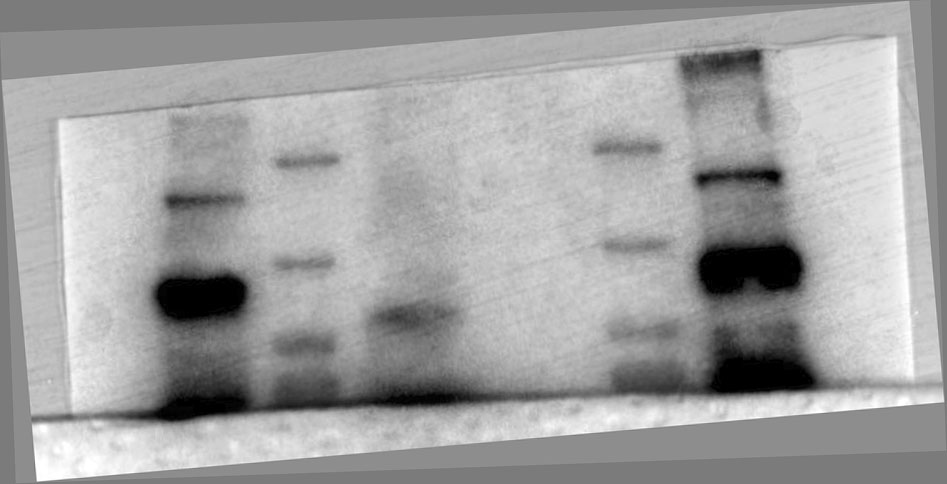

Supplement: Supplementary file 9 — Source Data for Figure 4 [file EMMM-14-e15677-s008.zip › Figure 4/Fig 4A (AhR).jpg]

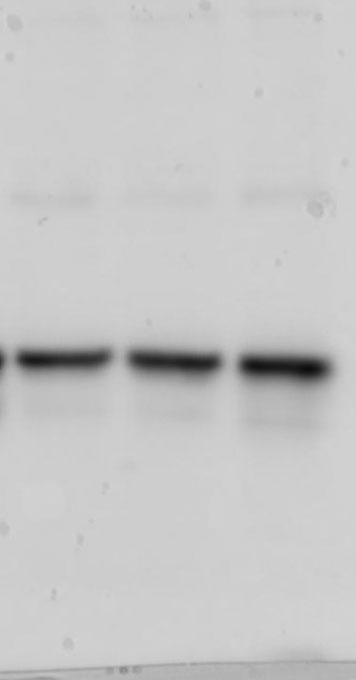

Supplement: Supplementary file 9 — Source Data for Figure 4 [file EMMM-14-e15677-s008.zip › Figure 4/Fig 4C (SRC).jpg]

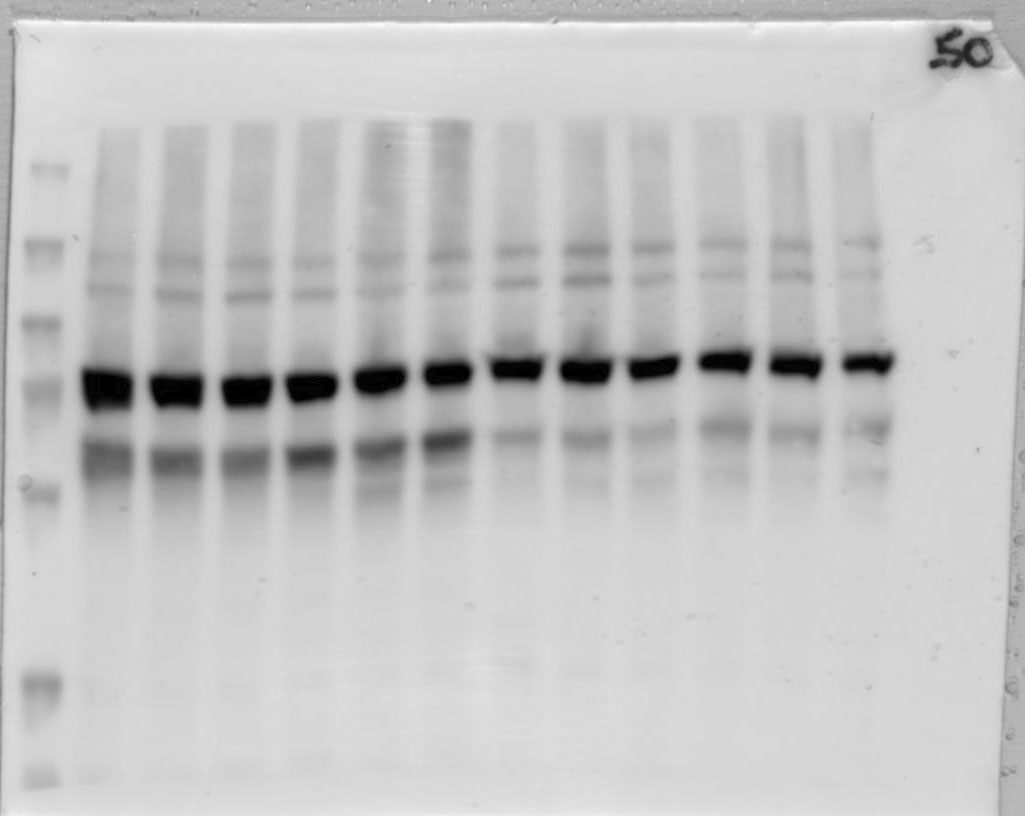

Supplement: Supplementary file 9 — Source Data for Figure 4 [file EMMM-14-e15677-s008.zip › Figure 4/Fig 4D (HSC70).jpg]

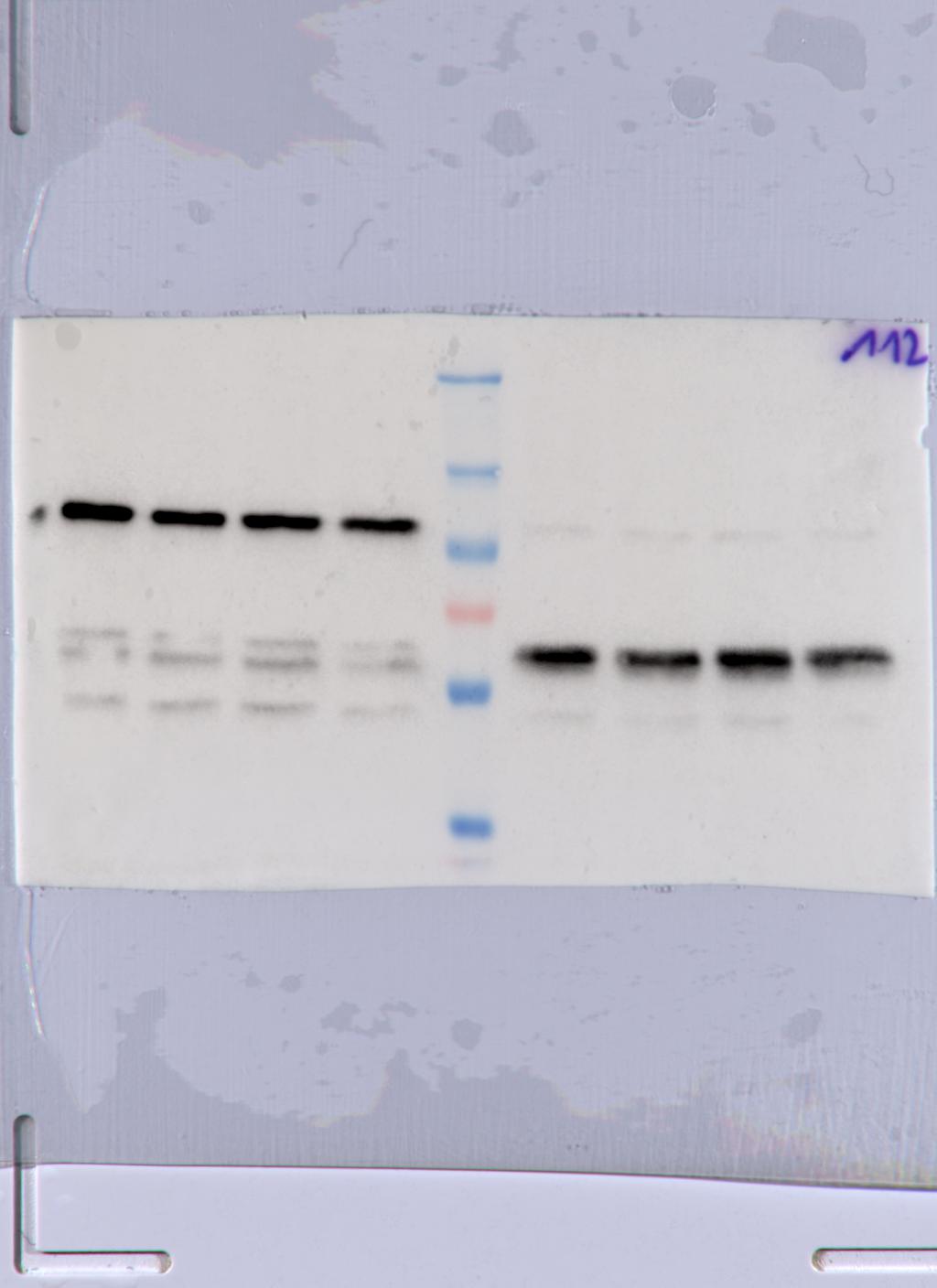

Supplement: Supplementary file 9 — Source Data for Figure 4 [file EMMM-14-e15677-s008.zip › Figure 4/Fig 4E (SRC).jpg]

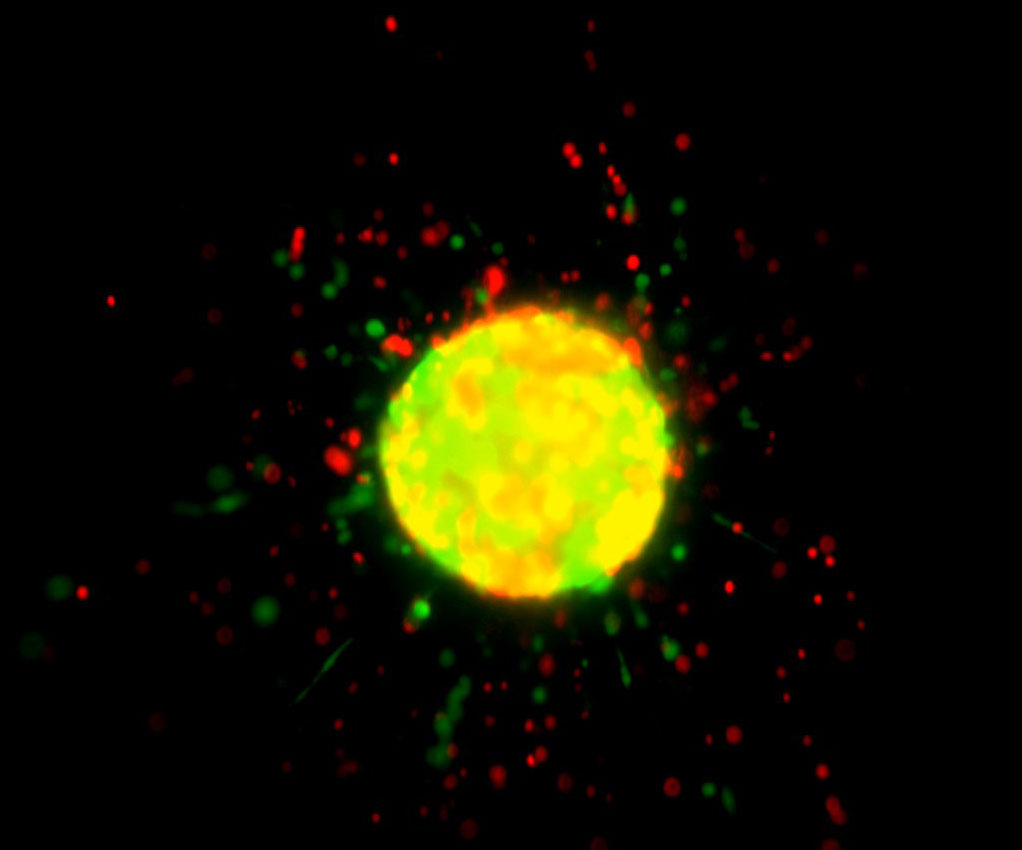

Supplement: Supplementary file 10 — Source Data for Figure 5 [file EMMM-14-e15677-s002.zip › Figure 5/Fig 5B (Dasa, D5.jpg]

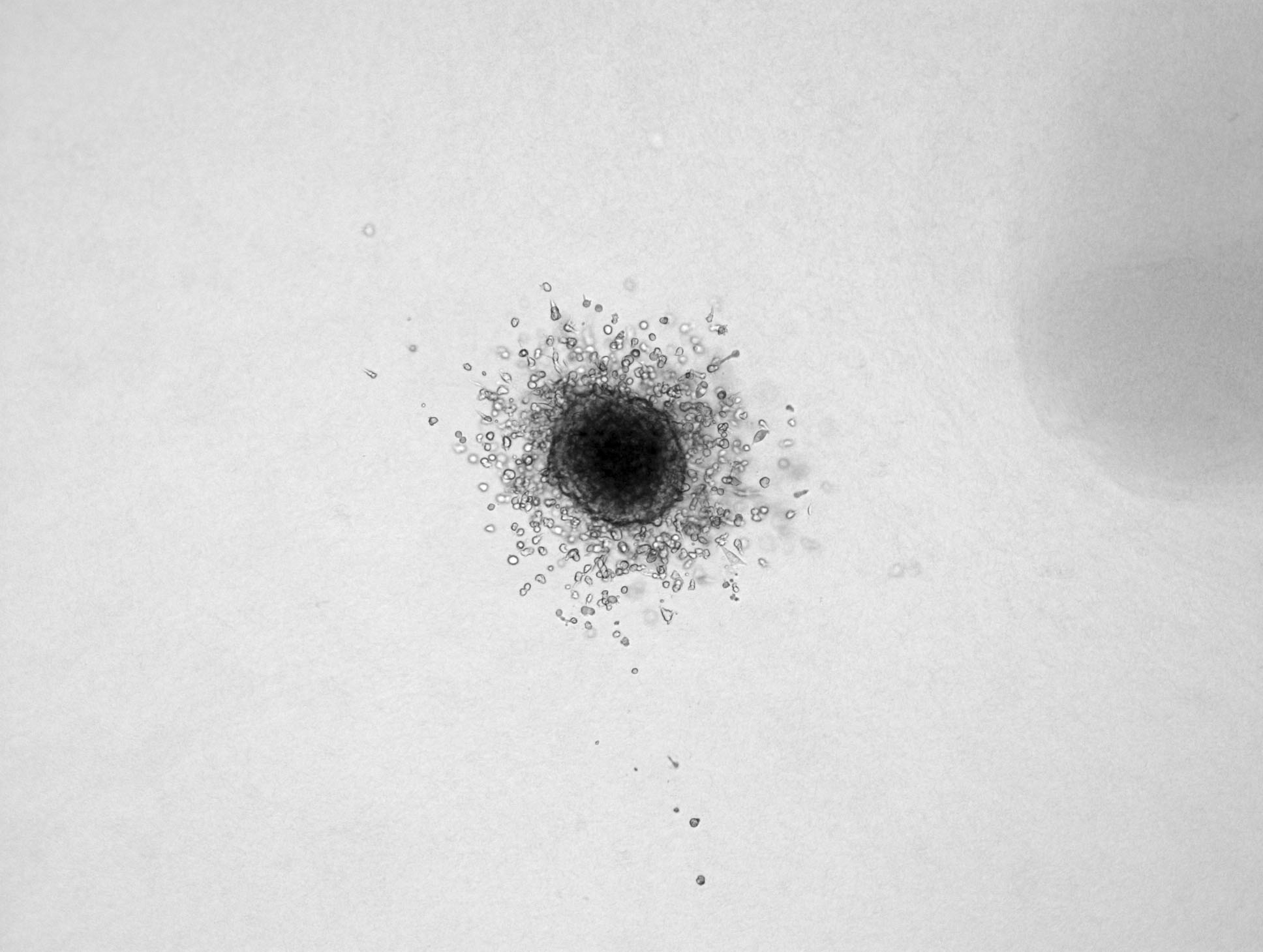

Supplement: Supplementary file 10 — Source Data for Figure 5 [file EMMM-14-e15677-s002.zip › Figure 5/Fig 5C Das (DMSO, D5).jpg]

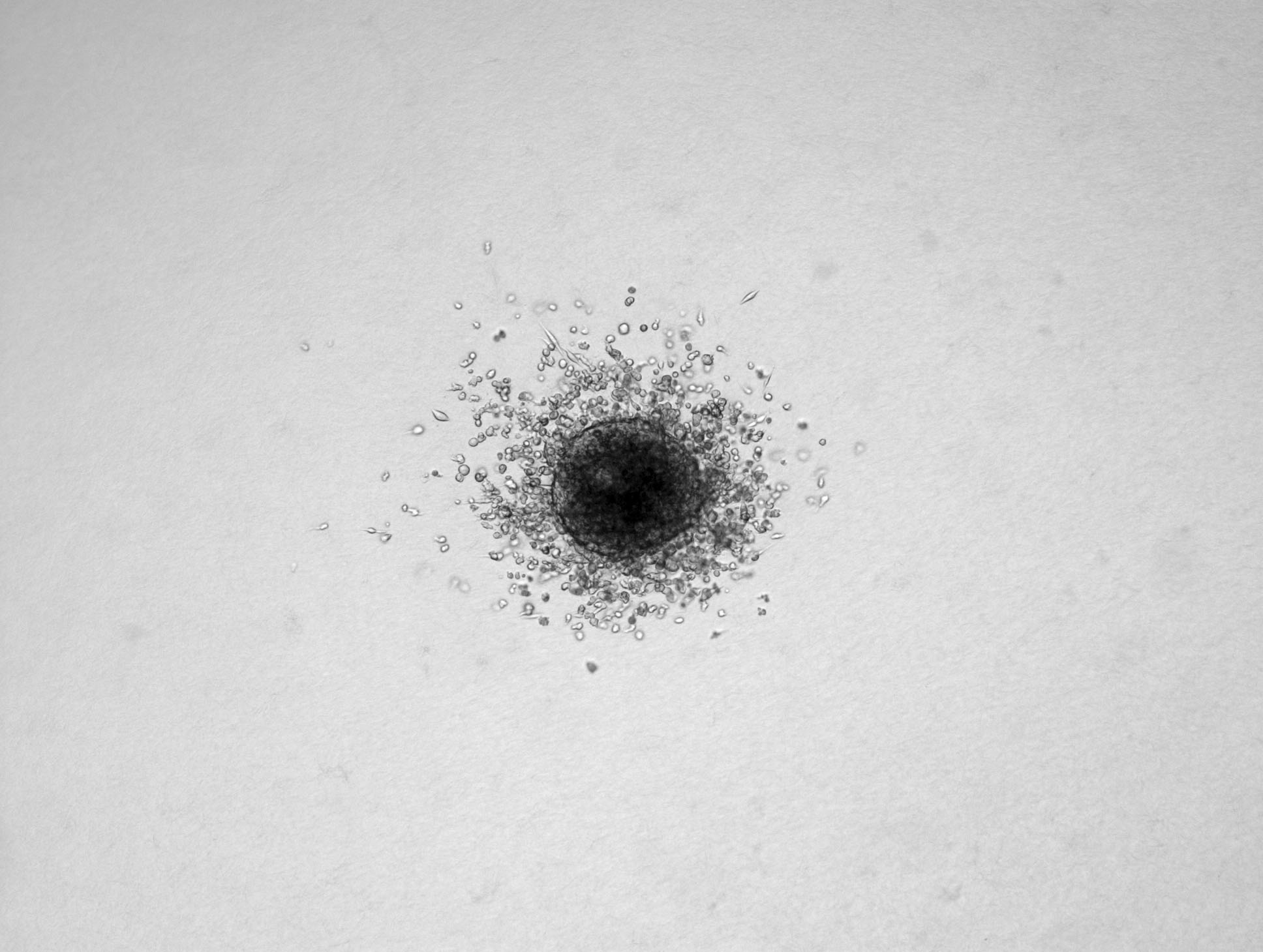

Supplement: Supplementary file 10 — Source Data for Figure 5 [file EMMM-14-e15677-s002.zip › Figure 5/Fig 5C Das (TCDD, D5).jpg]

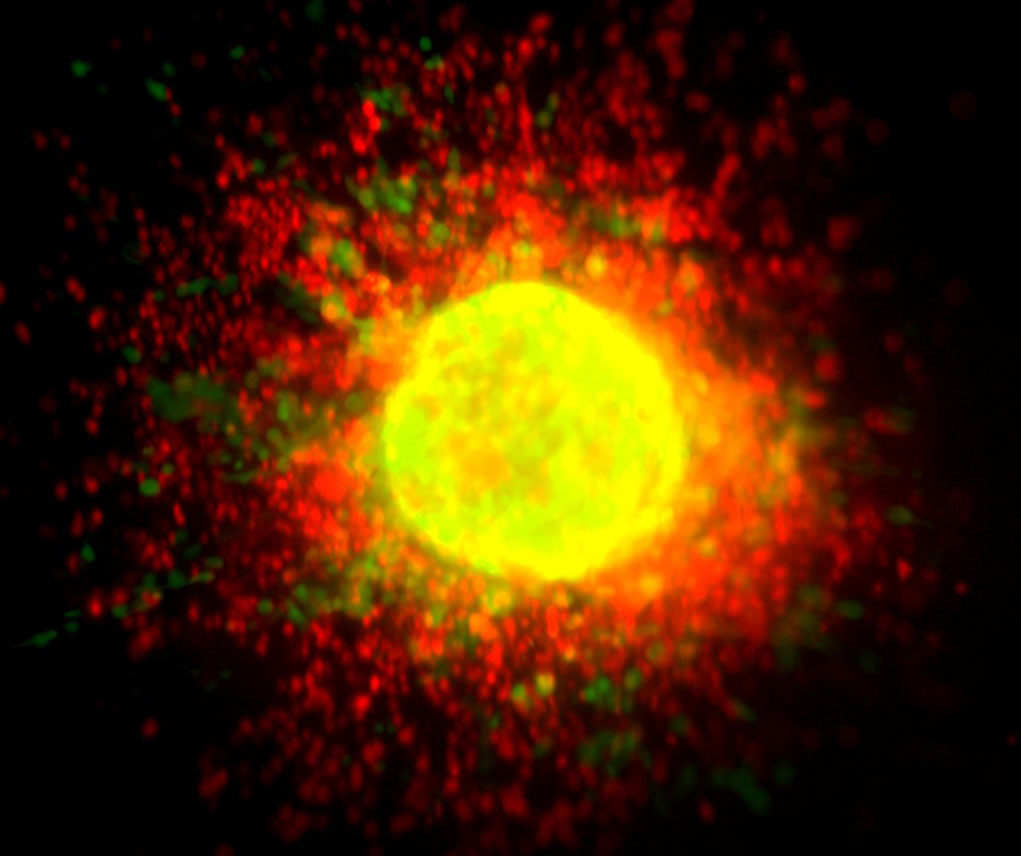

Supplement: Supplementary file 10 — Source Data for Figure 5 [file EMMM-14-e15677-s002.zip › Figure 5/Fig 5B (DMSO, D5).jpg]

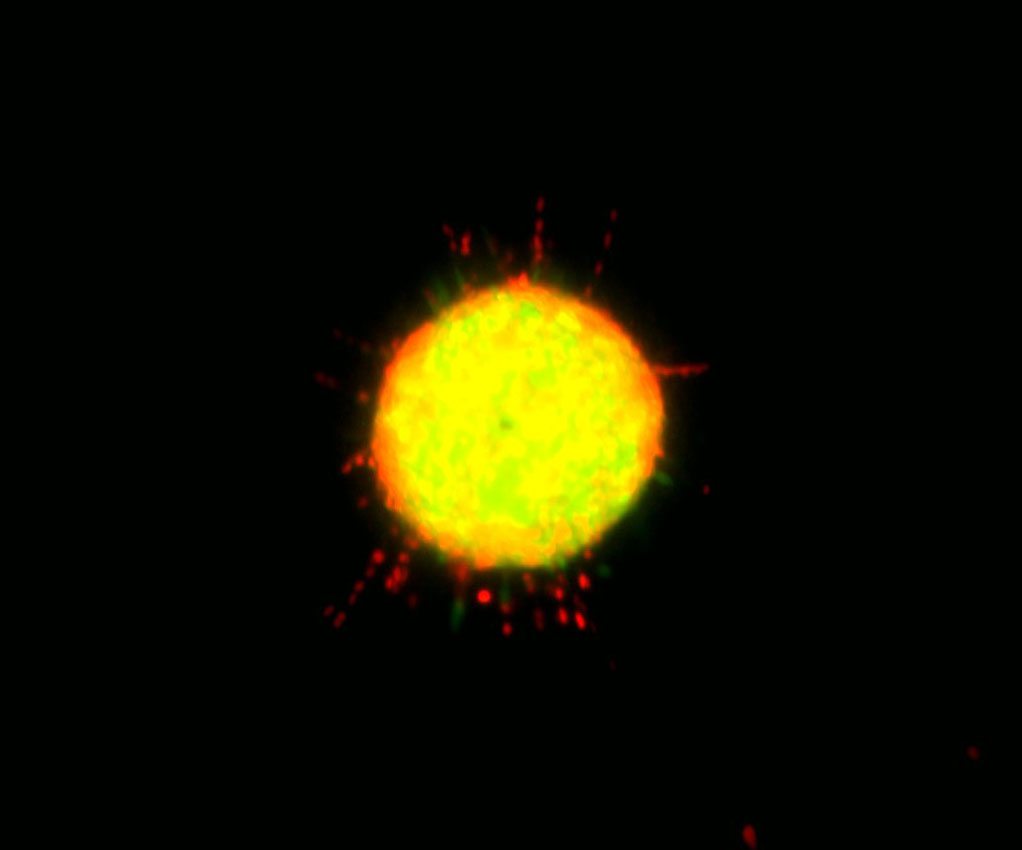

Supplement: Supplementary file 10 — Source Data for Figure 5 [file EMMM-14-e15677-s002.zip › Figure 5/Fig 5B (Bosu, D5).jpg]

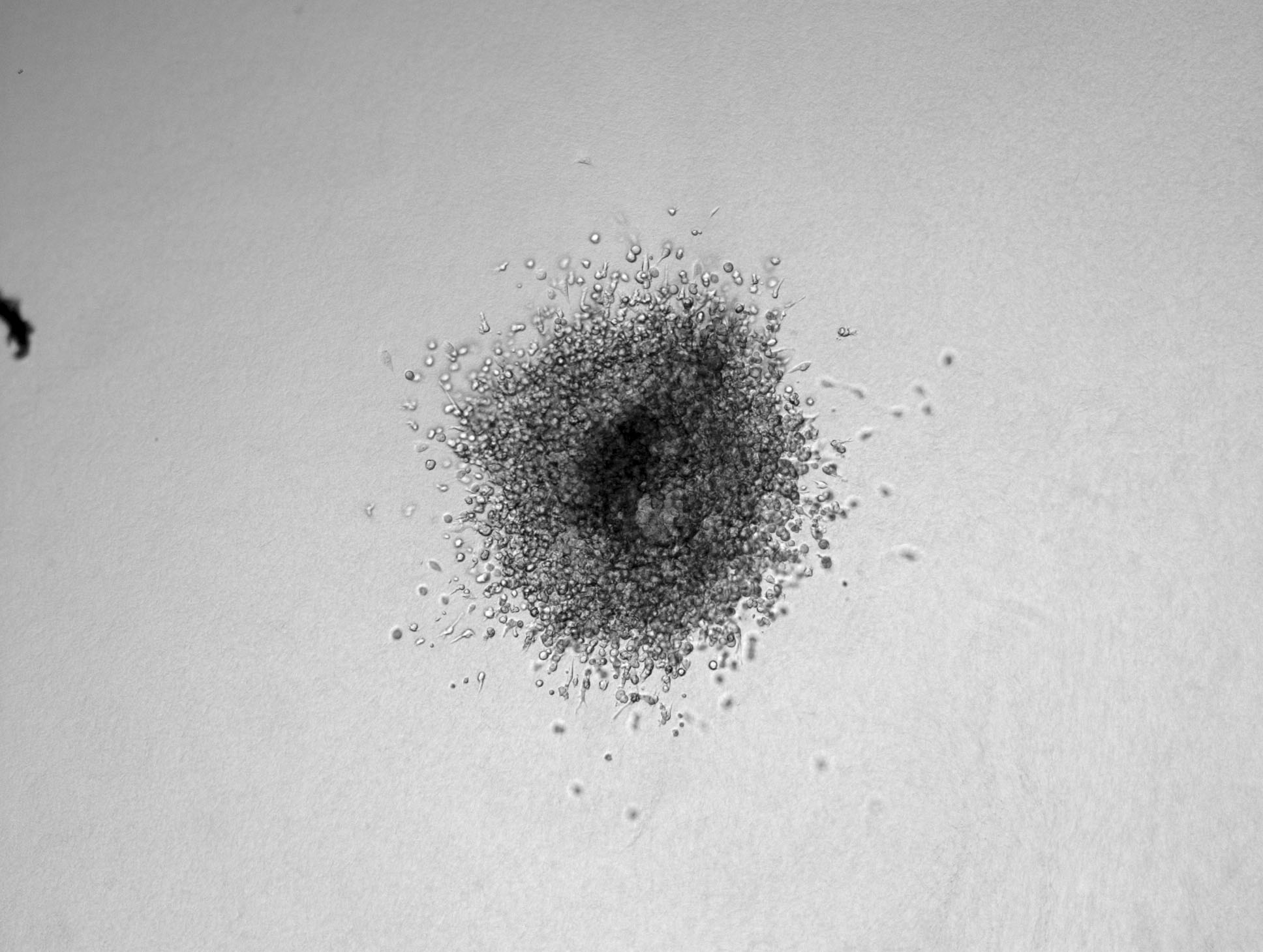

Supplement: Supplementary file 10 — Source Data for Figure 5 [file EMMM-14-e15677-s002.zip › Figure 5/Fig 5C CTR (DMSO, D5).jpg]

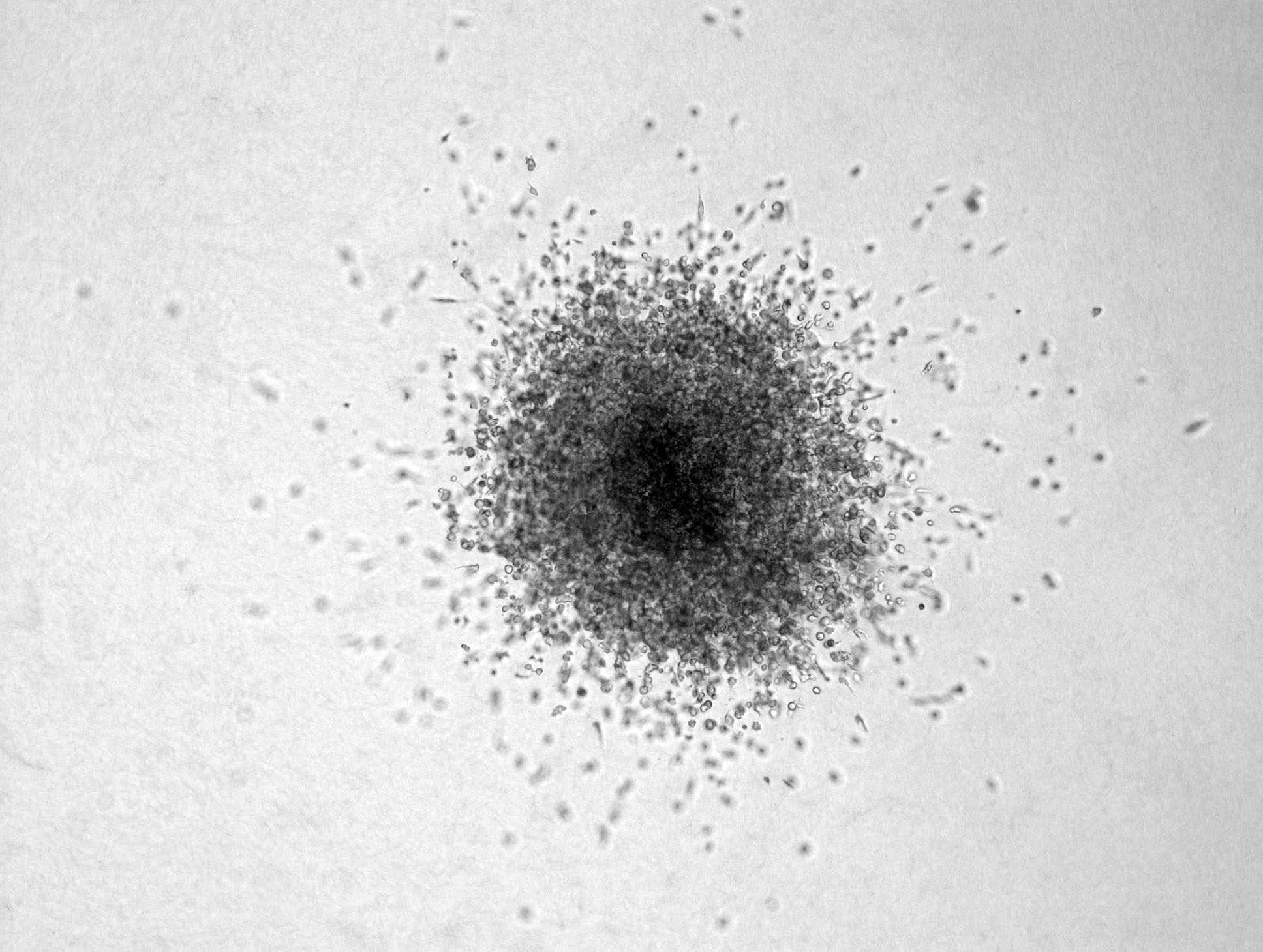

Supplement: Supplementary file 10 — Source Data for Figure 5 [file EMMM-14-e15677-s002.zip › Figure 5/Fig 5C CTR (TCDD, D5).jpg]

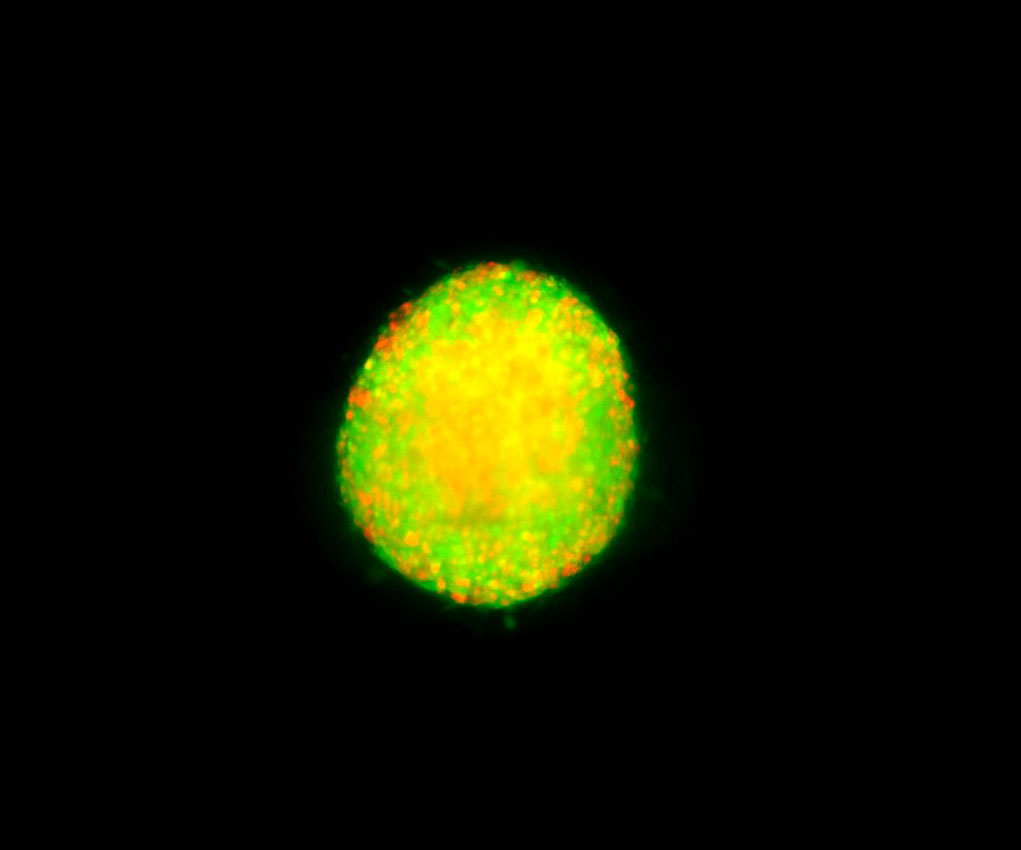

Supplement: Supplementary file 10 — Source Data for Figure 5 [file EMMM-14-e15677-s002.zip › Figure 5/Fig 5B (DMSO, D0).jpg]

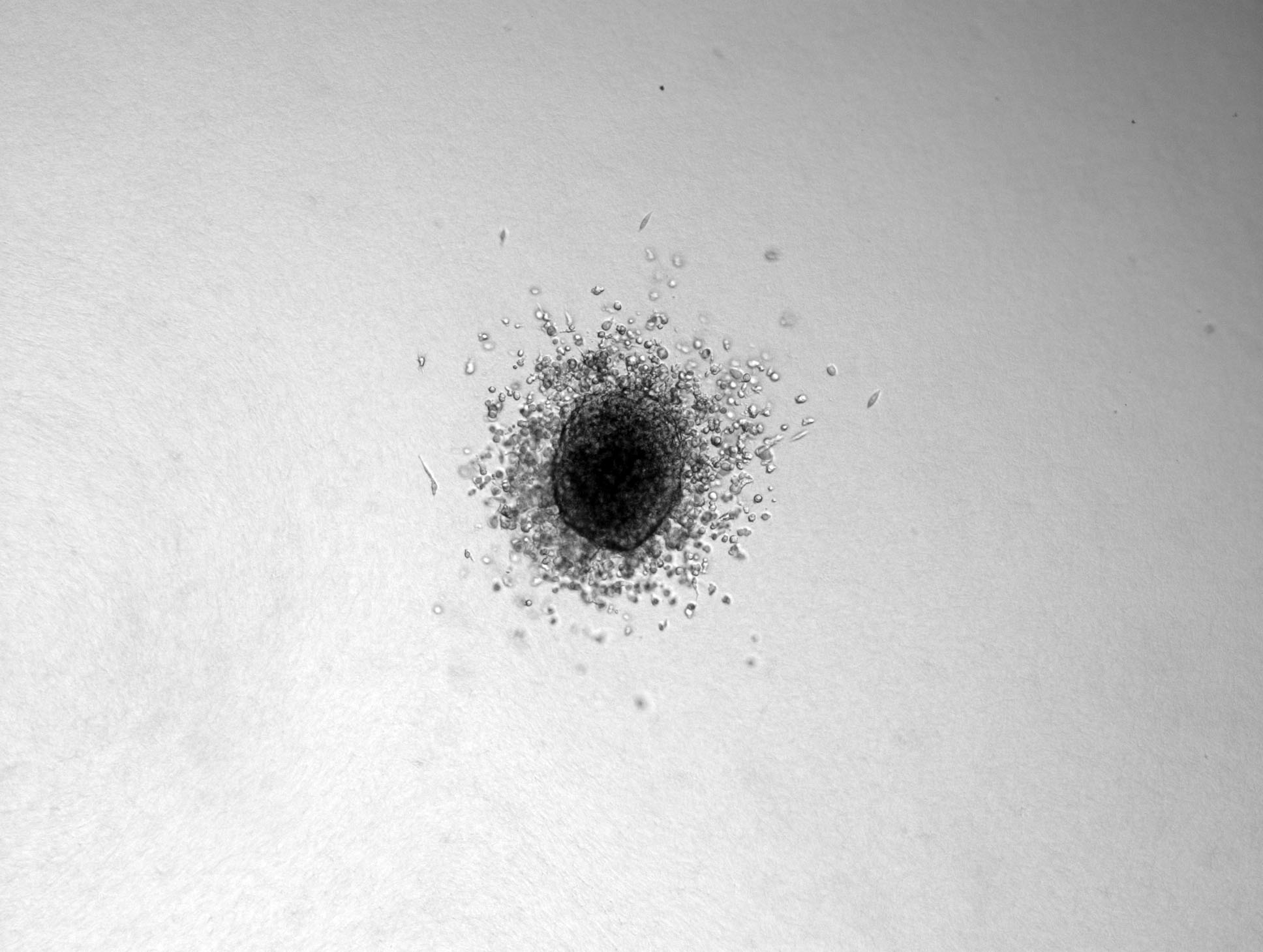

Supplement: Supplementary file 10 — Source Data for Figure 5 [file EMMM-14-e15677-s002.zip › Figure 5/Fig 5C Das (ITE, D5).jpg]

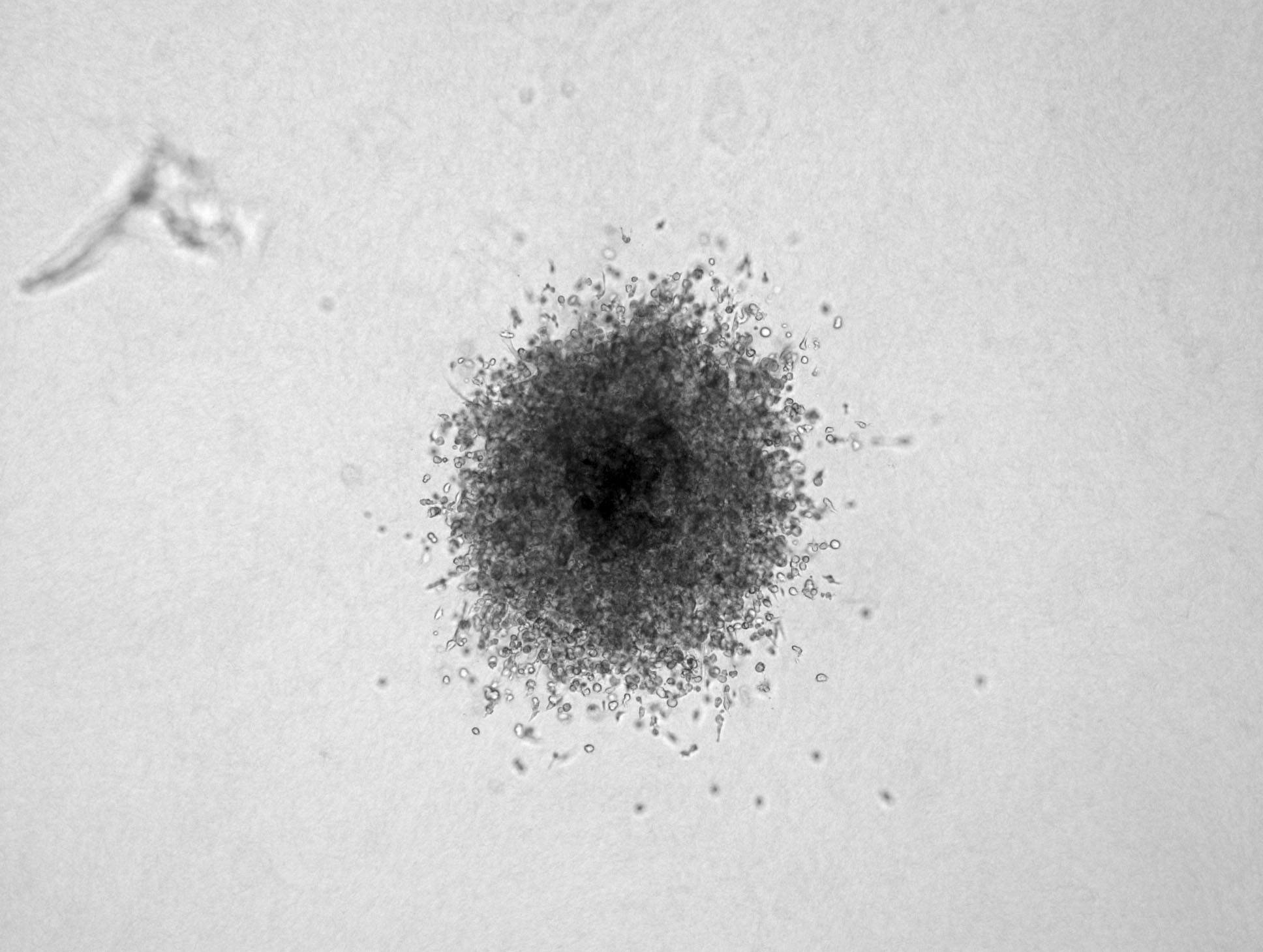

Supplement: Supplementary file 10 — Source Data for Figure 5 [file EMMM-14-e15677-s002.zip › Figure 5/Fig 5C CTR (ITE, D5).jpg]
