# Supplementary material for: The AhR‐SRC axis as a therapeutic vulnerability in BRAFi‐resistant melanoma
Source: EMBO Mol Med. 2022 Oct 28;14(12):e15677. doi: 10.15252/emmm.202215677 (PMC9728058; doi:10.15252/emmm.202215677)
Supplement: Supplementary file 11 — PDF+ [file EMMM-14-e15677-s004.pdf]

Article

SOURCE DATA TRANSPARENT PROCESS OPEN ACCESS

EMBO Molecular Medicine

The AhR-SRC axis as a therapeutic vulnerability in BRAFi-resistant melanoma

Anaïs Paris[1,†,‡], Nina Tardif[1,†,‡], Francesca M Baietti[2,3], Cyrille Berra[1,4], Héloïse M Leclair[1], Eleonora Leucci[2,3], Marie-Dominique Galibert[1,4,*,§] & Sébastien Corre[1,**,§]

Abstract

The nongenetic mechanisms required to control tumor phenotypic plasticity and shape drug-resistance remain unclear. We show here that the Aryl hydrocarbon Receptor (AhR) transcription factor directly regulates the gene expression program associated with the acquisition of resistance to BRAF inhibitor (BRAFi) in melanoma. In addition, we show in melanoma cells that canonical activation of AhR mediates the activation of the SRC pathway and promotes the acquisition of an invasive and aggressive resistant phenotype to front-line BRAFi treatment in melanoma. This nongenetic reprogramming identifies a clinically compatible approach to reverse BRAFi resistance in melanoma. Using a preclinical BRAFi-resistant PDX melanoma model, we demonstrate that SRC inhibition with dasatinib significantly re-sensitizes melanoma cells to BRAFi. Together we identify the AhR/SRC axis as a new therapeutic vulnerability to trigger resistance and warrant the introduction of SRC inhibitors during the course of the treatment in combination with front-line therapeutics to delay BRAFi resistance.

Keywords BRAFi resistance; cell plasticity; expression; melanoma
Subject Categories Cancer; Skin
DOI 10.15252/emmm.202215677 | Received 4 January 2022 | Revised 30 September 2022 | Accepted 30 September 2022 | Published online 28 October 2022
EMBO Mol Med (2022) 14: e15677

Introduction

Deciphering the genetic landscape of cancer led to a better understanding of tumor development, tumor annotation, and classification (Bailey *et al*, 2018). The identification of recurrent driver mutations underscored oncogenic addiction and designed new druggable targets revolutionizing patient care (Berger & Mardis, 2018). However, a major barrier to effective therapy is the capacity of cancer cells to resist. Melanoma represents a pioneering model to comprehend the multiple facets of resistance mechanisms.

The discovery of oncogenic BRAF mutations in about 50% of advanced melanomas has emerged as central, transforming melanoma therapy (Davies *et al*, 2002). The most common BRAF mutation consists of a T to A transition (T1799A), encoding a BRAF^{V600E} oncogenic protein with constitutive kinase activity, leading to downstream MAPKinase signaling activation. Patient-tumors carrying such mutations are treated with BRAF inhibitors (BRAFi) namely vemurafenib (Bollag *et al*, 2010), dabrafenib (Hauschild *et al*, 2012), or encorafenib (Koelblinger *et al*, 2018), in combination with MEK inhibitors (MEKi) respectively cobimetinib (Larkin, 2014), trametinib (Salama & Kim, 2013; Robert *et al*, 2014; Daud *et al*, 2017), and binimetinib (Dummer *et al*, 2018; Shirley, 2018) to overcome BRAF paradoxical activation (Zhang *et al*, 2015) and maximize the therapeutic response. Under such front-line double blockade, patients show remarkable immediate responses. However, the response is transient, with median progression-free survival (PFS) of 15 months and a median overall survival up to 30 months (Michielin *et al*, 2020), followed by the development of resistance, leading to relapse and death (Dummer *et al*, 2018; Shirley, 2018).

Understanding the molecular mechanism of resistance to BRAFi/MEKi double blockade is critical to maximize clinical response. Unlike other oncogenic addicted tumors, namely EGFR driven lung cancer (NSCLC), where the appearance of secondary mutation in the target gene (EGFR) is a common mechanism of resistance to EGFR inhibitors (Kobayashi *et al*, 2005), no BRAF secondary mutation has been so far reported in BRAFi-resistant melanomas.

Resistance to MAPK inhibitors proceeds through different genetic route mainly mutation, amplification mechanisms, leading to reactivation of the MAPK pathway or MAPK-redundant signaling pathway such as activation of the PI3K/AKT pathway, along with the upregulation of tyrosine kinase receptors (TKRs; EGFR, IGF1R, PDGFR, AXL, etc.) (Arozarena & Wellbrock, 2017; Rossi *et al*, 2019; Czarnecka *et al*, 2020).

1 Univ Rennes, CNRS, INSERM, IGDR (Institut de Génétique et Développement de Rennes) – UMR6290, ERL U1305, Rennes, France
2 Laboratory for RNA Cancer Biology, Department of Oncology, LKI, KU Leuven, Leuven, Belgium
3 Trace PDX Platform, Department of Oncology, LKI, KU Leuven, Leuven, Belgium
4 Department of Molecular Genetics and Genomics, Hospital University of Rennes (CHU Rennes), Rennes, France
*Corresponding author. Tel: +33 223 234 705; E-mail: mgaliber@univ-rennes1.fr
**Corresponding author. Tel: +33 223 235 392; E-mail: sebastien.corre@univ-rennes1.fr
[†]These authors contributed equally to this work as first authors
[‡]These authors contributed equally to this work
[§]These authors contributed equally to this work as senior authors

©2022 The Authors. Published under the terms of the CC BY 4.0 license.

In addition to these acquired genetic alterations, a new concept of resistance has emerged based on the capacity of melanoma cells to undergo transcriptomic reprogramming. Single cell transcriptomic analysis showed that the adaptive response to BRAFi is diverse, leading to the generation of a gradient of dedifferentiated cell states from melanocytic to neural crest state (Rambow *et al*, 2018; Tsoi *et al*, 2018). The plasticity of melanoma cells mediates a phenotype switching of the cells, which constitutes a robust escape route to therapy (Hoek *et al*, 2008; Kemper *et al*, 2014; Marin-Bejar *et al*, 2021). Under the control of the microenvironment or intrinsic cell factors, melanoma cells could switch from a proliferative to invasive state, acquiring resistance to targeted therapies. These phenotypic changes are mainly associated with a process of dedifferentiation similar to the epithelial-to-mesenchymal transition (EMT-like) that promotes metastatic spreading (Carreira *et al*, 2006; Hoek *et al*, 2008; Cheli *et al*, 2012; Verfaillie *et al*, 2015; Dilshat *et al*, 2021). Nonetheless, in some cases, melanoma cells still exhibit a differentiated state and are resistant to BRAFi (Tirosh *et al*, 2016; Rambow *et al*, 2018). Transcription factors, such as the master regulator of the melanocytic lineage, the microphthalmia-associated transcription factor (MITF) plays a critical and founding role in directing melanoma cell plasticity (Wellbrock & Marais, 2005; Müller *et al*, 2014; Noguchi *et al*, 2017; Goding & Arnheiter, 2019). While $MITF^{High}$ state is associated with melanocyte differentiation and drives melanoma proliferation (Hoek *et al*, 2008; Rambow *et al*, 2018), the $MITF^{Low}$ (Müller *et al*, 2014) state is associated with drug resistance, supporting the notion of transcriptional balance.

We demonstrated that the Aryl hydrocarbon Receptor (AhR) transcription factor is constitutively activated in a subset of melanoma cells, promoting the dedifferentiation of melanoma cells and the expression of BRAFi-resistant genes (Corre *et al*, 2018). Using two complementary genome-wide CRISPR/Cas9 screens (CRISPR-a and CRISPR-i), we and others further underscored the role of AhR in the acquisition of BRAFi resistance (Gautron *et al*, 2021; Goh *et al*, 2021).

AhR is a ligand-activated transcription factor belonging to the family of the basic-helix–loop–helix (bHLH) Per-Arnt-Sim (PAS) transcription factor. In its inactive state, AhR is part of a cytosolic multi-protein complex that includes heat-shock protein 90, p23, AhR-interacting protein (AIP) and SRC (Enan & Matsumura, 1996; Cox & Miller, 2004; Nukaya *et al*, 2010). Upon ligand binding, AhR dissociates from its chaperone complex and translocates into the nucleus, where it interacts with its partner the AhR nuclear translocator (ARNT). AhR-ARNT nuclear dimers regulate the expression of target genes through recognition and binding to xenobiotic-responsive elements (XREs) located within the promoter of their target genes allowing cell specific gene expression programs. Remarkably, through ligand-binding interaction, AhR has the capacity to integrate environmental and cell-dependent signals (Denison *et al*, 2002) to shape and adapt the cell response, making AhR a very attractive candidate in regulating melanoma plasticity.

In addition, AhR activation has been shown to participate in the phosphorylation of the non-receptor tyrosine kinase SRC (Y416 residue) (Randi *et al*, 2008; Tomkiewicz *et al*, 2013; Fallahi-Sichani *et al*, 2017). SRC is known to be involved in many cellular functions, including the promotion of tumor-cell survival, motility, and invasion, through a rapid activation of focal adhesion kinase (FAK) contributing to cell migration and EMT (Nihal & Wood, 2016; Patel *et al*, 2016). However, the relation between AhR and SRC has not yet been explored in melanoma.

Herein, we aimed to delineate the role of AhR in orchestrating melanoma phenotypic switching during the acquisition of resistance through genomic and non-genomic routes. In particular, we pinpoint the crosstalk between AhR and SRC in reshaping cell fate and identify the AhR/SRC axis as a new therapeutic vulnerability for the treatment of BRAFi-resistant melanoma.

Results

AhR controls acquisition of the invasive phenotype of melanoma

AhR is markedly expressed in highly dedifferentiated, resistant, and invasive melanoma cells, mediating resistance to BRAFi (Corre *et al*, 2018). Accordingly, genetic depletion of AhR in BRAFi-resistant SKMel28 melanoma cells (SK28R) by CRISPR Cas9 technology (AhR KO; Fig EV1A) significantly reduced their resistance to various BRAFi (vemurafenib, dabrafenib, and encorafenib; Fig 1A). We specifically evaluated the role of AhR in controlling cell migration by performing wound-healing assays of BRAFi-sensitive or resistant melanoma cells (called SK28S and SK28R, respectively) after genetic depletion of AhR (SK28S/R KO; Fig 1B) or chemical inhibition using its specific antagonist (CH-223191, 10 μM; Fig 1C). The loss or inhibition of the AhR significantly reduced migration capacity of melanoma cells (Fig 1B and C). Next, we analyzed the invasive properties of SK28S and SK28R melanoma cell lines using tumor-spheroid assays, which mimic the 3D architecture of melanoma. BRAFi-resistant cells were far more invasive than BRAFi-sensitive cells (Fig 1D) and the loss of the AhR transcription factor significantly reduced invasion of SK28R cells on a collagen matrix at 4 days (Fig 1D). Comparable results were obtained by chemical inhibition (CH-223191, 10 μM) of AhR in SK28 cells and no effect was observed in AhR KO cells (Fig 1E), underscoring the specificity of the CH-223191 and AhR function.

The role of AhR transcription factor in governing the resistance and invasive capacity of melanoma cells was further highlighted after increasing the endogenous expression of AhR by CRISPR/SAM technology in melanoma cells expressing low levels of AhR protein (501Mel) or after rescuing SK28R AhR KO cell lines with a constitutively activated form of AhR, (CA-AhR; McGuire *et al*, 2001). CRISPR/SAM stable expression of endogenous *AhR* in 501Mel cells was obtained using two different single-guide RNAs (sgRNA) targeting the *AhR* promoter region, with a subsequent increase in the capacity to mediate the expression of AhR (Fig 2A). Increased AhR expression reduced slightly BRAFi sensitivity (Fig 2B) and increased the invasive capacity of the 501Mel cells (Fig 2C) according to the ability of the sgRNA to induce AhR expression. Comparable results were obtained with the stable SK28R KO cell line expressing the constitutively active form of AhR (CA-AhR; Fig 2D). Significant increase of BRAFi resistance (Fig 2E) and invasive capacity were observed (Fig 2F). Thus, both AhR expression and its activation control the phenotype of melanoma cells and their sensitivity to BRAFi.

The sensitivity of melanoma cells to BRAFi has been associated with a highly differentiated cell state under the control of the MITF transcription factor (i.e., $MITF^{high}$ or pigmentation signature) (Rose

 ©2022 The Authors

et al, 2016; Smith *et al*, 2016; Rambow *et al*, 2018). Conversely, we showed that AhR transcription factor participates in BRAFi resistance (Corre *et al*, 2018). To characterize the molecular role of AhR in such transcriptional reprogramming, we compared specific gene expression signatures (Invasion, Resistance, Proliferation, Melanocytic… corresponding to the median of gene expression of previously established gene-signatures associated with melanoma phenotype as described in Appendix Table S1). These comparisons were performed with the SK28S and SK28R melanoma cell lines before and after genetic depletion of AhR (SK28S/R KO) and its rescue with AhR constitutive active form (CA-AhR; from RNAseq data, GSE166617; Figs 2G, and EV1B and C). As we previously described and underscored here in Fig 2G, the β-signature (associated with BRAFi sensitivity; Corre *et al*, 2018) was highly represented in the proliferative, differentiated (Melanocytic and Transitory; Tsoi *et al*, 2018), and BRAFi-sensitive cell lines (SK28S). Conversely, the α-signature depicting canonical activation of AhR (Corre *et al*, 2018) was most prominent in dedifferentiated (neural crest-like and undifferentiated) BRAFi-resistant lines and co-occurred with the resistance signature (SK28R; Fig 2G). The absence of AhR expression (SK28R-KO) significantly decreased the expression of these gene-signatures (Fig 2G), while the re-expression of the constitutively active form of AhR (SK28R-KO CA-AhR) led to their overexpression (Fig 2G). Interestingly, these AhR associated signatures segregate BRAFi resistant melanoma cells from the sensitive ones (Cancer Cell Line Encyclopedia – CCLE RNA-seq data (Barretina *et al*, 2012)) and in Melanocytic-Transitory from Neural Crest like-Undiferentiated melanoma cells (GSE80824 (Tsoi *et al*, 2018); Appendix Fig S1A) and in invasive melanoma cell lines (Appendix Fig S1B; Verfaillie *et al*, 2015). Besides the role of AhR in the regulation of resistance gene expression, we underscored its role in the acquisition of the dedifferentiated/invasive/mesenchymal phenotype. Overall, these results report that AhR mediates specific gene signature controlling the phenotypic switch of melanoma cells.

AhR regulates the expression of genes associated with BRAFi resistance, invasion, and dedifferentiation phenotypes of melanoma

To further decipher the direct role of AhR in the acquisition of the BRAFi-resistant associated phenotype we compared the previously established gene phenotype-signatures (Appendix Table S1) with RNAseq data from 501Mel cells exposed to BRAFi (Vem, 1 μM) or AhR ligand (TCDD, 10 nM) for 48 h (GSE104869 (Corre *et al*, 2018)), and with ChiP-Seq data identifying AhR target genes following exposure to TCDD (GSE90550 (Yang *et al*, 2018); Fig 3A and Appendix Fig S2A). This led to the selection of 216 genes predicted to be regulated by AhR (Appendix Fig S2A). Among these genes, 92 were significantly enriched (GSEA) in the sensitive/differentiated phenotype and 75 in the resistant/dedifferentiated one (Appendix Fig S2B and C, and Dataset EV1). The 50 most highly enriched genes between these two states (25 sensitive/differentiation genes (green) and 25 resistant/dedifferentiation genes (red), Dataset EV1 and Fig 3B), segregated sensitive and resistant SK28 cells (GSE166617; Fig 3B). AhR knockout (SK28R KO) and expression of its constitutively active form confirmed the involvement of AhR in the regulation of these resistant genes (in red, Fig 3B and C). Comparable results were obtained in 501 Mel cells overexpressing endogenous *AhR* (CRISPR/SAM; Fig EV1D). Finally, these 50 AhR-associated genes segregated BRAFi-resistant melanoma cells from sensitive ones (CCLE RNA-Seq data (Barretina *et al*, 2012)) and Melanocytic-Transitory melanoma cells from Neural Crest like-Undifferentiated ones (GSE80824 (Tsoi *et al*, 2018); Fig 3B).

Interestingly, several of these AhR-associated genes have been involved in the aggressiveness of melanoma or other cancers (Appendix Table S2) and have been associated with a poor prognosis (*ABCG2*, *COL1A1*, *COL6A1*, *COL6A2*, *TGFBI*). *CCL2*, *CRIM1*, *COL1A1*, *6A1*, *6A2* participate in cell migration, invasion, or EMT and *ABCG2*, *ALDH1A1*, *NES* are cancer stem-cell markers. Furthermore, AhR has been shown to directly regulate the expression of some of them (*ABCG2*, *CCL2*, *STC2*, etc.; Appendix Table S2), supporting the role of AhR in resistance.

We next explored the clinical relevance of this AhR-associated genes-signature by first examining melanoma samples from the TCGA cohort (Anaya, 2016). Among, the analyzed melanoma samples ($n = 454$), 17% of Patients strongly expressing AhR-associated resistance genes (red box) showed significantly lower overall survival than those highly expressing genes for sensitivity (blue box; Appendix Fig S3A). We investigated the expression of these genes in melanoma patients exposed to single drug-blockage (BRAFi) by classifying their melanoma biopsies during the course of medication and disease progression (baseline, early: DP1, intermediate: DP2, late: DP3; RNAseq dataset from Hugo *et al* (2015), GSE65185). Again, their expression level decreased at the beginning of the treatment (response to BRAFi phase: DP1) and slowly but significantly increased during the acquisition of BRAFi resistance (Fig 3D). Using, additional RNAseq data from melanoma cells lines (M229 and M397; GSE110054 (Tsoi *et al*, 2018)), we confirmed that the acquisition of BRAFi resistance correlates with a late increase in the expression of AhR-associated gene-signature (Appendix Fig S3B). The BRAFi/MEKi double blockade led to similar reprogramming of gene expression (Appendix Fig S3C).

Canonical activation of AhR triggers the SRC pathway to promote the BRAFi-resistant/invasive phenotype of melanoma

AhR is part of a cytosolic multiprotein complex with HSP90 and the SRC kinase (Enan & Matsumura, 1996; Rey-Barroso *et al*, 2013) (Fig 4A). We investigated the potential cross-regulation between AhR and SRC signaling. We performed co-immunoprecipitation experiments to determine whether AhR and SRC are present in the same protein complex in melanoma cells (SKMel28). AhR was detected in the SRC immunoprecipitate (Fig 4A) and this interaction was confirmed by proximity ligation assay (Fig EV2A). We next tested whether AhR controls the activation of the SRC kinase in the context of BRAFi resistance. To this end, we examined their protein levels and the phosphorylated form (AhR, SRC, P-SRC, etc.) in four melanoma cell lines with increasing levels of BRAFi resistance (Figs 4B and EV2B). Concomitant to BRAFi resistance, we observed increased AhR protein levels and increased activation of SRC after phosphorylation on residue Tyr^{416} (Y416). Activation of FAK (phosphorylation on tyrosine 576/577) followed SRC phosphorylation (Figs 4B and EV2B). We next examined SRC and FAK protein levels and phosphorylation state in SK28R cells expressing or lacking AhR (Figs 4C and EV2C). While constitutive loss of AhR did not significantly induce SRC or FAK activation, rescue experiments with AhR

©2022 The Authors

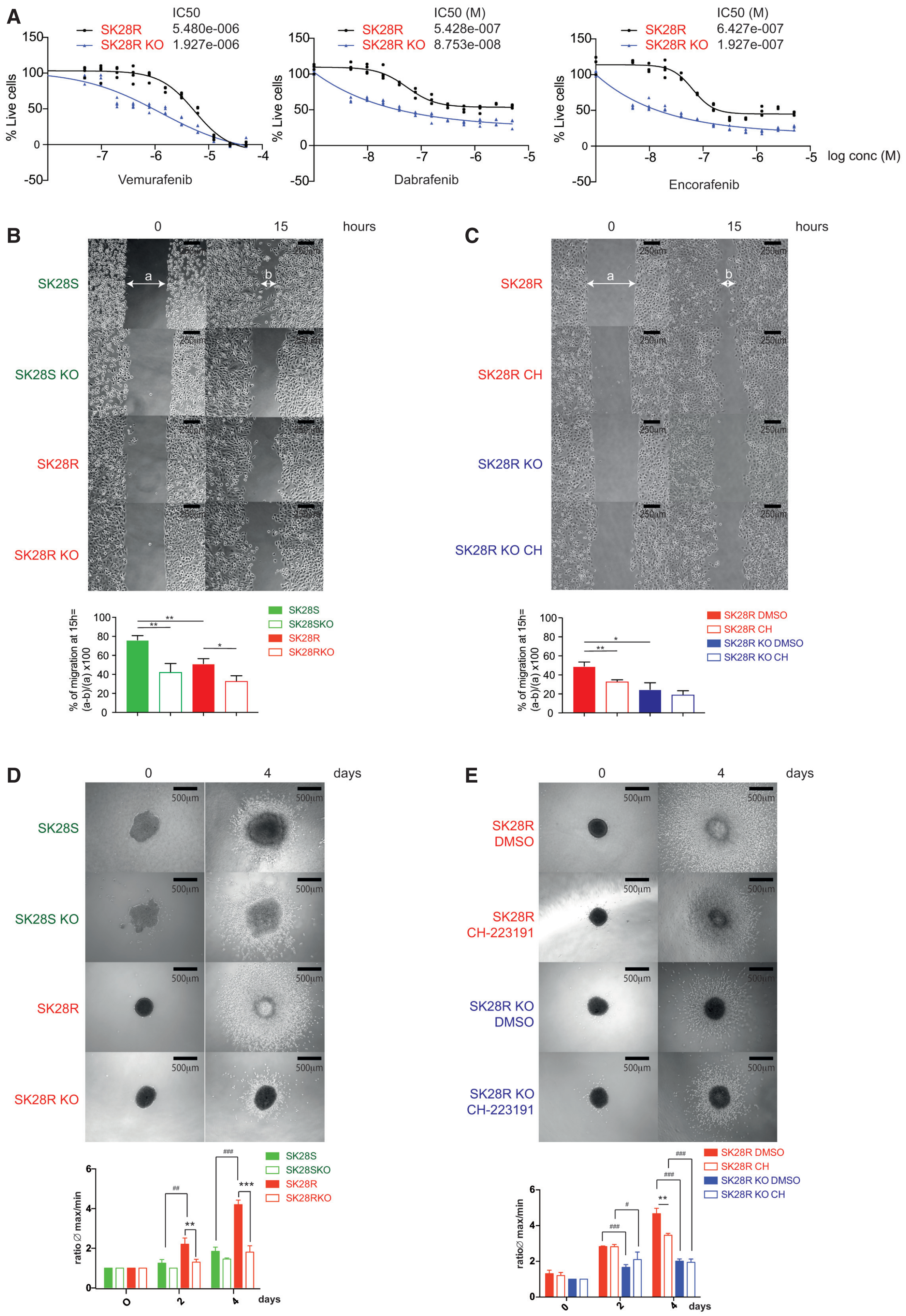

Figure 1.

©2022 The Authors

Figure 1. Loss of AhR reduces the invasive phenotype of BRAFi-resistant melanoma cells.

A Vem sensitivity was established in BRAFi-resistant SK28 cells before and after knockout out of *AhR* by CRISPR/Cas9, by cell density measurements for 4 days after treatment (every 2 days), with an increasing concentration of BRAFi (vemurafenib, dabrafenib, or encorafenib). The IC50 (M) was calculated using GraphPad (PRISM9.0®).

B, C Wound healing assays were performed using IBIDI® chambers to evaluate the role of the AhR on cell migration. Images of the wound were captured using an Axio Vert.A1 inverted microscope (Carl Zeiss®) at 5× magnification. The histogram represents the mean ± s.d. Wound closure was determined by measuring the distance between the edges of the wound at time 0 and 15 h ($n = 3$ independent technical experiments for each cell lines or conditions) and compared using unpaired t-tests with the Sidak–Bonferroni method. (B) Results obtained with BRAFi-sensitive or resistant SK28 cells KO for the AhR in the absence of treatment. ($n = 3$ independent technical experiments for each cell lines). (C) Results obtained for the migration assay (0–15 h) for SK28 R cells KO or not for the AhR after treatment or not with 10 μM CH-223191. ($n = 3$ independent technical experiments for each cell lines or conditions, mean ± s.d.). Statistical analysis using unpaired t-tests with the Sidak–Bonferroni method has been performed between the mean of the three independent experiments.

D Three-dimensional spheroid growth of BRAFi-sensitive or resistant SK28 cells KO before or after knockout of AhR by CRISPR/Cas9 in the absence of treatment. Images were captured 4 days after implantation of the spheroids into collagen gel. ($n = 4$ independent technical experiments, mean ± s.d.).

E Three-dimensional spheroid growth of BRAFi-resistant SK28 cells KO before or after knockout of AhR by CRISPR/Cas9 after daily treatment with the specific AhR inhibitor CH-223191 (5 μM) for 1 week or in the absence of treatment. Images were captured 4 days after implantation of the spheroids into collagen gel. ($n = 3$ independent technical experiments, mean ± s.d.). Statistical analysis using unpaired t-tests method has been performed with the Sidak–Bonferroni method, ($P < 0.01$ ##, **, $P < 0.001$ ###, ***).

Source data are available online for this figure.

constitutive active form (CA-AhR) led to a significant phosphorylation of SRC and FAK (Figs 4C and EV2C). Over-night exposure of SK28R cells to AhR canonical ligands (BaP, indirubin, ITE, TCDD, and FICZ), induced a massive phosphorylation of SRC on the Tyr[416] residue. Concomitantly, AhR level diminished, underscoring its activation and subsequent degradation (Fig 4D). The level of SRC phosphorylation (P-Y416) increased with increasing doses of the AhR ligand ITE, (Fig 4E) within only a few minutes after activation (Fig EV2D and E). This resulted in the up-regulation of the expression of SRC-related genes, such as *THBS1* and *MMP1* (Said *et al*, 2017; Fig EV2F).

Sustained canonical activation of AhR mediates BRAFi resistance and the activation of the SRC/FAK athway. To delineate the contribution of AhR-SRC axis in resistance acquisition, we established the gene expression profile (RNA-seq) of SK28R cells exposed to AhR agonist ITE (5 μM, 24 h) or to dasatinib (Das), a specific inhibitor of SRC (1 μM, 24 h). Comparative analysis of differentially expressed genes in SK28R cells exposed to ITE (10 μM for 24 h; Appendix Fig S4A) or Das (1 μM for 24 h; Appendix Fig S4B) allowed the identification of a significant number of genes with inversely correlated expression patterns (Appendix Fig S4C and D). Functional annotation (Dataset EV3) identified differentially expressed genes in focal adhesion (*PDGFC*, *THBS1*, *ITGA3*...), PI3K-Akt signaling, ECM-receptor interaction; pathways previously shown to be associated with BRAFi resistance and invasion (Ruffini *et al*, 2013; Vizkeleti *et al*, 2017; Zhang *et al*, 2020).

GSEA in different melanoma cell lines from the CCLE (Barretina *et al*, 2012), and Tsoi *et al* datasets (GSE80824 (Tsoi *et al*, 2018)) using oncogenic signature gene sets (https://www.gsea-msigdb.org/gsea/msigdb/genesets.jsp?collection=C6) underscored that the activation of several pathways (EGFR, YAP, KRAS, TGFβ, Integrin, etc.) correlates with the mechanisms of BRAFi resistance associated with the dedifferentiation process (Appendix Fig S5A and Datasets EV1 and EV2). They include the SRC, FAK, and focal-adhesion pathways. Such induction was also observed in both BRAFi-resistant melanoma cell lines (Fig 4F and Appendix Fig S5B; Tsoi *et al*, 2018) and patients (Appendix Fig S5C; RNAseq dataset from Hugo *et al* (2015), GSE65185). RNAseq performed on SK28 cells before and after knockout out of *AhR* or after canonical activation of the transcription factor confirmed the role of the AhR to induce the expression of genes (in bold) belonging to the integrin, SRC, FAK, and focal-adhesion pathways (Fig 4G and Appendix Fig S6A and B). Together this underlines AhR-induced genomic and non-genomic reprogramming of melanoma cells.

Inhibition of SRC sensitizes melanoma cells to BRAFi treatment and disrupts the acquisition of an invasive phenotype

Having pinpointed the cellular role of AhR in directing BRAFi resistance, we explored new therapeutic opportunities. Using the CellMiner database (https://discover.nci.nih.gov/cellminercdb), we correlated the therapeutic efficacy of a library of 300 chemical compounds (IC50) according to *AhR* mRNA levels (Z-score) in various cancer cell lines (lung, brain_CNS, breast, skin; Appendix Fig S7). Volcano plots showed a significant correlation ($P < 0.001$) for several drugs and scored the SRC inhibitor (dasatinib, Das) to be potentially effective in the context of BRAFi resistance (Appendix Fig S7). Correlative analysis (Das efficacy/gene expression) further showed that the SRCi Das was more effective in cell lines with a high level of *AhR* mRNA and strongly expressing genes mediating resistance, invasion, and melanoma dedifferentiation (Appendix Fig S8A and Table S3). The expressions of genes associated with AhR and SRC/FAK signatures were strongly correlated with the efficacy of Das (Appendix Fig S8B and C).

The two ATP-competitive protein tyrosine kinase inhibitors of SRC (bosutinib (Bos), dasatinib (Das)) were very effective resulting in the complete loss of the phospho-activated SRC form (P-Y416) in SK28R melanoma cells (WT, KO-AhR, CA-AhR; Fig EV3A). Concomitantly, we observed a decrease in the expression of SRC related genes (*THBS1*, *MMP1*; Fig EV3B). Treatment of SK28R melanoma cells with increasing doses of SRC inhibitors (Bos or Das) at different doses (10–500 nM) in combination with increasing doses of Vem significantly increased BRAFi sensitivity (Fig 5A). To analyze the effect of SCRi on cell viability, SK28R melanoma cells were treated alone with increasing doses of SRC inhibitors (Bos or Das, up to 0.5 μM) or in combination with BRAFi. Alone SCRi affected cell viability when used at relatively high concentration (> 0.125 μM for Bos and > 0.031 μM for Das). In contrast, when

used in combination with BRAFi, the effect on cell viability was observed at low doses of SRCi (< 0.015 μM for Bos and < 0.0078 μM for Das; Fig 5A). Since it has been shown that SRCi alone had poor effect on BRAFi-sensitive melanoma cells, together it suggests that SCRi may resensitize resistant-melanoma cells to BRAFi.

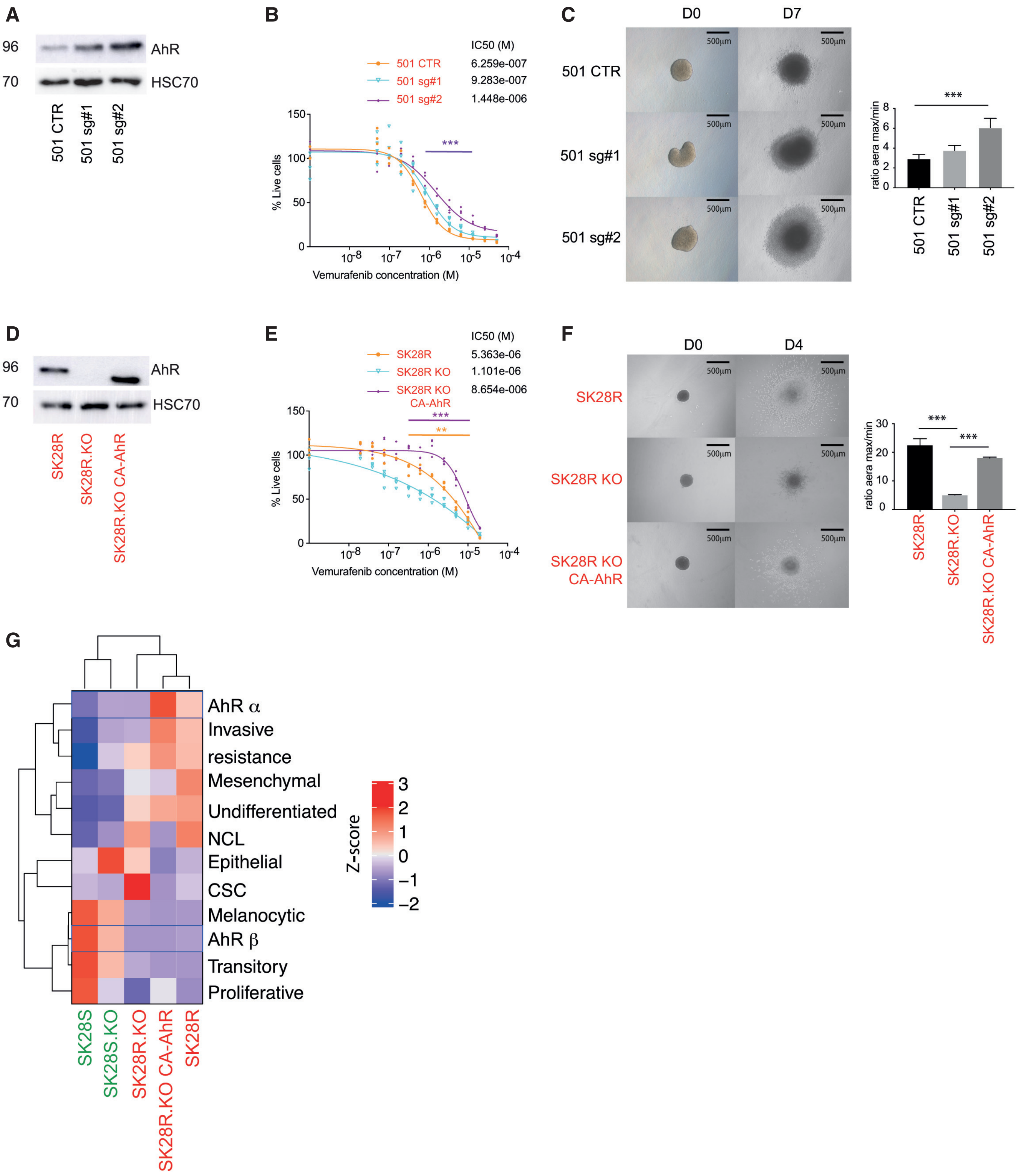

Figure 2.

©2022 The Authors

Figure 2. The activated form of AhR rescues the invasive and BRAFi-resistant phenotype of melanoma cell lines.

A AhR Protein levels in 501Mel CTR cells and those transduced with sgRNA targeting *AhR* (#1, #2) were analyzed by western blotting.

B BRAFi sensitivity was established in 501Mel cells or with endogenous overexpression of the constitutive form of the AhR (sgRNA, CRISPRi), by measuring cell density for 4 days after treatment (every 2 days), with an increasing concentration of BRAFi (vemurafenib). The IC50 (M) was calculated using GraphPad (PRISM9.0®). Statistical analysis (two-way ANOVA) has been performed between the mean of four independent experiments (SK28R KO vs. SK28R) at different concentrations; $P < 0.001$ ***.

C Three-dimensional spheroid growth of 501Mel cells. Images were captured 7 days after spheroid implantation ($n = 3$ independent technical experiments, mean ± s.d.). Each histogram represents the mean ± s.d. Comparisons with control were performed using unpaired *t*-tests with the Sidak–Bonferroni method, $P < 0.001$ ***.

D AhR Protein levels in SK28R WT, KO, and KO CA-AhR cells were analyzed by western blotting.

E BRAFi sensitivity was established in SK28R cells in the absence of AhR (SK28R KO (CRISPR-Cas9) or after overexpression of the constitutive form of AhR (CA-AhR), by measuring the cell density for 4 days after treatment (every 2 days), with increasing concentrations of BRAFi (vemurafenib). The IC50 (M) was calculated using GraphPad (PRISM9.0®). Statistical analysis (two-way ANOVA) has been performed between the mean of four independent experiments (SK28R KO or SK28R KO CA-AhR vs. SK28R) at different concentrations; $P < 0.01$ **, $P < 0.001$ ***.

F Three-dimensional spheroid growth of SK28 cells KO or not for AhR by CRISPR/Cas9 and rescued by the constitutive active form of AhR (KO CA-AhR). Images were captured 4 days after spheroid implantation ($n = 3$ independent technical experiments, mean ± s.d.). Comparisons were performed using unpaired *t*-tests with the Sidak–Bonferroni method.

G Expression heatmap for various gene signatures (established by the median of expression for specific genes) (invasive vs. proliferative, alpha, beta, resistant, melanocytic, transitory, neural crest-like, and undifferentiated, see Appendix Table S1) in SK28 BRAFi-sensitive or resistant cell lines KO or not for AhR by CRISPR/Cas9 and rescue with the constitute active form of AhR (KO CA-AhR; GSE166617). Genes and clusters with similar expression profiles across the cohort are placed close to each other in the grid. The scale corresponds to the Z scores.

Source data are available online for this figure.

Das also sensitized other resistant melanoma cell lines (M229R and M238R), to BRAFi (Fig EV3C). In addition to their roles in sensitizing melanoma cells to BRAFi, the SRC inhibitors Bos and Das, even at low doses (1 μM), prevented the invasive capacity of wild-type AhR melanoma cells (red) in three-dimensional spheroid assays (Fig 5B). Das was also able to reduce the low-invasive capacity of KO-AhR melanoma cells (green; Fig 5B). Remarkably, Das blocked invasion induced by AhR activation (ITE, TCDD; Fig 5C) without affecting AhR protein level (Fig 5D) but by significantly reducing AhR transcriptional activity, alone or after AhR activation by TCDD measured by Luciferase assay (Fig 5E). To further support this identified AhR/SRC cross-regulation, we performed RNAseq on SK28R cells before or after treatment with Bos or Das and characterized the effect of SRC inhibitors on gene-reprogramming signature and AhR-target genes. Both inhibitors significantly decreased the expression of genes associated with SRC, FAK, focal adhesion and invasive/dedifferentiation signatures and AhR-targets (Fig 5F).

This *in vitro* evidence prompted us to examine the clinical relevance of using SRC inhibitors to resensitize BRAFi-resistant tumors to BRAFi. To this end, we used the Mel006R BRAFi-resistant patient-derived xenograft (PDX) mice model. The PDX line MEL006R is a $BRAF^{V600E}$ mutant cutaneous melanoma derived from MEL006 PDX lesions at relapse (Vendramin *et al*, 2021) upon acquisition of resistance to BRAFi/MEKi (Dabrafenib and Trametinib). Once tumors reached 200 mm^3, grafted mice were treated with different treatment regimens: single-drug regimens (BRAFi or SRCi alone) or sequential administration (SRCi alone during the early growth phase of the tumor (16 days) followed by a BRAFi/SRCi double blockade). The growth of the tumor was monitored each 2 days until the tumor reached 1,500 mm^3 (Fig 6A). As anticipated, BRAFi alone was largely ineffective in controlling the tumor growth of this BRAFi-resistant PDX (Figs 6B and EV4A). On the contrary, SRCi alone induced significant control of the tumor growth and the mice under SRCi survived significantly longer than the ones treated with BRAFi alone (Figs 6B and EV4A). Together this reinforces the role of SRC activation as an escape route to BRAFi. Remarkably, introducing BRAFi, 16 days post treatment with SRCi (Fig 6A) or by combining BRAFi and dasatinib from the start of treatment (Appendix Fig S9) significantly diminished the tumor growth rate. As a consequence, those mice survived significantly longer, with an overall survival rate almost doubled compared to mice treated with BRAFi alone. In conclusion, dasatinib after specific inhibition of SRC phosphorylation (Fig EV4B) significantly resensitized resistant tumors to BRAFi treatment and significantly increased the overall survival (Fig 6B–D). These results emphasize the therapeutical interest of SRCi for BRAFi-resistant patients.

Discussion

The acquisition of resistance to targeted therapy can be in part mediated by transcriptional reprogramming, eliciting a phenotypic

Figure 3. AhR regulates the expression of genes associated with the BRAFi-resistant/dedifferentiated phenotype of melanoma.

A Workflow for the identification of AhR regulated genes among signatures.

B Expression heatmap for the for the most highly enriched genes ($n = 50$; Appendix Fig S2B and C) from BRAFi-sensitive/proliferation/differentiation (green) or BRAFi resistance/invasion/dedifferentiation signatures (red) in SK28 BRAFi-sensitive or resistant cell lines KO or not for AhR by CRISPR/Cas9 and rescued by the constitute active form of AhR (KO CA-AhR; GSE166617), BRAFi-sensitive or BRAFi-resistant melanoma cell lines from the Cancer Cell Line Encyclopedia (CCLE; Barretina *et al*, 2012) and melanoma cell lines from the Graeber datasets (Tsoi *et al*, 2018). The enrichment rank for the signatures are available in Dataset EV1.

C Expression heatmap for the most highly enriched genes ($n = 50$) in SK28R WT, KO, and KO CA-AhR cells.

D Expression heatmap for the median expression of the most highly enriched genes after GSEA for 9 BRAFi-treated melanoma patients during melanoma progression (pre-treatment, during disease progression DP1 $n = 9$, DP2 $n = 9$, DP3 $n = 5$; GSE65185 (Hugo *et al*, 2015)). Clinical data are available in supplemental Table S1 from Hugo *et al* (2015). The scale corresponds to the Z scores.

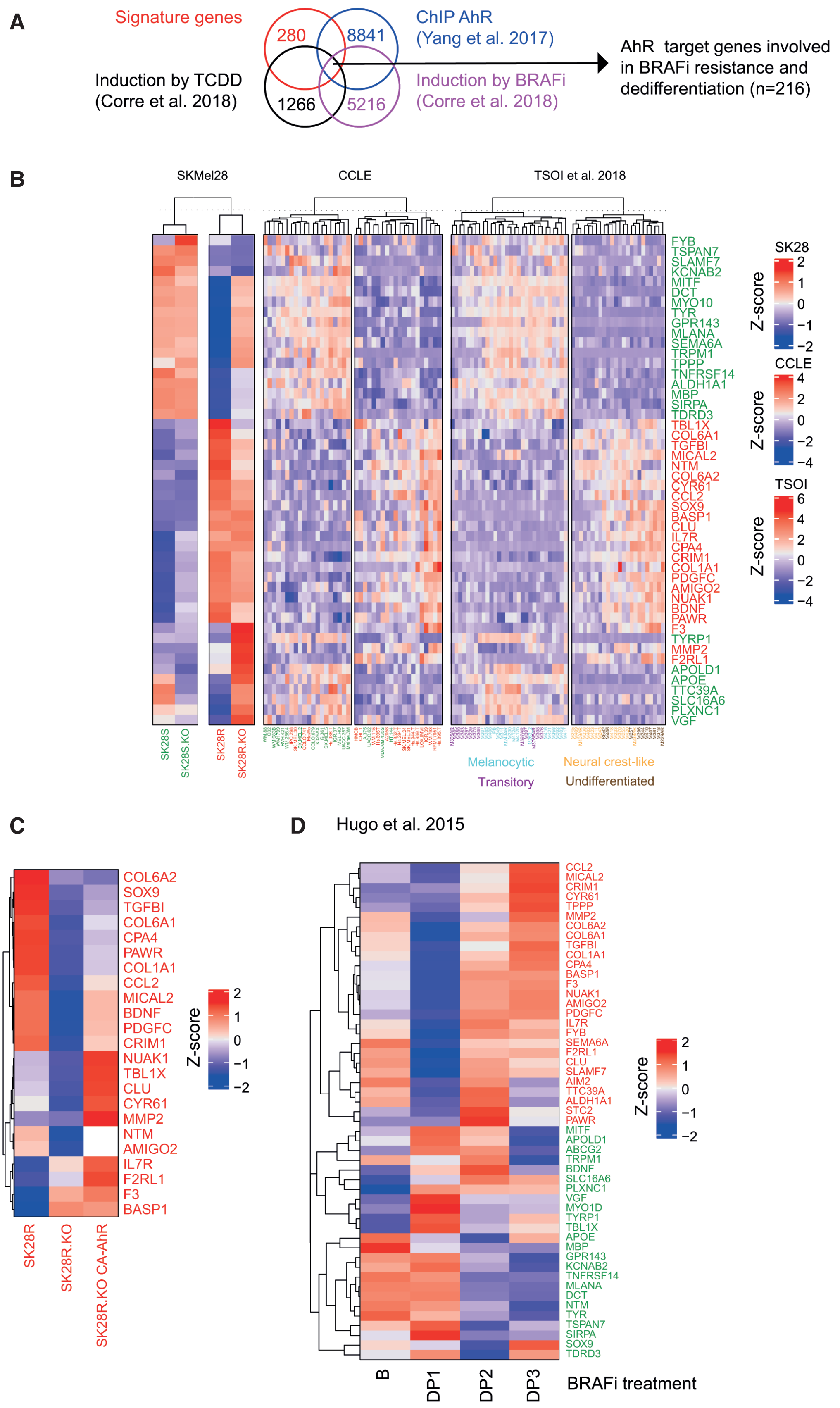

Figure 3.

©2022 The Authors

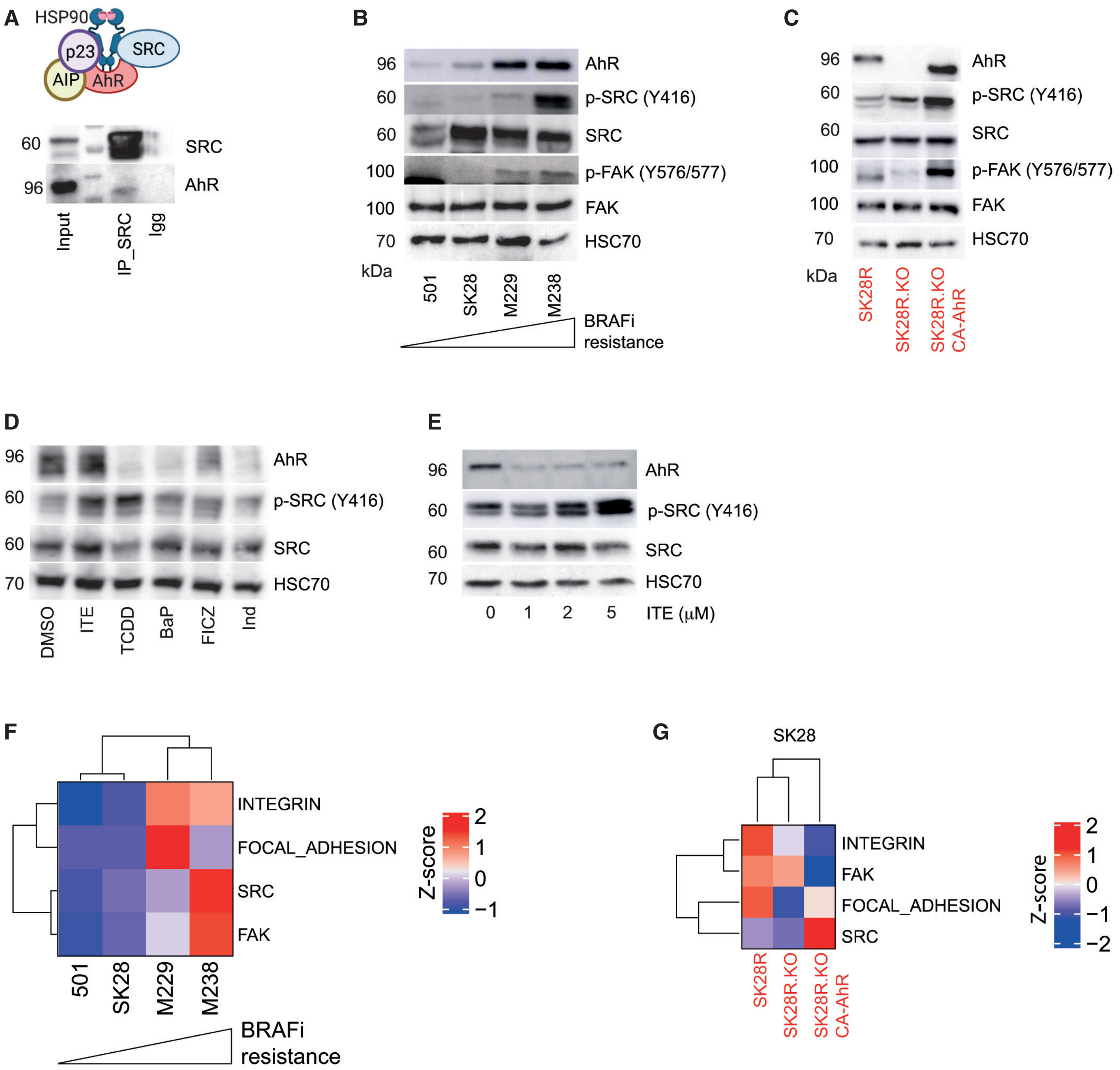

Figure 4. Canonical activation of AhR induces the activation of SRC/FAK associated with an increased activation of the focal adhesion pathway in BRAFi-resistant and dedifferentiated melanoma cell lines.

A At the basal level, AhR is located at the membrane layer in a protein complex that includes HSP90, p23, XAP, and SRC. Immunoprecipitation of SRC was performed with specific antibodies and SRC/AhR interaction analyzed by western blotting.

B Protein levels of the AhR, p-SRC (Y416), SRC, p-FAK (Y576/577), and FAK in the four different melanoma cell lines ($n = 3$). The level of BRAFi resistance corresponds to our measure of IC50 (Vemurafenib) for the different cell lines 501Mel (0.23 µM), SKMel28 (0.29 µM), M229 (0.89 µM), and M238 (2.16 µM). These cell lines correspond to the sensitive parental cells.

C Protein levels of the AhR, p-SRC (Y416), SRC, p-FAK (Y576/577), and FAK in the SK28R cell line KO or not for AhR by CRISPR/Cas9 or after rescue with the activated-form of the AhR (CA-AhR).

D Protein levels of AhR, p-SRC (Y416), and SRC, in the SK28R cell line after treatment with different AhR ligands for 24 h (5 µM BaP, 5 µM indirubin, 5 µM ITE, 10 nM TCDD, and 5 µM FICZ).

E Protein levels of AhR, p-SRC (Y416), and SRC, in the SK28R cell line after 24 h of treatment with increasing doses of ITE.

F Expression heatmap for SRC, FAK, focal-adhesion, and integrin signatures in four different BRAFi-sensitive and resistant melanoma cell lines.

G Expression heatmap for SRC, FAK, focal-adhesion, and integrin signatures in the SK28R cell line KO or not for AhR by CRISPR/Cas9 or after rescue with the activated-form of the AhR (CA-AhR). The scale corresponds to the Z scores.

Source data are available online for this figure.

©2022 The Authors

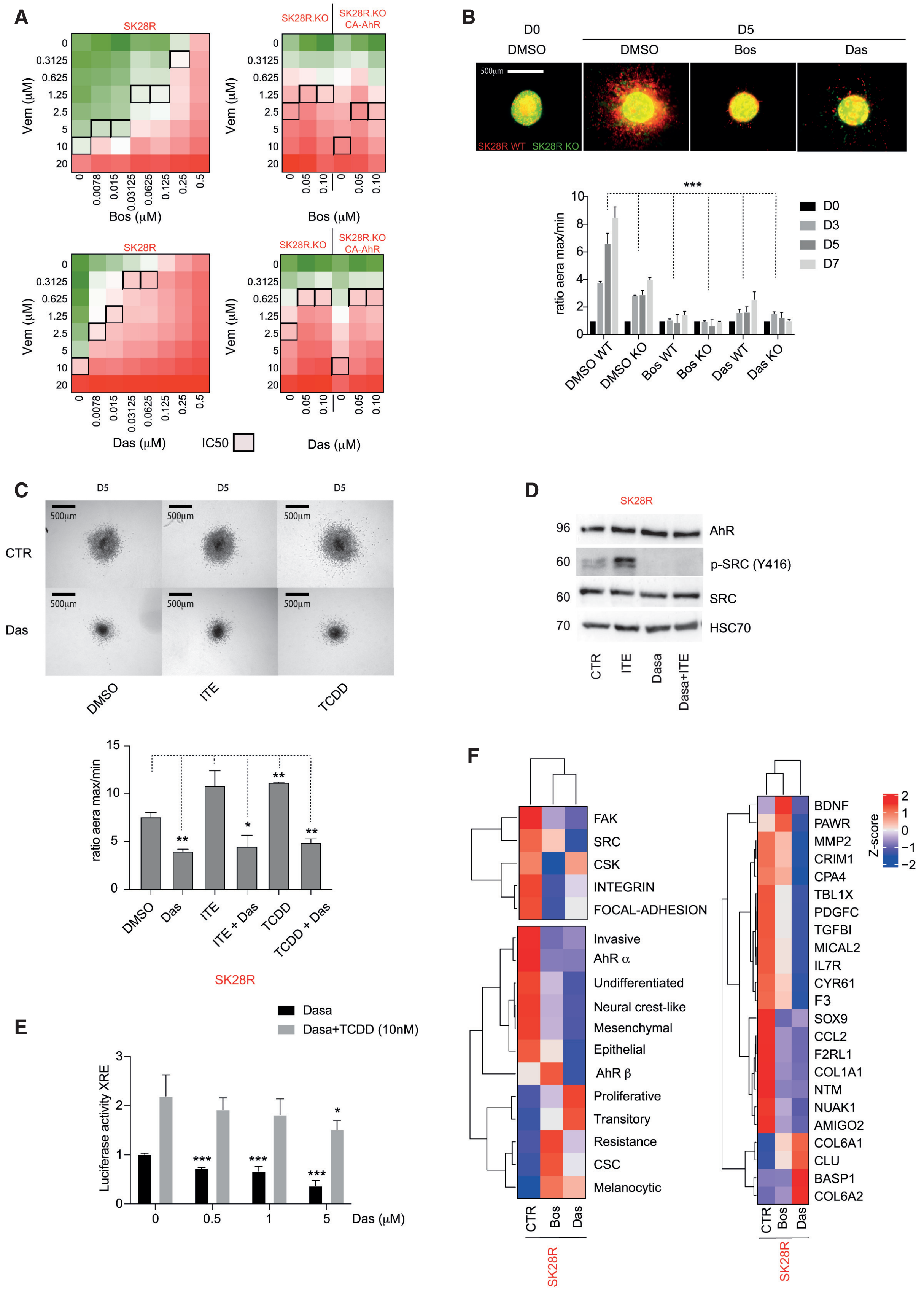

Figure 5.

©2022 The Authors

Figure 5. Inhibition of the SRC pathway increases BRAFi sensitivity and prevents invasive phenotype of melanoma cells.

A Heatmap of the percentage of cell viability in SKR (left) or SKR KO or SKR KO CA-AhR (right) cells treated with a combination of increasing doses of vemurafenib (y) and bosutinib or dasatinib (1 μM). IC50 values are represented by black squares.

B Three-dimensional spheroid growth of a 50%/50% mix of SK28R WT (in red) and KO cells (in green) over 5 days. Cells were treated or not with SRC inhibitors: bosutinib (1 μM) or dasatinib (1 μM) every 2 days ($n = 3$ independent technical experiments, mean ± s.d.). Comparisons with control (DMSO) were performed using unpaired t-tests with the Sidak–Bonferroni method, $P < 0.001$ ***.

C Three-dimensional spheroid growth of SK28R WT (in red) and KO cells (in green) over 5 days. Cells were treated or not with dasatinib (1 μM) in combination with ITE (5 μM) or TCDD (20 nM) every 2 days ($n = 3$ independent technical experiments, mean ± s.d.). Comparisons with control (DMSO) were performed using unpaired t-tests with the Sidak–Bonferroni method, $P < 0.05$ *, $P < 0.01$ **. Comparisons were performed using unpaired t-tests with the Sidak–Bonferroni method.

D Protein levels of AhR, p-SRC (Y416), and SRC in the SK28R cell line after 24 h of treatment with ITE (5 μM) or SRC inhibitor (dasatinib; 1 μM).

E Evaluation of AhR transcriptional activity related to AhR/ARNT binding sites (XRE) using p3xXRE-luciferase constructs. HaCat keratinocytes cells were exposed or not to 10 nM TCDD alone or in combination with increasing concentrations of dasatinib O/N ($n = 3$ independent technical experiments, mean ± s.d.). Comparisons with control (0) were performed using unpaired t-tests with the Sidak–Bonferroni method, $P < 0.05$ *, $P < 0.001$ ***.

F Expression heatmap of the median of gene expression for the various signatures (left) and AhR target genes (right) from RNAseq datasets for SK28R cells treated or not for 24 h with SRC inhibitors: bosutinib (Bos, 1 μM) or dasatinib (Das, 1 μM). The scale corresponds to the Z scores.

Source data are available online for this figure.

switch toward distinct drug-tolerant transcriptional states of melanoma cells state (Rambow *et al*, 2018; Tsoi *et al*, 2018).

Here, we identified a dual role of AhR transcription factor in the control of cell plasticity and phenotypic change during the acquisition of BRAFi resistance. First, high level and activity of AhR mediates the invasive/dedifferentiated phenotype of melanoma through the direct regulation of the expression of many genes involved in invasion (*COL1A1*, *COL6A1*, *COL6A2*, *CYR61*, *STC2*...) (Hoek *et al*, 2008; Verfaillie *et al*, 2015) and dedifferentiation phenotypes (*CCL2*, *NTM*, *NUAK2*, *SOX9*, *ABCG2*...) (Rambow *et al*, 2018; Tsoi *et al*, 2018) (Appendix Table S2). Interestingly, the phenotype of melanoma cells lacking Mitf transcription factor was similar to those observed while AhR is highly expressed and activated (Dilshat *et al*, 2021), allowing to consider a new balance between these two transcription factors for the control of melanoma plasticity.

Second, sustained activation of AhR mediates the activation of the SRC pathway following phosphorylation of the Tyr^{416} (Y416). Together, AhR-dependent transcriptional reprogramming and SRC activation triggers the cell plasticity of BRAFi-resistant melanoma. The identification of an AhR/SRC regulation node fully supports the importance of non-genomic cell reprogramming. It also provides a strong rationale for the understanding of the role of the SRC-family in BRAFi treatment (Girotti *et al*, 2015; Close *et al*, 2020; Krayem *et al*, 2020) and allows to delineate the pathway that mediates the activation of SRC and elevated integrin/FAK observed in melanoma (Hirata *et al*, 2015).

Indeed, the crucial role of SRC in many aspects of tumor development including migration, invasion and survival has warranted the use of SRC inhibitors to disrupt these effects in several cancer types (Roskoski, 2015). In this respect, SRC inhibitors have been tested in melanoma. However, the anti-proliferative effect of SRC inhibitors alone, on melanoma cells, was minor to no effect. Importantly cytotoxicity was mainly observed in cells that did not carry BRAF oncogenic mutation (Eustace *et al*, 2008; Halaban *et al*, 2019). In accordance, clinical studies using SRCi as a single agent showed only minimal therapeutic activity in stage III/IV chemotherapy-naive unresectable melanoma (Kluger *et al*, 2011). These results contrast with those supporting the use of SRC inhibitors in resistance settings in line with the upregulation of members of the SRC-family kinases (Girotti *et al*, 2013) and downstream SRC-dependent effectors such as MCF2 and VAV1, two DBL family members identified through a genetic screen as candidate drug resistance in melanoma cells (Feddersen *et al*, 2019). SRC inhibitors were also reported to promote a differentiated state through the upregulation of Mitf expression and downstream melanocytic markers (*TYR*, *TRP1*...) via the MAPK and CREB pathways (Ku *et al*, 2019). This gives some hints of how SRC may participate in melanoma cell reprogramming. The identification herein of the AhR/SRC activation loop in BRAFi-resistant melanoma gives rationale to these studies filling an important gap to understand cell plasticity and propose innovative therapeutic regimens.

We previously showed in a preclinical PDX melanoma mice model that antagonizing AhR delayed the emergence of resistant cells (Corre *et al*, 2018). Here, using a BRAFi-resistant PDX melanoma model, we demonstrated that SRC inhibition (dasatinib) significantly controlled tumor growth and remarkably re-sensitize melanoma cells to BRAFi (dabrafenib), doubling the overall survival rate compared to BRAFi alone. This allows us to envision new therapeutic settings using SRC inhibitors to resensitize tumor cells to BRAFi and to improve therapeutic benefits with delayed relapses. The time to introduce SRCi could be determined by monitoring the presence of circulating tumor DNA (ctDNA) in liquid biopsies as an early marker of tumor progression (Calapre *et al*, 2017). The detection of the $BRAF^{V600}$ mutation could serve as the starting point to initiate co-treatment with SRCi.

Our results also underscored that AhR-dependent activation of SRC in BRAFi-resistant cells leads to the activation of FAK kinase after phosphorylation. Marin-Bejar *et al*, 2021 have recently shown that gains activity of FAK signaling is associated with the emergence of neural crest stem cell (NCSC) subpopulation in BRAFi/MEKi drug-tolerant cells known as minimal residual disease (MRD). This activation of FAK in melanoma cells is driven in part by a "paradoxical" activation of melanoma-associated fibroblasts and the induction of β1/FAK/SRC signaling (Hirata *et al*, 2015) but also after activation of GFRA2/GDNF expression and AKT activation (Marin-Bejar *et al*, 2021). Interestingly, FAK-inhibitors strongly decreased the emergence of the NCSCs in MRD lesions, and drastically delayed the onset of resistance to RAF/MEK inhibitors in preclinical PDX models. They also proposed to test combinations of both FAK and SRC inhibitors, such as dasatinib, as a more effective strategy to suppress the emergence of the NCSC population at MRD (Marin-Bejar *et al*, 2021).

In parallel, we underscored that AhR-dependent activation of SRC mediates the activation of the epidermal growth factor receptor

©2022 The Authors

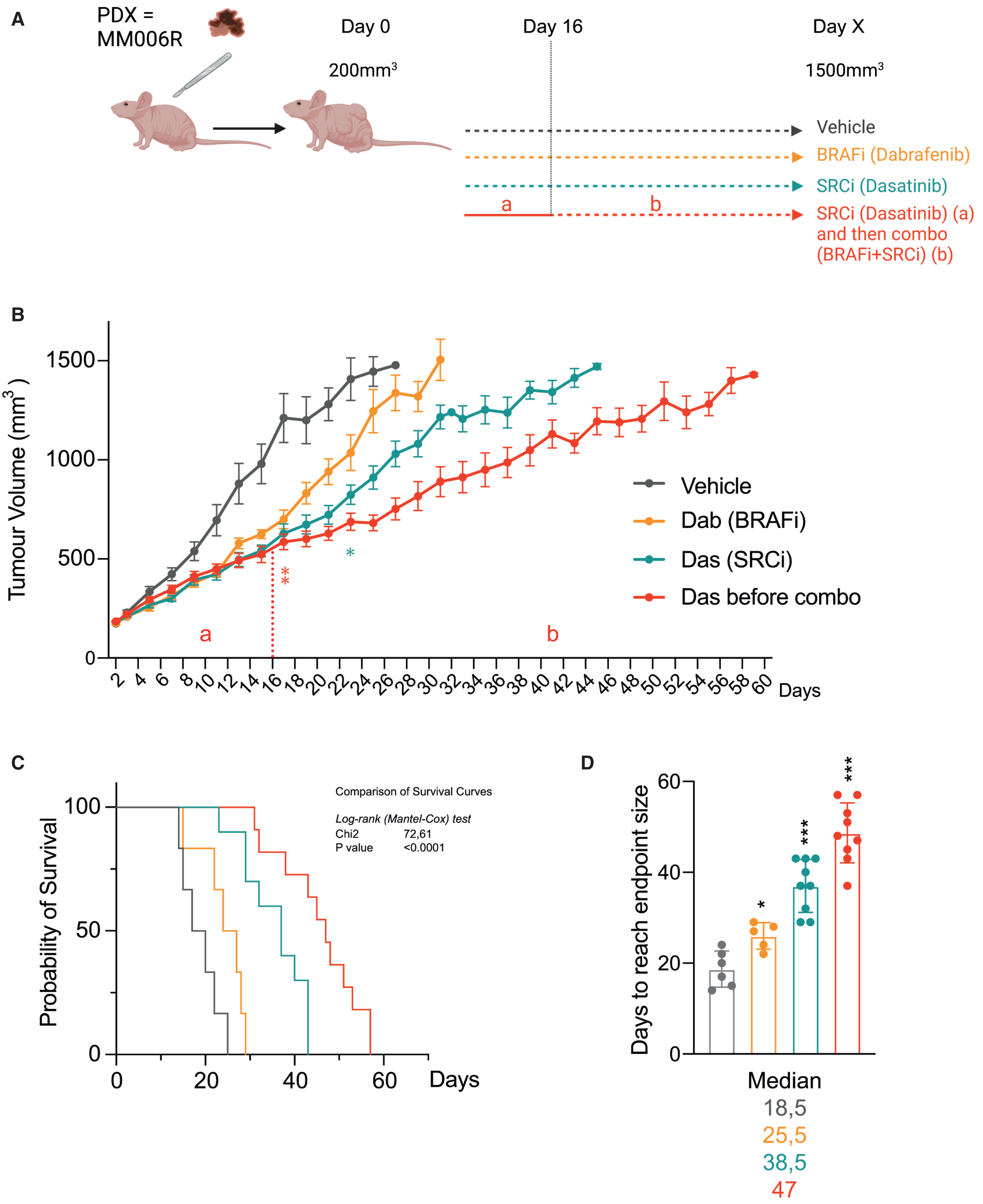

Figure 6.

©2022 The Authors

Figure 6. Inhibition of SRC sensitizes melanoma to BRAFi treatment in a PDX model.

A PDX model MEL006R (BRAFi resistant) was implanted in NMRI nude mice. Mice with tumors reaching 200 mm^3 were treated daily with vehicle (n = 6), dabrafenib alone (Dab, Biorbyt, 30 mg/kg, n = 5) dasatinib alone (Das, Selleckchem, 30 mg/kg, n = 9) or in combination dabrafenib + dasatinib (Das before combo, 30 mg/kg, n = 12).

B PDX tumor volumes were measured every 2 days until reaching 1,500 mm^3. Values correspond to the mean ± s.e.m. Statistical analysis (two-way ANOVA) has been performed between the different experiments (Das or Das before combo vs. Dab) at different times. Stars represent time from when tumor size is significantly lower than group treated with BRAFi alone (Dabrafenib) $P < 0.05$ *, $P < 0.01$ **.

C Kaplan–Meier survival curve for MEL006R mice treated with the different drugs. Comparison of survival curves have been performed using the nonparametric Log-rank (Mantel–Cox) test.

D Number of days to reach max tumor volume (endpoint point size). Values correspond to the median ± s.d. Non-parametric Mann–Whitney t-test for the different treatments was performed compared with vehicle ***$P < 0.001$.

(EGFR). Indeed, we showed that activation of SRC leads to the reactivation of the EGFR after its phosphorylation (Y845) in BRAFi-resistant melanoma cell lines (Fig EV5A–C). Such SRC-AhR cross talk has been previously described to mediate EGFR phosphorylation in colon and lung cancer cells (Xie *et al*, 2012; Ye *et al*, 2018) and to contribute to an aggressive phenotype in multiple human tumors (Biscardi *et al*, 1999). Accordingly, BRAFi resistance commonly correlates with a high level of EGFR expression and a poor prognosis (Luebker & Koepsell, 2019). In addition, EGFR activation after phosphorylation has been shown to be more highly associated with resistance and EMT transition (Gross *et al*, 2015), notably after reactivation of the ERK pathway. The direct role of AhR in regulating the phosphorylation of SRC (Y416) and EGFR (Y845) may promote together the acquisition of the aggressive/invasive EMT like phenotype of BRAFi-resistant melanoma (Sato *et al*, 2003; Sato, 2013) (Synopsis). Consistent with these data, several therapeutic strategies using erlotinib or gefitinib have already been tested in preclinical studies to increase the sensitivity to BRAFi and decrease invasive abilities in melanoma (Sun *et al*, 2014; Notarangelo *et al*, 2017; Kenessey *et al*, 2018; Simiczyjew *et al*, 2019) (Fig EV5D).

Together these results identify the central role of the AhR/SRC axis in supporting nongenetic cell reprogramming of melanoma cells exposed to targeted therapy. The AhR/SRC axis orchestrates cell plasticity, constituting an important therapeutic vulnerability. It warrants future clinical studies targeting the AhR-dependent SRC/FAK/EGFR axis in combination with BRAFi/MEKi double blockade to re-sensitize melanoma to standard melanoma treatment and counteract resistance.

Materials and Methods

Cell culture and reagents

Human melanoma cell lines (SK28, 501Mel, M229, and M238) were grown in humidified air (37°C, 5% CO_2) in RPMI-1640 medium (Thermo Fisher Scientific, Invitrogen, Waltham, MA, USA) supplemented with 10% fetal bovine serum (Eurobio, Les Ulis, France) and 1% penicillin–streptomycin antibiotics (Thermo Fisher Scientific). SK28 (S + R) cells were obtained from J.C Marine at the VIB Center for Cancer Biology, VIB, Leuven, Belgium. M229 cells were obtained from Graeber's lab at the UCLA Molecular Biology Institute, Los Angeles, CA, USA. 501Mel cells (S) were obtained from the ATCC and 501Mel BRAFi-resistant cells (R) were obtained after 3 months of treatment with Vem (1 μM every 2 days). No difference of proliferation has been observed between resistant cells and parental ones. Melanoma cells were grown in the absence of BRAFi treatment but challenged every 2 weeks with BRAFi at the IC50 dose of the sensitive corresponding cells to maintain a selective pressure. HEK 293T cells were obtained from the ATCC and grown in humidified air (37°C, 5% CO_2) in DMEM medium (Thermo Fisher Scientific). All cell lines were routinely tested for mycoplasma contamination.

Reagents

- *AhR ligands*: 2,3,7,8-tetrachlorodibenzo-p-dioxine (TCDD; Sigma Aldrich, St Louis, MO, USA, 48599), 2-(1' H-indole-3′-carbonyl)-thiazole-4-carboxylic acid methyl ester (ITE; Medchem Express, Monmouth Junction, NJ, USA HY-19317), benzo-a-pyrene (BaP; Sigma Aldrich, B1760), indirubin (Selleckchem, Houston, TX, USA, S2386), FICZ (6-formylindolo [3,2-b]carbazole; Medchem Express, HY-12451), and CH-223191 (Selleckchem, S7711).
- *BRAF inhibitors*: vemurafenib (Vem, PLX4032; Selleckchem, S1267), dabrafenib (Dab, GSK2118436; Selleckchem, S2807), and encorafenib (LGX818; Selleckchem, S7108).
- *SRC inhibitors*: dasatinib (Selleckchem S1021) and bosutinib (SKI-606; Selleckchem, S1014).
- *EGFR inhibitors*: gefitinib (Selleckchem S1025) and erlotinib (Selleckchem S7786).
- *Hsp90 inhibitor*: NVP-HSP990 (Selleckchem S7097).
- *DMSO* – Sigma-Aldrich (D8418).

CRISPR/Cas9 experiments

The AhR knockout was performed using CRISPR/Cas9 methodology. The guide sequence targeting AhR (Sigma-Genosys, St Louis, MO, USA) was cloned into the GeneArt CRISPR Nuclease vector according to the manufacturer's instructions (Life Technologies, Saint-Aubin, France). Next, 501Mel or SK28 cells were transfected with the vectors and the cells seeded 2 days later in 96-well plates at 0.5 cells/well for single-cell clonal expansion. The clones of interest were validated by DNA-sequencing, western blot analysis, and RT-qPCR.

CRISPR-SAM experiments

Lentiviral infections were used to obtain stable cell lines. Lentiviral production was performed as recommended (http://tronolab.epfl.ch) using HEK 293T cells, psPAX2 (Addgene, Cambridge, MA, USA #12260) and pVSV-G (Addgene, #14888) plasmids, and the required vectors. Infections were performed overnight. To generate 501Mel

©2022 The Authors

cells individually overexpressing *AhR*, 501Mel cells were first transduced to stably express dCAS-VP64 (Addgene, #61425) and MS2-P65-HSF1 (Addgene, #61426) before transduction with specific AhR sgRNAs (from Supplementary Table S of Gautron *et al*, 2021). Infected cells were selected using zeocin (600 µg/ml, 5 days). Lentivirus was manipulated in the biosafety level 3 containment laboratory core facility of the Biology and Health Federative Research Structure of Rennes (Biosit).

Overexpression of constitutively active form of AhR

The open reading frame for CA-AhR (McGuire *et al*, 2001) was cloned into pLL3.7 using Gblocks (IDT DNA, Coralville, IA, USA) and Gibson Assembly® Master Mix following the manufacturer's recommendations (NEB, Ipswich, MA, USA). Lentiviral infection was used to obtain stable cell lines. Lentiviral production was performed as recommended (http://tronolab.epfl.ch) using HEK 293T cells, pVSV-G (Addgene #14888), pRSV-Rev (Addgene #12253), pMDLg/pRRE (Addgene #12251), and a target vector. Infections were performed overnight. To generate SK28 cells overexpressing constitutive *AhR*, cells were infected to stably express the pLL3.7-AhR-CA vector (pLL3.7 backbone, Addgene #11795). Infected cells were selected twice by fluorescence-activated cell sorting using GFP detection.

Cell density evaluation

Cell density was assessed using a methylene blue colorimetric assay. Briefly, cells were fixed for at least 30 min in 95% ethanol. Following ethanol removal, the fixed cells were dried and stained for 30 min with 1% methylene blue dye in borate buffer. After four washes with tap water, 100 µl 0.1 N HCl was added to each well. Plates were then analyzed with a spectrophotometer at 620 nm.

Wound healing migration assay

Briefly, cells were grown until confluent in 2-well silicone inserts (Ibidi®, Germany) placed in 12-well tissue culture dishes. The cell culture inserts were removed after 1 day. Afterward, the plates were washed with PBS and incubated at 37°C in fresh RPMI-1640 medium (Gibco BRL, Invitrogen, Paisley, UK) supplemented with 10% fetal bovine serum (Eurobio) and 1% penicillin–streptomycin antibiotics (Gibco, Invitrogen), either naive or in the presence of vehicle (DMSO) or CH-223191 (5 µM). The wound was photographed with an inverted microscope at 5× magnification using an Axio Vert.A1 inverted microscope (Carl Zeiss). Wound closure was determined by measuring the distance between the edges of the wound at time 0 and 15 h using ImageJ (Fiji). Quantification of the distance migrated by the cells was performed as follows:

$$D = (\text{size of the wound at} \, t = 0\text{h} - \text{size of the wound at} \, t = 15\text{h}).$$

Spheroid formation assay

The spheroid formation assay was performed as previously described. Cells (20,000 cells/ml) were plated in 24-well plates coated with 1.5% agarose in complete RPMI medium and concentrated in the center by circular agitation. After 2 days, spheroids were recovered for inclusion in an extracellular matrix of collagen (100 µl; final concentration = 2 mg/ml in buffer (0.01 N acetic acid; neutralization buffer: 33 mM Hepes pH 7.4, 0.37% sodium bicarbonate, 0.03 N NaOH; 1× MEM)) in 24 well-plates coated with 1.5% agarose. Spheroids were maintained in complete medium with or without CH-223191 (5 µM) and images of the spheroids captured over several days (0–4 days) using an Axio Vert.A1 inverted microscope (Carl Zeiss) at 5× magnification. Invasion capacity was evaluated by determining the ratio between the maximum and initial diameter of the spheroid.

RNA extraction and RT-qPCR expression

RNA extraction & RT-qPCR expression was performed as previously described (Corre *et al*, 2018). The sequences of the primers used for the RT-qPCR experiments are available in Appendix Table S4.

Western blotting

Harvested cells were solubilized as previously described. Protein samples were denatured at 95°C, resolved by SDS-PAGE, and transferred onto Hybond™-C Extra nitrocellulose membranes (Amersham Biosciences, Bucks, UK). Membranes were probed with the appropriate antibodies Appendix Table S5 and the signals detected using a Fujifilm LAS-3000 Imager (Fuji Photo Film, Tokyo, Japan). Primary antibody information is available in Appendix Table S5. Horseradish-peroxidase-conjugated secondary antibodies were purchased from Jackson ImmunoResearch (Suffolk, UK) and used at a dilution of 1:10,000.

Immunoprecipitation

SKMel28-resistant cells were collected and lysed with cell lysis buffer (20 mM Tris–HCl, pH 8, 150 mM NaCl, 0.5 M EDTA) for 30 min on ice. The supernatant was incubated with 40 µl of protein G magnetic beads and 10 µl of SRC antibody (2108, Cell Signaling) or of normal rabbit IgG (sc2027, Santa Cruz Biotechnology) overnight at 4°C under rotation. Beads were washed with lysis buffer and eluted with 30 µl of Laemmli. Immunoprecipitates were analyzed by western blotting with appropriate antibody.

Luciferase activity

HaCat keratinocytes (2.10^5) were cultured in 12-well plates and transfected with the pGL3-XRE3-Luc construct carrying firefly luciferase. Transient transfection of cells was performed as previously described (Corre *et al*, 2018). After a 24-h period, cells were exposed to TCDD (10 nM) in the presence of increasing concentration of dasatinib (0.5–5 µM) overnight. Luciferase assays were then performed using a Promega kit according to the manufacturer's instructions. Data are expressed in arbitrary units relative to the value of luciferase activity levels found in DMSO-exposed cells, arbitrarily set to 1 arbitrary unit (a.u.). Firefly luciferase activity was normalized to protein content using the Bicinchoninic Acid Kit from Sigma-Aldrich® and measured using a luminometer CLARIOStar (BMG Labtech).

Proximity ligation assay

The proximity ligation assay was used to visualize AhR/SRC complexes in SK28 cells. The cells, grown on glass coverslips, were fixed

©2022 The Authors

with 4% PFA in 0.1 M phosphate buffer (15735-60S, Electron Microscopy Sciences) for 15 min at RT and PLA performed using the Duolink® *in Situ* detection Reagent Orange (DUO92007), Duolink® *in Situ* PLA® Probe Anti-Mouse PLUS (DUO92001), and Duolink® *in Situ* PLA® Probe Anti-Rabbit MINUS (DUO92005), SIGMA kits according to the manufacturer's protocol. After blocking, the reaction was performed with the primary antibodies: mouse anti-AhR (C20, 1/100) and rabbit anti-SRC (1C12, 1/100). Following the ligation and amplification steps, the coverslips were immobilized on microscopic slides using mounting medium containing DAPI. The ligation step was omitted in the control. Imaging analysis was carried out using a delta vision system (Applied Precision). The number of foci was quantified for at least 30 cells.

Patient-derived xenografts

In collaboration with TRACE and after approval by the University Hospital KU Leuven Medical Ethical Committee (S54185) and written informed consent from the patient, PDX model MEL006R (BRAFi resistant) was established from an in-transit metastasis resected as part of standard-of-care melanoma treatment at the University Hospital KU Leuven. The procedures involving mice were performed in accordance with the guidelines of the IACUC and KU Leuven and carried out within the context of approved project applications P147/2012, P038/2015 and P098/2015. Fresh tumor tissue was collected in transport medium (RPMI1640 medium supplemented with penicillin/streptomycin and amphotericin B). Tumor fragments were subsequently rinsed in phosphate-buffered saline supplemented with penicillin/streptomycin and amphotericin B and cut into small pieces of approximately $3 \times 3 \times 3$ mm^3. Tumor pieces were implanted in the interscapular fat pad of female SCID-beige mice (Taconic). After reaching generation 4 (F4), tumor fragments were implanted in the interscapular fat pad of female NMRI nude mice (8 weeks, Taconic). Ketamine, medetomidine and buprenorphine were used for anesthesia. Because tumor growth of the BRAFi-resistant PDX model (Mel006R) is very fast, this model is probably not appropriate to study the mechanisms of invasion and metastasis at least in the time window analyzed (20–60 days).

Pharmacologic treatment of mice

Mice with tumors reaching 200–300 mm^3 were treated via daily oral gavage. Dabrafenib (Biorbyt) and/or dasatinib (Selleckchem) were dissolved in DMSO at a concentration of 30 mg/ml respectively, aliquoted and stored at −80°C. Each day a new aliquot was diluted 1:10 with phosphate-buffered saline and mice were treated with a dose of 30 mg/kg for dabrafenib alone, with dasatinib alone (30 mg/kg) or with the combination dabrafenib + dasatinib (30 mg/kg each) after a pretreatment with dasatinib for 16 days. Tumor volume was monitored with a caliper and calculated using the following formula: $V = (\pi/6)*length*width*height$. The endpoint of the experiment corresponds when tumor volume reaches 1500 mm^3 according to ethical statements.

RNA-Seq

Total RNA was extracted from BRAFi-sensitive or resistant SK28, Mel501, and M229, cells before and after knockout out of AhR using the NucleoSpin RNA kit (Macherey Nagel, Düren, Germany). A complementary DNA library was prepared and sequencing performed according to the Illumina standard protocol by Beijing Novel Bioinformatics Co., Ltd. (https://en.novogene.com/). RNAseq was performed in collaboration with Novogene (Beijing, China). Libraries were generated from 500 ng total RNA using a Truseq Stranded mRNA kit (Illumina). The concentration of the library was first determined using a Qubit2.0 fluorimter and then diluted to 1 ng/μl. The size of the insert was checked using an Agilent bioanalyzer and further quantified by qPCR (library concentration > 2 nM). An aliquot (0.5 nM) of the pool was loaded on a high-output flow cell and sequenced on a NovaSeq 6000 instrument (Illumina) with 2×150 bp paired-end chemistry in two runs. Reads were aligned to human genome release hg38 using HISAT2 V2.0.5 with default parameters. Quantification of the expressed genes was performed using CUFFDIFF v2.2.1. The quality of the RNA-Seq count data was assessed using the Novogene standard protocol. The RNA-Seq data presented in this article was submitted to the Gene Expression Omnibus database (http://www.ncbi.nlm.nih.gov/geo/) under the accession number (GSE166617).

Data mining

TCGA/SKCM RNAseq data were analyzed using the OncoLnc portal [http://www.oncolnc.org] (Anaya, 2016). The raw data count matrix, composed of 454 samples (from SKCM melanoma cohort), was downloaded from the OncoLnc portal for the various transcriptional signatures. Expression heatmaps of differentially expressed genes between samples were obtained based on a log2 fold change using the ComplexHeatmap 2.0.0 (Gu *et al*, 2016) package in R/Bioconductor. Cluster-specific gene rankings were obtained by contrasting the samples with the rest. Cell density curves for the available melanoma cell lines were established using GraphPad PRISM 9.0® to establish the IC50 for the various treatments.

The raw data count matrices from the RNA seq data were obtained in GEO database for previous experiments on melanoma

The paper explained

Problem
Despite the considerable improvement made in the management of patients with metastatic BRAF^{V600} mutated melanoma, the vast majority of patients treated with BRAFi experience disease progression. Understanding the resistance mechanisms that support tumor progression is mandatory to overcome this process and to propose new therapeutic options.

Results
We show here that the ligand-activated transcription factor AhR drives cell plasticity, switching non-invasive and BRAFi-sensitive melanoma cells into invasive and resistant cells. AhR operates through genomic reprogramming and through the activation of the SRC kinase pathway. *In vitro* and *in vivo* use of SRC inhibitors in combination with BRAFi resensitize resistant melanoma cells to BRAFi treatment.

Impact
This study shows the AhR/SRC axis constitutes a therapeutic vulnerability in BRAFi-resistant melanoma, opening new therapeutic perspectives for BRAFi-resistant patients.

©2022 The Authors

cell lines (Barretina *et al*, 2012) GSE36134 [https://www.ncbi.nlm.nih.gov/gds/?term=GSE36134] (sensitive or resistant to PLX470; IC50 values for PLX4720 were obtained from Supplementary Table S7 of Barretina *et al*, 2012), BRAFi^{-} or BRAFi^{+}MEKi-resistant cell lines GSE75299 [https://www.ncbi.nlm.nih.gov/gds/?term=GSE752099 (Song *et al*, 2017)] and GSE80829 [https://www.ncbi.nlm.nih.gov/gds/?term=GSE80829 (Tsoi *et al*, 2018)] and GSE110054 [https://www.ncbi.nlm.nih.gov/geo/query/acc.cgi?acc=GSE110054 (Tsoi *et al*, 2018)], BRAFi-treated melanoma patients GSE65185 [https://www.ncbi.nlm.nih.gov/gds/?term=GSE65185 (Hugo *et al*, 2015)] and melanoma cell lines (proliferative or invasive) GSE60664 [https://www.ncbi.nlm.nih.gov/gds/?term=GSE60664 (Verfaillie *et al*, 2015)].

Analysis of the RNAseq dataset from the GDSC (Sanger/Massachusetts General Hospital Genomics of Drug Sensitivity in Cancer; Yang *et al*, 2013) was performed and recovered from the CellMinerCDB webtool (https://discover.nci.nih.gov/cellminercdb; Reinhold *et al*, 2012). CellMinerCDB is an interactive web application that simplifies access to and exploration of cancer cell line pharmacogenomic data from different sources. This webtool allows the comparison of molecular and/or drug response patterns across sets of cell lines to search for possible associations. Pearson's correlations with the reported p-values (not adjusted for multiple comparisons) between AhR expression (Appendix Fig S7) and drug activity (297 compounds) were recovered for various cancer cell lines ($n = 1{,}080$).

Statistics

Data are presented as the mean ± SD, unless otherwise specified, and differences were considered significant for a P value < 0.05. Comparisons between groups normalized to a control were carried out using a two-tailed *t*-test, with the Holm–Sidak multiple comparisons test when more than two groups are compared with the same control condition. Overall survival was estimated using the Kaplan–Meier method. Univariate analysis using the Cox regression model was performed to estimate the hazard ratios (HRs) and 95% confidence intervals (CI). All statistical analyses were performed using GraphPad (PRISM9.0®; La Jolla, CA, USA).

Data availability

The datasets generated during and/or analyzed during the current study are available from the corresponding author on reasonable request. RNA-Seq data: Gene Expression Omnibus GSE166617 (https://www.ncbi.nlm.nih.gov/geo/query/acc.cgi?acc=GSE166617).

Expanded View for this article is available online.

Acknowledgements
The authors would like to thank the Gene Expression and Oncogenesis team for helpful discussions and technical and administrative support (Anne-Gaëlle Rio), CNRS UMR6290, and the Rennes FHU CAMIn team. The authors acknowledge the SFR Biosit core facilities of Rennes University, along with the cell imaging ImPACcell (Rémy Le Guevel) and Microscopy Rennes Imaging Center (MRIC) platforms. The authors thank the staff of the KULeuven PDX platform (TRACE) for the PDX experiments. Trace staff are supported by Stichting Tegen Kanker grant 2016-054. This study received financial support from the following: AVIESAN plan Cancer (ENV201308 and ENV201603) Association Contre le Cancer (ARC) (No. PGA1*20160203868); Ligue National Contre le Cancer (LNCC) Départements du Grand-Ouest; FHU CAMIn-CHU Rennes, Région Bretagne; University of Rennes 1; CNRS; and Inserm. NT is a recipient of a doctoral fellowship from the French Ministry of Research. AP is a recipient of an (Institut National du Cancer) INCa grant.

Author contributions
Anaïs Paris: Formal analysis; investigation; methodology. **Nina Tardif:** Investigation; methodology. **Francesca M Baietti:** Investigation. **Cyrille Berra:** Investigation. **Héloïse M Leclair:** Investigation. **Eleonora Leucci:** Investigation. **Marie-Dominique Galibert:** Supervision; funding acquisition; writing – original draft; project administration; writing – review and editing. **Sébastien Corre:** Conceptualization; resources; formal analysis; supervision; funding acquisition; validation; investigation; writing – original draft; project administration; writing – review and editing.

Disclosure and competing interests statement
The authors declare that they have no conflict of interest.

References

Anaya J (2016) OncoLnc: linking TCGA survival data to mRNAs, miRNAs, and lncRNAs. *PeerJ Comput Sci* 2: e67

Arozarena I, Wellbrock C (2017) Overcoming resistance to BRAF inhibitors. *Ann Transl Med* 5: 387

Bailey MH, Tokheim C, Porta-Pardo E, Sengupta S, Bertrand D, Weerasinghe A, Colaprico A, Wendl MC, Kim J, Reardon B *et al* (2018) Comprehensive characterization of cancer driver genes and mutations. *Cell* 173: 371–385

Barretina J, Caponigro G, Stransky N, Venkatesan K, Margolin AA, Kim S, Wilson CJ, Lehár J, Kryukov GV, Sonkin D *et al* (2012) The cancer cell line encyclopedia enables predictive modelling of anticancer drug sensitivity. *Nature* 483: 603–607

Berger MF, Mardis ER (2018) The emerging clinical relevance of genomics in cancer medicine. *Nat Rev Clin Oncol* 15: 353–365

Biscardi JS, Maa M-C, Tice DA, Cox ME, Leu T-H, Parsons SJ (1999) c-Src-mediated phosphorylation of the epidermal growth factor receptor on Tyr845 and Tyr1101 is associated with modulation of receptor function. *J Biol Chem* 274: 8335–8343

Bollag G, Hirth P, Tsai J, Zhang J, Ibrahim PN, Cho H, Spevak W, Zhang C, Zhang Y, Habets G *et al* (2010) Clinical efficacy of a RAF inhibitor needs broad target blockade in BRAF-mutant melanoma. *Nature* 467: 596–599

Calapre L, Warburton L, Millward M, Ziman M, Gray ES (2017) Circulating tumour DNA (ctDNA) as a liquid biopsy for melanoma. *Cancer Lett* 404: 62–69

Carreira S, Goodall J, Denat L, Rodriguez M, Nuciforo P, Hoek KS, Testori A, Larue L, Goding CR (2006) Mitf regulation of Dia1 controls melanoma proliferation and invasiveness. *Genes Dev* 20: 3426–3439

Cheli Y, Giuliano S, Fenouille N, Allegra M, Hofman V, Hofman P, Bahadoran P, Lacour J-P, Tartare-Deckert S, Bertolotto C *et al* (2012) Hypoxia and MITF control metastatic behaviour in mouse and human melanoma cells. *Oncogene* 31: 2461–2470

Close DA, Kirkwood JM, Fecek RJ, Storkus WJ, Johnston PA (2020) Unbiased high-throughput drug combination pilot screening identifies synergistic drug combinations effective against patient-derived and drug-resistant melanoma cell lines. *SLAS Discov* 26: 712–729

 ©2022 The Authors

Corre S, Tardif N, Mouchet N, Leclair HM, Boussemart L, Gautron A, Bachelot L, Perrot A, Soshilov A, Rogiers A *et al* (2018) Sustained activation of the aryl hydrocarbon receptor transcription factor promotes resistance to BRAF-inhibitors in melanoma. *Nat Commun* 9: 4775

Cox MB, Miller CA (2004) Cooperation of heat shock protein 90 and p23 in aryl hydrocarbon receptor signaling. *Cell Stress Chaperones* 9: 4–20

Czarnecka AM, Bartnik E, Fiedorowicz M, Rutkowski P (2020) Targeted therapy in melanoma and mechanisms of resistance. *Int J Mol Sci* 21: 4576

Daud A, Gill J, Kamra S, Chen L, Ahuja A (2017) Indirect treatment comparison of dabrafenib plus trametinib versus vemurafenib plus cobimetinib in previously untreated metastatic melanoma patients. *J Hematol Oncol* 10: 3

Davies H, Bignell GR, Cox C, Stephens P, Edkins S, Clegg S, Teague J, Woffendin H, Garnett MJ, Bottomley W *et al* (2002) Mutations of the BRAF gene in human cancer. *Nature* 417: 949–954

Denison MS, Pandini A, Nagy SR, Baldwin EP, Bonati L (2002) Ligand binding and activation of the ah receptor. *Chem Biol Interact* 141: 3–24

Dilshat R, Fock V, Kenny C, Gerritsen I, Lasseur RMJ, Travnickova J, Eichhoff OM, Cerny P, Möller K, Sigurbjörnsdóttir S *et al* (2021) MITF reprograms the extracellular matrix and focal adhesion in melanoma. *eLife* 10: e63093

Dummer R, Ascierto PA, Gogas HJ, Arance A, Mandala M, Liszkay G, Garbe C, Schadendorf D, Krajsova I, Gutzmer R *et al* (2018) Encorafenib plus binimetinib versus vemurafenib or encorafenib in patients with BRAF-mutant melanoma (COLUMBUS): a multicentre, open-label, randomised phase 3 trial. *Lancet Oncol* 19: 603–615

Enan E, Matsumura F (1996) Identification of c-Src as the integral component of the cytosolic ah receptor complex, transducing the signal of 2,3,7,8-tetrachlorodibenzo-p-dioxin (TCDD) through the protein phosphorylation pathway. *Biochem Pharmacol* 52: 1599–1612

Eustace AJ, Crown J, Clynes M, O'Donovan N (2008) Preclinical evaluation of dasatinib, a potent Src kinase inhibitor, in melanoma cell lines. *J Transl Med* 6: 53

Fallahi-Sichani M, Becker V, Izar B, Baker GJ, Lin J-R, Boswell SA, Shah P, Rotem A, Garraway LA, Sorger PK (2017) Adaptive resistance of melanoma cells to RAF inhibition via reversible induction of a slowly dividing de-differentiated state. *Mol Syst Biol* 13: 905

Feddersen CR, Schillo JL, Varzavand A, Vaughn HR, Wadsworth LS, Voigt AP, Zhu EY, Jennings BM, Mullen SA, Bobera J *et al* (2019) Src-dependent DBL family members drive resistance to vemurafenib in human melanoma. *Cancer Res* 79: 5074–5087

Gautron A, Bachelot L, Aubry M, Leclerc D, Quéméner AM, Corre S, Rambow F, Paris A, Tardif N, Leclair HM *et al* (2021) CRISPR screens identify tumor-promoting genes conferring melanoma cell plasticity and resistance. *EMBO Mol Med* 13: e13466

Girotti MR, Lopes F, Preece N, Niculescu-Duvaz D, Zambon A, Davies L, Whittaker S, Saturno G, Viros A, Pedersen M *et al* (2015) Paradox-breaking RAF inhibitors that also target SRC are effective in drug-resistant BRAF mutant melanoma. *Cancer Cell* 27: 85–96

Girotti MR, Pedersen M, Sanchez-Laorden B, Viros A, Turajlic S, Niculescu-Duvaz D, Zambon A, Sinclair J, Hayes A, Gore M *et al* (2013) Inhibiting EGF receptor or SRC family kinase signaling overcomes BRAF inhibitor resistance in melanoma. *Cancer Discov* 3: 158–167

Goding CR, Arnheiter H (2019) MITF—The first 25 years. *Genes Dev* 33: 983–1007

Goh CJH, Wong JH, El Farran C, Tan BX, Coffill CR, Loh Y-H, Lane D, Arumugam P (2021) Identification of pathways modulating vemurafenib resistance in melanoma cells via a genome-wide CRISPR/Cas9 screen. *G3 (Bethesda)* 11: jkaa069

Gross A, Niemetz-Rahn A, Nonnenmacher A, Tucholski J, Keilholz U, Fusi A (2015) Expression and activity of EGFR in human cutaneous melanoma cell lines and influence of vemurafenib on the EGFR pathway. *Target Oncol* 10: 77–84

Gu Z, Eils R, Schlesner M (2016) Complex heatmaps reveal patterns and correlations in multidimensional genomic data. *Bioinformatics* 32: 2847–2849

Halaban R, Bacchiocchi A, Straub R, Cao J, Sznol M, Narayan D, Allam A, Krauthammer M, Mansour TS (2019) A novel anti-melanoma SRC-family kinase inhibitor. *Oncotarget* 10: 2237–2251

Hauschild A, Grob J-J, Demidov LV, Jouary T, Gutzmer R, Millward M, Rutkowski P, Blank CU, Miller WH, Kaempgen E *et al* (2012) Dabrafenib in BRAF-mutated metastatic melanoma: a multicentre, open-label, phase 3 randomised controlled trial. *Lancet* 380: 358–365

Hirata E, Girotti MR, Viros A, Hooper S, Spencer-Dene B, Matsuda M, Larkin J, Marais R, Sahai E (2015) Intravital imaging reveals how BRAF inhibition generates drug-tolerant microenvironments with high integrin β1/FAK signaling. *Cancer Cell* 27: 574–588

Hoek KS, Eichhoff OM, Schlegel NC, Döbbeling U, Kobert N, Schaerer L, Hemmi S, Dummer R (2008) In vivo switching of human melanoma cells between proliferative and invasive states. *Cancer Res* 68: 650–656

Hugo W, Shi H, Sun L, Piva M, Song C, Kong X, Moriceau G, Hong A, Dahlman KB, Johnson DB *et al* (2015) Non-genomic and immune evolution of melanoma acquiring MAPKi resistance. *Cell* 162: 1271–1285

Kemper K, de Goeje PL, Peeper DS, van Amerongen R (2014) Phenotype switching: tumor cell plasticity as a resistance mechanism and target for therapy. *Cancer Res* 74: 5937–5941

Kenessey I, Kramer Z, István L, Cserepes MT, Garay T, Hegedűs B, Dobos J, Tímár J, Tóvári J (2018) Inhibition of epidermal growth factor receptor improves antitumor efficacy of vemurafenib in BRAF-mutant human melanoma in preclinical model. *Melanoma Res* 28: 536–546

Kluger HM, Dudek AZ, McCann C, Ritacco J, Southard N, Jilaveanu LB, Molinaro A, Sznol M (2011) A phase 2 trial of dasatinib in advanced melanoma. *Cancer* 117: 2202–2208

Kobayashi S, Boggon TJ, Dayaram T, Jänne PA, Kocher O, Meyerson M, Johnson BE, Eck MJ, Tenen DG, Halmos B (2005) EGFR mutation and resistance of non-small-cell lung cancer to gefitinib. *N Engl J Med* 352: 786–792

Koelblinger P, Thuerigen O, Dummer R (2018) Development of encorafenib for BRAF-mutated advanced melanoma. *Curr Opin Oncol* 30: 125–133

Krayem M, Aftimos P, Najem A, van den Hooven T, van den Berg A, Hovestad-Bijl L, de Wijn R, Hilhorst R, Ruijtenbeek R, Sabbah M *et al* (2020) Kinome profiling to predict sensitivity to MAPK inhibition in melanoma and to provide new insights into intrinsic and acquired mechanism of resistance. *Cancer* 12: E512

Ku K-E, Choi N, Oh S-H, Kim W-S, Suh W, Sung J-H (2019) Src inhibition induces melanogenesis in human G361 cells. *Mol Med Rep* 19: 3061–3070

Larkin J (2014) Combined vemurafenib and cobimetinib in BRAF-mutated melanoma. *N Engl J Med* 371: 1867–1876

Luebker SA, Koepsell SA (2019) Diverse mechanisms of BRAF inhibitor resistance in melanoma identified in clinical and preclinical studies. *Front Oncol* 9: 268

Marin-Bejar O, Rogiers A, Dewaele M, Femel J, Karras P, Pozniak J, Bervoets G, Van Raemdonck N, Pedri D, Swings T *et al* (2021) Evolutionary predictability of genetic versus nongenetic resistance to anticancer drugs in melanoma. *Cancer Cell* 39: 1135–1149

McGuire J, Okamoto K, Whitelaw ML, Tanaka H, Poellinger L (2001) Definition of a dioxin receptor mutant that is a constitutive activator of

transcription: delineation of overlapping repression and ligand binding functions within the PAS domain. *J Biol Chem* 276: 41841–41849

Michielin O, Atkins MB, Koon HB, Dummer R, Ascierto PA (2020) Evolving impact of long-term survival results on metastatic melanoma treatment. *J Immunother Cancer* 8: e000948

Müller J, Krijgsman O, Tsoi J, Robert L, Hugo W, Song C, Kong X, Possik PA, Cornelissen-Steijger PDM, Foppen MHG *et al* (2014) Low MITF/AXL ratio predicts early resistance to multiple targeted drugs in melanoma. *Nat Commun* 5: 5712

Nihal M, Wood GS (2016) c-CBL regulates melanoma proliferation, migration, invasion and the FAK-SRC-GRB2 nexus. *Oncotarget* 7: 53869–53880

Noguchi K, Dalton AC, Howley BV, McCall BJ, Yoshida A, Diehl JA, Howe PH (2017) Interleukin-like EMT inducer regulates partial phenotype switching in MITF-low melanoma cell lines. *PLoS ONE* 12: e0177830

Notarangelo T, Sisinni L, Condelli V, Landriscina M (2017) Dual EGFR and BRAF blockade overcomes resistance to vemurafenib in BRAF mutated thyroid carcinoma cells. *Cancer Cell Int* 17: 86

Nukaya M, Lin BC, Glover E, Moran SM, Kennedy GD, Bradfield CA (2010) The aryl hydrocarbon receptor-interacting protein (AIP) is required for dioxin-induced hepatotoxicity but not for the induction of the Cyp1a1 and Cyp1a2 genes. *J Biol Chem* 285: 35599–35605

Patel A, Sabbineni H, Clarke A, Somanath PR (2016) Novel roles of Src in cancer cell epithelial-to-mesenchymal transition, vascular permeability, microinvasion and metastasis. *Life Sci* 157: 52–61

Rambow F, Rogiers A, Marin-Bejar O, Aibar S, Femel J, Dewaele M, Karras P, Brown D, Chang YH, Debiec-Rychter M *et al* (2018) Toward minimal residual disease-directed therapy in melanoma. *Cell* 174: 843–855

Randi AS, Sanchez MS, Alvarez L, Cardozo J, Pontillo C, de Pisarev DLK (2008) Hexachlorobenzene triggers AhR translocation to the nucleus, c-Src activation and EGFR transactivation in rat liver. *Toxicol Lett* 177: 116–122

Reinhold WC, Sunshine M, Liu H, Varma S, Kohn KW, Morris J, Doroshow J, Pommier Y (2012) CellMiner: a web-based suite of genomic and pharmacologic tools to explore transcript and drug patterns in the NCI-60 cell line set. *Cancer Res* 72: 3499–3511

Rey-Barroso J, Colo GP, Alvarez-Barrientos A, Redondo-Muñoz J, Carvajal-González JM, Mulero-Navarro S, García-Pardo A, Teixidó J, Fernandez-Salguero PM (2013) The dioxin receptor controls β1 integrin activation in fibroblasts through a Cbp-Csk-Src pathway. *Cell Signal* 25: 848–859

Robert C, Karaszewska B, Schachter J, Rutkowski P, Mackiewicz A, Stroiakovski D, Lichinitser M, Dummer R, Grange F, Mortier L *et al* (2014) Improved overall survival in melanoma with combined dabrafenib and trametinib. *N Engl J Med* 372: 30–39

Rose AAN, Annis MG, Frederick DT, Biondini M, Dong Z, Kwong L, Chin L, Keler T, Hawthorne T, Watson IR *et al* (2016) MAPK pathway inhibitors sensitize BRAF-mutant melanoma to an antibody-drug conjugate targeting GPNMB. *Clin Cancer Res* 22: 6088–6098

Roskoski R (2015) Src protein-tyrosine kinase structure, mechanism, and small molecule inhibitors. *Pharmacol Res* 94: 9–25

Rossi A, Roberto M, Panebianco M, Botticelli A, Mazzuca F, Marchetti P (2019) Drug resistance of BRAF-mutant melanoma: Review of up-to-date mechanisms of action and promising targeted agents. *Eur J Pharmacol* 862: 172621

Ruffini F, Tentori L, Dorio AS, Arcelli D, D'Amati G, D'Atri S, Graziani G, Lacal PM (2013) Platelet-derived growth factor C and calpain-3 are modulators of human melanoma cell invasiveness. *Oncol Rep* 30: 2887–2896

Said AH, Hu S, Abutaleb A, Watkins T, Cheng K, Chahdi A, Kuppusamy P, Saxena N, Xie G, Raufman J-P (2017) Interacting post-muscarinic receptor signaling pathways potentiate matrix metalloproteinase-1 expression and invasion of human colon cancer cells. *Biochem J* 474: 647–665

Salama AK, Kim KB (2013) Trametinib (GSK1120212) in the treatment of melanoma. *Expert Opin Pharmacother* 14: 619–627

Sato K, Nagao T, Iwasaki T, Nishihira Y, Fukami Y (2003) Src-dependent phosphorylation of the EGF receptor Tyr-845 mediates stat-p21waf1 pathway in A431 cells. *Genes Cells* 8: 995–1003

Sato K-I (2013) Cellular functions regulated by phosphorylation of EGFR on Tyr845. *Int J Mol Sci* 14: 10761–10790

Shirley M (2018) Encorafenib and binimetinib: first global approvals. *Drugs* 78: 1277–1284

Simiczyjew A, Pietraszek-Gremplewicz K, Dratkiewicz E, Podgórska M, Matkowski R, Ziętek M, Nowak D (2019) Combination of selected MET and EGFR inhibitors decreases melanoma Cells' invasive abilities. *Front Pharmacol* 10: 1116

Smith MP, Brunton H, Rowling EJ, Ferguson J, Arozarena I, Miskolczi Z, Lee JL, Girotti MR, Marais R, Levesque MP *et al* (2016) Inhibiting drivers of non-mutational drug tolerance is a salvage strategy for targeted melanoma therapy. *Cancer Cell* 29: 270–284

Song C, Piva M, Sun L, Hong A, Moriceau G, Kong X, Zhang H, Lomeli S, Qian J, Yu CC *et al* (2017) Recurrent tumor cell-intrinsic and -extrinsic alterations during MAPKi-induced melanoma regression and early adaptation. *Cancer Discov* 7: 1248–1265

Sun C, Wang L, Huang S, Heynen GJJE, Prahallad A, Robert C, Haanen J, Blank C, Wesseling J, Willems SM *et al* (2014) Reversible and adaptive resistance to BRAF(V600E) inhibition in melanoma. *Nature* 508: 118–122

Tirosh I, Izar B, Prakadan SM, Wadsworth MH, Treacy D, Trombetta JJ, Rotem A, Rodman C, Lian C, Murphy G *et al* (2016) Dissecting the multicellular ecosystem of metastatic melanoma by single-cell RNA-seq. *Science* 352: 189–196

Tomkiewicz C, Herry L, Bui L-C, Métayer C, Bourdeloux M, Barouki R, Coumoul X (2013) The aryl hydrocarbon receptor regulates focal adhesion sites through a non-genomic FAK/Src pathway. *Oncogene* 32: 1811–1820

Tsoi J, Robert L, Paraiso K, Galvan C, Sheu KM, Lay J, Wong DJL, Atefi M, Shirazi R, Wang X *et al* (2018) Multi-stage differentiation defines melanoma subtypes with differential vulnerability to drug-induced iron-dependent oxidative stress. *Cancer Cell* 33: 890–904

Vendramin R, Katopodi V, Cinque S, Konnova A, Knezevic Z, Adnane S, Verheyden Y, Karras P, Demesmaeker E, Bosisio FM *et al* (2021) Activation of the integrated stress response confers vulnerability to mitoribosome-targeting antibiotics in melanoma. *J Exp Med* 218: e20210571

Verfaillie A, Imrichova H, Atak ZK, Dewaele M, Rambow F, Hulselmans G, Christiaens V, Svetlichnyy D, Luciani F, Van den Mooter L *et al* (2015) Decoding the regulatory landscape of melanoma reveals TEADS as regulators of the invasive cell state. *Nat Commun* 6: 6683

Vizkeleti L, Kiss T, Koroknai V, Ecsedi S, Papp O, Szasz I, Adany R, Balazs M (2017) Altered integrin expression patterns shown by microarray in human cutaneous melanoma. *Melanoma Res* 27: 180–188

Wellbrock C, Marais R (2005) Elevated expression of MITF counteracts B-RAF–stimulated melanocyte and melanoma cell proliferation. *J Cell Biol* 170: 703–708

Xie G, Peng Z, Raufman J-P (2012) Src-mediated aryl hydrocarbon and epidermal growth factor receptor cross talk stimulates colon cancer cell proliferation. *Am J Physiol Gastrointest Liver Physiol* 302: G1006–G1015

 ©2022 The Authors

Yang SY, Ahmed S, Satheesh SV, Matthews J (2018) Genome-wide mapping and analysis of aryl hydrocarbon receptor (AHR)- and aryl hydrocarbon receptor repressor (AHRR)-binding sites in human breast cancer cells. *Arch Toxicol* 92: 225–240

Yang W, Soares J, Greninger P, Edelman EJ, Lightfoot H, Forbes S, Bindal N, Beare D, Smith JA, Thompson IR *et al* (2013) Genomics of drug sensitivity in cancer (GDSC): A resource for therapeutic biomarker discovery in cancer cells. *Nucleic Acids Res* 41: D955–D961

Ye M, Zhang Y, Gao H, Xu Y, Jing P, Wu J, Zhang X, Xiong J, Dong C, Yao L *et al* (2018) Activation of the aryl hydrocarbon receptor leads to resistance to EGFR TKIs in non-small-cell lung cancer by activating Src-mediated bypass signaling. *Clin Cancer Res* 24: 1227–1239

Zhang C, Spevak W, Zhang Y, Burton EA, Ma Y, Habets G, Zhang J, Lin J, Ewing T, Matusow B *et al* (2015) RAF inhibitors that evade paradoxical MAPK pathway activation. *Nature* 526: 583–586

Zhang X, Yang L, Szeto P, Abali GK, Zhang Y, Kulkarni A, Amarasinghe K, Li J, Vergara IA, Molania R *et al* (2020) The hippo pathway oncoprotein YAP promotes melanoma cell invasion and spontaneous metastasis. *Oncogene* 39: 5267–5281

License: This is an open access article under the terms of the Creative Commons Attribution License, which permits use, distribution and reproduction in any medium, provided the original work is properly cited.

Expanded View Figures

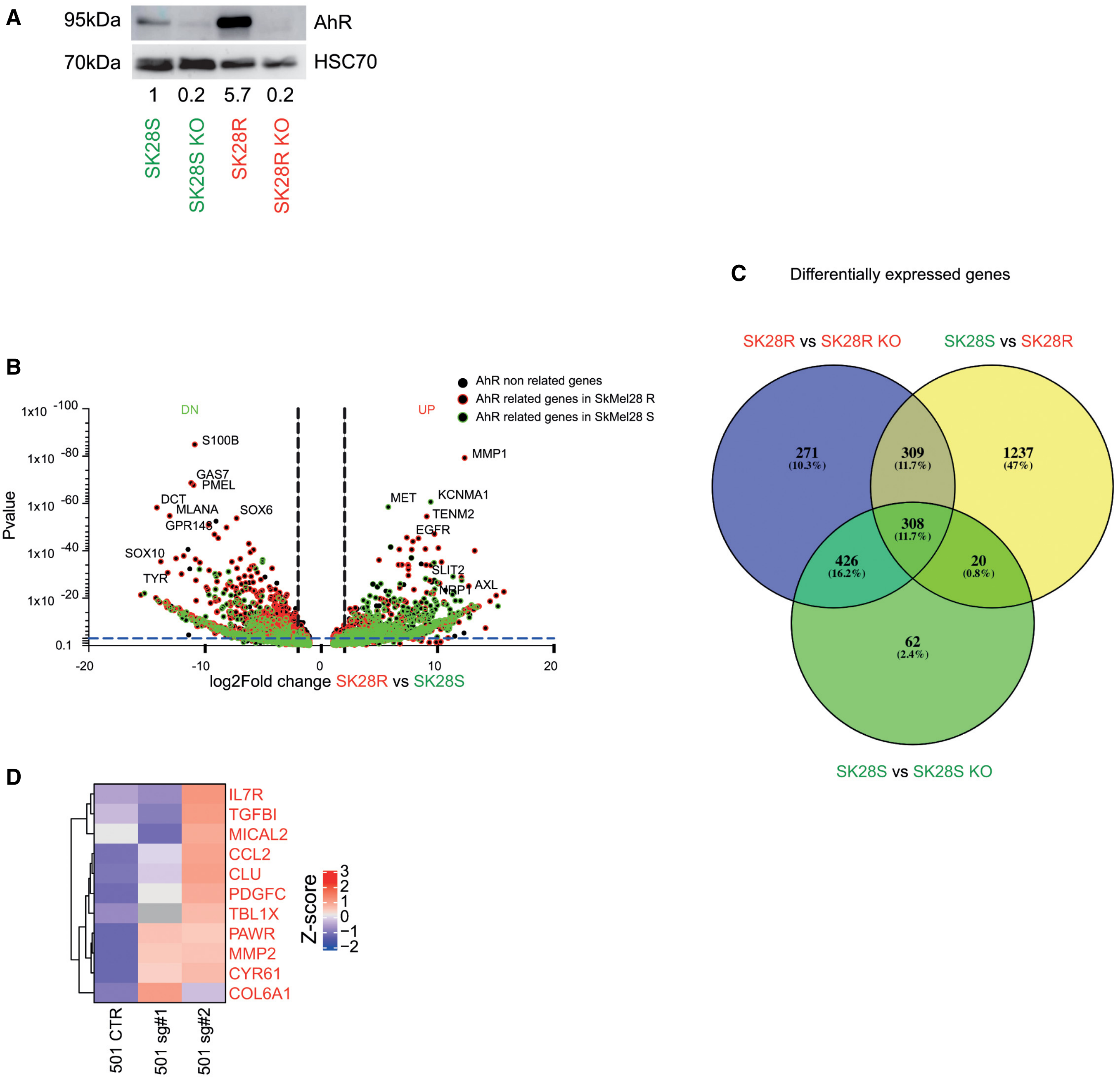

Figure EV1. Role of AhR in the regulation of gene expression in SK28 melanoma cell lines.

A Protein levels of AhR were analyzed by western blotting in BRAFi-sensitive or resistant SK28 cells invalidated or not for the AhR and quantified using Fiji® relative to the level of HSC70 protein.

B Volcano plot combining the magnitude of the fold change (ratio of expression) between SK28S (green), R (red) wild-type, or invalidated for AhR (KO) and the *P*-values.

C Venn diagram representing the overlap between the differentially expressed genes between SK28 R vs. R (Fig 2D), SK28S vs. SKO, and SK28R vs. RKO.

D Expression heatmap of the median expression ($n = 2$; RT-qPCR) for AhR target genes (invasion) in 501Mel CTR cells and those transduced with sgRNA targeting *AhR* (#1, #2). The scale corresponds to the Z scores.

Source data are available online for this figure.

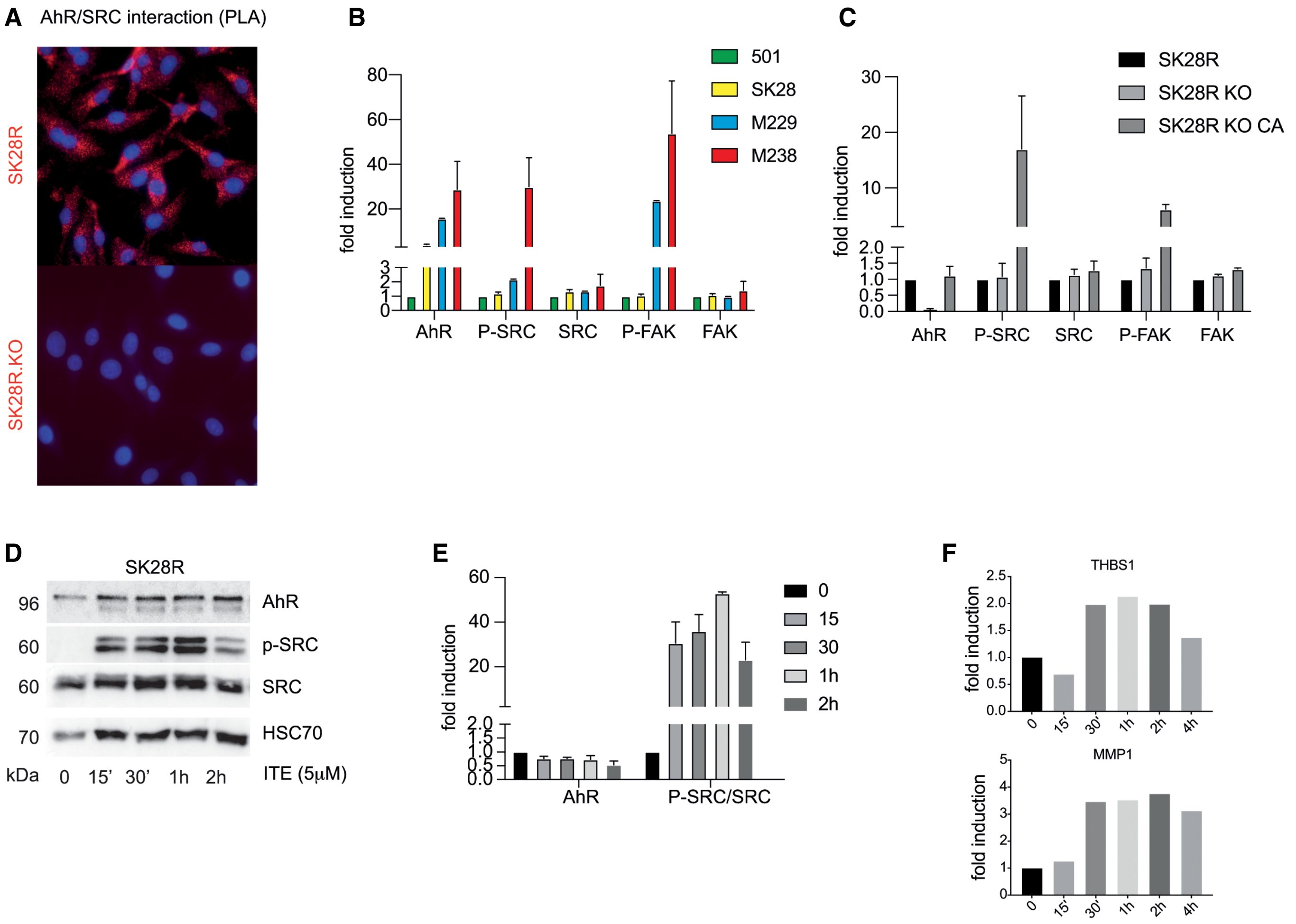

Figure EV2. Activation of SRC after canonical activation of AhR in melanoma cell lines.

A The AhR-SRC interaction was quantified by proximity ligation assay. Hoechst-stained nucleus in blue (20× magnification).

B Quantification of protein levels using ImageJ (Fiji) corresponding to Fig 4B ($n = 3$ biological experiments, mean ± s.d.).

C Quantification of protein levels corresponding to Fig 4C ($n = 3$ biological experiments, mean ± s.d.).

D, E Protein levels of the AhR, p-SRC (Y416), and SRC were analyzed by western blotting in SK28 after various times of treatment with ITE (5 µM) and quantified using ImageJ (Fiji) (E).

F Histogram showing the expression (RT-qPCR) of *THBS1* and *MMP1* in SK28 after treatment by ITE (5 µM) at different times ($n = 1$).

Source data are available online for this figure.

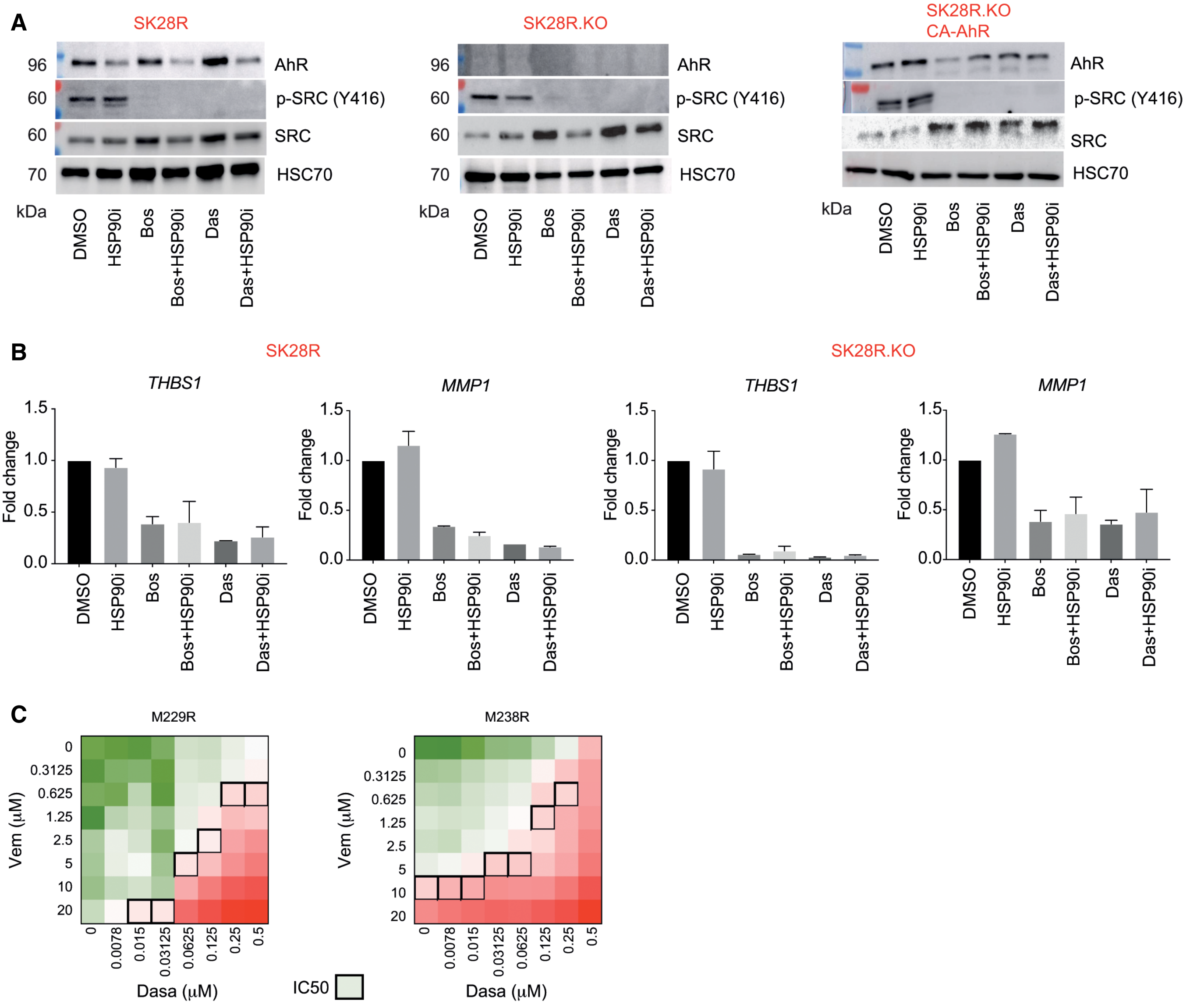

Figure EV3. SRC inhibitors sensitize melanoma cells to BRAFi treatment.

A Protein levels of AhR, p-SRC (Y416), and SRC in SK28R cells invalidated or not for AhR by CRISPR/Cas9 or after rescue with the activated-form of the AhR (CA-AhR) and after treatment for 24 h with an HSP90 inhibitor (HSP990, 10 nM) with or without two different SRC inhibitors: dasatinib (1 μM) or bosutinib (1 μM).

B Histogram representing the expression (RT-qPCR) of *THBS1* and *MMP1* in SK28R and SR28R KO treated for 24 h with an HSP90 inhibitor (HSP990, 10 nM) with or without two different SRC inhibitors: dasatinib (1 μM) or bosutinib (1 μM; $n = 3$ biological experiments, mean ± s.d.).

C Heatmap of the percentage of cell viability of M229R (left) and M238R cells treated with a combination of increasing doses of vemurafenib (y) and bosutinib or dasatinib (1 μM). IC50 values are represented by black squares.

Source data are available online for this figure.

Figure EV4. Impact of inhibition of SRC to sensitize PDX melanoma to BRAFi treatment.

A PDX model MEL006R (BRAFi resistant) was implanted in NMRI nude mice. Mice with tumors reaching 200 mm^3 were treated daily with vehicle ($n = 6$), dabrafenib alone (Dab, Biorbyt, 30 mg/kg, $n = 5$) dasatinib alone (Das, Selleckchem, 30 mg/kg, $n = 9$) or in combination dabrafenib + dasatinib (Das before combo, 30 mg/kg, $n = 12$). PDX tumor volumes were measured every 2 days until reaching 1,500 mm^3.

B IHC for AHR and P-SRC PDX model MEL006R representative for each group of treatment in order to confirm the action of SRCi to specifically prevent phosphorylation of SRC. Quantification of % of positive immunostaining area has been performed using ImageJ (Fiji) after integration of specific signal. Semi-quantitative analysis of optical density for several tumors for each group ($n = 4$) has been performed after calibration of the image using step tablet (Fiji).

 ©2022 The Authors

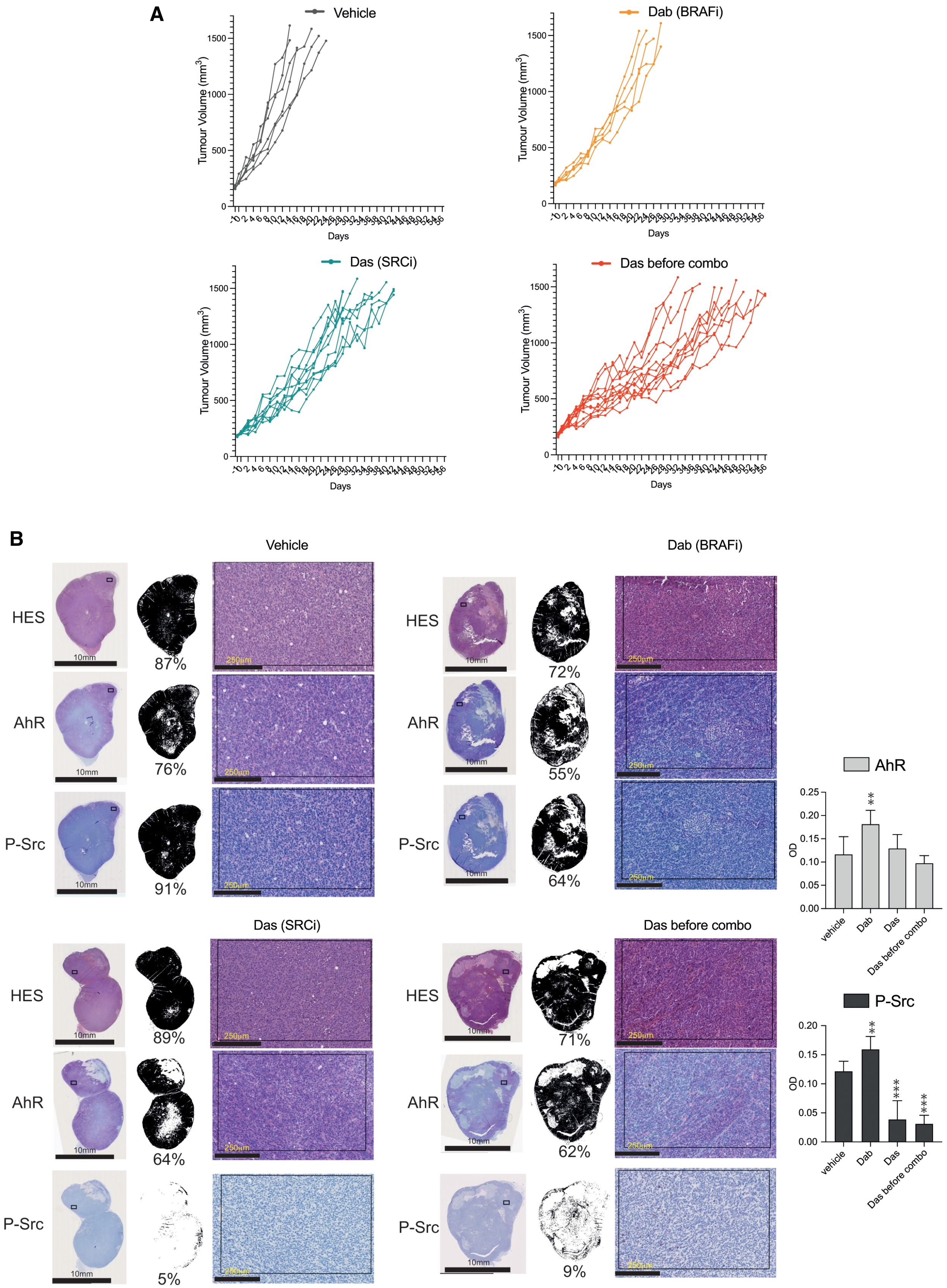
A
Vehicle
Dab (BRAFi)
Das (SRCi)
Das before combo
Tumour Volume (mm3)
Days
B
Vehicle
Dab (BRAFi)
Das (SRCi)
Das before combo
HES
AhR
P-Src
87%
76%
91%
72%
55%
64%
89%
64%
5%
71%
62%
9%
AhR
P-Src
OD
vehicle
Dab
Das
Das before combo

Figure EV4.

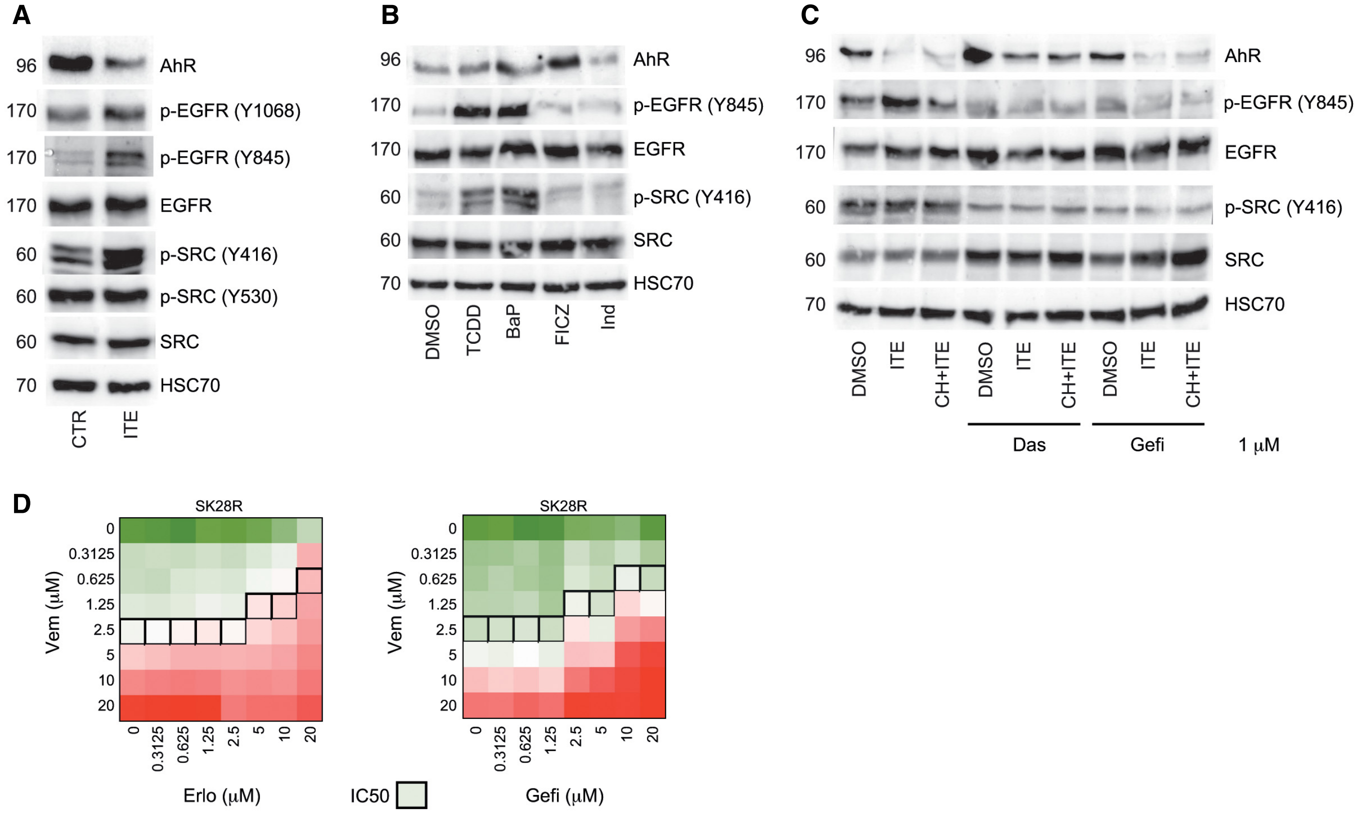

Figure EV5. Canonical activation of AhR leads to activation of the SRC/EGFR axis in melanoma cells.

A Protein levels of AhR, p-SRC (Y416, Y530), SRC, p-EGFR (Y1068, Y845) and EGFR were analyzed by western blotting in SK28R cells treated or not with ITE (5 μM, 24 h).

B Protein levels of AhR, p-SRC (Y416), SRC, p-EGFR (Y845), and EGFR, in the SK28R cell line after treatment with different AhR ligands for 24 h (10 nM TCDD, 5 μM BaP, 5 μM FICZ and 5 μM indirubin).

C Protein levels of AhR, p-SRC (Y416), SRC, p-EGFR (Y845) and EGFR were analyzed by western blotting in SK28R cells treated or not with ITE (5 μM, 24 h) with or not AhR inhibitor (CH-223191, 5 μM), Src inhibitor: dasatinib (0.5 μM) and EGFR inhibitor: gefitinib (20 μM).

D Heatmap of the percentage of cell viability in SKR cells treated with a combination of increasing doses of vemurafenib (y) and erlotinib (left) or gefitinib (right) (1 μM). IC50 values are represented by black squares.

Source data are available online for this figure.

 ©2022 The Authors
