# Supplementary material for: The AhR‐SRC axis as a therapeutic vulnerability in BRAFi‐resistant melanoma
Source: EMBO Mol Med. 2022 Oct 28;14(12):e15677. doi: 10.15252/emmm.202215677 (PMC9728058; doi:10.15252/emmm.202215677)
Supplement: Supplementary file 12 [file EMMM-14-e15677-s001.pdf]

EMBO Molecular Medicine

The AhR-SRC axis as a therapeutic vulnerability in BRAFi-resistant melanoma.

Anais Paris, Nina TARDIF, Maria Francesca Baietti, Cyrille BERRA, Héloise LECLAIR, Eleonora Leucci, Marie-Dominique GALIBERT, and Sébastien Corre
DOI: 10.15252/emmm.202215677

Corresponding authors: Sébastien Corre (sebastien.corre@univ-rennes1.fr) , Marie-Dominique GALIBERT (mgaliber@univ-rennes1.fr)

Review Timeline:

Submission Date:	4th Jan 22
Editorial Decision:	3rd Feb 22
Revision Received:	29th Aug 22
Editorial Decision:	12th Sep 22
Revision Received:	30th Sep 22
Accepted:	30th Sep 22

Editor: Zeljko Durdevic

Transaction Report:

(Note: With the exception of the correction of typographical or spelling errors that could be a source of ambiguity, letters and reports are not edited. Depending on transfer agreements, referee reports obtained elsewhere may or may not be included in this compilation. Referee reports are anonymous unless the Referee chooses to sign their reports.)

3rd Feb 2022

Dear Dr. Corre,

Thank you for the submission of your manuscript to EMBO Molecular Medicine. We have now received feedback from the three reviewers who agreed to evaluate your manuscript. As you will see from the reports below, the referees acknowledge the interest of the study but also raise important concerns that should be addressed in a major revision. After cross-commenting discussion it became clear that the focus of the revision should be on the in vivo invasive/metastatic potential in a xenograft mouse model as suggested by the referee #2.

Further consideration of a revision that addresses reviewers' concerns in full will entail a second round of review. EMBO Molecular Medicine encourages a single round of revision only and therefore, acceptance or rejection of the manuscript will depend on the completeness of your responses included in the next, final version of the manuscript. For this reason, and to save you from any frustrations in the end, I would strongly advise against returning an incomplete revision.

We would welcome the submission of a revised version within six months for further consideration. Please let us know if you require longer to complete the revision.

Please use this link to login to the manuscript system and submit your revision: Link Not Available

I look forward to receiving your revised manuscript.

Yours sincerely,

Zeljko Durdevic

Zeljko Durdevic
Editor
EMBO Molecular Medicine

***** Reviewer's comments *****

Referee #1 (Comments on Novelty/Model System for Author):

The technical quality is not optimal, as some statistical analysis and controls are missing. If the authors meet my requirements the technical quality will be high.

The novelty is for me average, because all aspects have already been published, i.e. SRC and BRAFi resistance, AhR and SRC and Ahr and resistance. Here again the work has the merit to link these different observations.
The medical impact may be strong because SRC inhibitors are already used in the clinic and could quite easily be combined with BRAF inhibitors

Referee #1 (Remarks for Author):

The work of Paris et al, dissects the molecular mechanisms regulated by AhR that are involved in the regulation of phenotypic transition in melanoma cells, and resistance to targeted therapies. Overall, this is a nice work that identifies SRC as a key player in the transmission of AhR effects and as a potential therapeutic target to resensitize resistant melanoma cells to BRAF inhibitors.
The results presented in this manuscript move the field forward and to my opinion deserve a publication in EMBO Mol Med provided that the authors are able to improve some points and to answer satisfactorily my questions
Major points

1) In the abstract, line 3 and in several other places in the text, the word "monitoring" is misused in my opinion. This word refers more to an observation or evaluation, rather than an active function on the process. I recommend changing this word to clearly show the functional link between AhR and the downstream processes.
2) In the introduction
*Line 48, the PFS data under BRAFi+MEKi are no longer true. The more recent analysis, (DOI:10.1136/jitc-2020-000948) shows a median PFS of at least 30 months with Ecorafenib and binimetinib. Please change this and cite the more recent reference.
*Line 62, "genetic reprogramming" is rather "transcriptomic reprogramming"
*Line 67-70, As written, it might appear to readers that all cases of non-genetic resistance are associated with de-differentiation. This is not the case, as the work of the Garraway and Marine groups has shown that resistance to BRAFi and MEKi can also be due to increased differentiation. Please include this notion in the introduction.
*Line 71-76, The most relevant references to show that MITF controls plasticity, EMT-like and invasiveness of melanoma are Carreira et al, Genes & Dev 2006 and Cheli et al, Oncogene 2012. Please note, citations are repeated twice.
3) In the Results. Overall, the results are accurately described, but the bioinformatics data would benefit from simplification. Some messages are blurred by an excess of bioinformatics analyses whose purpose is not always understood.
4) In the discussion,
the first sentence is an over-statement. See comment 3 on the introduction. Please introduce, here, some more moderation.
*Lines 363-366, please clarify the point about the timing for the introduction of SRC inhibitors.
5) Specific points
*Figure1.B. The difference of migration between SK28S and SK28R that we see on the histogram, is not visible on the pictures.
*Please explain what "% of migration" means
*Fig. 2.B and E. A statistical analysis must be done to show the specificity of the effect.
*Figure 3.A. Please explain why this workflow allows to decipher the direct role of AhR in resistance? Please simplify the description and highlight the key points of these analyses. I am not convinced of the value of the GDSC data analysis
*Figure 3.D. How many patients are in each category (B, DP1, DP2, DP3)?

*Figure 4.A. an immunoprecipitation with a control antibody is mandatory to draw a conclusion from this experiment.
*Figure 4.D. There is no basal phosphorylation of SRC in this experiment on SK28R, while in the ones shown in C and E, there is a strong basal. Please explain.
*The Figure 5 caption is incorrect.
*Figure 5.E. the effect of Dasatinib alone must be shown
*Figure 6 B and C, require statistical analysis.
6) Global question
In vitro and in vivo, because BRAFi, dasatinib and bosutinib appear to have effects on viability on their own, it is difficult to know whether there is a true resensitization to BRAFi or whether there is an additivity of the effects of BRAFi and SRCi. A solid argumentation on this point is essential.

Referee #2 (Remarks for Author):

This manuscript by Paris et al describes the role of AhR as a regulator of an invasive gene expression programme in melanoma and the interaction of AhR with SRC and how SRC inhibition sensitizes BRAF-mutant cells and tumours to BRAF inhibition. The work is interesting, the paper clearly written and the figures easy to read. However, there are a number of issues that should be addressed, mostly involving clarity and consistency but also experiments. First, in the first section the authors show that AhR is involved in regulating melanoma invasion. The models they use are scratch assay and tumor spheroid assays, both of which have limitations. Did they use transwell assays or analyze the migration ability of live cells? In Figure 2B the curves are sigmoid in shape whereas in Figure 2E they are not. Why is this difference? Second, it is rather difficult to follow the text on page 6 describing the data in Figure 2G and Sup Fig 2. What exactly is the point and can it be stated clearly early on? Would geneset enrichments be more appropriate here? Also, since AhR seems to follow the phenotypic switch in melanoma cells the question arises if it depends on MITF or vice versa. Third, it is rather difficult to follow the discussion in the section on ´AhR regulates the expression of genes...´. I can understand why they want to search for overlaps between AhR targets and the differentiation/invasion signatures but it is not clear why they involve Braf-treatment at this point. Also, they say they identified 216 direct or indirect AhR targets but do not say in what analysis they found them (among the 280 differentiation/invasion genes? Or is Braf also involved?). The logic of the rest of the discussion is also rather difficult to follow and at least this reader needs more help. The status of MITF in the gene expression analysis should be drawn out clearly. Fourth, the data presented in Sup Fig. 4A is not very clearly described in the text. What are the red and blue boxes on top, and what are the red and green genes and what does the 17% in the text refer to. Should the order of the genes not be the same in Figures 3B and Sup Fig 4A (this is true for some of the other figures also, including Figures 4F and G)? Why is SLAMF7 not in Figure 3B? Fifth, I am not convinced by their conclusion that ´The functional annotation of these differentially expressed genes underscores AhR-induced SRC as an integral component....´ in the pathways mentioned. Functional annotations of GO terms are very general and broad and should be used with caution. The last paragraph on page 9 which continues at the top of page 10 needs editing and further analysis. Lastly, the xenograft experiments do not assay for migratory/invasive potential of the tumor. This is the key feature studied througout the manuscript and therefore invasion needs to be the focus of the tumour analysis in the animal model. They only determine tumour volume and survival but do not tell us about invasive potential. Also, it would have been informative to see the effects of SRC and AhR knockdowns in the xenograft models and to have an estimate of the expression of SRC and AhR in these cells.

Minor points:
1. Page 9, line 250: ´....allowed THE identifICATION OF a significant number of genes...´
2. Page 9, line 259: What does ´(red)´ refer to in the sentence ´GSEA in different melanoma cell lines (red)...´?
3. The term ´invalidated or not´ is not good English. It is more accurate to say ´RNAseq performed after knockout out of AhR....´
4. Figure 5B is rather difficult to read. What are the dots and what do the labels in the figure say?
5. Figures 5D and 5E are inverted in the text on page 11 (line 299).

Referee #3 (Comments on Novelty/Model System for Author):

Although authors use published expression data to support their claims, additional patient samples via stainings (i.e. AhR and p-SRC?) would increase the translational relevance. PDX tumours should be stained too for p-SRC to show that drug was on target.

Referee #3 (Remarks for Author):

In this interesting and important study, Paris and colleagues find more mechanistic insights into how Aryl hydrocarbon (AhR) signalling promotes resistance to BRAF inhibitors in melanomas, which was previously described by the group. AhR regulates an invasive gene expression programme through activation of SRC/FAK signalling. Therefore, SRC blockade overcomes

resistance and invasive phenotype. Authors also provide preclinical evidence using a BRAF inhibitor-resistant PDX mouse model that SRC inhibition delays growth of the BRAFi-resistant PDX tumours.

The study is very relevant since it provides additional mechanistic insights into a pathway involved in resistance (AhR), and also another potential therapeutic approach (SRC inhibition). SRC inhibition has already been shown to overcome resistance as authors already acknowledge in the discussion (Girotti 2013, Krayem 2020). Whether combined SRC+AhR (and EGFR?) inhibition can truly shrink BRAFi-resistant tumours may be in the works for future studies of the group, but this should be discussed in the manuscript.

Although authors use published expression data to support their claims, additional patient samples via stainings (i.e. AhR and p-SRC?) would increase the translational relevance. PDX tumours should be stained too for p-SRC to show that dasatinib was on target.

Before publication, authors should clarify a few issues.

Western blot and q-RT-PCR experiments should state that they are representative of n experiments, and show average+error in the quantifications (if they come from independent experiments).

Specific comments.

Were Resistant cells grown in the presence of BRAFi? And in the migration/invasion assays?
It should be clarified since it is not clear from methods. This is important regarding combination therapy. In particular, given that SK28R KO for AhR die in the presence of vemurafenib (Fig.1A). Could these KO cells grow as the parental (R)?

Fig.2E- it is impressive that CA-AhR in R-KO is even more resistant than R. In 3C transcriptomic analysis, the rescue with CA-AhR also increased expression of some genes even higher expression than basal R (NUAK1 etc) while other genes are not fully restored (COL6A SOX9 etc).
Is this CA somehow more stable, or is it doing something else?
When comparing the gene list of SK28-R and R-CA (Fig.4C) vs the 501mel AhR-SAM (Fig.S3D), the latter only shows a subset, were the other genes not analysed, or there were no differences?
In the 501mel, some genes were only upregulated with sg2 that in 2A seems to give higher AhR levels. Authors should comment on this.

Fig.3B- what does the colour on the genes (green or red) mean?
An on CCLE- GDSC? If it relates to BRAFi-sensitivity or Resistance it should be stated in legend/panel
Red genes that are high in Tsoi-undifferentiated-NCL are high in 28R but do not change much in R-KO (do not turn blue). However, the high in Tsoi-melanocytic are lower in R compared to R-KO.

Fig. 3D- which patients were used in this analysis? Not all patients in Hugo 2015 had 3 DP biopsies. This should be clarified. Perhaps authors could also include BRAFi+MEKi+DP biopsies from Hugo 2015, since S4C shows similar results in BRAFi+MEKi-R vs BRAFi-R lines.

Fig.4A - from the text it looks like this is skmel28, legend should state this. IP experiments should show an IgG control. Especially given the weak FAK band. There is a grey line on top of AhR in input, what is it?

Fig.4B- panel should say BRAFi intrinsic resistance. Does this intrinsic resistance data come from the author's own data or is it published? Perhaps it should be shown or through reference.
For the blots, how representative are these blots? The quantification in S5 shows a fold induction with error bars, does this come from several experiments? Also, total SRC barely changes in the quantification, but the blot shows higher levels in all lines vs 501mel.
The fold induction is vs 501mel? It's unclear.
Are phospho levels normalized vs the total protein of interest (i.e., p-SRC/SRC, etc) or vs loading control (Hsc70?). This should be clarified.

Fig.4C and S5C. similar comment as previous regarding reproducibility.
In addition, p-SRC blot shows increased levels in SK28R.KO, but the graph in S5C shows even a lower bar (no error bars here). The reduction of p-FAK (blot) is difficult to see in the graph, the lower part of the graph should be expanded so downregulations can be observed.

In resistant cells the link between p-SRC and p-FAK may not be that obvious, since in AhR KO p-SRC blot increases (or does not change according to the author's comment in line 235) while p-FAK decreases. Only the CA-AhR in KO cells induces both phospho-proteins. Perhaps it is just AhR that activates directly FAK?

Fig.4D. Treatment with AhR ligands was for 24h. If AhR and SRC are in the same complex, should we expect an activation of

SRC (and FAK?) after a much shorter treatment with ligands?
In fact, p-SRC shows a huge increase after 15 min ITE treatment in skmel28 parental cells (Fig. S5D).
Fig.4E legend says "Protein levels of AhR, p-SRC (Y416), and SRC, in the SK28S cell line after 24 h of treatment with increasing doses of ITE and in SK28S AHR KO cells at various short times after ITE treatment (5 μM)". However, blots seem to be from only one of those conditions.
These panels should be clarified, it is confusing which cell lines were used, and if the mechanism is operative in both BRAFi-sensitive and -resistant cells.
In some cases, cell line is called SK28 (fig.s5d), in others SK28S (fig.4E), this should be consistent throughout the manuscript.

Fig.4G. here again the CA-AhR only rescues one signature (focal adhesion), in the sense of bringing back levels to the R control line. The integrin and FAK are even more downregulated than in the R control. SRC is upregulated vs R control. Authors should comment on this.

Fig.5B- given the effects on viability (Fig.5A), it is not clear if the reduced spheroid invasion is rather reduced growth/increased death.

Fig.5F. Bos increases Resistance and CSC signature in SK28R.

Fig.6. Mel006R PDX is said to be BRAFi resistant. Just to clarify, were PDX-implanted mice treated with BRAFi before randomization?
Fig.6 title of figure legend "Inhibition of SRC delays BRAFi resistance in a PDX model" is misleading, it could be interpreted as if SRC inhibition delays development of BRAFi resistance in a therapy naïve model. I suggest changing it to the one is used in the results section
"Inhibition of SRC sensitizes melanoma cells to BRAFi treatment", which is also similar to a sentence in the Abstract describing these results.

Fig.6A- just out of curiosity, did authors try the combination from the beginning (instead of pre-treating first with SRCi and then adding along BRAFi)?
Fig.6B- legend says "Two-tailed unpaired t test for the different treatments was performed: **$p<0.01$.". Comparing what with what? At which day? In any case, since there are 4 groups, multiple comparison test (ANOVA) should be employed.
Also, were there any regressing tumour, or stable (flat line)? Perhaps the individual mice should be shown in a supplementary figure.
Fig.6C- are the differences in survival statistically significant?
Fig.6D - the asterisks are comparisons of what with what? Does this test take into account multiple comparisons?
Fig.6 - Authors stain tumours for p-SRC to show on target inhibition.

Discussion. Line 371-374. Authors say that activation of FAK in melanoma cells is driven by a paradoxical activation of melanoma-associated fibroblastas, induction of matrix production and remodeling leading to elevated integrin b1-FAK-SRC signaling (Hirata 2015).

However, Marin-Bejar et al 2021 Cancer Cell and Fallahi-Sichani 2017 Mol Syst Biol showed that BRAFi or BRAFi+MEKi treatment (24-48h) of melanoma cells cultured alone increased FAK activation. Therefore, FAK can be activated via intrinsic and extrinsic factors. This is in fact mentioned in Marine's lab publication (Marin-Bejar 2021 Cancer Cell, page 1140) "...However, FAK activation in NCSCs following MAPK inhibition did not appear to be strictly dependent on extrinsic factors. DT exposure indeed induced phosphorylation of FAK (and AKT) in BRAFV600E-mutant melanoma cultures ... This observation indicated that MAPK inhibition triggers FAK signaling in NCSC melanoma cells in a cell-autonomous manner."
This

Related to this, lines 368-371: Marin Bejar et al is mentioned, but then the reference in 371 is Rambow et al 2018 and unpublished data. Should this "unpublished" be Marin-Bejar et al 2021 Cancer Cell? Or is it the author's own unpublished data? Same comment for the unpublished data in line 378.

Minor
Some comments in the text regarding the font colour of genes should be stated in the corresponding figure legend too.

Fig.S4B and S7B: it looks like the time-course data is from Tsoi 2018 Cancer Cell, not Song. Line 213-214 of manuscript mentions Song.
Fig.S4C: legend mentions 2d, DTP, DTPP, but the panel only shows SDR, DDR lines.

Fig.5D,E- panel letters do not correspond with figure legend
Lines 74-76, 83-84: duplicated references

S10A. there are too many genes in upper right quadrant and it's difficult to see which colour is the most abundant here. Perhaps only showing the dot and the gene in a supplementary table would help?

S11C. In M229R, should the Vem IC50 start in the 0 uM Dasa (and not in 0.03125), like in the other lines?

***** Reviewer's comments *****

Referee #1 (Comments on Novelty/Model System for Author):

The technical quality is not optimal, as some statistical analysis and controls are missing. If the authors meet my requirements the technical quality will be high.

Statistical information and missing controls have been added to the main document in Figure legends.

The novelty is for me average, because all aspects have already been published, i.e. SRC and BRAFi resistance, AhR and SRC and Ahr and resistance. Here again the work has the merit to link these different observations. The medical impact may be strong because SRC inhibitors are already used in the clinic and could quite easily be combined with BRAF inhibitors.

We acknowledge and thank Revierwer #1 for his marked interest shown in our work on understanding the molecular mechanisms mediated by the AhR/SRC axis during the acquisition of resistance to BRAFi in melanoma, since it may have strong medical impact. We have considered all comments of Revierwer #1 and clarified the message on the dual role of the transcription factor AhR in the control of genomic expression and activation of the SRC pathway, both of which are involved in acquisition of the resistance phenotype.

Referee #1 (Remarks for Author):

The work of Paris et al, dissects the molecular mechanisms regulated by AhR that are involved in the regulation of phenotypic transition in melanoma cells, and resistance to targeted therapies. Overall, this is a nice work that identifies SRC as a key player in the transmission of AhR effects and as a potential therapeutic target to resensitize resistant melanoma cells to BRAF inhibitors.
The results presented in this manuscript move the field forward and to my opinion deserve a publication in EMBO Mol Med provided that the authors are able to improve some points and to answer satisfactorily my questions

We acknowledge and thank Revierwer #1 for his marked interest shown in our work on understanding the molecular mechanisms mediated by the AhR/SRC axis during the acquisition of resistance to BRAFi in melanoma, since it may have strong medical impact. We have considered all comments of Revierwer #1 and clarified the message on the dual role of the transcription factor AhR in the control of genomic expression and activation of the SRC pathway, both of which are involved in acquisition of the resistance phenotype.

Major points

1) In the abstract, line 3 and in several other places in the text, the word "monitoring" is misused in my opinion. This word refers more to an observation or evaluation, rather than an active function on the process. I recommend changing this word to clearly show the functional link between AhR and the downstream processes.

The sentence has been changed in the abstract (L19) “We show here that the Aryl hydrocarbon Receptor (AhR) transcription factor directly regulates the gene expression program associated with the acquisition of resistance to BRAFi in melanoma.”

In other places, “monitoring” has been changed as recommended: L94-116-425.

2) In the introduction
*Line 48, the PFS data under BRAFi+MEKi are no longer true. The more recent analysis, (DOI:10.1136/jitc-2020-000948) shows a median PFS of at least 30 months with Ecorafenib and binimetinib. Please change this and cite the more recent reference.

This sentence has been changed, L67 in the revised MS referring to Michielin *et al*, 2020.

*Line 62, "genetic reprogramming" is rather "transcriptomic reprogramming"

This sentence has been changed now in L80.

*Line 67-70, As written, it might appear to readers that all cases of non-genetic resistance are associated with de-differentiation. This is not the case, as the work of the Garraway and Marine groups has shown that resistance to BRAFi and MEKi can also be due to increased differentiation. Please include this notion in the introduction.

Indeed, as mentioned BRAFi and MEKi resistance can also occur from differentiated melanoma cells. This aspect is now discussed in the introduction from L90 to 91.

*Line 71-76, The most relevant references to show that MITF controls plasticity, EMT-like and invasiveness of melanoma are Carreira et al, Genes & Dev 2006 and Cheli et al, Oncogene 2012. Please note, citations are repeated twice.

This has been changed in the manuscript now in L89.

3) In the Results. Overall, the results are accurately described, but the bioinformatics data would benefit from simplification. Some messages are blurred by an excess of bioinformatics analyses whose purpose is not always understood.

This part of the results has been simplified in the main text in order to clarify the message (L192-209).

4) In the discussion,
the first sentence is an over-statement. See comment 3 on the introduction. Please introduce, here, some more moderation.

This point has been moderated in the discussion (L346-348) “The acquisition of resistance to targeted therapy can be in part mediated by transcriptional reprogramming, eliciting a phenotypic switch towards distinct drug-tolerant transcriptional states of melanoma cells state (Tsoi *et al*, 2018; Rambow *et al*, 2018)”.

*Lines 363-366, please clarify the point about the timing for the introduction of SRC inhibitors.

According to *in vivo* experiments using BRAFi resistant PDX, the addition of SRC inhibitors during BRAFi treatment allowed to re-sentisize melanoma to treatment and so to delay relapse. We propose that the cotreatment of patients with SRC inhibitors with BRAFi/MEKi should counteract resistance associated with transcriptomic reprogramming and so delay relapse (L388-393). SRC inhibitors seem to be more effective in a context of BRAFi resistance when the levels of activation of AhR and SRC (after phosphorylation) are greater. It would seem possible to monitor the evolution of the tumor response and to detect the presence of circulating tumor DNA in order to anticipate relapse and therefore to be able to propose co-treatment with SRC inhibitors. This is now clarified in the revised version (L389-394).

5) Specific points

*Figure1.B. The difference of migration between SK28S and SK28R that we see on the histogram, is not visible on the pictures.

The picture illustrating migration in SK28R has been changed on Figure 1 to best illustrate the average calculated on at least 3 independent experiments.

*Please explain what "% of migration" means

The percentage of migration corresponds to the calculation of the difference between the measurement of the residual space between the cells (after 15h) (mean of measurement of 10 spaces represented in the figure for n>3 independent experiments) and the initial space between the cells (T0). % of migration = (a-b)/(a)x100
This information is now present in Figure 1B

*Fig. 2.B and E. A statistical analysis must be done to show the specificity of the effect.

Statistical analysis (2way ANOVA) has been performed between the mean of 3 independent experiments (501 CTR, 501 sg#1, 501 sg#2) or (SK28R, SK28R KO, SK28R KO CA-AhR) at different concentrations; $p<0.001$ ***. Missing comparisons have been added to Figure 2B and 2E.

*Figure 3.A. Please explain why this workflow allows to decipher the direct role of AhR in resistance? Please simplify the description and highlight the key points of these analyses.

Several genes have been previously associated with the different melanoma phenotypes (proliferative/invasive; differenciated/dedifferenciated). We compare these genes with AhR target genes identified by both ChIPseq analysis or with genes overexpressed in response to TCDD (AhR canonical agonist) in order to identify putative AhR target genes among melanoma gene signatures. This part has been simplified in the main text (L192-209).

I am not convinced of the value of the GDSC data analysis

Since Reviewer #1 is not convinced by GDSC data analysis and since this information is not indispensable, to clarify the message we have moved these data from the main figure to the related supplementary figure (Appendix Figure S1A).

*Figure 3.D. How many patients are in each category (B, DP1, DP2, DP3)?

9 patients under BRAFi treatment alone have been analyzed for the genes signatures
B = 9, DP1 = 9, DP2 = 9, DP3 = 5
This information has been added to the legend of Figure 3D (L744-746).

*Figure 4.A. an immunoprecipitation with a control antibody is mandatory to draw a conclusion from this experiment.

Immunoprecipitation experiment has been repeated with the control antibody. Item in Fig. 4A has been replace.

*Figure 4.D. There is no basal phosphorylation of SRC in this experiment on SK28R, while in the ones shown in C and E, there is a strong basal. Please explain.

Indeed, the level of phosphorylation of SRC, Figure 4D, is weak in control condition and is significantly higher after a 24h induction with AhR ligands. We repeated AhR induction experiments in Figure 4D. In this new experiment we observed a basal phosphorylation of SRC in control condition (DMSO) and still a significant but weaker induction phosphorylation of SRC after 24h induction of AhR by its different ligands. Figure 4D has been replaced.

*The Figure 5 caption is incorrect.

Legend of Figure 5 has been corrected.

*Figure 5.E. the effect of Dasatinib alone must be shown

This information has been added to Figure 5E. Increasing concentrations of Dasatinib on its own led to the decrease of AhR transcriptional activity. This result reinforces the feedback loop of AhR/SRC axis.

*Figure 6 B and C, require statistical analysis.

Statistical information has been implemented on Figure 6B and C.
In Figure 6B, we compared tumor size between different conditions of treatment at different times in order to know when tumor growth curve starts to be significantly decreased upon Das or combined therapy compared to BRAFi treatment alone (Dab).
In Figure 6C, we performed statistical analysis (logrank test) of survival curve.
This information has been implemented in Figure 6 (L802-809).

6) Global question

In vitro and in vivo, because BRAFi, dasatinib and bosutinib appear to have effects on viability on their own, it is difficult to know whether there is a true resensitization to BRAFi or whether there is an additivity of the effects of BRAFi and SRCi. A solid argumentation on this point is essential.

Indeed, the treatment of cells at relatively high concentrations (>0.125 uM for Bos and >0.031 uM for Das) seems to affect cell viability (Figure 5A). However, the combined effect observed on cell viability in combination with BRAFi is observed at low doses of SRC inhibitors (<0.015uM for Bos and <0.0078 uM for Das) (Figure 5A). This lets us think of the combined effect of the two inhibitors even if the SRC inhibitors are already impacting tumor growth.

Concerning the *in vivo* experiment, it is quite difficult to answer whether SRCi really sensitized BRAFi treatment while Dasatinib alone had already had an impact on tumor growth. However previous experiments have shown that SRCi were poorly efficient on BRAFi sensitive melanoma whereas in our experiment it really impacts growth of BRAFi resistant tumors. It seemed in fact that SRCi were much more efficient in a context of BRAFi resistance for which AhR and SRC are highly activated.

The MS has been modified accordingly as follow:

"To analyse the effect of SCRi on cell viability, SK28R melanoma cells were treated alone with increasing doses of SRC inhibitors (Bos or Das, up to 0,5 uM)) or in combination with BRAFi. Alone SCRi affected cell viability when used at relatively high concentration (>0.125 uM for Bos and >0.031 uM for Das). In contrast, when used in combination with BRAFi, the effect on cell viability was observed at low doses of SRCi (<0.015uM for Bos and <0.0078 uM for Das (**Fig 5A**). Since it has been shown that SRCi alone had poor effect on BRAFi-sensitive melanoma cells, together it suggests that SCRi may resensitize resistant-melanoma cells to BRAFi." (L302-309).

Referee #2 (Remarks for Author):

This manuscript by Paris et al describes the role of AhR as a regulator of an invasive gene expression programme in melanoma and the interaction of AhR with SRC and how SRC inhibition sensitizes BRAF-mutant cells and tumours to BRAF inhibition. The work is interesting, the paper clearly written and the figures easy to read. However, there are a number of issues that should be addressed, mostly involving clarity and consistency but also experiments.

We thank Referee #2 for his interest in the present in vitro and in vivo work he found clear and have addressed all issues underscored by the reviewer.

First, in the first section the authors show that AhR is involved in regulating melanoma invasion. The models they use are scratch assay and tumor spheroid assays, both of which have limitations. Did they use transwell assays or analyze the migration ability of live cells?

Transwell assays have indeed been performed to compare SK28R (labeled with dtomato) and SK28R KO (labeled with GFP). The results obtained were significant and confirmed that the lack of AhR prevents the invasive phenotype of the cells (see informative figure for reviewer #2-A). Since these invasion assays have been only performed in these 2 cell lines (SK28R labeled with dtomato and AhR-KO SK28R labeled with GFP) we filled that there should not be included in the main MS. This can be modified according to Reviewer #2 feedback.

In Figure 2B the curves are sigmoid in shape whereas in Figure 2E they are not. Why is this difference?

Indeed, the curves of cell viability are quite different between 501Mel cells and SKMel28R. This may be explained by their difference in sensitivity to BRAFi. 501Mel are per se sensitive to BRAFi (IC50 of….) which is reflected by the marked impact of BRAFi. Cells enter a death crisis when the IC50 is reached. SKMel28R have been obtained after exposure of sensitive SKMel28 cells to BRAFi. Acquisition of resistance is the result of cell reprogramming. The absence of sigmoid may thus reflect cell resistance heterogeneity.
To support this hypothesis, we can visualize a sigmoid shape when the cells express the constitutive active form of the AhR transcription factor (SK28KO-CA-AhR) even though we lack of values after 10uM.

Second, it is rather difficult to follow the text on page 6 describing the data in Figure 2G and Sup Fig 2. What exactly is the point and can it be stated clearly early on?

Fig 2G aims to underscore the biological pathways that characterize sensitive versus resistant melanoma cells expressing or not WT-AhR or the constitutive form of AhR (CA-AhR). This figure emphasizes the role of AhR in directing invasion, resistance and dedifferentiation.
To this end we examined the median of expression of different gene signatures (Invasive, Resistance, Mesenchymal….) previously associated with melanoma phenotype. The genes belonging to these signatures are described in Supplemented Table EV1.
This point has been simplified and clarified in the main text (L192-208).

Would geneset enrichments be more appropriate here?

For Fig 2G, Geneset enrichment is not possible because of too few conditions (5), Geneset enrichment recommends having at least 10 conditions.

Also, since AhR seems to follow the phenotypic switch in melanoma cells the question arises if it depends on MITF or vice versa.

Indeed, this remark is perfectly relevant since the phenotypic changes are associated with the loss of expression and activity of MITF (Hoek, Carreira, Cheli, Steingrimsson). We looked at the level of expression of MITF in our cell lines and we observed a significant decrease or loss of MITF protein expression in resistant cell lines compared to the sensitive ones (see informative figure for reviewer #2-B). In addition the increase of AhR expression in 501Mel (sgRNA, Figure 5A) correlates with a decrease of MITF expression (see informative figure for reviewer #2-C). However, in the current state, it is not possible for us to say whether the aggressive phenotype of melanoma is due to the loss of MITF or to the overexpression of AhR or to both. This axis is currently under investigation in our team in order to understand this balance of expression and activity between the AhR and MITF factors associated with phenotypic switching. The balance between both transcription factors will be part of a new manuscript.

Third, it is rather difficult to follow the discussion in the section on ´AhR regulates the expression of genes...´. I can understand why they want to search for overlaps between AhR targets and the differentiation/invasion signatures but it is not clear why they involve Braf-treatment at this point.

It is well established that short term BRAFi treatment of melanoma cells induces the expression of genes associated with differentiation (pigmentation genes…) mediated by MITF. On the contrary, we showed previously that the canonical activation of AhR by its ligands leads to the increase of expression of BRAFi resistance genes. We therefore compared the expression data of melanoma lines treated with Vemurafenib or TCDD in order to focus on genes associated respectively with differentiation or dedifferentiation of melanoma cells.

Also, they say they identified 216 direct or indirect AhR targets but do not say in what analysis they found them (among the 280 differentiation/invasion genes? Or is Braf also involved?). The logic of the rest of the discussion is also rather difficult to follow and at least this reader needs more help.

The bioinformatic part of the text has been simplified and clarified in the main text (L192-208). Several genes have been previously associated with the different melanoma phenotypes (proliferative/invasive; differenciated/dedifferenciated). We compare these genes with AhR target genes identified by both ChIPseq analysis or with genes overexpressed in response to TCDD (AhR canonical agonist) in order to identify putative AhR target genes among melanoma gene signatures allowing us to identify 216 genes potentially regulated by the transcription factor (Appendix Fig S2A). Using the KO SKMel28R cell line, we then confirmed the role of AhR in the regulation of the expression of 25 genes, the most significantly enriched in both resistant and dedifferentiated melanoma cell lines (Fig. 3B).

The status of MITF in the gene expression analysis should be drawn out clearly.

Dilshat et al. have recently shown that the MITF transcription factor is essential to maintain differentiation phenotype of melanoma cells by directly regulating the expression of genes participating in pigmentation (*DCT, MLANA, OCA2..*). On the contrary, they have shown that the lack of MITF transcription factor was associated with focal adhesion activation, a feature observed in drug-resistant melanomas. They also proposed using MITF CUT-and-RUN peaks that MITF acts as a repressor of several ECM and focal adhesion genes whereas MITF has been frequently defined as a transcription activator.
Interestingly, we observed similar phenotypes in our BRAFi resistant cells while AhR transcription factor is highly expressed after activation of SRC pathway. Both results could be correlated with a balance between both transcription factors. It would be interesting to see whether AhR could positively regulate the expression of ECM and focal adhesion genes.
The analysis of the expression of genes belonging to the different signatures in deltaMITF-X6 cells (exhibiting complete loss of wild type MITF) to the EV-SkMel28 control cells (Dilshat et al. 2021) showed a similar pattern with our SKMel28R. In these deltaMITF-X6 cells, we observed a significant decrease of proliferation/differentiation genes and at the opposite an significant increase of resistant/dedifferentiation/invasion genes but also an increase of Focal adhesion or SRC pathway genes (see informative figure for reviewer #2-D). Furthermore, we also observed in their deltaMITF-X6 cells a significant increase of AhR expression and of its target genes (see provided figure D). In conclusion, both loss of MITF and gain of AhR favor the activation of focal adhesion and aggressive phenotype suggesting a balance between the two transcription factors for the control of melanoma plasticity. This aspect has been implemented in the discussion part (L355-358).
However, as already mentioned to Reviewer #1, the AhR/Mitf balance and cross-regulation is under investigation. We fell that a whole manuscript should be dedicated to this important question.

Fourth, the data presented in Sup Fig. 4A is not very clearly described in the text. What are the red and blue boxes on top, and what are the red and green genes and what does the 17% in the text refer to. Should the order of the genes not be the same in Figures 3B and Sup Fig 4A (this is true for some of the other figures also, including Figures 4F and G)?

In Sup Fig. 4A (now Appendix Figure S3A), we analyzed the expression level of the different genes previously associated with BRAFi sensitivity (25 genes in green) or with BRAFi resistance (25 genes in red) and AhR, namely the AhR-associated gene-signature in RNAseq data from the TCGA (SKCM, n= 454). The AhR-associated gene-signature allowed to cluster melanoma samples in two groups. One with a high expression of AhR-associated resistance genes (red box) (17% of patients) and one expressing BRAFi sensitive genes (blue box). Interestingly, these 17% of patients showed significantly lower overall survival than those highly expressing genes for sensitivity (green). This point has been clarified in the text (L217-221).
The order of the genes horizontally in the heatmap corresponds to the hierarchical clustering between the expression levels of the different genes (proximity of expression profile between genes) for the same experimental condition (patient, cell) and can therefore vary between the different figures. Order of genes in Figure 3B is defined with respect to hierarchical clustering in SKMel28 (left panel). Vertical clustering between different cells or patients is more informative to associate genetic signatures (susceptible/resistant) with phenotype.

Why is SLAMF7 not in Figure 3B?

SLAMF7 is present in Figure 3B and its expression is decreased in BRAFi resistant cell lines (CCLE).

Fifth, I am not convinced by their conclusion that ´The functional annotation of these differentially expressed genes underscores AhR-induced SRC as an integral component....´ in the pathways mentioned. Functional annotations of GO terms are very general and broad and should be used with caution. The last paragraph on page 9 which continues at the top of page 10 needs editing and further analysis.

After identifying differentially expressed genes in SKMel28R cells after induction of AhR by its ligands (ITE) or after treatment with Dasatinib, we performed functional annotations using EnrichR (KEGG_2019_human) (Table EV5) in order to identify common potential pathway linked to AhR/SRC axis. As indicated, functional annotations are very general and broad, that's why we illustrate the role of identified genes in melanoma with several references in the main text. Among the genes regulated by the AhR/SRC axis, *THBS1*, *PDGFC*, *NRP1* or *ITGA3* have been previously described as being particularly important to promote invasive phenotype in melanoma. References have been added in the results part (L268).

Lastly, the xenograft experiments do not assay for migratory/invasive potential of the tumor. This is the key feature studied througout the manuscript and therefore invasion needs to be the focus of the tumour analysis in the animal model. They only determine tumour volume and survival but do not tell us about invasive potential. Also, it would have been informative to see the effects of SRC and AhR knockdowns in the xenograft models and to have an estimate of the expression of SRC and AhR in these cells.

The main objective of the *in vivo* experiments carried out using the BRAFi-resistant PDX model (Mel006R) was to assess the effect of treatment of Dasatinib (SRCi) in combination with BRAFi on tumor growth in a resistant setting.
As proposed by Marin-Bejar et al. (2021) MEL006 PDX model is particularly relevant to study i*n vivo* nongenetic reprogramming of resistance to BRAFi/MEKi since MEL006 do not accumulate new somatic mutations. However, they are poor model to evaluate invasion as they do not metastasize in the time frame of the experiments. In accordance, Krepler et al., showed that PDX models for melanoma are highly heterogeneous and only a small proportion of these PDX have the ability to metastasize. To generate spontaneous metastasis, cells from PDX were inoculated into a human skin graft on NSG mice, tumors formed within the human dermis. These then metastasized out of the human graft into the lungs of host mice https://doi.org/10.1016/j.celrep.2017.10.021). Because tumor growth of the BRAFi-resistant PDX model (Mel006R) is very fast, this model is probably not appropriate to study the mechanisms of invasion and metastasis at least in the time window analyzed (20 to 60 days).
However, as recommended by the reviewer we performed a Sirius red staining of the PDXs recovered at the end of the experimentation following the various treatments (see informative figure for reviewer #2 and #3-A). Sirius red staining makes it possible to evaluate the presence or absence of local micro-invasion in the contours of the tumor with the presence of diffuse edges (see informative figure for reviewer #2 and #3-A). In our experiment, we didn't observe any local invasion of tumor cells. This point has been also confirmed by IHC (with the melanoma specific marker S100B) of lung. Again, S100B IHC (see informative figure for reviewer #2 and #3-A) didn't reveal the presence of metastatic melanoma cells in the lung in all different groups.

Minor points:

1. Page 9, line 250: ´....allowed THE identifICATION OF a significant number of genes...´

This has been corrected in the text (L263).

2. Page 9, line 259: What does ´(red)´ refer to in the sentence ´GSEA in different melanoma cell lines (red)...´?

(red) has been removed in the text (L265).

3. The term ´invalidated or not´ is not good English. It is more accurate to say ´RNAseq performed after knockout out of AhR....´

This has been corrected in the text (L277).

4. Figure 5B is rather difficult to read. What are the dots and what do the labels in the figure say?

The dots correspond to pictures of spheroids composed by a 50%/50% mix of SK28R WT labeled with dTomato (in red) and AhR KO cells labeled with GFP (in green) before and after five days of treatment with SRC inhibitors (1µM). Size of the different pictures have been increased on Figure 5B in order to clearly identify cells that invade the collagen matrix around spheroids.

5. Figures 5D and 5E are inverted in the text on page 11 (line 299).

This point has been corrected in Figure 5 (L316-317).

Referee #3 (Comments on Novelty/Model System for Author):

Although authors use published expression data to support their claims, additional patient samples via stainings (i.e. AhR and p-SRC?) would increase the translational relevance. PDX tumours should be stained too for p-SRC to show that drug was on target.

Referee #3 (Remarks for Author):

In this interesting and important study, Paris and colleagues find more mechanistic insights into how Aryl hydrocarbon (AhR) signalling promotes resistance to BRAF inhibitors in melanomas, which was previously described by the group. AhR regulates an invasive gene expression programme through activation of SRC/FAK signalling. Therefore, SRC blockade overcomes resistance and invasive phenotype. Authors also provide preclinical evidence using a BRAF inhibitor-resistant PDX mouse model that SRC inhibition delays growth of the BRAFi-resistant PDX tumours.

The study is very relevant since it provides additional mechanistic insights into a pathway involved in resistance (AhR), and also another potential therapeutic approach (SRC inhibition). SRC inhibition has already been shown to overcome resistance as authors already acknowledge in the discussion (Girotti 2013, Krayem 2020). Whether combined SRC+AhR (and EGFR?) inhibition can truly shrink BRAFi-resistant tumours may be in the works for future studies of the group, but this should be discussed in the manuscript.

We acknowledge and thank the Referee #3 for his/her interest in our work, underscoring how the AhR/SRC axis participates in the acquisition of resistance to BRAFi in melanoma, giving important prospects for therapeutics approach. Also, he/she underscored the importance of providing IHC staining to support our findings. We have considered these comments, and in particular we performed p-SRC IHC staining of PDX tumors in order to show the specific effect of dasatinib on its target.
Also because of the respective roles of AhR, SRC and EGFR in controlling BRAFi resistance may be critical, future experiments combining the different respective inhibitors would be particularly relevant. This point has been raised in the discussion (L417-423).

Although authors use published expression data to support their claims, additional patient samples via stainings (i.e. AhR and p-SRC?) would increase the translational relevance. PDX tumours should be stained too for p-SRC to show that dasatinib was on target.

In order to confirm the specific role of dasatinib in inhibiting activating phosphorylation of SRC during the treatment of tumor growth of PDX, we performed immunostaining (IHC) of P-SRC (Figure EV4). Treatment with dasastinib alone or in combination with BRAFi (Das before combo) led to a significant decrease in the level of P-SRC in treated PDX. BRAFi treatment alone didn’t prevent this phosphorylation (Figure EV4). Interestingly, dasatinib treatment also led to a slight decrease in AhR expression level in PDX confirming the feedback between the SRC and AhR pathway.
As proposed by Reviewer 3, the analysis of the level of P-SRC in patient tumors may serve as a marker for therapeutic monitoring to anticipate the mechanisms of resistance to BRAF inhibitors. Studying the AhR/SRC pathway in patient samples has been started. Preliminary data on retrospective tumor samples showed a slight increase in the level of P-SRC in non-responding patients, which now remains to be confirmed in a prospective cohort with tumor

sample from different stage. According to the time required to collect tumor samples this cannot be included in the present MS.

Before publication, authors should clarify a few issues.

Western blot and q-RT-PCR experiments should state that they are representative of n experiments, and show average+error in the quantifications (if they come from independent experiments).

This information has been added to figure legends.

Specific comments.

Were Resistant cells grown in the presence of BRAFi?

The BRAFi resistant cells have been established after a long terme treatment with increasing doses of BRAFi (3 month with BRAFi). After this, cells were usually grown in the absence of BRAFi except for the measure of BRAFi sensitivity (2x 2 days of treatment with BRAFi alone or in combination). A preventive treatment every two weeks with BRAFi (at the IC50 dose for sensitive cells) has been performed to confirm that cells remain resistant to treatment. This point has been implemented in the material and methods section (L443-445).

And in the migration/invasion assays?

Consisting with the previous procedure, migration and invasion assays have been performed in the absence of treatment with BRAFi.

It should be clarified since it is not clear from methods. This is important regarding combination therapy. In particular, given that SK28R KO for AhR die in the presence of vemurafenib (Fig.1A). Could these KO cells grow as the parental (R)?

SKMel 28 AhR KO cells grow at the same level of the SKMel28 parental ones in the absence of BRAFi treatment. So, the increase of BRAFi sensitivity in these KO cells is not related to a difference in cell proliferation (L442).

Fig.2E- it is impressive that CA-AhR in R-KO is even more resistant than R. In 3C transcriptomic analysis, the rescue with CA-AhR also increased expression of some genes even higher expression than basal R (NUAK1 etc) while other genes are not fully restored (COL6A SOX9 etc).
Is this CA somehow more stable, or is it doing something else?

The CA-AhR corresponds to the constitutively active form of the transcription factor. It lacks the PAS-B domain (ΔPASB) which is required to interact with AhR ligands (McGuire et al. 2001). This mutant does not require any more to be activated by canonical ligands, it locates into the nucleus with a higher transcriptional activity. CA-AhR has been shown to strongly stimulate XRE-driven reporter gene activity upon coexpression with ARNT in CHO cells in the absence of ligand (McGuire et al. 2001).
This supports our finding, with the CA-AhR leading to significantly increase the expression of AhR target genes. This also may explain why cells expressing CA-AhR were even more

resistant than the parental Resistant ones. Reference of McGuire et al. article describing the CA-AhR has been added in the material and methods part of the text (L480).
Some genes were not fully restored in the presence of CA-AhR, this may reflect a more complex transcriptional program, implicating specific regulators. We cannot exclude that the activity of such regulators was not modulated by the rescue by CA-AhR, leading to mild expression of COL6A and SOX9.

When comparing the gene list of SK28-R and R-CA (Fig.4C) vs the 501mel AhR-SAM (Fig.S3D), the latter only shows a subset, were the other genes not analysed, or there were no differences?

The missing genes for Fig. 4C were not represented on heatmap since they're not expressed in 501mel cells.

In the 501mel, some genes were only upregulated with sg2 that in 2A seems to give higher AhR levels. Authors should comment on this.

Indeed, the increase in the expression of resistance genes is greater in the sg2 condition than in the sg1 where the level of AhR overexpression is lower. Although an increasing trend is observed in the sg1 lines, there seems to be a threshold level of AhR required for the activation of the expression of the target genes. This agrees with the level of expression and activity of AhR which increases in lines resistant to BRAFi.

Fig.3B- what does the colour on the genes (green or red) mean?

The genes in green correspond to genes previously correlated with BRAFi sensitivity, differentiation and proliferation while the genes in red correspond to genes associated with resistance to BRAFi, the dedifferentiated and invasive phenotype.

And on CCLE- GDSC? If it relates to BRAFi-sensitivity or Resistance it should be stated in legend/panel

This point has been implemented in the legend of Figure 3 (L738).

Red genes that are high in Tsoi-undifferentiated-NCL are high in 28R but do not change much in R-KO (do not turn blue). However, the high in Tsoi-melanocytic are lower in R compared to R-KO.

As judiciously indicated by the referee, the loss of AhR in resistant cells, even if it significantly reduces the level of expression of the resistance/dedifferentiation genes, does not allow a return to a level comparable to those observed in the BRAFi sensitive lines (SK28S) (IC50 SKS< IC50 SKR KO). Even though AhR is critical to mediate BRAFi resistance, AhR is not the unique actor. This may explain while AhR KO cells maintain a relatively high level of expression of genes associated with BRAFi resistance.

Fig. 3D- which patients were used in this analysis? Not all patients in Hugo 2015 had 3 DP biopsies. This should be clarified.

Indeed, the analysis has been done on patients (Pt2, Pt3, Pt5, Pt6, Pt8, Pt9, Pt10, Pt15, Pt17) B = 9, DP1 = 9, DP2 = 9, DP3 = 5 that acquired resistance.

This information has been added on legend of Figure 3D (L744-746).

Perhaps authors could also include BRAFi+MEKi+DP biopsies from Hugo 2015, since S4C shows similar results in BRAFi+MEKi-R vs BRAFi-R lines.

The number of patients treated with BRAFi+MEKi is very low in this study (n=3) without all time of disease progression. The preliminary analysis from BRAFi+MEKi+DP biopsies from Hugo 2015 confirmed the increase of expression of AhR target genes for long term treatment.

Fig.4A - from the text it looks like this is skmel28, legend should state this. IP experiments should show an IgG control. Especially given the weak FAK band. There is a grey line on top of AhR in input, what is it?

Immunoprecipitation experiment has been repeated with the control antibody. Item in Fig. 4A has been replace.

Fig.4B- panel should say BRAFi intrinsic resistance. Does this intrinsic resistance data come from the author's own data or is it published? Perhaps it should be shown or through reference.

The level of BRAFi resistance corresponds to our measure of IC50 (Vemurafenib) for the different cell lines 501Mel (0,23uM), SKMel28 (0,29uM), M229 (0,89uM) and M238 (2,16uM). These cell lines correspond to the sensitive parental cells. This information has been added on legend of Figure 4B (L756-759).

For the blots, how representative are these blots?
The quantification in S5 shows a fold induction with error bars, does this come from several experiments?

The blots (now Figure EV2) are representative of 3 independent experiments corresponding to the quantification in S5.

Also, total SRC barely changes in the quantification, but the blot shows higher levels in all lines vs 501mel.

The increase of expression of total SRC in all cell lines vs 501mel is not significant compared to the increase of phosphorylation even though we observed a slight increase of its expression in resistant cell lines (Figure EV2).

The fold induction is vs 501mel? It's unclear.

The different values correspond to the mean of fold enrichment of expression in the different cell lines compared to 501mel (= 1) for each experiment.

Are phospho levels normalized vs the total protein of interest (i.e., p-SRC/SRC, etc) or vs loading control (Hsc70?). This should be clarified.

Quantification of protein levels (Figure EV2B) using ImageJ (Fiji) corresponding to Fig. 4B has been normalized vs loading control (Hsc70) and compared to the level of protein in

501Mel cells for each experiment (=1), phospho levels of P-SRC was normalized vs the total protein of interest (p-SRC/SRC) (n=3).
This information has been added in the legend of Figure EV2 (L828-829).

Fig.4C and S5C. similar comment as previous regarding reproducibility.
In addition, p-SRC blot shows increased levels in SK28R.KO, but the graph in S5C shows even a lower bar (no error bars here). The reduction of p-FAK (blot) is difficult to see in the graph, the lower part of the graph should be expanded so downregulations can be observed.

Quantification of protein levels using ImageJ (Fiji) (Figure EV2C) corresponding to Fig. 4C has been normalized vs loading control (Hsc70) and compared to the level of protein in SK28R cells for each experiment (=1), phospho levels of P-SRC was normalized vs the total protein of interest (p-SRC/SRC) (n=3). Comparing the different blots, both the increase of P-SRC and the slight decrease of P-FAK in SK28R KO didn't seem to be significant.

In resistant cells the link between p-SRC and p-FAK may not be that obvious, since in AhR KO p-SRC blot increases (or does not change according to the author's comment in line 235) while p-FAK decreases. Only the CA-AhR in KO cells induces both phospho-proteins. Perhaps it is just AhR that activates directly FAK?

The quantification of n=3 membranes showed variation of P-SRC and P-FAK is not significant in SKR KO. Significant phosphorylation of both SRC and FAK appeared only in the presence of activated form of AhR (CA-AhR). Even if phosphorylation of FAK is mediated by P-SRC, indeed we can't exclude that AhR may regulate direct FAK phosphorylation.

Fig.4D. Treatment with AhR ligands was for 24h. If AhR and SRC are in the same complex, should we expect an activation of SRC (and FAK?) after a much shorter treatment with ligands?

Indeed, and as shown with the use of ITE, SRC phosphorylation proceeds at early time points after exposure to AhR-ligand (Fig. EV2).

In fact, p-SRC shows a huge increase after 15 min ITE treatment in skmel28 parental cells (Fig. S5D).

Indeed, and as already mentioned above.

Fig.4E legend says "Protein levels of AhR, p-SRC (Y416), and SRC, in the SK28S cell line after 24 h of treatment with increasing doses of ITE and in SK28S AHR KO cells at various short times after ITE treatment (5 μM)". However, blots seem to be from only one of those conditions.
These panels should be clarified, it is confusing which cell lines were used, and if the mechanism is operative in both BRAFi-sensitive and -resistant cells.
In some cases, cell line is called SK28 (fig.s5d), in others SK28S (fig.4E), this should be consistent throughout the manuscript.

Thank you for identifying this error in the caption. The treatment with different doses of ITE (for 24 h) was carried out in the SK28R cells as for the 4D panel. This information has been

corrected on the legend of Figure 4D (L764) . All the experiments related to AhR activation and SRC phosphorylation have been performed in SK28R. This has been corrected in the text.

Fig.4G. here again the CA-AhR only rescues one signature (focal adhesion), in the sense of bringing back levels to the R control line. The integrin and FAK are even more downregulated than in the R control. SRC is upregulated vs R control. Authors should comment on this.

The heatmap represented in Fig 4G corresponds to the median of expression of the various genes associated with the signatures (SRC, INTEGRIN, FAK, etc.) allowing to capture the main information. In addition, Appendix Figure S6 shows the expression variations for the different genes. It is noted that the rescue of the activated form of AhR in the KO lines partially restores the level of expression observed in the parental resistant line. However, it can also be seen that KO also disrupts expression profiles. These differences of gene expression program may be explained by the difference of transcriptional activity of endogenous AhR (ARNT dependant) and the CA-AhR (McGuire et al. 2001) since AhR directly regulates the expression of genes belonging to SRC signature (such as *THBS1*…).

Fig.5B- given the effects on viability (Fig.5A), it is not clear if the reduced spheroid invasion is rather reduced growth/increased death.

It is difficult to compare directly Fig. 5A with Fig. 5B because in the spheroid model we used Das or Bos at the dose of 1uM. At this dose we didn't observe cell death. Moreover, we do not observe any significant difference in growth between SK28R and SK28R KO cells in the absence or in the presence of SRC inhibitors. However, the cell invasion capacity (in the collagen matrix) is different between the two lines. Treatment with SRCi makes it possible to have a level of invasion comparable to that observed in KO cells for AhR.

Fig.5F. Bos increases Resistance and CSC signature in SK28R.

Indeed, Bos seems to increase the median expression level of the signature associated with resistance or CSC. However, the expression of all the target genes of AhR associated with the acquisition of the aggressive phenotype is reduced. An increase in the level of expression of genes associated with proliferation and differentiation is also observed, which may explain the resensitization of cells resistant to BRAFi by SRC inhibitors.

Fig.6. Mel006R PDX is said to be BRAFi resistant. Just to clarify, were PDX-implanted mice treated with BRAFi before randomization?

PDX-implanted mice were not treated with BRAFi before randomization but they derive from MEL006 PDX lesions at relapse (Vendramin et al., 2021), upon acquisition of resistance to Dabrafenib and Trametinib. In early response to Dabrafenib, we indeed didn't observe any significant decrease in tumor growth usually observed with PDX models.

Fig.6 title of figure legend "Inhibition of SRC delays BRAFi resistance in a PDX model" is misleading, it could be interpreted as if SRC inhibition delays development of BRAFi resistance in a therapy naïve model. I suggest changing it to the one is used in the results section

"Inhibition of SRC sensitizes melanoma cells to BRAFi treatment", which is also similar to a sentence in the Abstract describing these results.

Thank you for this relevant suggestion. This has been modified in the text (line 775).

Fig.6A- just out of curiosity, did authors try the combination from the beginning (instead of pre-treating first with SRCi and then adding along BRAFi)?

Indeed, we tested the BRAFi + Dasatinib combination from the start of treatment. Even if tumor growth seems slightly slowed down in the first days of treatment, the growth curve (in black) is from the 16th day (corresponding to pre-treatment with Das) identical to the Das pre-treatment condition then the Das + BRAFi combination (in red) (see informative figure for reviewer #3).

Fig.6B- legend says "Two-tailed unpaired t test for the different treatments was performed: **p<0.01.". Comparing what with what? At which day? In any case, since there are 4 groups, multiple comparison test (ANOVA) should be employed.

Multiple comparison (2way ANOVA) statistical test have been performed for each group compared to Dab group and at different times. stars represent (*<0.05, **<0.01) time from when tumor size is significantly lower than in the group treated with BRAFi alone (Dabrafenib). This information has been added in legend of figure 6B (L803-806).

Also, were there any regressing tumour, or stable (flat line)? Perhaps the individual mice should be shown in a supplementary figure.

Tumor size values for individual mice have be added in a supplementary figure (Figure EV4A)

Fig.6C- are the differences in survival statistically significant?

Statistical tests for comparison of survival curves have been added on Figure 6C and in legend of Figure 6C (L808-809).

Fig.6D - the asterisks are comparisons of what with what? Does this test take into account multiple comparisons?
The asterisks correspond to non-parametric Mann-Whitney t test for the different treatments compared to vehicle.

Fig.6 - Authors stain tumours for p-SRC to show on target inhibition.

In order to confirm the specific role of dasatinib in inhibiting activating phosphorylation of SRC during the treatment of tumor growth of PDX, we performed immunostaining (IHC) of P-SRC (Figure EV4) on representative tumours for each group. Treatment with dasastinib alone or in combination with BRAFi (Das before combo) led to a significant decrease in the level of P-SRC in treated PDX. BRAFi treatment alone didn't prevent this phosphorylation (Figure EV4). Interestingly, dasatinib treatment also led to a slight decrease in AhR expression level in PDX confirming the feedback between the SRC and AhR pathway.

Discussion. Line 371-374. Authors say that activation of FAK in melanoma cells is driven by a paradoxical activation of melanoma-associated fibroblastas, induction of matrix production and remodeling leading to elevated integrin b1-FAK-SRC signaling (Hirata 2015). However, Marin-Bejar et al 2021 Cancer Cell and Fallahi-Sichani 2017 Mol Syst Biol showed that BRAFi or BRAFi+MEKi treatment (24-48h) of melanoma cells cultured alone increased FAK activation. Therefore, FAK can be activated via intrinsic and extrinsic factors. This is in fact mentioned in Marine's lab publication (Marin-Bejar 2021 Cancer Cell, page 1140) "...However, FAK activation in NCSCs following MAPK inhibition did not appear to be strictly dependent on extrinsic factors. DT exposure indeed induced phosphorylation of FAK (and AKT) in BRAFV600E-mutant melanoma cultures ... This observation indicated that MAPK inhibition triggers FAK signaling in NCSC melanoma cells in a cell-autonomous manner."

This comment is particularly relevant, it seems in fact that the activation of FAK in melanoma cells resistant to BRAFi/MEKi takes place in a cell-autonomous manner. As proposed in Marine's lab publication, MAPK inhibition led in undifferentiated NCSC subpopulation to the increase of expression of the transmembrane receptor GFRA2 and its ligand GDNF leading to the activation of AKT pathway and as a result of the FAK pathway.
This is in line with our results following the activation of AhR in resistant cells and following the activation of the SRC pathway after its phosphorylation. FAK activation has also been shown in melanoma cells lacking MITF transcription factor (Dilshat et al., 2021). The activation of FAK pathway and activation of focal adhesion could be associated with both the activation of AhR and the loss of MITF transcription factor which is associated with proliferation and differentiation phenotype. The AhR/SRC/FAK pathway seems to be insofar particularly important to control nongenetic reprogramming of resistance to BRAFi in an autocrine manner but also probably after activation of both AhR and SRC by paracrine factors secreted by cancer associated fibroblasts (DOI: 10.3389/fonc.2021.628821).
This dual activation of SRC/FAK upon resistance has been implemented in the discussion part (L426-429).

Related to this, lines 368-371: Marin Bejar et al is mentioned, but then the reference in 371 is Rambow et al 2018 and unpublished data. Should this "unpublished" be Marin-Bejar et al 2021 Cancer Cell? Or is it the author's own unpublished data? Same comment for the unpublished data in line 378.

Marin-Bejar's publication (Cancer cell 2021) is now referenced in the manuscript.

Minor
Some comments in the text regarding the font colour of genes should be stated in the corresponding figure legend too.

Color information of genes has been now added in the text of figure legend.

Fig.S4B and S7B: it looks like the time-course data is from Tsoi 2018 Cancer Cell, not Song. Line 213-214 of manuscript mentions Song.

Indeed, this has been corrected in Appendix Fig S3B and in the text (L228)

Fig.S4C: legend mentions 2d, DTP, DTPP, but the panel only shows SDR, DDR lines.

Many thanks the mistake on legend of Fig.S4C has been corrected (Appendix FigS3).

Fig.5D,E- panel letters do not correspond with figure legend

This point has been corrected on Figure 5.

Lines 74-76, 83-84: duplicated references

This point has been corrected in the text.

S10A. there are too many genes in upper right quadrant and it's difficult to see which colour is the most abundant here. Perhaps only showing the dot and the gene in a supplementary table would help?

The gene names in the upper Appendix Fig 8A have been removed from the figure and values have been added in table EV8.

S11C. In M229R, should the Vem IC50 start in the 0 uM Dasa (and not in 0.03125), like in the other lines?

For this experiment M229R were highly resistant to Vem (Figure EV3C). At the dose of 20uM, only 40% of cells died. That's why the IC50 start only in combination with low dose of Das (0.015uM)

Data Availability :
RNA-Seq data: Gene Expression Omnibus GSE166617 (https://www.ncbi.nlm.nih.gov/geo/query/acc.cgi?acc=GSE166617)
reviewer token : kbwfaksmbvghngf

A Transwell assays (D4) related to figure 1D

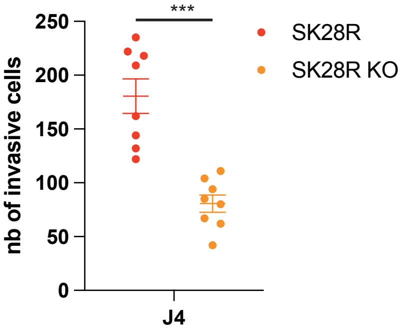

B related to Fig sup 1A

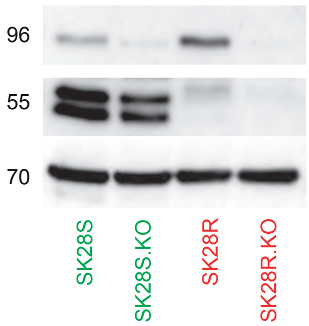

C related to Fig 2A

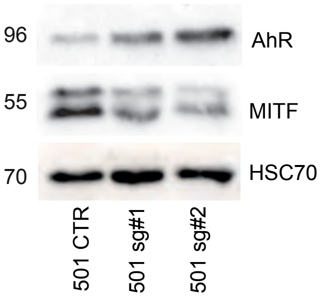

D Dilshat et al. 2021 elife

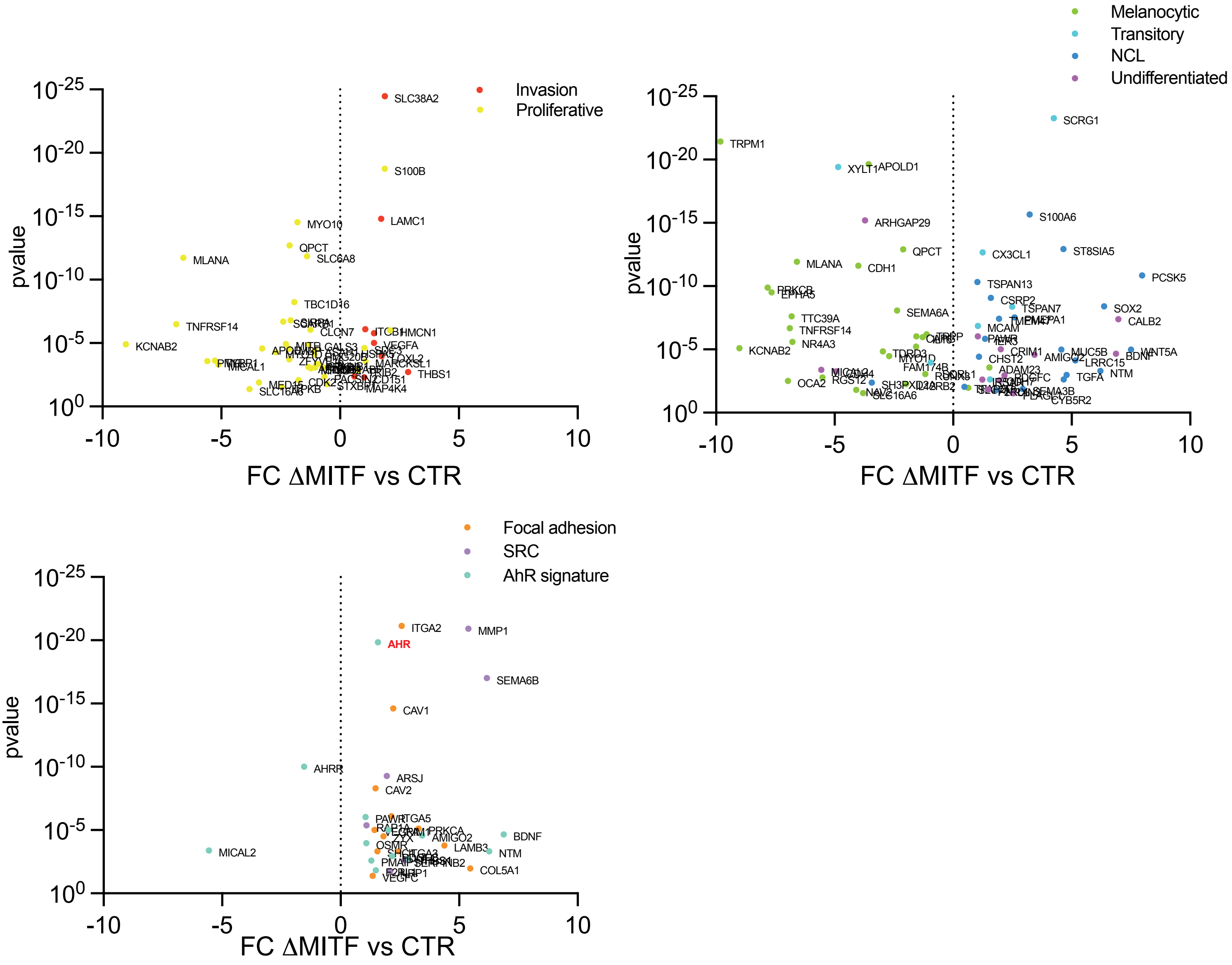

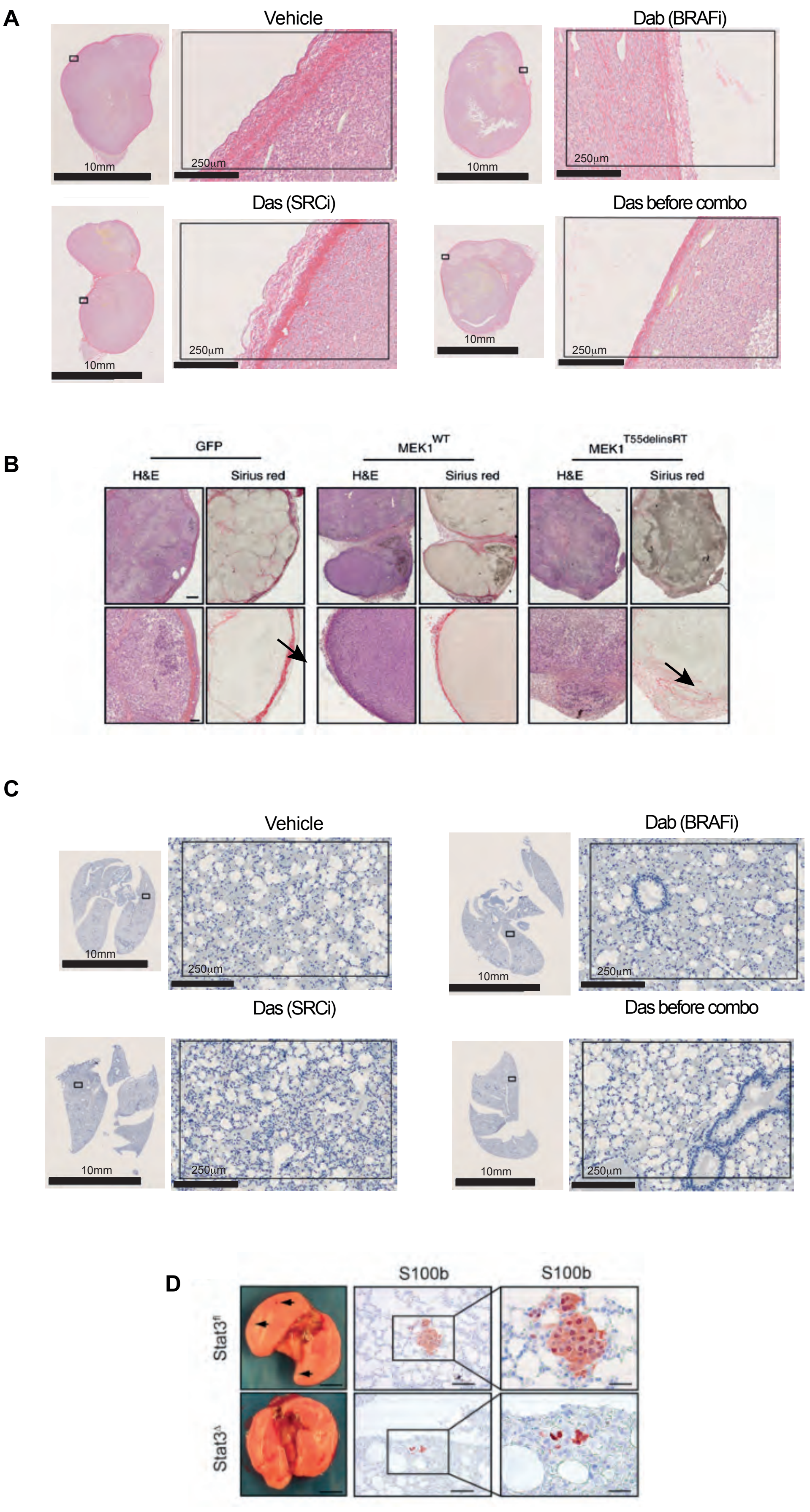

Immunohistochemistry for Sirius Red (staining for collagen) on Mel006R PDX tumors from all groups, to identify grade of demarcation and local invasion **(A)**. Illustration of local invasion of A375 melanoma cells after xenografts in mice (from Fig. 5D Kemper et al. 2015) **(B)**. We didn't observe local invasion with this Mel006R in all different groups nor the presence of metastatic melanoma cells in lung of mice **(C)** characterized by melanoma specific S100B immunostainning **(D,** picture form Fig3E, Swoboda et al. 2021**)**

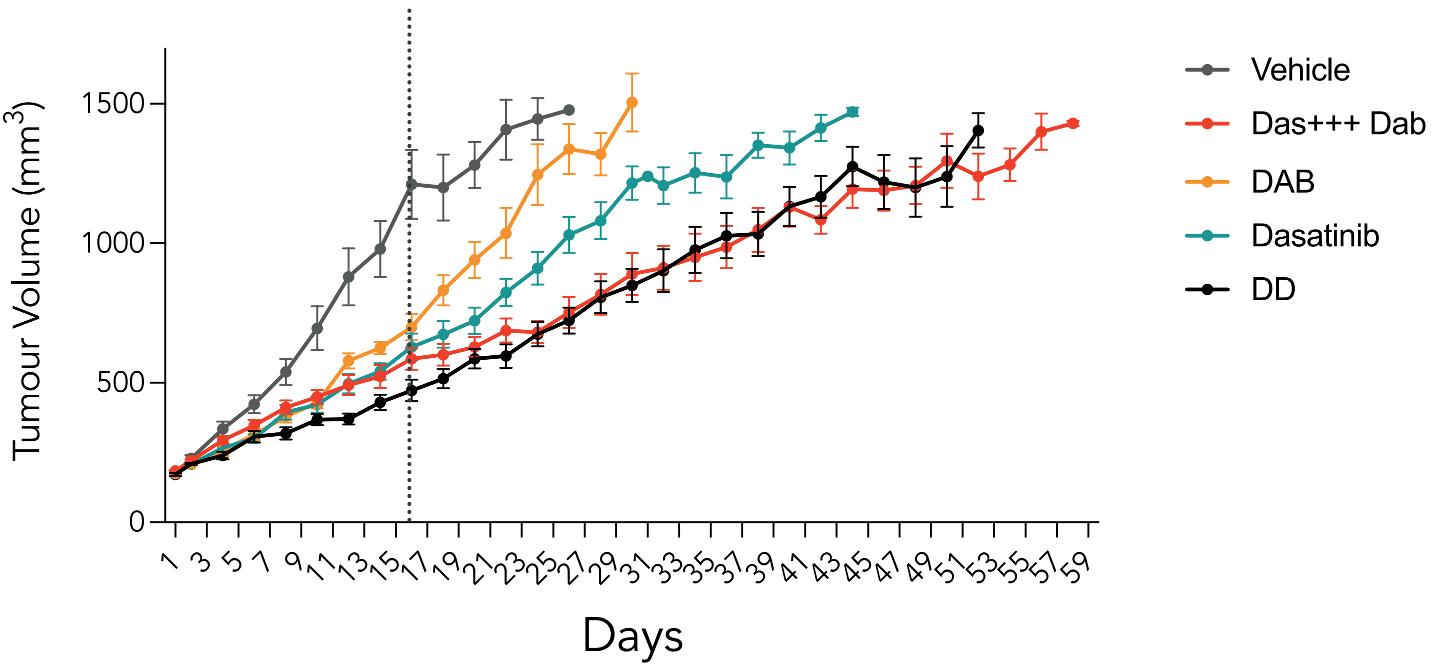
Vehicle
Das+++ Dab
DAB
Dasatinib
DD
Tumour Volume (mm^3)
0
500
1000
1500
1 3 5 7 9 11 13 15 17 19 21 23 25 27 29 31 33 35 37 39 41 43 45 47 49 51 53 55 57 59
Days

12th Sep 2022

Dear Dr. Corre,

Thank you for the submission of your revised manuscript to EMBO Molecular Medicine. I am pleased to inform you that we will be able to accept your manuscript pending the following final amendments:

1) Please implement the referees #2 and #3 suggestions and address referee #3 minor concerns.
2) In the main manuscript file, please do the following:
- Please correct callouts of supplementary tables and figures with corresponding Appendix Tables and Figures.
- In M&M, please include statement that the experiments using human samples conformed to the principles set out in the WMA Declaration of Helsinki and the Department of Health and Human Services Belmont Report. Please also amend it in the "Author Checklist" as well.
- In M&M, statistical paragraph should reflect all information that you have filled in the Authors Checklist, especially regarding randomization, blinding, replication.
- Please be aware that all datasets should be made freely available upon acceptance, without restriction. Please check "Author Guidelines" for more information. https://www.embopress.org/page/journal/17574684/authorguide#availabilityofpublishedmaterial
3) Appendix:
- Please merge all appendix tables with appendix figures, and references into one PDF. Update appendix ToC and correct the legend of Appendix Table 3, which is currently labeled as Appendix Table 4. Also, references should be in alphabetical order, where there are more than 10 authors on a paper, 10 will be listed, followed by "et al.".
- Please provide the antibody dilutions that were used for each antibody listed in Appendix Table 5.
4) Synopsis: Please check your synopsis text and image before submission with your revised manuscript. Please be aware that in the proof stage minor corrections only are allowed (e.g., typos).
5) For more information: This space should be used to list relevant web links for further consultation by our readers. Could you identify some relevant ones and provide such information as well? Some examples are patient associations, relevant databases, OMIM/proteins/genes links, author's websites, etc...
6) Source data: We encourage you to include the source data for figure panels that show essential data. Numerical data should be provided as individual .xls or .csv files (including a tab describing the data). For blots or microscopy, uncropped images should be submitted (using a zip archive if multiple images need to be supplied for one panel). For the main figures please upload one file per figure, while all EV figures should be zipped and uploaded as one file. Please check "Author Guidelines" for more information. https://www.embopress.org/page/journal/17574684/authorguide#sourcedata
7) As part of the EMBO Publications transparent editorial process initiative (see our Editorial at http://embomolmed.embopress.org/content/2/9/329), EMBO Molecular Medicine will publish online a Review Process File (RPF) to accompany accepted manuscripts. This file will be published in conjunction with your paper and will include the anonymous referee reports, your point-by-point response and all pertinent correspondence relating to the manuscript. Let us know whether you agree with the publication of the RPF and as here, if you want to remove or not any figures from it prior to publication. Please note that the Authors checklist will be published at the end of the RPF.
8) Please provide a point-by-point letter INCLUDING my comments as well as the reviewer's reports and your detailed responses (as Word file).

I look forward to reading a new revised version of your manuscript as soon as possible.

Yours sincerely,

Zeljko Durdevic

Zeljko Durdevic
Editor
EMBO Molecular Medicine

***** Reviewer's comments *****

Referee #1 (Remarks for Author):

the authors made strong efforts to meet most of my concerns. The paper is much stronger and to my opinion, can be published as that in EMBO Molecular Medicine.

Referee #2 (Remarks for Author):

The authors have addressed my earlier points. The manuscript is now much improved in terms of clarity and presentation.

Minor comments:

Page 6, line 169: Something is missing in the sentence „Conversely, we showed...."

Page 6, line 174: „...melanoma cell lines BEFORE AND AFTER..." After or not is not good english - this same phrasing is found in several places and needs to be amended.

Figure 3A is a Venn-diagram but the numbers discussed on page 7, line 201 are not seen in the figure.

Page 7, line 213: It is not clear what the authors mean by Fig 3A, right panel.

Page 9, line 253: „..cells expressing or LACKING AhR..."

Page 10, line 282: „...performed on SK28 cells BEFORE or AFTER knocking out AhR...."

Page 11,, line 324: „...on SK28R cells BEFORE OR after treatment with Bos...."

Page 11, line 330: „...THE PDX line MEL0600R is a BRAF v600e MUTANT cutaneous melanoma derived from MEL006....."

Referee #3 (Comments on Novelty/Model System for Author):

Authors stained the PDXs tissues as suggested in the previous revision. However, as explained below, these stainings should be quantified to account for variability between mice

Referee #3 (Remarks for Author):

Authors have clarified most of my and the other reviewer's comments. However, there are still a couple of issues that should be addressed.

1. Regarding local invasion from the primary tumour, in Informative Figure for reviewers2-3 authors show stainings for picosirius red and conclude that they did not observe any local invasion of tumour cells.
However, in my opinion the images in panel A show less red picosirius red staining in Dab(BRAFi) and "Das before combo" groups compared to Vehicle and Das only groups. This suggests more disrupted and/or disorganized ECM under treatment, which could be due, among other reasons, to more tumour invasion. A proper quantification including all tumours would clarify this.

2. p-SRC and Ahr stainings (Figure EV4B). In the point-by-point response, authors state that "BRAFi treatment alone didn't prevent this phosphorylation (Figure EV4)." However, from the picture shown I would say that there is less pink in BRAFi than in vehicle (although the change is smaller than with SRCi, as expected). In my opinion, proper quantification of these stainings (Ahr, p-SRC) should be included.

Both 1 and 2 would support that the mechanisms described in the paper could be operative in vivo.

3. Unless used for other purposes, I suggest that info on combination BRAFi+dasatinib from start of treatment (informative figure for reviewer #3) is mentioned/shown in the manuscript.

I would also include, perhaps in methods, some explanation (from point-by-point, page 9) for the lack of metastasis in this PDX model in this specific setting. This information may be useful for other researchers using this model and/or building up on this work.

Referee #1 (Remarks for Author):

the authors made strong efforts to meet most of my concerns. The paper is much stronger and to my opinion, can be published as that in EMBO Molecular Medicine.

We thank the referee for the trust placed in this work.

Referee #2 (Remarks for Author):

The authors have addressed my earlier points. The manuscript is now much improved in terms of clarity and presentation.

We thank the referee for the trust placed in this work and for the minor comments that have been now corrected in the main text.

Minor comments:

Page 6, line 169: Something is missing in the sentence „Conversely, we showed...."

This has been corrected in the text : "Conversely, we showed that AhR transcription factor participates in BRAFi resistance" (L166-167).

Page 6, line 174: „...melanoma cell lines BEFORE AND AFTER..." After or not is not good english - this same phrasing is found in several places and needs to be amended.

Many thanks for this comment. This has been corrected in the text (L172, L279, L594, L675).

Figure 3A is a Venn-diagram but the numbers discussed on page 7, line 201 are not seen in the figure.

Figure 3A has been modified to add the different numbers corresponding to each condition of the Venn diagram. The detail of this diagram can be found in figure Appendix Figure S2A.

Page 7, line 213: It is not clear what the authors mean by Fig 3A, right panel.

This is in fact Fig 3B, "right panel", this has been removed in the text (L211). It corresponds to Tsoi et al. dataset.

Page 9, line 253: „..cells expressing or LACKING AhR..."

This has been modified in the text (L250).

Page 10, line 282: „...performed on SK28 cells BEFORE or AFTER knocking out AhR...."

This has been modified in the text (L279).

Page 11,, line 324: „...on SK28R cells BEFORE OR after treatment with Bos...."

This has been modified in the text (L320).

Page 11, line 330: „...THE PDX line MEL0600R is a BRAF v600e MUTANT cutaneous melanoma derived from MEL006....."

This has been modified in the text (L327-329).

Referee #3 (Comments on Novelty/Model System for Author):

Authors stained the PDXs tissues as suggested in the previous revision. However, as explained below, these stainings should be quantified to account for variability between mice

Referee #3 (Remarks for Author):

Authors have clarified most of my and the other reviewer's comments. However, there are still a couple of issues that should be addressed.

We particularly thank reviewer 3 for the various comments and suggestions offered to clarify the last questions. In particular, we carried out the quantification of the immunostaining of the various PDX tumors using ImageJ (Fiji).

1. Regarding local invasion from the primary tumour, in Informative Figure for reviewers2-3 authors show stainings for picosirius red and conclude that they did not observe any local invasion of tumour cells.
However, in my opinion the images in panel A show less red picosirius red staining in Dab(BRAFi) and "Das before combo" groups compared to Vehicle and Das only groups. This suggests more disrupted and/or disorganized ECM under treatment, which could be due, among other reasons, to more tumour invasion. A proper quantification including all tumours would clarify this.

Thank you for this remark. Indeed, the images presented in particular in the Dab and Das before combo conditions (Figure for reviewer 2-3 A) do not seem the most representative. We first carried out the integration of the signal subtracted from the background noise. This allows to clearly delineate the edge of tumors that are well marked and not diffuse for the different treatment conditions. We also performed the measurement of the edge thickness at several positions of different tumors (n=4) for each tumor condition (PDX) (informative figure for reviewer #2 and #3 A). The edge thickness for the different tumors is relatively homogeneous overall with no significant difference between the groups (vehicle, Dab, Das or Das before combo). We did not notice any local invasion of cells at the margin of the tumors since the demarcations were well defined by the stromal tissue of mainly collagen (stained by Sirius red histochemistry).

2. p-SRC and Ahr stainings (Figure EV4B). In the point-by-point response, authors state that "BRAFi treatment alone didn't prevent this phosphorylation (Figure EV4)." However, from the picture shown I would say that there is less pink in BRAFi than in vehicle (although the change is smaller than with SRCi, as expected). In my opinion, proper quantification of these stainings (Ahr, p-SRC) should be included.

As recommended by reviewer 3, we performed a semi-quantitative analysis of the different immunostainings using imageJ (Fiji). The integration of the positive signal subtracted from the background noise first made it possible to measure the percentage of positive zones in each tumor. Thanks to this analysis we confirmed that treatment with Das specifically inhibit p-SRC in the tumor with only residual positive area (5-10%). Then, the measurement of the optical density for a set of fields for different tumors (n=4) this after calibration of the image using step tablet (Fiji), allowed us to confirm again that Das significantly prevent p-SRC in the tumor. Conversely, single treatment with BRAFi slightly increased the level of AhR and p-SRC.
The information for semi-quantitative quantification of IHC immunostaining has been implemented in the legend of Figure EV4D (L866-L871): “Quantification of % of positive immunostaining area has been performed using ImageJ (Fiji) after integration of specific signal. Semi-quantitative analysis of optical density for several tumors for each group (n=4) has been performed after calibration of the image using step tablet (Fiji).” A new version of Figure EV4D is now provided.

Both 1 and 2 would support that the mechanisms described in the paper could be operative in vivo.

3. Unless used for other purposes, I suggest that info on combination BRAFi+dasatinib from start of treatment (informative figure for reviewer #3) is mentioned/shown in the manuscript.

As proposed by reviewer 3, we have added in Appendix Figure S9 the data concerning the BRAFi + dasatinib combination from the start of treatment. Indeed, the BRAFi + Dasatinib combination (Appendix Figure S9, black) from the start of treatment seemed to be as efficient than the pretreatment with Das for 16 days before combo (Appendix Figure S9, red) to slow down tumor growth. These results are informative regarding the therapeutic strategy that can be used in the patient. The addition of SRC inhibitor from the start of treatment did not seem to disturb the response to BRAFi and would make it possible to slow down the tumor progression of resistant melanomas. This information has been implemented in the main manuscript (L338-340):
“Remarkably, introducing BRAFi, 16 days post treatment with SRCi (Fig 6A) or by combining BRAFi and dasatinib from the start of treatment (Appendix Fig S9) significantly diminished the growth rate of the BRAFi-resistant PDX tumor.”

I would also include, perhaps in methods, some explanation (from point-by-point, page 9) for the lack of metastasis in this PDX model in this specific setting. This information may be useful for other researchers using this model and/or building up on this work.

Some of the explanation for the lack of metastasis in this PDX model have been implemented in methods section of the main text (L577-579) :

"Because tumor growth of the BRAFi-resistant PDX model (Mel006R) is very fast, this model is probably not appropriate to study the mechanisms of invasion and metastasis at least in the time window analyzed (20 to 60 days)."

A Transwell assays (D4) related to figure 1D

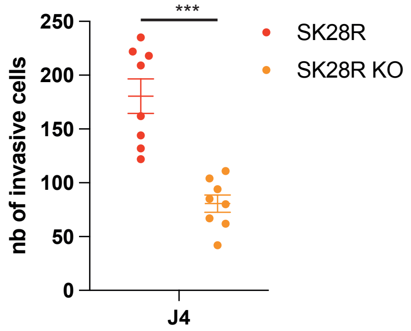

B related to Fig sup 1A

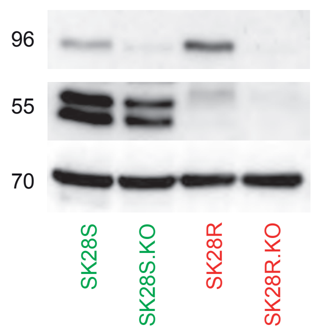

C related to Fig 2A

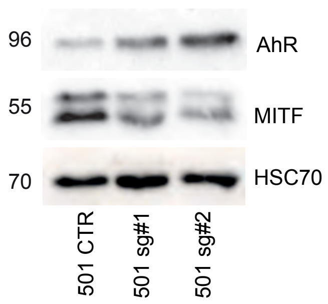

D Dilshat et al. 2021 elife

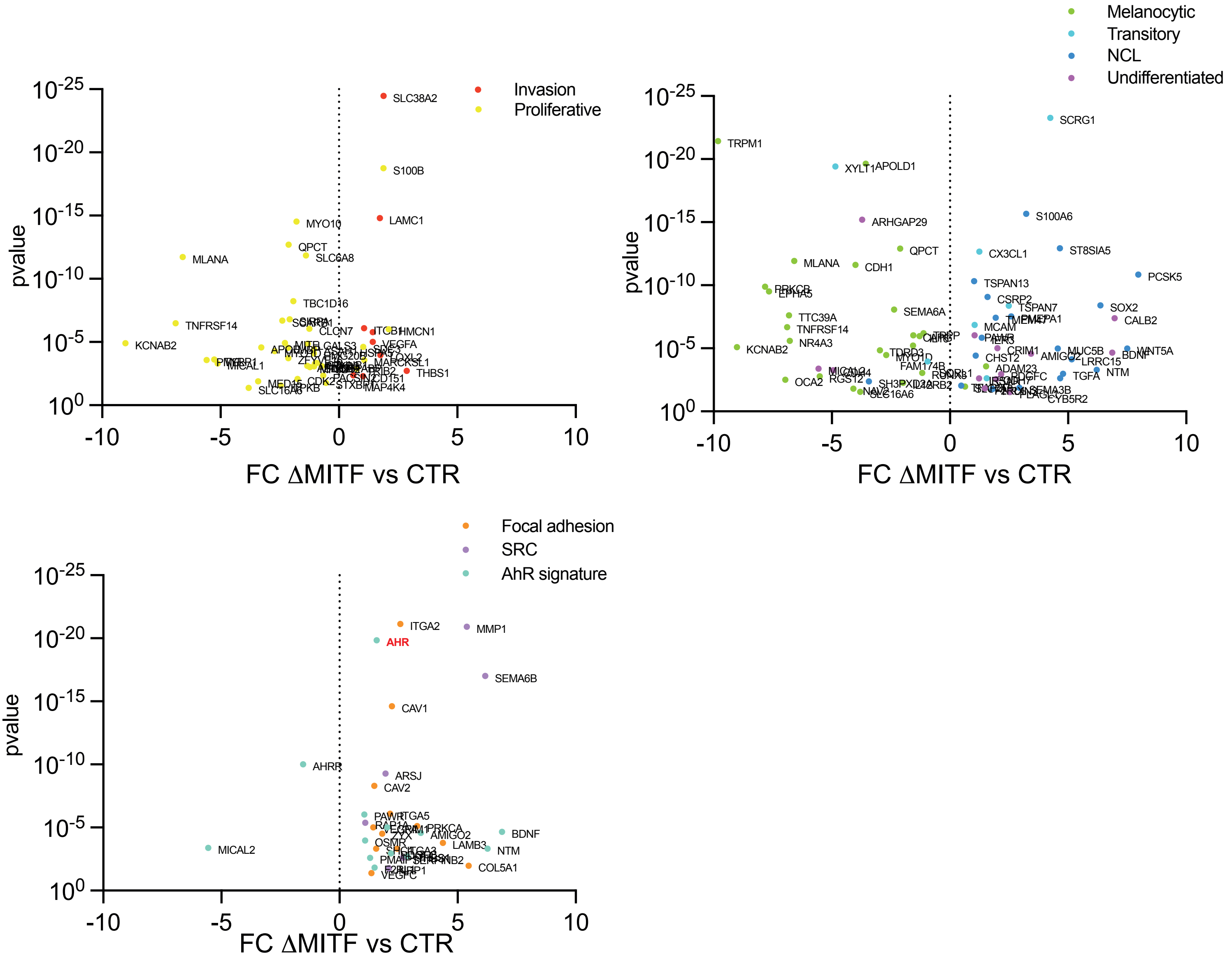

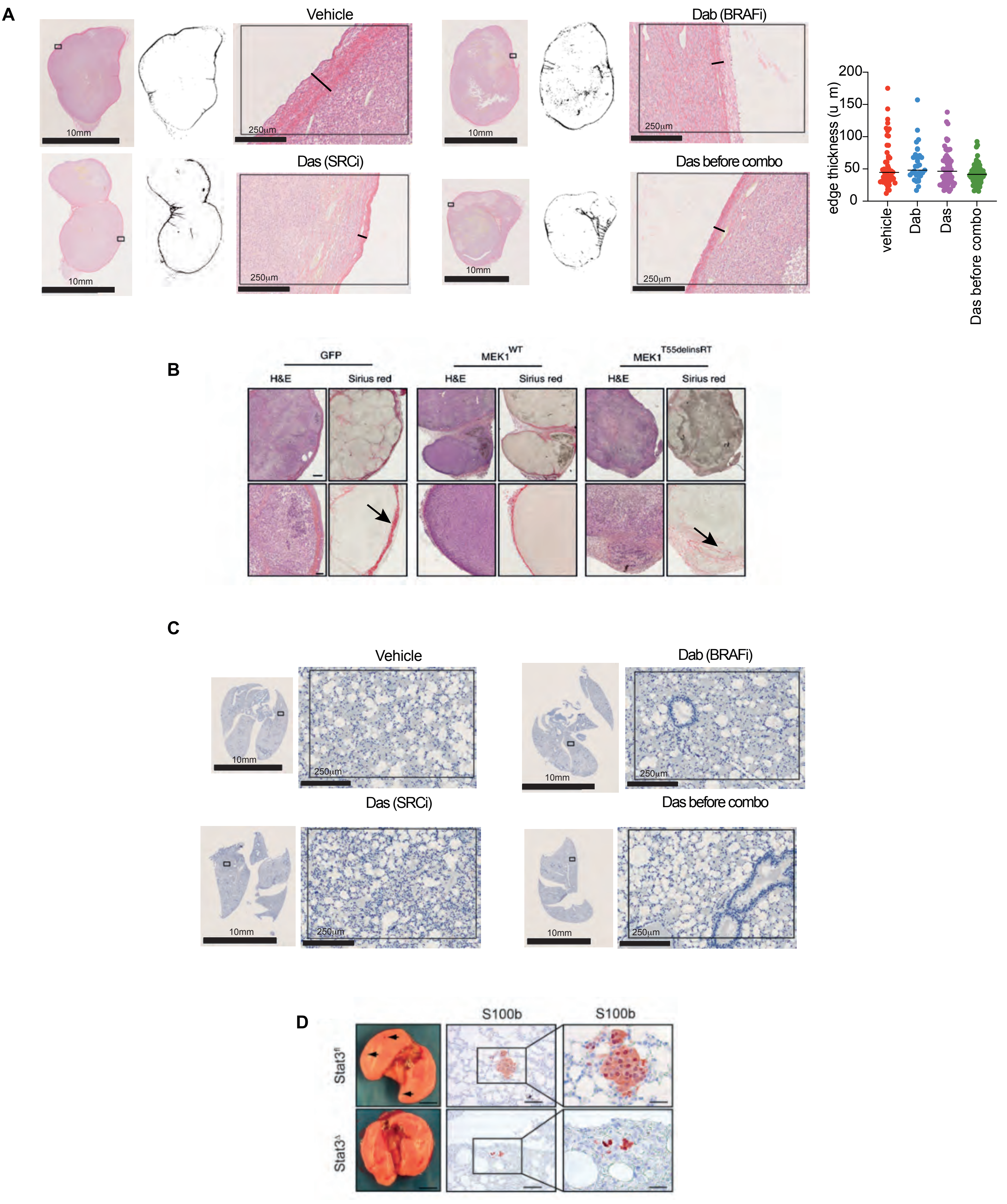

Immunohistochemistry for Sirius Red (staining for collagen) on Mel006R PDX tumors from all groups, to identify grade of demarcation and local invasion **(A)**. Illustration of local invasion of A375 melanoma cells after xenografts in mice (from Fig. 5D Kemper et al. 2015) **(B)**. We didn't observe local invasion with this Mel006R in all different groups nor the presence of metastatic melanoma cells in lung of mice **(C)** characterized by melanoma specific S100B immunostainning **(D,** picture form Fig3E, Swoboda et al. 2021**)**

2nd Revision - Editorial Decision 30th Sep 2022

We are pleased to inform you that your manuscript is accepted for publication and is now being sent to our publisher to be included in the next available issue of EMBO Molecular Medicine.

EMBO Press Author Checklist

Corresponding Author Name: Sébastien CORRE	
Journal Submitted to: Embo Molecular Medecine	
Manuscript Number: EMM-2022-15677-V2	

USEFUL LINKS FOR COMPLETING THIS FORM
The EMBO Journal - Author Guidelines
EMBO Reports - Author Guidelines
Molecular Systems Biology - Author Guidelines
EMBO Molecular Medicine - Author Guidelines

Reporting Checklist for Life Science Articles (updated January 2022)
This checklist is adapted from Materials Design Analysis Reporting (MDAR) Checklist for Authors. MDAR establishes a minimum set of requirements in transparent reporting in the life sciences (see Statement of Task: 10.31222/osf.io/9sm4x). Please follow the journal's guidelines in preparing your manuscript.
Please note that a copy of this checklist will be published alongside your article.

Abridged guidelines for figures
1. Data
The data shown in figures should satisfy the following conditions:
- the data were obtained and processed according to the field's best practice and are presented to reflect the results of the experiments in an accurate and unbiased manner.
- ideally, figure panels should include only measurements that are directly comparable to each other and obtained with the same assay.
- plots include clearly labeled error bars for independent experiments and sample sizes. Unless justified, error bars should not be shown for technical replicates.
- if n<5, the individual data points from each experiment should be plotted. Any statistical test employed should be justified.
- Source Data should be included to report the data underlying figures according to the guidelines set out in the authorship guidelines on Data Presentation.

2. Captions
Each figure caption should contain the following information, for each panel where they are relevant:
- a specification of the experimental system investigated (eg cell line, species name).
- the assay(s) and method(s) used to carry out the reported observations and measurements.
- an explicit mention of the biological and chemical entity(ies) that are being measured.
- an explicit mention of the biological and chemical entity(ies) that are altered/varied/perturbed in a controlled manner.
- the exact sample size (n) for each experimental group/condition, given as a number, not a range;
- a description of the sample collection allowing the reader to understand whether the samples represent technical or biological replicates (including how many animals, litters, cultures, etc.).
- a statement of how many times the experiment shown was independently replicated in the laboratory.
- definitions of statistical methods and measures:
  - common tests, such as t-test (please specify whether paired vs. unpaired), simple χ2 tests, Wilcoxon and Mann-Whitney tests, can be unambiguously identified by name only, but more complex techniques should be described in the methods section;
  - are tests one-sided or two-sided?
  - are there adjustments for multiple comparisons?
  - exact statistical test results, e.g., P values = x but not P values < x;
  - definition of 'center values' as median or average;
  - definition of error bars as s.d. or s.e.m.

Please complete ALL of the questions below.
Select "Not Applicable" only when the requested information is not relevant for your study.

Materials

Newly Created Materials	Information included in the manuscript?	In which section is the information available? (Reagents and Tools Table, Materials and Methods, Figures, Data Availability Section)
New materials and reagents need to be available; do any restrictions apply?	Not Applicable	

Antibodies	Information included in the manuscript?	In which section is the information available? (Reagents and Tools Table, Materials and Methods, Figures, Data Availability Section)
For **antibodies** provide the following information:<br>- Commercial antibodies: RRID (if possible) or supplier name, catalogue number and or/clone number<br>- Non-commercial: RRID or citation	Yes	Supplemented tables Table EV7

DNA and RNA sequences	Information included in the manuscript?	In which section is the information available? (Reagents and Tools Table, Materials and Methods, Figures, Data Availability Section)
**Short novel DNA or RNA including primers, probes**: provide the sequences.	Yes	Supplemented tables Table EV6

Cell materials	Information included in the manuscript?	In which section is the information available? (Reagents and Tools Table, Materials and Methods, Figures, Data Availability Section)
**Cell lines:** Provide species information, strain. Provide accession number in repository **OR** supplier name, catalog number, clone number, and/**OR** RRID.	Yes	Material and Methods
**Primary cultures:** Provide species, strain, sex of origin, genetic modification status.	Not Applicable	
Report if the cell lines were recently **authenticated** (e.g., by STR profiling) and tested for mycoplasma contamination.	Not Applicable	

Experimental animals	Information included in the manuscript?	In which section is the information available? (Reagents and Tools Table, Materials and Methods, Figures, Data Availability Section)
**Laboratory animals or Model organisms:** Provide species, strain, sex, age, genetic modification status. Provide accession number in repository **OR** supplier name, catalog number, clone number, **OR** RRID.	Yes	Material and Methods
**Animal observed in or captured from the field:** Provide species, sex, and age where possible.	Not Applicable	
Please detail **housing and husbandry conditions**.	Yes	Material and Methods

Plants and microbes	Information included in the manuscript?	In which section is the information available? (Reagents and Tools Table, Materials and Methods, Figures, Data Availability Section)
**Plants:** provide species and strain, ecotype and cultivar where relevant, unique accession number if available, and source (including location for collected wild specimens).	Not Applicable	
**Microbes:** provide species and strain, unique accession number if available, and source.	Not Applicable	

Human research participants	Information included in the manuscript?	In which section is the information available? (Reagents and Tools Table, Materials and Methods, Figures, Data Availability Section)
If collected and within the bounds of privacy constraints report on age, sex and gender or ethnicity for all study participants.	Not Applicable	

Core facilities	Information included in the manuscript?	In which section is the information available? (Reagents and Tools Table, Materials and Methods, Figures, Data Availability Section)
If your work benefited from core facilities, was their service mentioned in the acknowledgments section?	Not Applicable	

Design

Study protocol	Information included in the manuscript?	In which section is the information available? (Reagents and Tools Table, Materials and Methods, Figures, Data Availability Section)
If study protocol has been **pre-registered, provide DOI in the manuscript**. For clinical trials, provide the trial registration number **OR** cite DOI.	Not Applicable	
Report the **clinical trial registration number** (at ClinicalTrials.gov or equivalent), where applicable.	Not Applicable	

Laboratory protocol	Information included in the manuscript?	In which section is the information available? (Reagents and Tools Table, Materials and Methods, Figures, Data Availability Section)
Provide DOI OR other citation details if **external detailed step-by-step protocols** are available.	Not Applicable	

Experimental study design and statistics	Information included in the manuscript?	In which section is the information available? (Reagents and Tools Table, Materials and Methods, Figures, Data Availability Section)
Include a statement about **sample size** estimate even if no statistical methods were used.	Yes	Figures
Were any steps taken to minimize the effects of subjective bias when allocating animals/samples to treatment (e.g. **randomization procedure**)? If yes, have they been described?	Not Applicable	
Include a statement about **blinding** even if no blinding was done.	Not Applicable	
Describe **inclusion/exclusion criteria** if samples or animals were excluded from the analysis. Were the criteria pre-established?<br><br>If sample or data points were omitted from analysis, report if this was due to attrition or intentional exclusion and provide justification.	Not Applicable	
For every figure, are **statistical tests** justified as appropriate? Do the data meet the assumptions of the tests (e.g., normal distribution)? Describe any methods used to assess it. Is there an estimate of variation within each group of data? Is the variance similar between the groups that are being statistically compared?	Yes	Material and Methods

Sample definition and in-laboratory replication	Information included in the manuscript?	In which section is the information available? (Reagents and Tools Table, Materials and Methods, Figures, Data Availability Section)
In the figure legends: state number of times the experiment was **replicated** in laboratory.	Yes	Figures
In the figure legends: define whether data describe **technical or biological replicates**.	Not Applicable	

Ethics

Ethics	Information included in the manuscript?	In which section is the information available? (Reagents and Tools Table, Materials and Methods, Figures, Data Availability Section)
Studies involving **human participants**: State details of **authority granting ethics approval** (IRB or equivalent committee(s), provide reference number for approval.	Not Applicable	
Studies involving **human participants**: Include a statement confirming that **informed consent** was obtained from all subjects and that the experiments conformed to the principles set out in the WMA Declaration of Helsinki and the Department of Health and Human Services Belmont Report.	Not Applicable	
Studies involving **human participants:** For publication of **patient photos**, include a statement confirming that consent to publish was obtained.	Not Applicable	
Studies involving experimental **animals**: State details of **authority granting ethics approval** (IRB or equivalent committee(s), provide reference number for approval. Include a statement of compliance with ethical regulations.	Yes	Material and Methods
Studies involving **specimen and field samples:** State if relevant **permits** obtained, provide details of authority approving study; if none were required, explain why.	Not Applicable	

Dual Use Research of Concern (DURC)	Information included in the manuscript?	In which section is the information available? (Reagents and Tools Table, Materials and Methods, Figures, Data Availability Section)
Could your study fall under dual use research restrictions? Please check biosecurity documents and list of **select agents and toxins** (CDC): https://www.selectagents.gov/sat/list.htm	Not Applicable	
If you used a select agent, is the security level of the lab appropriate and reported in the manuscript?	Not Applicable	
If a study is subject to dual use research of concern regulations, is the name of the **authority granting approval and reference number** for the regulatory approval provided in the manuscript?	Not Applicable	

Reporting

The MDAR framework recommends adoption of discipline-specific guidelines, established and endorsed through community initiatives. Journals have their own policy about requiring specific guidelines and recommendations to complement MDAR.

Adherence to community standards	Information included in the manuscript?	In which section is the information available? (Reagents and Tools Table, Materials and Methods, Figures, Data Availability Section)
State if relevant guidelines or checklists (e.g., **ICMJE, MIBBI, ARRIVE, PRISMA**) have been followed or provided.	Not Applicable	
For **tumor marker prognostic studies,** we recommend that you follow the **REMARK** reporting guidelines (see link list at top right). See author guidelines, under 'Reporting Guidelines'. Please confirm you have followed these guidelines.	Not Applicable	
For **phase II and III randomized controlled trials**, please refer to the **CONSORT** flow diagram (see link list at top right) and submit the CONSORT checklist (see link list at top right) with your submission. See author guidelines, under 'Reporting Guidelines'. Please confirm you have submitted this list.	Not Applicable	

Data Availability

Data availability	Information included in the manuscript?	In which section is the information available? (Reagents and Tools Table, Materials and Methods, Figures, Data Availability Section)
Have **primary datasets** been deposited according to the journal's guidelines (see 'Data Deposition' section) and the respective accession numbers provided in the Data Availability Section?	Yes	Material and Methods
Were **human clinical and genomic datasets** deposited in a public access-controlled repository in accordance to ethical obligations to the patients and to the applicable consent agreement?	Yes	Material and Methods
Are **computational models** that are central and integral to a study available without restrictions in a machine-readable form? Were the relevant accession numbers or links provided?	Not Applicable	
If publicly available data were reused, provide the respective **data citations in the reference list**.	Not Applicable	
